# One-Pot, Multi-Component Green Microwave-Assisted Synthesis of Bridgehead Bicyclo[4.4.0]boron Heterocycles and DNA Affinity Studies †

**DOI:** 10.3390/ijms25189842

**Published:** 2024-09-12

**Authors:** Polinikis Paisidis, Maroula G. Kokotou, Antigoni Kotali, George Psomas, Konstantina C. Fylaktakidou

**Affiliations:** 1 Laboratory of Organic Chemistry, Department of Chemistry, Aristotle University of Thessaloniki, 54124 Thessaloniki, Greece; ppaisidis@chem.auth.gr; 2 Laboratory of Chemistry, Department of Food Science and Human Nutrition, Agricultural University of Athens, Iera Odos 75, 11855 Athens, Greece; mkokotou@aua.gr; 3 Laboratory of Organic Chemistry, Department of Chemical Engineering, Aristotle University of Thessaloniki, 54124 Thessaloniki, Greece; kotali@cheng.auth.gr; 4 Laboratory of Inorganic Chemistry, Department of Chemistry, Aristotle University of Thessaloniki, 54124 Thessaloniki, Greece; gepsomas@chem.auth.gr

**Keywords:** boron heterocycles, multi-component reaction, one-pot reaction, microwave irradiation, green synthesis, DNA interaction

## Abstract

Anthranilic acids, salicylaldehydes and arylboronic acids reacted in EtOH/H_2_O (1/3) at 150 °C under microwave irradiation for 1 h to give, in excellent yields and purity, twenty-three bridgehead bicyclo[4.4.0]boron heterocycles via one-pot, three-component green synthesis. The scope and the limitations of the reactions are discussed in terms of the substitution of ten different anthranilic acids, three salicylaldehydes and three arylboronic acids. The replacement of salicylaldehyde with *o*-hydroxyacetophenone demanded a lipophilic solvent for the reaction to occur. Eight novel derivatives were isolated following crystallization in a toluene-containing mixture that included molecular sieves. The above one-pot, three-component reactions were completed under microwave irradiation at 180 °C within 1.5 h, thus avoiding the conventional prolonged heating reaction times and the use of a Dean–Stark apparatus. All derivatives were studied for their affinity to calf thymus DNA using proper techniques like viscosity and UV–vis spectroscopy, where DNA-binding constants were found in the range 2.83 × 10^4^–8.41 × 10^6^ M^−1^. Ethidium bromide replacement studies using fluorescence spectroscopy indicated Stern–Volmer constants between 1.49 × 10^4^ and 5.36 × 10^4^ M^−1^, whereas the corresponding quenching constants were calculated to be between 6.46 × 10^11^ and 2.33 × 10^12^ M^−1^ s^−1^. All the above initial experiments show that these compounds may have possible medical applications for DNA-related diseases.

## 1. Introduction

**Figure 1:**
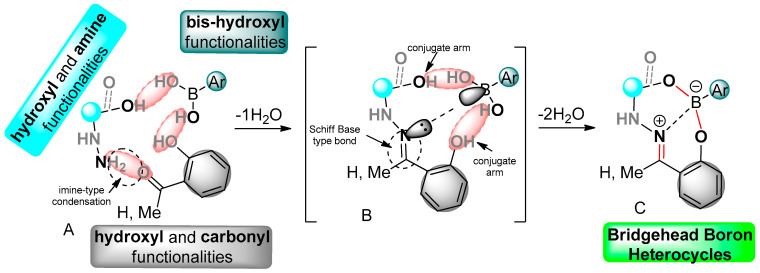
Triad of compatible functional pairs for the synthesis of bridgehead boron heterocycles. Groups or atoms in gray color indicate moieties that may be part of the molecule or not. For example, hydroxyl and amine functionalities may include a phenolic or alcoholic OH (no C=O) or an acid (C=O)OH. The amine group might be NH_2_ only or be part of a hydrazine (NHNH_2_) or hydrazone (=NNH_2_) residue. (Comments on (**A**): The aromatic ring at hydroxyl and carbonyl functionalities might be a phenyl group or an aryl group and is specified to only show SAs and *o*-hydroxyacetophenones, which are the compounds used in the vast majority (if not all) of the literature examples. Comments on (**B**): The order of the reactions can be different in the three-component reaction. However, reactions of such Schiff bases with ABAs exist in the literature and have also been performed in this manuscript in a certain experiment. Structure (**C**) represents the Bridgehead Boron Heterocycles which are formed due to the overlap of the lone pair electrons of the nitrogen with the electron-free Boron *p*-bond.

B-N and B-O bridgehead boron heterocycles are compounds that are produced, more often than not, via the assembly of versatile materials that possess reactive functionalities with the capacity to condense and/or dehydrate to one another [1,2,3,4,5,6,7]. For complex materials, in order for an assembly plan of a three-component reaction to occur, all three counterparts should be bis-functional and able to provide compatible couples with one another within the triplet (Figure 1A). In such boron heterocycles, the boron-containing partners are arylboronic acids (ABAs) due to the variety of such compounds that are commercially and readily available from the market. Furthermore, ABAs allow for the potential derivatization of their aromatic ring so as to carry pharmacophores and/or fluorophores [4,8,9,10,11,12,13]. The two hydroxyl groups, attached on the boron atom, are able to react with alcoholic, carboxylic and phenolic hydroxyl groups and form relatively stable esteric bonds. Within this context, ideal partners of ABAs are salicylaldehydes (SAs) or 2-OH-acetophenones that possess, in vicinal positions, the couple of groups “hydroxyl” and “carbonyl” and anthranilic acids or other derivatives that contain, in proper positions, “hydroxyl” and “amine” functional groups (Figure 1A).

SAs or 2-OH-acetophenones provide the C=O moiety for condensation with the amine of the amine- and OH-containing bis-functional individual (Figure 1A, hydroxyl and carbonyl functionalities and hydroxyl and amine functionalities, respectively) so as to provide an “imine-type” conjugate, in certain cases named the “Schiff base” (Figure 1B). Continuously, the dehydration of the ABA’s two hydroxyl groups with the Schiff base conjugate’s two arms, which sequences the condensation reaction, removes two more water molecules to give the bridgehead boron heterocycle (Figure 1B,C). The iminic nitrogen has its lone pair electrons in position to overlap with the electron-free Boron *p*-bond and to form a polycyclic structure, giving, depending on the rigidity of the final structure, potential applications [4].

**Figure 2:**
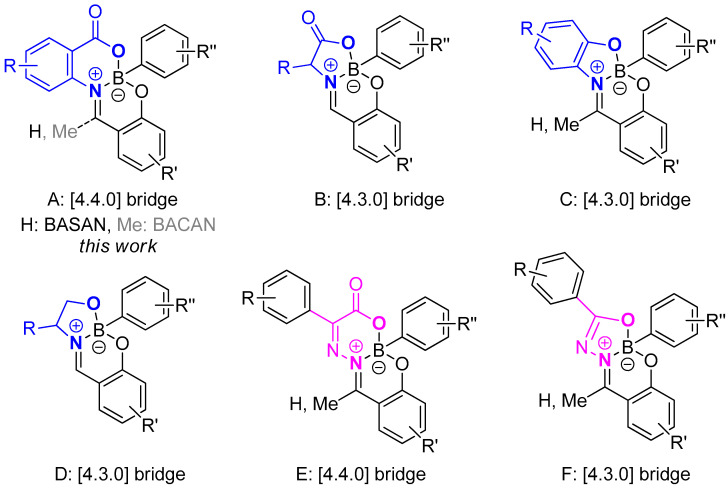
Bridgehead boron complex heterocycles: [4.4.0] [(**A**) (BASAN, BACAN—not known), (**E**)] and [4.3.0] (**B**–**D**,**F**). Blue color indicates counterparts with OH and NH_2_ functionalities and pink color with OH and =N-NH_2_ functionalities.

More specifically, in the literature, the counterparts of ABAs on such bridgehead boron heterocycles are SAs and anthranilic acids (AAs) that form [4.4.0] bridgeheads (Figure 2A, Boronic Acid Salicylidene ANthranylimine—BASAN) [4,9,14,15,16], with derivatives that are derived from *o*-hydroxyacetophenones being unknown (Figure 2A, Boronic Acid *o*-hydroxy aCetophenone ANthranylimine—BACAN). SAs and amino acids (Figure 2B) [4,6,9,17,18] as well as SAs and *o*-aminophenols (Figure 2C) [9,19], etc. (Figure 2D) [5,17,20], may all form [4.3.0] bridgeheads, whereas hydrazines provide, very often, salicylhydrazone residues aiming to form mixed azines (Figure 2E,F) [7,10,11,12,13,21] and other structures with similarities to the scaffolds that bridge boron in [4.4.0] and [4.3.0] structures, respectively [1,2,3].

The utility of such compounds varies from bioimaging to medicinal chemistry and organic synthesis. Thus, luminescence and optical studies have been performed for structures like A [15,16], C [9], E [4,10,11,12,13] and F [21] (Figure 2), where functionalization on the boron aryl moiety has been used as an important tool to enhance the fluorescence quantum yield as well as drug delivery. Derivatives of A, however, were not reported to exhibit high fluorescence quantum yields because the central atom adopts an out-of-plane tetrahedral geometry [14,15,16], contrary to the compounds of E [4,7,9]. As far as biological activity concerns, compounds of the structure B have been evaluated for their activity against human neutrophile elastase [17] and as phenylalanine hydroxylase modulators [18], and specific compounds bearing the E structure were found to be highly efficient singlet-oxygen photosensitizers that induce ferroptosis triggered by photodynamic therapy [22]. Very limited studies on their chemistry revealed that the iminic carbon was vulnerable to nucleophilic substitution by glutathione in cancer cells, which resulted in the decomposition of the compound and the release of a properly substituted boron fluorophore [9]. The same position also underwent an acetolysis reaction after heating for some hours in acetone [19]. The catalytic asymmetric transfer hydrogenation of aromatic ketones to high-value alcohols has been studied with boron compounds of structure D [5] (Figure 2).

DNA is the primary biological target for the discovery of therapeutic applications and studies in Molecular Biology. DNA interactions with small molecules are, thus, of immense importance [23,24,25,26,27] for the discovery of mainly anticancer [28] and antimicrobial agents [29]. The affinities to DNA may be studied with various spectroscopic techniques that include UV–vis and fluorescence spectroscopy [24] as well as viscosity experiments [30] in order to identify non-covalent interactions, such as electrostatic and intercalative interactions.

We are interested in the chemistry and biological activities of AA derivatives [31] in DNA affinity studies [32,33,34,35,36] as well as in boron chemistry [1,2,3]. BASAN complexes (Figure 2A) are assemblies of boron reagents with AAs, and their chemistry has attracted interest during the last decade with studies to deal with their efficient synthesis [4,9,14,15,16]. The most recent one is related to a natural product, the anthraquinone *o*-hydroxy-carbaldehyde “Damnacanthal” [16], as a counterpart of the triad. To the best of our knowledge, the assembly of AAs and ABAs with *o*-hydroxyacetophenones has not been studied yet. In a study concerning the stability, in plasma, of compounds C with H or Me on the imine group at pH 7.4, it was found that the Me group enhanced the stability of the compound to 39.8 h instead of 17.6 min for the derivative coming from SA [9]. In our plan, we have envisioned green one-pot synthesis for compounds of the general structure A (BASAN), the synthesis of their methyl analogues (BACAN), the exploration of their stability in solution and that of their calf thymus (CT) DNA-binding affinities in an effort to investigate their possible applications in diseases related to DNA.

## 2. Results

### 2.1. Chemistry

**Scheme 1:**
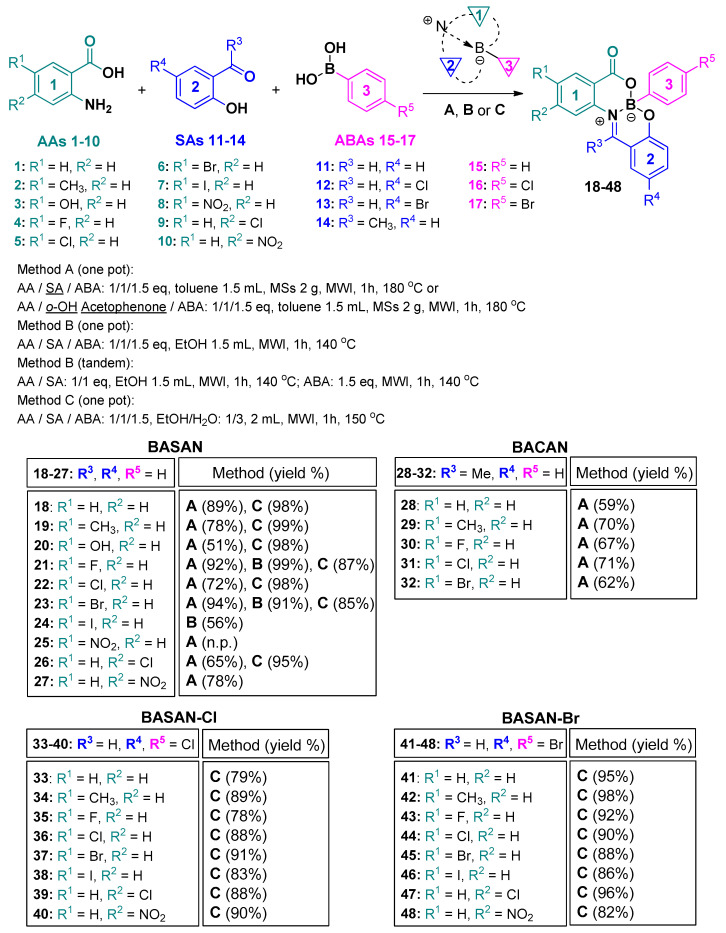
One-pot, three-component reaction for the synthesis of derivatives **18**–**48**. Combinations for all [4.4.0] bridgehead boron heterocycles; n.p. means not pure.

Conditions for the synthesis of the desired BASAN complexes under microwave irradiation (MWI) between AAs (**1**–**10**), SA (**11**) and phenylboronic acid (PBA) (**15**) in toluene at 180 °C for 1 h have been established (Scheme 1) in order to test a methodology that was going to avoid thermal conditions with the use of a Dean–Stark apparatus. Activated molecular sieves (MSs) were added within the mixture so as to absorb the produced water. All products were precipitated in toluene, filtered and washed with ethyl acetate (EA) to give BASAN compounds **18**–**27** in 78–94% yields, except for derivatives **20**, **24** and **26,** which were obtained in yields as low as 36, 49 and 55%, respectively (Method A). In addition, compound **25** was obtained as a precipitate; however, it was contaminated with some residues of intermediates, with the reaction not reaching completion, even after prolonged heating. Proton NMR (Supporting Information part 1, Section S.1) indicated the formation of the compound; however, the compound was unstable, and therefore, no purification method was suitable for it.

In comparison to the previously reported components of BASAN derivatives, scientists have emphasized SAs and ABAs more, keeping AAs either non-substituted or with a donor (OMe) in the *m*-position to the NH_2_. 5-Cl-AA has also been used previously. The existence of various substituents, however, in the *p*-position to the NH_2_, which is crucial for coupling with SAs, is a factor that, in our opinion, is worth extensive experimentation. Reviewing the syntheses of BASANs, the use of MWI in CCl_4_ for 3 min [14], MeOH reflux for 12 h [15], CH_3_CN reflux for 0.5 h [16] or EtOH reflux for 18 h [4] can be observed. The methodology presented herein under MWI used a greener solvent than CCl_4_; however, toluene still ranks low on the list of high-environmental-risk solvents [37].

Therefore, in order to offer a possibly improved methodology, other one-pot synthesis procedures under MWI have been investigated, focusing on green and environmentally benign approaches. The design afforded the replacement of toxic or unsafe solvents, prevention of waste, energy saving by decreasing the number of steps and reaction times and high reaction rates by using the eco-friendly MWI [38,39]. EtOH has been used instead (ranks second after 1-propanol in the list of the environmentally safer solvents [37]), and thus, the one-pot, three-component mixture reacted under MWI at 140 °C for 1 h to give products **21** and **23** in 99 and 91% yields, respectively (Method B).

These encouraging results allowed for experimentation with the use of mixtures of EtOH/H_2_O that, evidently, in a ratio of 1/3 at 150 °C for 1 h allowed for the fruitful production of derivatives **18**–**23** and **26** in excellent yields, 85–99% (Method C; Scheme 1). In this solvent system, all products were obtained as crystalline precipitates that needed no further purification. The only exception in this synthetic plan was compound **24**, which was produced via a MWI tandem reaction in EtOH (again green) that afforded the condensation of AA **7** and SA **11** for 1 h followed by the addition of phenylboronic acid and the irradiation of the mixture for 1 more h (56% yield for two steps, Method B, tandem). Unfortunately, neither of the above two Methods, B and C, was suitable for the synthesis of derivatives **25** and **27**, probably due to solubility problems. In general, in spite of the fact that Method A provided the desired products, the yields were lower than those of Methods B and C. In addition, the precipitate contained a percentage of starting materials and intermediates, albeit very small; however, those products needed purification.

To our delight, Method C gave, via filtration, extremely pure compounds that needed no further purification. In the filtrate, the only compound found was the excess ABA, which was found necessary for the reaction to go to completion. Therefore, BASAN-Cl complexes (**33**–**40**) (consisted of AAs **1**–**2**, **4**–**7**, **9**–**10**, 5-Cl-SA **12** and 4-Cl-PBA **16**) and BASAN-Br complexes (**41**–**48**) (consisted of AAs **1**–**2**, **4**–**7**, **9**–**10**, 5-Br-SA **13** and 4-Br-PBA **17**) were synthesized using Method C. Problems arose when AAs **3** and **8** were used, as the desired products were not formed, and only the starting materials were recovered. For these reactions, Method A also proved unsuccessful. In the case of AA **8**, the *p*-position of the NO_2_ to the NH_2_ group of AA in conjunction with the -I effect of the halogen atoms on **12** and **13** were destabilizing the system, and thus, any product formed decomposed readily. In the case of **3**, the Schiff base was the only product formed.

Methods C and B were not suitable for the condensation of *o*-hydroxy ketones with the aromatic amine group (BACAN synthesis), obviously due to solubility issues. Thus, the exchange of SA **11** to *o*-hydroxyacetophenone **14** required a more lipophilic reaction solvent. These one-pot, three-component reactions have been attempted for the first time, and it seems that the bulkiness of the methyl group played a role in preventing the completion of the reactions due to steric hindrance. Thus, neither 5-OH, 5-I, 5-NO_2_, 4-NO_2_ or 4-Cl AA (**3** and **7**–**10**, respectively) gave the desired products. However, the rest of the starting materials gave products **28**–**32** in 55–90% yields. The identification of compounds has been achieved via IR and NMR spectra and HRMS analysis. The data list with the analysis of all derivatives is provided in the Experimental Section, whereas all NMR spectra (^1^H, ^13^C) and HRMS pictures are shown in Supporting Information part 1, Section S.1. In addition, some recrystallized and crude NMRs are provided in order to show the level of purity of the synthesized compounds via Method C.

**Figure 3:**
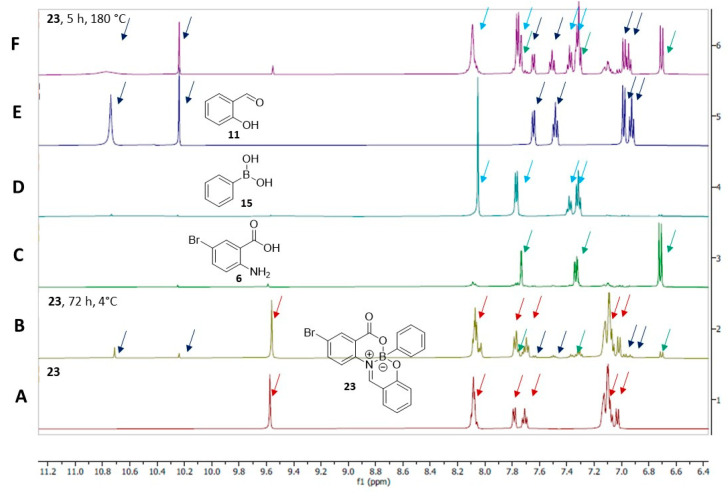
NMR spectra of compound **23** at time 0 h (picture (**A**)—red arrows) and 72 h (picture (**B**)—the red arrows indicate the remaining of compound **23** whereas dark blue, light blue and green arrows the new peaks that appeared) and after heating at 180 °C (picture (**F**)—no red arrows shown due to the almost entire decomposition of **23** to its components). Comparison of pictures (**A**,**B**,**F**) with the components of **23**: AA **6** (picture (**C**)—green arrows at pictures (**B**,**C**,**F**)), SA **11** (picture (**E**)—dark blue arrows at pictures (**B**,**E**,**F**)) and PBA **15** (picture (**D**)—light blue arrows at pictures (**B**,**D**,**F**)).

The stability test of the compounds towards hydrolysis was qualitative. Freshly prepared solutions of **23** and **27** were heated in DMSO-*d*_6_ at 180 °C, and NMR spectra were recorded at 2 and 5 h. Derivative **23** decomposed completely to its components after heating for 5 h (Figure 3F: dark blue arrows—SA **11**, Figure 3E; light blue arrows—PBA **15**, Figure 3D; green arrows—5-Br-AA **6**, Figure 3C), whereas the sample, after standing for 72 h in the fridge, showed considerable stability (Figure 3B: time 72 h; red arrows—compound **23**, Figure 3A: time 0 h).

**Figure 4:**
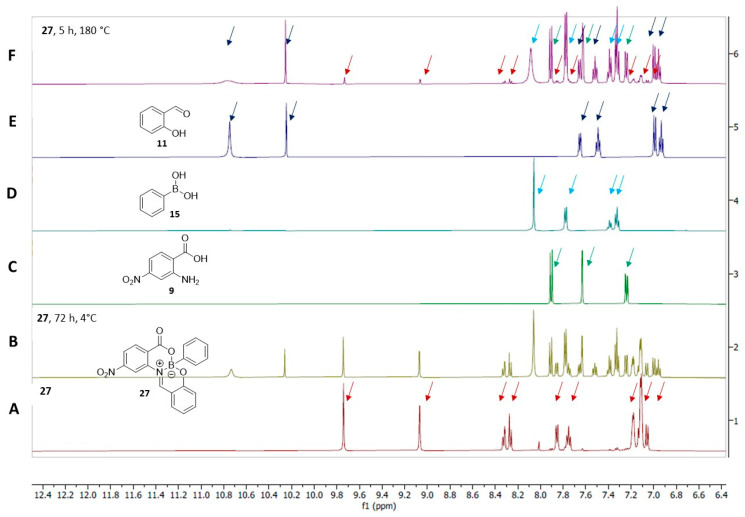
NMR spectra of compound **27** at time 0 h (picture (**A**)—red arrows) and 72 h (picture (**B**) and after heating at 180 °C (picture (**F**)—red arrows indicate the remaining of **27** whereas dark blue, light blue and green arrows indicate the position of the new compounds that appear. Comparison of pictures (**A**,**B**,**F**) with the components of **27**: AA **9** (picture (**C**)—green arrows at pictures (**C**,**F**)), SA **11** (picture (**E**)—dark blue arrows at pictures (**E**,**F**) and PBA **15** (picture (**D**)—light blue arrows at pictures (**D**,**F**)).

On the contrary, the NO_2_ derivative **27** decomposed considerably by standing for 72 h, Figure 4. In addition, all samples have been recorded by ^1^H-NMR at 0, 12, 24, 48 and 72 h intervals from their dissolution in DMSO-*d*_6_. In the meantime, the samples were kept at 4 °C (Supporting Information part 1, Section S.2). Within the BASAN group, the most unstable compound was derivative **25**. Comparing the derivatives coming from the same AA, the most stable derivatives were BASAN followed by BASAN-Cl and then BASAN-Br (Supporting Information part 1, Section S.3). These experimental results explain the difficulty of forming assemblies of AA **8**. Finally, comparing BASAN and BACAN derivatives, the results were dependent on the AA used. The more stable derivatives were pairs **18**/**28** and **19**/**29** derived from AAs **1** and **4**, respectively, where BACANs seemed to be more stable, although barely noticeable. On the other hand, 5-Cl and 5-Br AAs **5** and **6** gave BACAN derivatives **31** and **32**, which decomposed by standing in the fridge much quicker than their BASAN relatives **22** and **23**, respectively (Supporting Information part 1, Section S.4).

### 2.2. CT DNA Binding Studies of Boron Heterocycles ***18***–***24*** and ***26***–***48***

The interaction of freshly prepared solutions of compounds **18**–**24** and **26**–**48** in DMSO with calf thymus DNA (CT DNA) was investigated in vitro using UV–vis spectroscopy and viscosity measurements and via their ability to displace ethidium bromide (EB) from the EB-DNA adduct. The latter displacement can be monitored using fluorescence emission spectroscopy due to the intense fluorescence emission band of EB intercalated within DNA.

#### 2.2.1. UV–Vis Experiments

**Figure 5:**
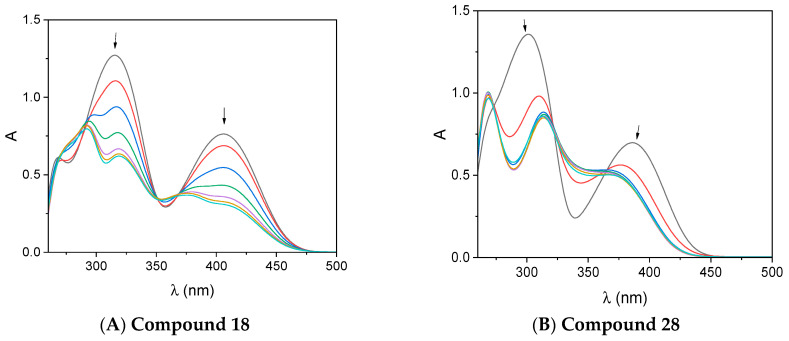

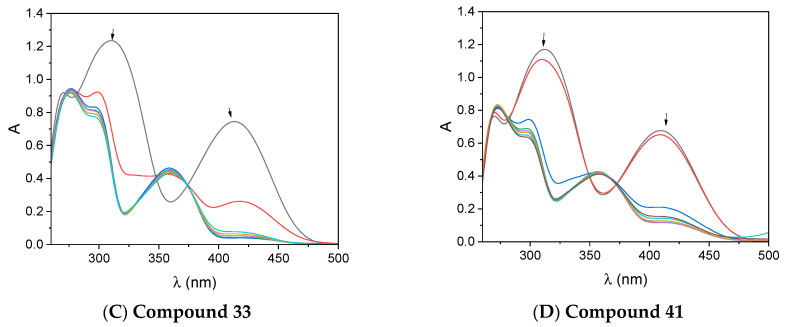
(**A**–**D**): UV–vis spectra of compounds **18**, **28**, **33** and **41**, respectively. Concentration: 10^−4^ M in DMSO in the presence of increasing amounts of CT DNA. The arrows show the changes upon increasing the amount of CT DNA.

UV–vis spectroscopy allows for the monitoring of structural changes induced by the interaction of CT DNA with the examined compounds (UV–vis spectra of compounds **18**–**24** and **26**–**48**, Supporting Information part 2, Section S.1) and the determination of the corresponding DNA-binding constants (K_b_) (Supporting Information part 2, Section S.2.1, Equation (S1)). In the UV–vis spectra of the compounds with increasing amounts of CT DNA, two intense bands appeared: band I in the region 387–420 nm and band II in the region 295–335 nm, while for compounds **24**, **41**, **43** and **45**–**48**, an additional third band (band III) appeared in the range 265–278 nm (Supporting Information part 2, Sections S.1 and S.3 for UV–vis spectra without CT DNA and with increasing amounts of CT DNA, respectively). The addition of a CT DNA solution resulted in a significant decrease in the absorbance of bands I and II, which, in some cases, led to disappearance of the band (Figure 5 and Supporting Information part 2, Section S.2.2.1). The effect of CT DNA on band III was a slighter increase in the absorbance (Table 1). In most cases, the bands exhibited a slight red-/blue-shift (Table 1). All these changes observed in the UV–vis spectra of the compounds in the presence of CT DNA offer evidence of the existence of their intense interaction with CT DNA, although the mode of interaction cannot be safely predicted [40].

Based on the Wolfe–Shimer equation (Supporting Information part 2, Section S.2.1, Equation (S1)) [41] and the plots [DNA]/(ε_A_-ε_f_) vs. [DNA] (Supporting Information part 2, Section S.2.2.2), the K_b_ values of compounds **18**–**24** and **26**–**48** were calculated (Table 1) and were found to range from 2.83 (± 0.12) × 10^4^ to 8.41 (± 0.13) × 10^6^ M^–1^. With the exception of compound **24**, all compounds exhibited a K_b_ value equal or higher than the classical intercalator EB (K_b(EB)_ = 1.23 (± 0.07) × 10^5^ M^–1^ [42]).

Comparing the K_b_ values of compounds **18**–**24** and **26**–**27** where only the substitution on AA has been changed, one may realize that the 5-Me, 5-OH, 4-Cl and 4-NO_2_ (compounds **19**, **20**, **26** and **27**, respectively; numbering on substituents is based on the AAs **2**, **3**, **9 10**) derivatives exhibited higher values than the non-substituted one (**18**) and all 5-halogenated compounds (compounds **21**–**24**). In the case of the iodo derivative **24**, the binding dropped considerably. The same change for K_b_ values was observed when SA **11** was changed to *o*-hydroxyacetophenone **14** (BAHCAN complexes **28**–**32**). Thus, all three halogenated derivatives (F-, Cl- and Br-, i.e., **30**, **31** and **32**, respectively) exhibited lower binding affinities of approximately ten times. Interestingly, in both the BACAN and BASAN groups, Me derivatives **19** and **29** exhibited the highest K_b_ values (1.63 (± 0.06) × 10^6^ M^−1^ and 8.41 (± 0.13) × 10^6^ M^−1^, respectively) in comparison with the rest of the compounds of their own group. The OH derivative **20** had the highest K_b_ value within groups **18**–**24** and **26**–**27**; however, such a derivative could not be synthesized for the other three groups of boronates, so we did not have the opportunity to discuss these results.

Nevertheless, regarding BASAN-Cl and BASAN-Br derivatives, all compounds were almost equally active, except compounds **37** and **45**, which were derived from the 5-Br-AA. Slightly lower was the K_b_ value of compound **40**, being almost equal to **37** and **45**. In the series of compounds **41**–**48,** the Me derivative **42**, again, had the highest binding affinity, whereas this was not the case for the BASAN-Cl, where the fluoro derivative exhibited the best binding within the series.

#### 2.2.2. Viscosity Experiments

The interaction mode with linear DNA may not be safely interpreted from UV–vis spectroscopy titrations, necessitating the performance of other experiments such as DNA viscosity measurements. The viscosity of linear DNA is a parameter that indicates changes in the DNA’s structure upon the addition of a studied compound (Supporting Information part 2, Section S.2.1.2) and provides information about the mode of interaction due to its sensitivity to the relative DNA length changes (L/L_0_) [43]. To be more specific, when a compound intercalates to DNA, the distance between the DNA base pairs increases in order to facilitate the insertion of the hosted compound into the intercalation site. This phenomenon leads to an increase in the DNA viscosity, the value of which is often proportional to the strength of the interaction [44]. The method is very sensitive and may distinguish non-classical intercalation (i.e., electrostatic interaction or groove binding), where the relative DNA length suffers a rather slight shortening, and accordingly, a slight decrease in the DNA viscosity may be induced [44].

**Figure 6:**
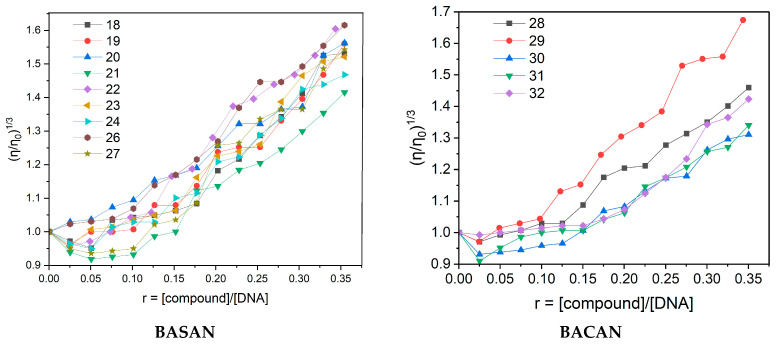

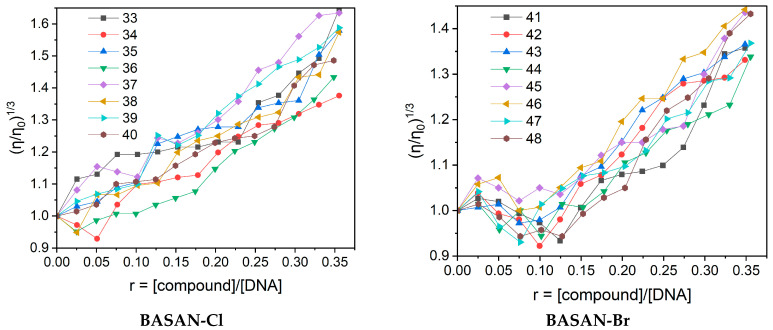
Relative viscosity (η/η_0_)^1/3^ of CT DNA (0.1 mM) in buffer solution (150 mM NaCl and 15 mM trisodium citrate at pH 7.0) in the presence of increasing amounts of compounds **18**–**24** and **26**–**48** (*r* = [compound]/[DNA] = 0–0.36).

Within this context, the viscosity of a CT DNA solution (0.1 mM) was monitored upon the addition of increasing amounts of compounds **18**–**24** and **26**–**48** (up to the value of *r* = 0.36, Figure 6).

Initially and up to an *r* value of ~0.1, the viscosity of the CT DNA solution remains practically stable for derivatives **21**, **22**, **24** and **27**, suggesting an external interaction with the compounds (obviously groove-binding). For *r* values above 0.1, the observed increase in the DNA viscosity could be attributed to an intercalative interaction [43,45]. It is, however, obvious that the derivatives that exhibited the higher K_b_ values (**19**, **20** and **26**) show an increase in the DNA compound length from the very beginning, indicating an intercalating mode of action. On the contrary, **21**, having the lowest K_b_ value, was reluctant to intercalate (Table 1: BASAN compounds **18**–**24** and **26**, Figure 6 BASAN).

Moving to the second BACAN group, **28**–**32**, in Figure 6 BACAN, it is obvious that Me derivative **29** followed by non-substituted compound **28** exhibited the best K_b_ values within their group, showing a length increase from the beginning of the addition of the compound in the DNA solution. Derivative **29** shows the more inclining graphical representation, and this is in correlation with its much higher K_b_ value of 8.41 (± 0.13) × 10^6^ M^−1^. The halogenated compounds **30**–**32** induce a lower DNA length increase (Table 1: BACAN compounds **28**–**32**, Figure 6 BACAN).

For the third and fourth groups (BASAN-Cl **33**–**40** and BASAN-Br **41**–**48**) with the Cl- and Br-substituted SA and ABA, respectively, we observe differences among them and with the other two groups. For example, the third group shows an intercalative profile from the beginning, contrary to groups 1 and 2 (Figure 6, BASAN-Cl and BASAN, BACAN). On the contrary, all compounds of group 4 show a significant DNA length decrease, and the final DNA lengths, as depicted with the viscosity values (η/η_0_)^1/3^, do not reach the same values (Figure 6, BASAN-Cl and BASAN-Br, respectively).

#### 2.2.3. EB-Displacement Fluorometric Experiments

EB is a fluorescent dye that intercalates to DNA and forms an adduct with an intense emission band at 592–593 nm when excited at 540 nm [46]. If a compound is able to intercalate to DNA equally or stronger than EB, it might be added into the EB-DNA solution, thus changing the EB-DNA emission band. This phenomenon, i.e., the competition of the compound with EB for the DNA intercalation site, can be observed and monitored [46]. Usually, the fluorescence emission spectra of 1 h pretreated EB-DNA complex ([EB] = 20 µM, [DNA] = 26 µM) is recorded in the presence of increasing amounts (up to *r* (= [compound]/[DNA]) value of 0.44) of the examined compounds (Supporting Information part 2, Section S.2.3.1). The Stern–Volmer (K_SV_) constants of the compounds were calculated using the Stern–Volmer equation (Supporting Information part 2, Section S.2.1.3, Equation (S2)) and the corresponding Stern–Volmer plots (Supporting Information part 2, Section S.2.3.2). In addition, the EB-DNA quenching constants (K_q_) of the compounds were calculated using Equation (S3) (Supporting Information part 2, Section S.2.1.3, Equation (S3)) considering τ_o_ = 23 ns as the fluorescence lifetime value of the EB-DNA system [47]. In addition, the fluorescence spectra of all compounds are given in Supporting Information part 2, Section S.3.

**Figure 7:**
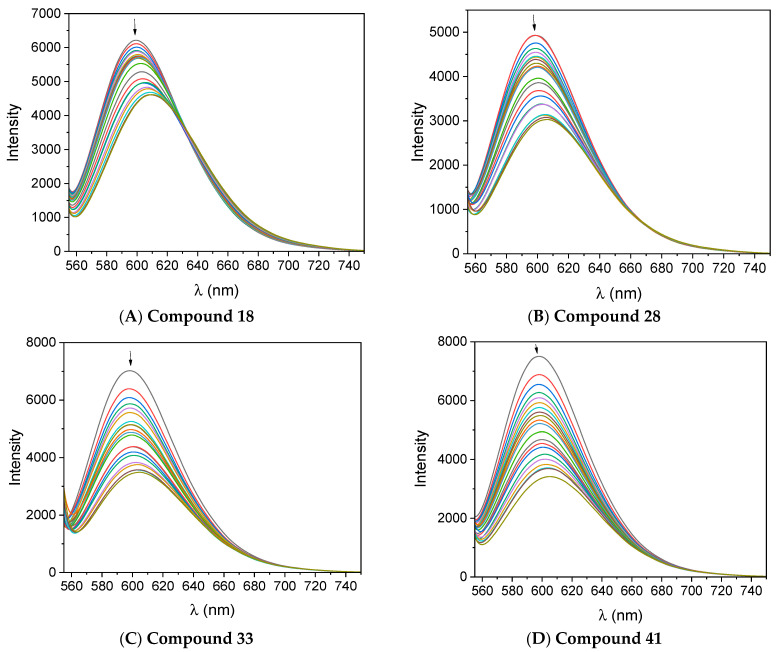
(**A**–**D**): Fluorescence emission spectra (λ_exc_ = 540 nm) for EB-DNA conjugate ([EB] = 20 μM, [DNA] = 26 μM) in buffer solution (150 mM NaCl and 15 mM trisodium citrate at pH = 7.0) in the presence of increasing amounts of compounds **18**, **28**, **33** and **41** (*r* = [compound]/[DNA] = 0–0.44). The arrows show the changes in the intensity upon adding increasing amounts of the compounds.

**Figure 8:**
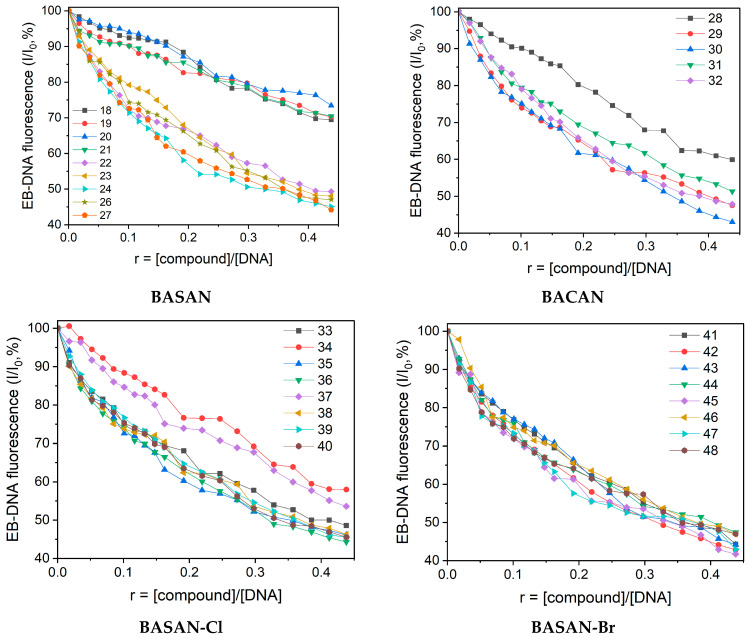
Plots of EB–DNA relative fluorescence emission intensities (I/I_0_, %) at λ_emission_ = 592 nm vs. *r* (*r* = [compound]/[DNA]) in the presence of compounds **18**–**24** and **26**–**48**.

Figure 7 depicts the fluorescence emission spectra of the EB-DNA adduct and the changes caused by the sequential addition of incremental amounts of compounds **18**, **28**, **33** and **41**, which all derive from 5-Me AA **2**. Even at a glance, one may observe that **41** is a better intercalator than **33**. Derivatives **28** and **18** decrease in the same order (Figure 8, Table 2, (BACAN and BASAN, respectively)). Regarding the K_sv_ constants, the K_q_ constants, and the percentage of EB-DNA fluorescence quenching (ΔI/I_o_ %), which were all calculated from the fluorescence spectrometry experiments (Table 2), we may conclude K_SV,**41**_ (4.60 × 10^4^ M^−1^) > K_SV,**33**_ (4.11 × 10^4^ M^−1^) > K_SV,**28**_ (2.82 × 10^4^ M^−1^) > K_SV,**18**_ (1.84 × 10^4^ M^−1^) and, subsequently, K_q,**41**_ (2.00 × 10^12^ M^−1^ s^−1^) > K_q,**33**_ (1.79 × 10^12^ M^−1^ s^−1^) > K_q,**28**_ (1.23 × 10^12^ M^−1^ s^−1^) > K_q,**18**_ (0.80 × 10^12^ M^−1^ s^−1^).

Among all compounds, a significant decrease in the fluorescence emission band of EB-DNA at 592 nm (up to 58.3%) is observed for compound **47** (Table 2), which means that this compound is the most capable of displacing EB in the EB–DNA adduct. The K_SV_ constants of the compounds are relatively high (Table 2), and for each group of compounds, derivatives **24**, **30**, **36** and **47** exhibit the highest values (4.88 × 10^4^, 5.13 × 10^4^, 4.96 × 10^4^ and 5.36 × 10^4^ M^–1^, respectively), with **47** giving the highest among all compounds, suggesting tight binding to DNA. In addition, the quenching constants K_q_ of the compounds (Table 2) are higher than 10^10^ M^–1^ s^–1^, proposing the existence of a static quenching mechanism induced by the compounds [46] and subsequently suggesting the interaction of the compounds with the fluorophore and the displacement of EB. Thus, an intercalative mode of interaction of the complexes with CT DNA can be indirectly proposed [48].

## 3. Materials and Methods

### 3.1. General

All commercially available reagent-grade chemicals and solvents were used without further purification. Trisodium citrate, NaCl, CT DNA and ethidium bromide (EB) were purchased from Sigma-Aldrich Co and all other solvents from Chemlab. CT DNA stock solution was prepared via the dilution of CT DNA with buffer (containing 150 mM NaCl and 15 mM trisodium citrate at pH 7.0) followed by exhaustive stirring at 4 °C for 3 days and storage at 4 °C for no longer than a week. The stock solution of CT DNA gave a ratio of UV absorbance at 260 and 280 nm (A_260_/A_280_) of ~1.90, indicating that the DNA was sufficiently free of protein contamination [49]. The DNA concentration per nucleotide was determined by the absorbance of the band at 260 nm after a 1:20 dilution using ε = 6600 M^−1^ cm^−1^ [50]. UV–vis spectra were recorded on a Hitachi U–2001 dual-beam spectrophotometer (Hitachi, Tokyo, Japan). Fluorescence spectra were recorded in solution on a Hitachi F-7000 fluorescence spectrophotometer (Hitachi, Tokyo, Japan). FT–infrared (FT–IR) spectra were recorded in the range (400–4000 cm^−1^) on a Thermo Scientific Nicolet iS20 FT–IR ATR spectrometer (Thermo Fisher Scientific). Viscosity experiments were carried out using an ALPHA L Fungilab rotational viscometer (Fungilab, Barcelona, Spain) equipped with an 18 mL LCP spindle and the measurements were performed at 100 rpm. NMR spectra were recorded on an Agilent 500/54 (500 MHz and 125 MHz for ^1^H and ^13^C, respectively) spectrometer (Agilent Technologies, Santa Clara, CA, USA) using CDCl_3_ and/or DMSO-*d*_6_ as solvent. *J* values are reported in Hz. The high-resolution mass spectra measured with a Q−TOF (Time-of-Flight Mass Spectrometry) Maxis Impact (Bruker Daltonics, Bremen, Germany) with an ESI source and U-HPLC Thermo Dionex Ultimate 3000 RSLC (ThermoFisher Scientific, Dreieich, Germany) pump and autosampler. N_2_ was used as the collision gas, and electrospray ionization (ESI) was used for the MS experiments. Data acquisition was carried out with data analysis software from Bruker Daltonics (Bremen, Germany) (version 4.1). MW experiments were performed on a scientific focused microwave reactor (Biotage Initiator 2.0, Uppsala, Sweden). All reactions were monitored on commercially available pre-coated TLC plates (layer thickness of 0.25 mm; Kieselgel 60 F_254_) (Merck, Darmstadt, Germany). The calculation of the yields was based on the amount of the directly crystallized product or after purification (when needed) and recrystallization. The melting points were measured with GallenKamp MFB-595 melting point apparatuses (GallenKamp, MFB-595 (USA)) and are uncorrected.

### 3.2. General Method for the One-Pot Synthesis of Compounds **18**–**32** in Toluene (BASAN and BACAN): Method A

Anthranilic acids **1**–**10** (1 mmol), salicylaldehyde **11** or o-hydroxyacetophenone **14** (1 mmol) and phenyl boronic acid **15** (1.5 mmol) were mixed in toluene (1.5 mL) in the presence of molecular sieves (2 g), and the mixture was subjected to microwave irradiation for 1 h (in the case of salicylaldehyde **11**) or for 1.5 h (in case of o-hydroxyacetophenone **14**) at 180 °C. After cooling the reaction mixture at room temperature, a precipitate was formed, which was isolated via simple filtration. The obtained products **18**–**32** were washed with ethyl acetate (5 × 1 mL) and petroleum ether (2 × 1 mL) and dried. The filtrates contained the remaining Schiff base, phenyl boronic acid and a small amount of the product (TLC test).

### 3.3. General Method for the One-Pot Synthesis of Compounds **21** and **23** and the Tandem Synthesis of Compound **24** in EtOH (BASAN): Method B

Anthranilic acids **4** or **6** (1 mmol), salicylaldehyde **11** (1 mmol) and phenyl boronic acid **15** (1.5 mmol) were mixed in ethanol (1.5 mL), and the mixture was subjected to microwave irradiation for 1 h at 140 °C**.** After cooling the reaction mixture at room temperature, a precipitate was formed, which was isolated via simple filtration. The obtained products **21** or **23** were washed with ethyl acetate (5 × 1 mL) and petroleum ether (2 × 1 mL) and dried. The filtrates contained the remaining Schiff base, salicylaldehyde and phenyl boronic acid (TLC test). Tandem reaction: Anthranilic acid **7** (1 mmol) and salicylaldehyde **11** (1 mmol) were mixed in ethanol (1.5 mL), and the mixture was subjected to microwave irradiation for 1 h at 140 °C. The reaction was monitored via TLC (formation of the corresponding Schiff base). Phenyl boronic acid **15** (1.5 mmol) was added, and the mixture was subjected to microwave irradiation for 1 h at 140 °C. After cooling the reaction mixture at room temperature, a solid was formed, which was isolated as above.

### 3.4. General Method for the One-Pot Synthesis of Compounds ***18***–***23***, ***26*** and ***33***–***48*** BASAN, BASAN-Cl and BASAN-Br: Method C

Anthranilic acids **1**–**6** and **9** (1 mmol), salicylaldehydes **11**–**13** (1 mmol) and phenyl boronic acids **15**–**17** (1.5 mmol) were individually added into a solvent system, H_2_O:EtOH 3:1 (2 mL), and the mixture was subjected to microwave irradiation for 1 h at 150 °C. After cooling the reaction mixture at room temperature, a precipitate was formed, which was isolated via simple filtration. The obtained products **18**–**23**, **26** and **33**–**48** were subsequently washed with ethyl acetate (5 × 1 mL) and petroleum ether (2 × 1 mL) and dried. The filtrate contained only the remaining phenyl boronic acid.

### 3.5. Data of Compounds (Pictures of ^1^H and ^13^C-NMR and HRMS: Supporting Information Part 1, Section S.1)

#### 3.5.1. 7-Phenyl-5H,7H-7λ^4^,14λ^4^-benzo[d]benzo [5,6][1,3,2]oxazaborinino [2,3b][1,3,2]oxazaborinin-5-one (**18**)

Methods A and C: light-yellow solid; mp: 256–257 °C; yield: 89% and 98%, respectively; IR (neat) cm^−1^: 3069, 3006, 2981, 1692 (C=O), 1615 (C=N), 1595, 1550 (B-N), 1471, 1451, 1391, 1330, 1300 (B-O), 1191, 1178, 1153, 1017, 999, 947, 882, 831, 764, 744, 699, 689; ^1^H−NMR (DMSO-*d*_6_, 500 MHz) δ 9.57 (s, 1H, H-5), 8.11 (d, *J* = 8.2 Hz, 1H, H-1), 8.02 (dd, *J* = 7.8, 1.5 Hz, 1H, H-4), 7.86–7.76 (m, 2H, H-6, H-2), 7.69 (dt, *J* = 8.7, 1.8 Hz, 1H, H-8), 7.56 (t, *J* = 7.6 Hz, 1H, H-3), 7.16–7.11 (m, 2H, B-Ar), 7.10–7.04 (m, 4H, B-Ar and H-7 obscured), 7.02 (d, *J* = 8.4 Hz, 1H, H-9) ppm; ^13^C−NMR (DMSO-*d*_6_, 125 MHz) δ 162.0, 161.3, 158.6, 140.0, 139.8, 134.6, 134.2, 130.6, 130.1, 129.5, 127.6, 127.5, 123.7, 120.2, 119.6, 118.7, 116.4 ppm; HRMS(ESI) *m*/*z* [M+Na]^+^: C_20_H_14_BNNaO_3_^+^; calc.: 350.0959; found: 350.0954.

#### 3.5.2. 3-Methyl-7-phenyl-5H,7H-7λ^4^,14λ^4^-benzo[d]benzo [5,6][1,3,2]oxazaborinino [2,3-b] [1,3,2]oxazaborinin-5-one (**19**)

Methods A and C: dark-yellow solid; mp: 270–271 °C; yield: 78% and 99%; IR (neat) cm^−1^: 3038, 2980, 1677 (C=O), 1620 (C=N), 1548 (B-N), 1475, 1454, 1396, 1329, 1312 (B-O), 1225, 1181, 1152, 1015, 996, 948, 844, 829, 762, 747, 699, 652, 550, 516; ^1^H−NMR (DMSO-*d*_6_, 500 MHz) δ 9.53 (s, 1H, H-5), 8.01 (d, *J* = 8.3 Hz, 1H, H-4), 7.83 (s, 1H, H-1), 7.77 (dd, *J* = 7.9. 1.8 Hz, 1H, H-6), 7.67 (dt, *J* = 8.7, 1.7 Hz, 1H, H-8), 7.63 (dd, *J* = 8.5, 2.1 Hz, 1H, H-3), 7.18–7.10 (m, 2H, B-Ar), 7.09–7.03 (m, 4H, B-Ar and H-7) 7.01 (d, *J* = 8.4 Hz, 1H, H-9), 2.37 (s, 3H, CH_3_) ppm; ^13^C−NMR (DMSO-*d*_6_, 125 MHz) δ 161.4, 161.0, 158.4, 139.8, 137.4, 135.2, 134.0, 130.7, 130.1, 127.5, 127.5, 123.4, 120.2, 119.4, 118.7, 116.5, 20.6 ppm; HRMS(ESI) *m*/*z* [M+Na]^+^: C_21_H_16_BNNaO_3_^+^; calc.: 364.1115; found: 364.1120.

#### 3.5.3. 3-Hydroxy-7-phenyl-5H,7H-7λ^4^,14λ^4^-benzo[d]benzo [5,6][1,3,2]oxazaborinino [2,3-b][1,3,2]oxazaborinin-5-one (**20**)

Methods A and C: black solid; mp: 285–286 °C yield: 51% and 98%; IR (neat) cm^−1^: 3304, 3035, 3003, 2980, 1667 (C=O), 1615 (C=N), 1548 (B-N), 1471, 1455, 1432, 1392, 1357, 1310 (B-O), 1251, 1190, 1150, 1087, 1007, 942, 886, 847, 826, 768, 741, 695, 646, 561, 516; ^1^H−NMR (DMSO-*d*_6_, 500 MHz) δ 10.45 (s, 1H, OH), 9.41 (s, 1H, H-5), 7.96 (d, *J* = 8.9 Hz, 1H, H-4), 7.73 (dd, *J* = 7.8, 1.8 Hz, 1H, H-6), 7.63 (dt, *J* = 8.6, 1.8 Hz, 1H, H-8), 7.37 (d, *J* = 2.8 Hz, 1H, H-1), 7.16 (dd, *J* = 8.9, 2.8 Hz, 1H, H-3), 7.14–7.06 (m, 5H, B-Ar), 7.04 (t, *J* = 7.5 Hz, 1H, H-7), 7.00 (d, *J* = 8.4 Hz, 1H, H-9) ppm; ^13^C−NMR (DMSO-*d*_6_, 125 MHz) δ 161.3, 159.1, 158.5, 158.0, 139.1, 133.6, 131.5, 130.1, 127.5, 127.4, 125.0, 121.7, 121.2, 120.1, 118.5, 116.7, 115.9 ppm; HRMS(ESI) *m*/*z* [M+Na]^+^: C_20_H_14_BNNaO_4_^+^; calc.: 366.0908; found: 366.0917.

#### 3.5.4. 3-Fluoro-7-phenyl-5H,7H-7λ^4^,14λ^4^-benzo[d]benzo [5,6][1,3,2]oxazaborinino [2,3-b][1,3,2]oxazaborinin-5-one (**21**)

Methods A, B and C: dark-yellow solid; mp: 277–278 °C; yield: 92%, 99% and 87%; IR (neat) cm^−1^: 3065, 1687 (C=O), 1616 (C=N), 1552 (B-N), 1474, 1441, 1384, 1307 (B-O), 1292, 1279, 1180, 1154, 1143, 1080, 1013, 1003, 952, 897, 845, 795, 764, 743, 702, 653, 564, 554; ^1^H−NMR (DMSO-*d*_6_, 500 MHz) δ 9.54 (s, 1H, H-5), 8.20 (dd, *J* = 9.9, 4.4 Hz, 1H, H-3), 7.81–7.72 (m, 3H, H-1, H-4, H-6), 7.69 (dt, *J* = 8.8, 1.8 Hz, 1H, H-8), 7.18–7.11 (m, 2H, B-Ar), 7.11–7.05 (m, 4H, B-Ar and H-7 obscured) 7.03 (d, *J* = 8.4 Hz, 1H, H-9) ppm; ^13^C−NMR (DMSO-*d*_6_, 75 MHz) δ 161.7 (d, ^1^J_C-F_ = 247.4 Hz), 162.2, 160.3 (d, ^4^J_C-F_ = 2.2 Hz), 158.5, 140.1, 136.4 (d, ^4^J_C-F_ = 2.8 Hz), 134.2, 130.1, 127.6, 127.5, 125.9, 125.8 (d, ^3^J_C-F_ = 7.8 Hz), 122.4 (d, ^3^J_C-F_ = 8.5 Hz), 122.0 (d, ^2^J_C-F_ = 23.8 Hz), 120.3, 118.7, 116.6 (d, ^2^J_C-F_ = 24.2 Hz), 116.3 ppm; HRMS(ESI) *m*/*z* [M+Na]^+^: C_20_H_13_BFNNaO_3_^+^; calc.: 368.0865; found: 368.0866.

#### 3.5.5. 3-Chloro-7-phenyl-5H,7H-7λ^4^,14λ^4^-benzo[d]benzo [5,6][1,3,2]oxazaborinino [2,3-b][1,3,2]oxazaborinin-5-one (**22**)

Methods A and C: light-yellow solid; mp: 298–299 °C; yield: 72% and 98%; IR (neat) cm^−1^: 3077, 1683 (C=O), 1615 (C=N), 1549 (B-N), 1471, 1456, 1426, 1385, 1300 (B-O), 1276, 1179, 1152, 1014, 999, 949, 843, 773, 742, 699, 651, 546; ^1^H−NMR (DMSO-*d*_6_, 500 MHz) δ 9.57 (s, 1H, H-5), 8.16 (d, *J* = 8.5 Hz, 1H, H-3), 8.00–7.90 (m, 2H, H-1, H-4), 7.78 (dd, *J* = 7.8, 1.8 Hz, 1H, H-6), 7.71 (dt, *J* = 8.7, 1.8 Hz, 1H, H-8), 7.16–7.06 (m, 6H, B-Ar and H-7 obscured), 7.03 (d, *J* = 8.4 Hz, 1H, H-9) ppm; ^13^C−NMR (DMSO-*d*_6_, 125 MHz) δ 162.6, 160.2, 158.6, 140.4, 138.7, 134.5, 134.3, 133.9, 130.1, 129.8, 127.7, 127.6, 125.2, 121.9, 120.4, 118.8, 116.3 ppm; HRMS(ESI) *m*/*z* [M+Na]^+^: C_20_H_13_BClNNaO_3_^+^; calc.: 384.0569; found: 384.0570.

#### 3.5.6. 3-Bromo-7-phenyl-5H,7H-7λ^4^,14λ^4^-benzo[d]benzo [5,6][1,3,2]oxazaborinino [2,3-b][1,3,2]oxazaborinin-5-one (**23**)

Methods A, B and C: light-yellow solid; mp: 306–307 °C; yield: 94%, 91% and 85%; IR (neat) cm^−1^: 3068, 1683 (C=O), 1615 (C=N), 1548 (B-N), 1470, 1456, 1387, 1300 (B-O), 1277, 1182, 1150, 997, 950, 906, 840, 757, 727, 697, 652, 557, 546; ^1^H−NMR (DMSO-*d*_6_, 500 MHz) δ 9.57 (s, 1H, H-5), 8.13–8.05 (m, 3H, H-1, H-3, H-4), 7.78 (dd, *J* = 7.8, 1.7 Hz, 1H, H-6), 7.71 (t, *J* = 7.6 Hz, 1H, H-8), 7.16–7.05 (m, 6H, B-Ar and H-7 obscured), 7.03 (d, *J* = 8.4 Hz, 1H, H-9) ppm; ^13^C−NMR (DMSO-*d*_6_, 125 MHz) δ 162.6, 160.1, 158.6, 140.4, 139.1, 137.4, 134.3, 132.7, 130.1, 127.7, 127.5, 125.3, 122.3, 122.1, 120.4, 118.7, 116.3 ppm; HRMS(ESI) *m*/*z* [M+Na]^+^: C_20_H_13_BBrNNaO_3_^+^; calc.: 428.0064; found: 428.0062.

#### 3.5.7. 3-Iodo-7-phenyl-5H,7H-7λ^4^,14λ^4^-benzo[d]benzo [5,6][1,3,2]oxazaborinino [2,3-b][1,3,2]oxazaborinin-5-one (**24**)

Method B: light-yellow solid; mp: 313–314 °C; yield: 56%; IR (neat) cm^−1^: 3069, 1683, (C=O), 1615 (C=N), 1549 (B-N), 1469, 1456, 1386, 1300 (B-O), 1278, 1181, 1152, 1001, 947, 837, 756, 743, 696, 651, 556, 545; ^1^H−NMR (DMSO-*d*_6_, 500 MHz) δ 9.57 (s, 1H, H-5), 8.26 (d, *J* = 2.0 Hz, 1H, H-1), 8.20 (dd, *J* = 8.5, 2.1 Hz, 1H, H-3), 7.92 (d, *J* = 8.6 Hz, 1H, H-4), 7.78 (dd, *J* = 7.8, 1.8 Hz, 1H, H-6), 7.70 (dt, *J* = 8.7, 1.8 Hz, 1H, H-8), 7.17–7.05 (m, 6H, B-Ar and H-7 obscured), 7.02 (d, *J* = 8.4 Hz, 1H, H-9) ppm; ^13^C−NMR (DMSO-*d*_6_, 75 MHz) δ 162.3, 160.1, 158.6, 143.0, 140.3, 139.5, 138.7, 134.3, 130.1, 127.7, 127.5, 125.1, 121.7, 120.4, 118.7, 116.3, 95.5 ppm; HRMS(ESI) *m*/*z* [M+Na]^+^: C_20_H_13_BINNaO_3_^+^; calc.: 475.9925; found: 475.9934.

#### 3.5.8. 3-Nitro-7-phenyl-5H,7H-7λ^4^,14λ^4^-benzo[d]benzo [5,6][1,3,2]oxazaborinino [2,3-b][1,3,2]oxazaborinin-5-one (**25**)

Method A: orange solid; mp: -; IR (neat-crude) cm^−1^: 2981, 1694 (C=O), 1620 (C=N), 1587, 1545 (B-N), 1470, 1452, 1387, 1347 (B-O), 1326, 1286, 1211, 1152, 1123, 1011, 850, 760, 750, 690, 540, 459; ^1^H−NMR (DMSO-*d*_6_, 500MHz) ppm: the crude not-purified product shows an NMR picture in parallel to the rest of the compounds in the series (Supporting Information, part 1, Section S.1.); ^13^C−NMR (DMSO-*d*_6_, 125 MHz) δ ppm: the compound started decomposing, and this spectrum, even not purified, has not been taken. Derivative **25** was kept in the list only for the discussion concerning the stability and the behavior of anthranilic acid **8**. It was unstable, and there was no way to purify with recrystallization.

#### 3.5.9. 2-Chloro-7-phenyl-5H,7H-7λ^4^,14λ^4^-benzo[d]benzo [5,6][1,3,2]oxazaborinino [2,3-b][1,3,2]oxazaborinin-5-one (**26**)

Methods A and C: light-yellow solid; mp: 334–335 °C; yield: 65% and 95%; IR (neat) cm^−1^: 3087, 3008, 2958, 1690 (C=O), 1615 (C=N), 1591, 1548 (B-N), 1471, 1456, 1389, 1346, 1333, 1305 (B-O), 1244, 1209, 1179, 1157, 1086, 1014, 998, 966, 880, 858, 835, 761, 745, 698, 687, 564, 538; ^1^H−NMR (DMSO-*d*_6_, 500 MHz) δ 9.61 (s, 1H, H-5), 8.32 (d, *J* = 2.0 Hz, 1H, H-4), 8.01 (d, *J* = 8.4 Hz, 1H, H-1), 7.77 (dd, *J* = 7.8, 1.7 Hz, 1H, H-6), 7.72 (dt, *J* = 8.7, 1.8 Hz, 1H, H-8), 7.62 (dd, *J* = 8.4, 1.9 Hz, 1H, H-2), 7.17–7.06 (m, 6H, B-Ar and H-7 obscured), 7.04 (d, *J* = 8.4 Hz, H-9) ppm; ^13^C−NMR (DMSO-*d*_6_, 125 MHz) δ 163.2, 160.6, 158.8, 141.0, 141.0, 139.0, 134.4, 132.3, 130.1, 129.5, 127.7, 127.6, 122.5, 120.5, 120.0, 118.8, 116.2 ppm; HRMS (ESI) *m*/*z* [M+Na]^+^: C_20_H_13_BClNNaO_3_^+^; calc.: 384.0569; found: 384.0567.

#### 3.5.10. 2-Nitro-7-phenyl-5H,7H-7λ^4^,14λ^4^-benzo[d]benzo [5,6][1,3,2]oxazaborinino [2,3-b][1,3,2]oxazaborinin-5-one (**27**)

Method A: orange solid; mp: 317–318 °C; yield: 78%; IR (neat) cm^−1^: 3076, 1698 (C=O), 1619 (C=N), 1548 (B-N), 1531, 1471, 1456, 1393, 1349, 1330, 1304 (B-O), 1183, 1009, 977, 911, 865, 840, 822, 767, 747, 702; ^1^H−NMR (DMSO-*d*_6_, 500 MHz) δ 9.73 (s, 1H, H-5), 9.06 (d, *J* = 2.2Hz, 1H, H-4), 8.26 (d, *J* = 8.6 Hz, 1H, H-1), 7.85 (dd, *J* = 8.0, 1.7 Hz, 1H, H-6), 7.74 (dt, *J* = 8.4, 1.8 Hz, 1H, H-8), 7.20–7.13 (m, 2H, B-Ar), 7.15–7.07 (m, 4H, B-Ar and H-7 obscured), 7.05 (d, *J* = 8.4 Hz, 1H, H-9) ppm; ^13^C−NMR (DMSO-*d*_6_, 75 MHz) δ 164.33, 159.92, 158.86, 150.89, 140.87, 134.62, 132.35, 130.18, 128.36, 127.76, 127.58, 123.64, 120.56, 118.80, 116.13, 115.61 ppm; HRMS (ESI) *m*/*z* [M+Na]^+^: C_20_H_13_BN_2_NaO_5_^+^; calc.: 395.0810; found: 395.0808.

#### 3.5.11. 13-Methyl-7-phenyl-5H,7H-7λ^4^,14λ^4^-benzo[d]benzo [5,6][1,3,2]oxazaborinino [2,3-b] [1,3,2]oxazaborinin-5-one (**28**)

Method A: light-green solid; mp: 221–222 °C; yield: 59%; IR (neat) cm^−1^: 3734, 2981, 1695 (C=O), 1610 (C=N), 1589, 1548 (B-N), 1475, 1455, 1431, 1373, 1356, 1316 (B-O), 1272, 1189, 1135, 1013, 977, 936, 837, 767, 739, 703; ^1^H−NMR (DMSO-*d*_6_, 500 MHz) δ 7.97 (d, *J* = 8.2 Hz, 1H, H-4), 7.88 (d, *J* = 7.7 Hz, 1H, H-1), 7.71 (d, *J* = 8.0 Hz, 1H, H-6), 7.66–7.60 (m, 2H, H-2, H-3), 7.46 (t, *J* = 7.6 Hz, 1H, H-8), 7.14–7.07 (m, 2H, B-Ar), 7.06–6.97 (m, 4H, B-Ar and H-7 obscured), 6.96 (d, *J* = 8.4 Hz, 1H, H-9), 2.88 (s, 3H, CH_3_) ppm; ^13^C−NMR (DMSO-*d*_6_, 125 MHz) δ 173.8, 162.1, 156.6, 138.8, 138.4, 132.6, 131.0, 131.0, 130.0, 129.1, 127.2, 127.1, 127.0, 126.0, 120.0, 119.0, 117.3, 19.1 ppm; HRMS (ESI) *m*/*z* [M+Na]^+^: C_21_H_16_BNNaO_3_^+^; calc.: 364.1115; found: 364.1125.

#### 3.5.12. 3,13-Dimethyl-7-phenyl-5H,7H-7λ^4^,14λ^4^-benzo[d]benzo [5,6][1,3,2]oxazaborinino [2,3-b] [1,3,2]oxazaborinin-5-one (**29**)

Method A: orange solid; mp: 278–279 °C; yield: 70%; IR (neat) cm^−1^: 3075, 2982, 1698 (C=O), 1610 (C=N), 1593, 1549 (B-N), 1480, 1456, 1434, 1357, 1305 (B-O), 1277, 1185, 1145, 1041, 1018, 965, 929, 890, 835, 765, 727, 702; ^1^H−NMR (DMSO-*d*_6_, 500 MHz) δ 7.95 (d, *J* = 8.1 Hz, 1H, H-4), 7.70 (s, 1H, H-1), 7.66–7.56 (brs, 2H, H-6, H-8), 7.45 (d, *J* = 8.2 Hz, 1H, H-3), 7.15–7.06 (brs, 2H, B-Ar), 7.05–6.97 (brs, 4H, B-Ar and H-7 obscured), 6.95 (d, *J* = 8.3 Hz, 1H, H-9), 2.90 (s, 3H, CH_3_), 2.34 (s, 3H, CH_3_) ppm; ^13^C−NMR (DMSO-*d*_6_, 125 MHz) δ 173.2, 162.2, 156.5, 139.1, 138.2, 136.4, 133.1, 130.9, 130.5, 129.9, 127.2, 127.0, 126.8, 125.6, 120.0, 119.0, 117.4, 20.4886, 19.0419 ppm; HRMS (ESI) *m*/*z* [M+K]^+^: C_22_H_18_BKNO_3_^+^; calc.: 394.1011; found: 394.1015.

#### 3.5.13. 3-Fluoro-13-methyl-7-phenyl-5H,7H-7λ^4^,14λ^4^-benzo[d]benzo [5,6][1,3,2]oxazaborinino [2,3-b][1,3,2]oxazaborinin-5-one (**30**)

Method A: light-green solid; mp: 286–288 °C; yield: 67%; IR (neat) cm^−1^: 3733, 3070, 2981, 1704 (C=O), 1609 (C=N), 1591, 1548 (B-N), 1477, 1456, 1432, 1374, 1355, 1340, 1292 (B-O), 1191, 1133, 1083, 1039, 1018, 982, 937, 904, 862, 830, 773, 764, 750, 704, 654, 560, 507; ^1^H−NMR (DMSO-*d*_6_, 500 MHz) δ 7.98 (d, ^3^J_F-H_ = 8.1 Hz, 1H, H-1), 7.81 (dd, ^3^J_H-H_ = 8.9 Hz, ^4^J_F-H_ = 4.6 Hz, 1H, H-4), 7.67–7.60 (m, 2H, H-3, H-6), 7.54 (td, *J* = 8.4, 3.0 Hz, 1H, H-8), 7.15–7.07 (m, 2H, B-Ar), 7.06–6.98 (m, 4H, B-Ar and H-7 obscured), 6.96 (d, *J* = 8.3 Hz, 1H, H-9), 2.89 (s, 3H, CH_3_) ppm; ^13^C−NMR (DMSO-*d*_6_, 75 MHz) δ 174.2, 161.2 (d, ^1^J_C-F_ = 247.3 Hz), 161.0 (d, ^4^J_C-F_ = 2.3 Hz), 156.6, 138.5, 135.4 (d, ^4^J_C-F_ = 3.1 Hz), 131.0, 130.5, 129.4 (d, ^3^J_C-F_ = 7.8 Hz), 128.2 (d, ^3^J_C-F_ = 8.4 Hz), 127.3, 127.1, 119.8 (d, ^2^J_C-F_ = 23.1 Hz), 119.8, 119.0, 117.2, 116.0 (d, ^2^J_C-F_ = 24.1 Hz), 19.0 ppm; HRMS (ESI) *m*/*z* [M+Na]^+^: C_21_H_15_BFNNaO_3_^.+^; calc.: 382.1021; found: 382.1021.

##### 3.5.14. 3-Chloro-13-methyl-7-phenyl-5H,7H-7λ^4^,14λ^4^-benzo[d]benzo [5,6][1,3,2]oxazaborinino [2,3-b][1,3,2]oxazaborinin-5-one (**31**)

Method A: light-green solid; mp: 276–278 °C; yield: 71%; IR (KBr) cm^−1^: 3099, 3076, 1703 (C=O), 1610 (C=N), 1600, 1587, 1548 (B-N), 1478, 1468, 1455, 1415, 1374, 1358, 1298 (B-O), 1286, 1249, 1186, 1084, 1043, 1020, 960, 930, 863, 838, 791, 764, 701, 652; ^1^H−NMR (DMSO-*d*_6_, 500 MHz) δ 7.99 (d, *J* = 8.3 Hz, 1H, H-4), 7.83 (d, *J* = 2.4 Hz, 1H, H-1), 7.78 (d, *J* = 8.6 Hz, 1H, H-3), 7.74 (dd, *J* = 8.6, 2.4 Hz, 1H, H-6), 7.64 (t, *J* = 8.6 Hz, 1H, H-6), 7.15–7.09 (m, 2H, B-Ar), 7.08–7.02 (m, 4H, B-Ar and H-7 obscured) 6.97 (d, *J* = 8.3 Hz, 1H, H-9), 2.90 (s, 3H, CH_3_) ppm; ^13^C−NMR (DMSO-*d*_6_, 125 MHz) δ 174.6, 160.9, 156.6, 138.6, 137.8, 133.5, 132.5, 131.1, 130.6, 129.0, 128.9, 128.8, 127.7, 127.3, 127.1, 119.8, 119.0, 117.2, 19.1453 ppm; HRMS (ESI) *m*/*z* [M+Na]^+^: C_21_H_15_BClNNaO_3_^+^; calc.: 398.0726; found: 398.0737.

#### 3.5.15. 3-Bromo-13-methyl-7-phenyl-5H,7H-7λ^4^,14λ^4^-benzo[d]benzo [5,6][1,3,2]oxazaborinino [2,3-b][1,3,2]oxazaborinin-5-one (**32**)

Method A: dark-green solid; mp: 288–289 °C; yield: 62%; IR (neat) cm^−1^: 3733, 3097, 3074, 2981, 1705 (C=O), 1599 (C=N), 1548 (B-N), 1474, 1456, 1433, 1410, 1357, 1297 (B-O), 1284, 1248, 1184, 1083, 1020, 957, 928, 863, 835, 790, 764, 753, 701; ^1^H−NMR (DMSO-*d*_6_, 500 MHz) δ 7.99 (d, *J* = 8.1 Hz, 1H, H-4), 7.95 (s, 1H, H-1), 7.86 (d, *J* = 8.6 Hz, 1H, H-3), 7.71 (d, *J* = 8.5 Hz, 1H, H-6), 7.64 (t, *J* = 7.8 Hz, 1H, H-8), 7.14–7.08 (m, 2H, B-Ar), 7.07–7.00 (m, 4H, B-Ar and H-7 obscured), 6.96 (d, *J* = 8.4 Hz, 1H, H-9) 2.90 (s, 3H, CH_3_) ppm; ^13^C−NMR (DMSO-*d*_6_, 125 MHz) δ 174.6, 160.8, 156.6, 138.7, 138.2, 135.3, 131.9, 131.1, 130.6, 128.9, 128.2, 127.9, 127.3, 127.1, 121.9, 119.9, 119.1, 117.2, 19.2 ppm; HRMS (ESI) *m*/*z* [M+Na]^+^: C_21_H_15_BBrNNaO_3_^+^; calc.: 442.0221; found: 442.0213.

#### 3.5.16. 11-Chloro-7-(4-chlorophenyl)-5H,7H-7λ^4^,14λ^4^-benzo[d]benzo [5,6][1,3,2]oxazaborinino [2,3-b][1,3,2]oxazaborinin-5-one (**33**)

Method C: light-yellow solid; mp: 308–308.5 °C; yield: 79%; IR (neat) cm^−1^: 3726, 2981, 1684 (C=O), 1620 (C=N), 1596, 1587, 1541 (B-N), 1469, 1386, 1334, 1306 (B-O) 1280, 1233, 1205, 1277, 1149, 1087, 1007, 996, 950, 866, 833, 819, 776, 762, 691, 479; ^1^H−NMR (DMSO-*d*_6_, 500 MHz) δ 9.53 (s, 1H, H-5), 8.05–8.00 (m, 2H, H-1, H-4), 7.88–7.82 (m, 2H, H-2, H-6), 7.71 (dd, *J* = 8.9, 2.7 Hz, 1H, H-8), 7.59 (t, *J* = 7.6 Hz, 1H, H-3), 7.14 (brs, 4H, B-Ar), 7.07 (d, *J* = 9.0 Hz, 1H, H-9) ppm; ^13^C−NMR (DMSO-*d*_6_, 125 MHz) δ 161.8, 161.0, 157.0, 139.4, 139.3, 134.9, 132.6, 132.4, 132.0, 130.7, 130.0, 127.6, 123.7, 123.4, 120.8, 119.7, 117.2 ppm; HRMS(ESI) *m*/*z* [M+Na]^+^: C_20_H_12_BCl_2_NNaO_3_^+^; calc.: 418.0180; found: 418.0186.

#### 3.5.17. 11-Chloro-7-(4-chlorophenyl)-3-methyl-5H,7H-7λ^4^,14λ^4^-benzo[d]benzo [5,6][1,3,2]oxazaborinino [2,3-b][1,3,2]oxazaborinin-5-one (**34**)

Method C: dark-yellow solid; mp: 309–310 °C; yield: 89%; IR (neat) cm^−1^: 3015, 1688 (C=O), 1615 (C=N), 1589, 1541 (B-N), 1488, 1469, 1457, 1376, 1316 (B-O), 1218, 1190, 1176, 1089, 1028, 1006, 950, 845, 833, 790, 773, 505, 481; ^1^H−NMR (DMSO-*d*_6_, 500 MHz) δ 9.49 (s, 1H, H-5), 7.92 (d, *J* = 8.3 Hz, 1H, H-4), 7.83–7.80 (m, 2H, H-1, H-6), 7.68 (dd, *J* = 8.9, 2.7 Hz, 1H, H-8), 7.65 (d, *J* = 8.3 Hz, 1H, H-3), 7.14 and 7.11 (AB q, *J* = 8.1 Hz, 4H, B-Ar), 7.05 (d, *J* = 8.9 Hz, 1H, H-9), 2.36 (s, 3H, CH_3_) ppm; ^13^C−NMR (DMSO-*d*_6_, 75 MHz) δ 161.0, 160.7, 156.9, 140.3, 139.0, 137.0, 135.4, 132.5, 132.3, 131.9, 130.8, 127.5, 123.4, 120.7, 119.4, 117.3, 20.6 ppm; HRMS (ESI) *m*/*z* [M+Na]^+^: C_21_H_14_BCl_2_NNaO_3_^+^; calc.: 432.0336; found: 432.0326.

#### 3.5.18. 11-Chloro-7-(4-chlorophenyl)-3-fluoro-5H,7H-7λ^4^,14λ^4^-benzo[d]benzo [5,6][1,3,2]oxazaborinino [2,3-b][1,3,2]oxazaborinin-5-one (**35**)

Method C: light-yellow solid; mp: 288–289 °C; yield: 78%; IR (neat) cm^−1^: 3033, 2981, 2361, 1683 (C=O), 1620 (C=N), 1596, 1541 (B-N), 1497, 1471, 1381, 1329, 1292 (B-O), 1244, 1221, 1182, 1088, 1019, 999, 985, 949, 895, 844, 818, 789, 781, 534, 485, 463; ^1^H−NMR (DMSO-*d*_6_, 500 MHz) δ 9.51 (s, 1H, H-5), 8,11 (dd, ^3^J_H-H_ = 9.0 Hz, ^4^J_H-F_ = 4.3 Hz, 1H, H-4), 7.83 (d, *J* = 2.7 Hz, 1H, H-6), 7.82–7.66 (m, 3H, H-1, H-3, H-8), 7.15 (brs, 4H, B-Ar), 7.07 (d, *J* = 8.9 Hz, 1H, H-9) ppm; ^13^C−NMR (DMSO-*d*_6_, 75 MHz) δ 162.0 (d, ^1^J_C-F_ = 248.2 Hz), 162.0, 160.0 (d, ^4^J_C-F_ = 2.4 Hz), 156.9, 139.3, 136.0 (d, ^4^J_C-F_ = 2.9 Hz), 132.7, 132.4, 132.0, 127.6, 125.8 (d, ^3^J_C-F_ = 7.8 Hz), 123.5, 122.5 (d, ^3^J_C-F_ = 8.6 Hz), 122.2 (d, ^2^J_C-F_ = 23.8 Hz), 120.8, 117.1, 116.8 (d, ^2^J_C-F_ = 24.2 Hz) ppm; HRMS(ESI) *m*/*z* [M+Na]^+^: C_20_H_11_BCl_2_FNNaO_3_^+^; calc.: 436.0085; found: 436.0093.

#### 3.5.19. 3,11-Dichloro-7-(4-chlorophenyl)-5H,7H-7λ^4^,14λ^4^-benzo[d]benzo [5,6][1,3,2]oxazaborinino [2,3-b][1,3,2]oxazaborinin-5-one (**36**)

Method C: orange solid; mp: 325–327 °C; yield: 88%; IR (neat) cm^−1^: 3106, 3046, 2362, 2342, 1694 (C=O), 1614 (C=N), 1600, 1590, 1540 (B-N), 1488, 1467, 1457, 1417, 1376, 1317 (B-O), 1302, 1233, 1203, 1179, 1089, 1052, 1023, 1002, 948, 902, 844, 832, 813, 760, 505, 487; ^1^H−NMR (DMSO-*d*_6_, 500 MHz) δ 9.53 (s, 1H, H-5), 8.06 (d, *J* = 8.4 Hz, 1H, H-4), 7.96 (dd, *J* = 8.8, 2.5 Hz, 1H, H-3), 7.96 (s, 1H, H-1), 7.83 (d, *J* = 2.8 Hz, 1H, H-6), 7.72 (dd, *J* = 9.0, 2.8 Hz, 1H, H-8), 7.16 and 7.13 (ABq, *J* = 8.2 Hz, 4H, B-Ar), 7.07 (d, *J* = 8.9 Hz, 1H, H-9) ppm; ^13^C−NMR (DMSO-*d*_6_, 125 MHz) δ 162.4, 159.9, 157.1, 139.6, 138.4, 134.8, 134.5, 132.7, 132.5, 132.1, 129.9, 127.6, 125.2, 123.6, 122.0, 121.0, 117.1 ppm; HRMS(ESI) *m*/*z* [M+Na]^+^: C_20_H_11_BCl_3_NNaO_3_^+^; calc.: 451.9790; found: 451.9779.

#### 3.5.20. 3-Bromo-11-chloro-7-(4-chlorophenyl)-5H,7H-7λ^4^,14λ^4^benzo[d]benzo [5,6][1,3,2]oxazaborinino [2,3-b][1,3,2]oxazaborinin-5-one (**37**)

Method C: orange solid; mp: 334–336 °C; yield: 91%; IR (neat) cm^−1^: 3103, 3031, 1694 (C=O), 1612 (C=N), 1599, 1539 (B-N), 1466, 1456, 1415, 1374, 1317 (B-O), 1300, 1232, 1202, 1180, 1088, 1001, 948, 901, 843, 830, 812, 789, 752, 503, 483; ^1^H−NMR (DMSO-*d*_6_, 500 MHz) δ 9.54 (s, 1H, H-5), 8.13–8.04 (brs, 2H, H-1, H-4), 7.99 (d, *J* = 8.6 Hz, 1H, H-3), 7.84 (s, 1H, H-6), 7.72 (d, *J* = 8.1 Hz, 1H, H-8), 7.15 (brs, 4H, B-Ar), 7.08 (d, *J* = 9.0 Hz, 1H, H-9) ppm; ^13^C−NMR (DMSO-*d*_6_, 125 MHz) δ 162.4, 159.8, 157.1, 139.6, 138.7, 137.6, 132.9, 132.7, 132.5, 132.0, 127.6, 125.3, 123.6, 122.8, 122.1, 120.9, 117.1 ppm; HRMS(ESI) *m*/*z* [M+Na]^+^: C_20_H_11_BBrCl_2_NNaO_3_^+^; calc.: 495.9285; found: 495.9281.

#### 3.5.21. 11-Chloro-7-(4-chlorophenyl)-3-iodo-5H,7H-7λ^4^,14λ^4^-benzo[d]benzo [5,6][1,3,2]oxazaborinino [2,3-b][1,3,2]oxazaborinin-5-one (**38**)

Method C: dark-yellow solid; mp: 334–335 °C; yield: 83%; IR (neat) cm^−1^: 3733, 3097, 2881, 1688 (C=O), 1613 (C=N), 1597, 1538 (B-N), 1465, 1410, 1374, 1318, 1296 (B-O), 1274, 1233, 1204, 1179, 1088, 1023, 999, 948, 841, 826, 811, 788, 747, 730, 502, 480; ^1^H−NMR (DMSO-*d*_6_, 500 MHz) δ 9.53 (s, 1H, H-5), 8.25 (s, 1H, H-1), 8.23 (d, *J* = 8.0 Hz, 1H, H-4), 7.83 (s, 1H, H-6), 7.82 (d, *J* = 9.1 Hz, 1H, H-3), 7.72 (dd, *J* = 9.0, 2.8 Hz, 1H, H-8), 7.16 and 7.14 (ABq, *J* = 8.1 Hz, 4H, B-Ar), 7.07 (d, *J* = 8.9 Hz, 1H, H-9) ppm; ^13^C−NMR (DMSO-*d*_6_, 125 MHz) δ 162.1, 159.8, 157.1, 143.3, 139.5, 139.1, 138.8, 132.7, 132.5, 132.0, 127.6, 125.0, 123.6, 121.8, 120.9, 117.2, 96.2 ppm; HRMS (ESI) *m*/*z* [M+Na]^+^: C_20_H_11_BCl_2_INNaO_3_^+^; calc.: 543.9146; found: 543.9146.

#### 3.5.22. 2,11-Dichloro-7-(4-chlorophenyl)-5H,7H-7λ^4^,14λ^4^-benzo[d]benzo [5,6][1,3,2]oxazaborinino [2,3-b][1,3,2]oxazaborinin-5-one (**39**)

Method C: dark-yellow solid; mp: 348–348.5 °C; yield: 88%; IR (neat) cm^−1^: 3086, 3020, 1684 (C=O), 1616 (C=N), 1588, 1540 (B-N), 1463, 1425, 1381, 1370, 1326, 1295 (B-O), 1227, 1205, 1178, 1148, 1088, 1027, 1008, 972, 902, 880, 835, 820, 776, 689, 487; ^1^H−NMR (DMSO-*d*_6_, 500 MHz) δ 9.58 (s, 1H, H-5), 8.22 (s, 1H, H-4), 8.00 (d, *J* = 8.4 Hz, 1H, H-2), 7.81 (d, *J* = 2.7 Hz, 1H, H-6), 7.73 (dd, *J* = 9.0, 2.9 Hz, 1H, H-8), 7.65 (d, *J* = 8.4 Hz, 1H, H-1), 7.28 (brs, 4H, B-Ar), 7.08 (d, *J* = 8.9 Hz, 1H, H-9) ppm; ^13^C−NMR (DMSO-*d*_6_, 125 MHz) δ 163.1, 160.2, 157.2, 140.6, 139.8, 139.1, 132.7, 132.5, 132.4, 132.0, 129.9, 127.6, 123.6, 122.5, 121.0, 120.1, 117.0 ppm; HRMS (ESI) *m*/*z* [M+Na]^+^: C_20_H_11_BCl_3_NNaO_3_^+^; calc.: 451.9790; found: 451.9790.

#### 3.5.23. 11-Chloro-7-(4-chlorophenyl)-2-nitro-5H,7H-7λ^4^,14λ^4^-benzo[d]benzo [5,6][1,3,2]oxazaborinino [2,3-b][1,3,2]oxazaborinin-5-one (**40**)

Method C: orange solid; mp: 348–349 °C; yield: 90%; IR (neat) cm^−1^: 3093, 3049, 1695 (C=O), 1616 (C=N), 1590, 1541, (B-N), 1530, 1461, 1433, 1376, 1323 (B-O), 1296, 1206, 1181, 1088, 1021, 1002, 985, 906, 840, 817, 791, 779, 746, 682, 490, 479; ^1^H−NMR (DMSO-*d*_6_, 500 MHz) δ 9.7 (s, 1H, H-5), 8.96 (d, *J* = 2.1 Hz, 1H, H-4), 8.34 (dd, *J* = 8.7, 2.1 Hz, 1H, H-2), 8.25 (d, *J* = 8.6 Hz, 1H, H-1), 7.90 (d, *J* = 2.8 Hz, 1H, H-6), 7.76 (dd, *J* = 8.9, 2.8 Hz, 1H, H-8), 7.18 and 7.16 (ABq, *J* = 8.3 Hz, 4H, B-Ar), 7.10 (d, *J* = 9.0 Hz, 1H, H-9) ppm; ^13^C−NMR (DMSO-*d*_6_, 125 MHz) δ 164.2, 159.6, 157.3, 150.9, 140.5, 140.1, 132.8, 132.8, 132.4, 132.2, 128.4, 127.7, 124.1, 123.7, 121.0, 116.9, 115.7 ppm; HRMS (ESI) *m*/*z* [M+Na]^+^: C_20_H_11_BCl_2_N_2_NaO_5_^+^; calc.: 463.0030; found: 463.0035.

#### 3.5.24. 11-Bromo-7-(4-bromophenyl)-5H,7H-7λ^4^,14λ^4^-benzo[d]benzo [5,6][1,3,2]oxazaborinino [2,3-b][1,3,2]oxazaborinin-5-one (**41**)

Method C: light-yellow solid; mp: 318 °C; yield: 95%; IR (neat) cm^−1^: 3030, 1683 (C=O), 1616 (C=N), 1539 (B-N), 1466, 1384, 1332, 1306 (B-O), 1281, 1231, 1199, 1177, 1068, 1006, 948, 863, 830, 815, 777, 763, 694, 581, 479; ^1^H−NMR (DMSO-*d*_6_, 500 MHz) δ 9.54 (s, 1H, H-5), 8.04 (d, *J* = 8.1 Hz, 2H, H-1, H-4), 7.99 (s, 1H, H-6), 7.88–7.82 (m, 2H, H-2, H-8), 7.60 (t, *J* = 7.7 Hz, 1H, H-3), 7.30 (d, *J* = 7.9 Hz, 2H, B-Ar), 7.10 (d, *J* = 7.8 Hz, 2H, B-Ar) 7.03 (d, *J* = 8.9 Hz, 1H, H-9)ppm; ^13^C−NMR (DMSO-*d*_6_, 125 MHz) δ 161.8, 161.0, 157.3, 141.9, 139.4, 135.5, 134.9, 132.3, 130.7, 130.5, 130.0, 123.7, 121.4, 121.2, 119.7, 117.9, 110.7 ppm; HRMS (ESI) *m*/*z* [M+Na]^+^: C_20_H_12_BBr_2_NNaO_3_^+^; calc.: 505.9169; found: 505.9168.

#### 3.5.25. 11-Bromo-7-(4-bromophenyl)-3-methyl-5H,7H-7λ^4^,14λ^4^-benzo[d]benzo [5,6][1,3,2]oxazaborinino [2,3-b][1,3,2]oxazaborinin-5-one (**42**)

Method C: dark-yellow solid; mp: 304–306 °C; yield: 98%; IR (neat) cm^−1^: 3049, 1689 (C=O), 1620 (C=N), 1584, 1542 (B-N), 1488, 1469, 1367, 1310 (B-O), 1293, 1288, 1216, 1197, 1177, 1074, 1038, 998, 985, 944, 840, 832, 813, 792, 683, 548, 514; ^1^H−NMR (DMSO-*d*_6_, 500 MHz) δ 9.51 (s, 1H, H-5), 7.97 (s, 1H, H-6), 7.93 (d, *J* = 8.3 Hz, 1H, H-4), 7.84 (s, 1H, H-1), 7.81 (dd, *J* = 8.7, 2.6 Hz, 1H, H-8), 7.67 (d, *J* = 8.3 Hz, 1H, H-3), 7.30 (d, *J* = 7.8 Hz, 2H, B-Ar), 7.08 (d, *J* = 7.9 Hz, 2H, B-Ar), 7.02 (d, *J* = 8.9 Hz, 1H, H-9), 2.38 (s, 3H, CH_3_) ppm; ^13^C−NMR (DMSO-*d*_6_, 75 MHz) δ 161.1, 160.7, 157.2, 141.7, 140.4, 137.0, 135.5, 135.4, 132.3, 130.8, 130.5, 123.4, 121.4, 121.1, 119.5, 118.0, 110.7, 20.6 ppm; HRMS (ESI) *m*/*z* [M+K]^+^: C_21_H_14_BBr_2_KNO_3_^+^; calc.: 535.9065; found: 535.9077.

#### 3.5.26. 11-Bromo-7-(4-bromophenyl)-3-fluoro-5H,7H-7λ^4^,14λ^4^-benzo[d]benzo [5,6][1,3,2]oxazaborinino [2,3-b][1,3,2]oxazaborinin-5-one (**43**)

Method C: light-yellow solid; mp: 284–285 °C; yield: 92%; IR (neat) cm^−1^: 3073, 3020, 1694 (C=O), 1620 (C=N), 1584, 1540 (B-N), 1493, 1468, 1443, 1370, 1312 (B-O), 1285, 1224, 1178, 1149, 1082, 1026, 990, 948, 923, 844, 839, 831, 813, 794, 520; ^1^H−NMR (DMSO-*d*_6_, 500 MHz) δ 9.52 (s, 1H, H-5), 8.12 (s, 1H, H-1), 7.98 (s, 1H, H-6), 7.82–7.78 (m, 3H, H-4, H-3, H-8), 7.31 (d, *J* = 7.6 Hz, 2H, B-Ar), 7.10 (d, *J* = 7.9 Hz, 2H, B-Ar), 7.03 (d, *J* = 8.9 Hz, 1H, H-9) ppm; ^13^C−NMR (DMSO-*d*_6_, 75 MHz) δ 161.9 (d, ^1^J_C-F_ = 247.7 Hz), 161.9, 159.9 (d, ^4^J_C-F_ = 1.6 Hz), 157.2, 142.0, 136.0 (d, ^4^J_C-F_ = 1.4 Hz), 135.4, 132.3, 130.5, 125.8 (d, ^3^J_C-F_ = 7.8 Hz), 122.5 (d, ^3^J_C-F_ = 9.0 Hz), 122.2 (d, ^2^J_C-F_ = 23.9 Hz), 121.5, 121.1, 117.8, 117.0 (d, ^2^J_C-F_ = 24.1 Hz), 110.7 ppm; HRMS (ESI) *m*/*z* [M+Na]^+^: C_20_H_11_BBr_2_FNNaO_3_^+^; calc.: 523.9075; found: 523.9074.

#### 3.5.27. 11-Bromo-7-(4-bromophenyl)-3-chloro-5H,7H-7λ^4^,14λ^4^-benzo[d]benzo [5,6][1,3,2]oxazaborinino [2,3-b][1,3,2]oxazaborinin-5-one (**44**)

Method C: light-orange solid; mp: 312–313 °C; yield: 90%; IR (neat) cm^−1^: 3079, 1692 (C=O), 1615 (C=N), 1584, 1539 (B-N), 1467, 1301 (B-O), 1276, 1232, 1199, 1178, 1080, 1030, 987, 944, 903, 834, 804, 791, 759, 546, 514, 492; ^1^H−NMR (DMSO-*d*_6_, 500 MHz) δ 9.55 (s, 1H, H-5), 8.08 (d, *J* = 8.5 Hz, 1H, H-4), 8.00 (dd, *J* = 8.0, 2.5 Hz, 1H, H-3), 7.98 (d, *J* = 2.7 Hz, 2H, H-1, H-6), 7.85 (dd, *J* = 2.7, 9.0 Hz, 1H, H-8), 7.32 (d, *J* = 7.8 Hz, 2H, B-Ar), 7.10 (d, *J* = 7.8 Hz, 2H, B-Ar), 7.04 (d, *J* = 9.0 Hz, 1H, H-9) ppm; ^13^C−NMR (DMSO-*d*_6_, 125 MHz) δ ^13^C NMR (126 MHz, dmso) δ 162.3, 159.9, 157.4, 142.2, 138.3, 135.6, 134.8, 134.5, 132.4, 130.5, 129.9, 125.2, 122.0, 121.6, 121.2, 117.8, 110.8 ppm; HRMS(ESI) *m*/*z* [M+Na]^+^: C_20_H_11_BBr_2_ClNNaO_3_^+^; calc.: 539.8779; found: 539.8778.

#### 3.5.28. 3,11-Dibromo-7-(4-bromophenyl)-5H,7H-7λ^4^,14λ^4^-benzo[d]benzo [5,6][1,3,2]oxazaborinino [2,3-b][1,3,2]oxazaborinin-5-one (**45**)

Method C: light-orange solid; mp: 319–320 °C; yield: 88%; IR (neat) cm^−1^: 3076, 1691 (C=O), 1615 (C=N), 1584, 1539 (B-N), 1465, 1410, 1365, 1308 (B-O), 1275, 1232, 1198, 1178, 1076, 1054, 1030, 986, 944, 837, 803, 751, 544, 513; ^1^H−NMR (DMSO-*d*_6_, 500 MHz) δ 9.55 (s, 1H, H-5), 8.12 (dd, *J* = 8.7, 2.2 Hz, 2H, H-4, H-3), 8.00 (s, 1H, H-1), 8.00 (d, *J* = 2.5 Hz, 1H, H-6), 7.85 (dd, *J* = 9.0, 2.6 Hz, 1H, H-8), 7.32 (d, *J* = 7.9 Hz, 2H, B-Ar), 7.10 (d, *J* = 7.6 Hz, 2H, B-Ar), 7.04 (d, *J* = 8.8 Hz, 1H, H-9) ppm; ^13^C−NMR (DMSO-*d*_6_, 125 MHz) δ ^13^C NMR (126 MHz, dmso) δ 162.3, 159.8, 157.4, 142.2, 138.7, 137.6, 135.6, 132.9, 132.4, 130.5, 125.3, 122.8, 122.1, 121.6, 121.2, 117.8, 110.8 ppm; HRMS(ESI) *m*/*z* [M+Na]^+^: C_20_H_11_BBr_3_NNaO_3_^+^; calc.: 583.8274; found: 583.8265.

#### 3.5.29. 11-Bromo-7-(4-bromophenyl)-3-iodo-5H,7H-7λ^4^,14λ^4^-benzo[d]benzo [5,6][1,3,2]oxazaborinino [2,3-b][1,3,2]oxazaborinin-5-one (**46**)

Method C: light-orange solid; mp: 325–327 °C; yield: 86%; IR (neat) cm^−1^: 3068, 1695 (C=O), 1683, 1615 (C=N), 1581, 1538 (B-N), 1462, 1407, 1367, 1312 (B-O), 1292, 1233, 1198, 1177, 1071, 1025, 993, 947, 842, 807, 745, 545; ^1^H−NMR (DMSO-*d*_6_, 500 MHz) δ 9.55 (s, 1H, H-5), 8.27 (s, 1H, H-1), 8.26 (d, *J* = 10.8 Hz, 1H, H-4), 7.98 (s, 1H, H-6), 7.83 (d, *J* = 8.3 Hz, 2H, H-3, H-8), 7.32 (d, *J* = 7.8 Hz, 2H, B-Ar), 7.09 (d, *J* = 7.8 Hz, 2H, B-Ar), 7.03 (d, *J* = 9.0 Hz, 1H, H-9) ppm; ^13^C−NMR (DMSO-*d*_6_, 125 MHz) δ 162.0, 159.8, 157.4, 143.3, 142.2, 139.1, 138.8, 135.6, 132.4, 130.5, 125.0, 121.7, 121.5, 121.2, 117.8, 110.8, 96.3 ppm; HRMS(ESI) *m*/*z* [M+Na]^+^: C_20_H_11_BBr_2_INNaO_3_^+^; calc.: 631.8136; found: 631.8125.

#### 3.5.30. 11-Bromo-7-(4-bromophenyl)-2-chloro-5H,7H-7λ^4^,14λ^4^-benzo[d]benzo [5,6][1,3,2]oxazaborinino [2,3-b][1,3,2]oxazaborinin-5-one (**47**)

Method C: light-orange solid; mp: 343–344 °C; yield: 96%; IR (neat) cm^−1^: 3753, 3017, 1680 (C=O), 1614 (C=N), 1591, 1535 (B-N), 1459, 1424, 1378, 1327, 1294 (B-O), 1227, 1204, 1176, 1146, 1087, 1070, 992, 973, 911, 900, 879, 833, 816, 775, 688, 569, 480; ^1^H−NMR (DMSO-*d*_6_, 500 MHz) δ 9.59 (s, 1H, H-5), 8.23 (s, 1H, H-4), 8.02 (d, *J* = 8.4 Hz, 1H, H-2), 7.95 (s, 1H, H-6), 7.85 (d, *J* = 8.3 Hz, 1H, H-8), 7.67 (d, *J* = 8.4 Hz, 1H, H-1), 7.33 (d, *J* = 7.9 Hz, 2H, B-Ar), 7.11 (d, *J* = 7.9 Hz, 2H, B-Ar), 7.04 (d, *J* = 9.0 Hz, 1H, H-9) ppm; ^13^C−NMR (DMSO-*d*_6_, 125 MHz) δ 163.0, 160.2, 157.5, 142.4, 140.6, 139.1, 135.6, 132.4, 132.4, 130.5, 129.9, 122.5, 121.6, 121.3, 120.1, 117.7, 110.9 ppm; HRMS (ESI) *m*/*z* [M+Na]^+^: C_20_H_11_BBr_2_ClNNaO_3_^+^; calc.: 539.8779; found: 539.8769.

#### 3.5.31. 11-Bromo-7-(4-bromophenyl)-2-nitro-5H,7H-7λ^4^,14λ^4^-benzo[d]benzo [5,6][1,3,2]oxazaborinino [2,3-b][1,3,2]oxazaborinin-5-one (**48**)

Method C: light-orange solid; mp: 349–350 °C; yield: 82%; IR (neat) cm^−1^: 3091, 1694 (C=O), 1614 (C=N), 1589, 1527 (B-N), 1464, 1431, 1372, 1349, 1322 (B-O), 1295, 1204, 1179, 1136, 1070, 1021, 1002, 985, 921, 904, 874, 838, 813, 790, 739, 729, 681, 537, 485; ^1^H−NMR (DMSO-*d*_6_, 500 MHz) δ 9.71 (s, 1H, H-5), 8.98 (s, 1H, H-4), 8.35 (d, *J* = 8.5 Hz, 1H, H-2), 8.27 (d, *J* = 8.5 Hz, 1H, H-1), 8.05 (s, 1H, H-6), 7.88 (d, *J* = 9.0 Hz, 1H, H-8), 7.32 (d, *J* = 7.9 Hz, 2H, B-Ar), 7.14 (d, *J* = 7.9 Hz. 2H, B-Ar), 7.06 (d, *J* = 9.0 Hz, 1H, H-9) ppm; ^13^C−NMR (DMSO-*d*_6_, 125 MHz) δ 164.1, 159.6, 157.6, 150.9, 142.7, 140.5, 135.9, 132.5, 130.6, 128.4, 124.2, 121.7, 121.3, 117.6, 115.7, 111.0 ppm; HRMS(ESI) *m*/*z* [M+Na]^+^: C_20_H_11_BBr_2_N_2_NaO_5_^+^; calc.: 550.9020; found: 550.9015.

### 3.6. Interaction with CT DNA

UV–vis spectra of compounds **18**–**24** and **26**–**48** were obtained for freshly prepared solutions in DMSO (Supporting Information part 2: Section S.1). The interaction of the compounds with CT DNA was evaluated in vitro using their solutions in DMSO (1 mM) due to their low solubility in water. These studies were performed in the presence of aqueous buffer solutions, where mixing of each solution never exceeded 5% DMSO (*v*/*v*) in the final solution. Control experiments were undertaken to assess the effect of DMSO on the data, and no changes were observed in the spectra of CT DNA. The interaction of the compounds with CT DNA was investigated using UV–vis spectroscopy, viscosity measurements, and via the evaluation of their EB-displacing ability, which was studied using fluorescence emission spectroscopy. Detailed procedures and equations regarding the in vitro study of the interaction of the compounds with CT DNA are given in the Supporting Information file (Supporting Information part 2: Section S.2). Fluorescent spectra of all derivatives are given in Supporting Information part 2: Section S.3.

## 4. Conclusions

The investigation of the most environmentally safe and green conditions for the synthesis of bridgehead bicyclo[4.4.0]boron heterocycles has led to the selection of the solvent system EtOH/H_2_O at a ratio of 1/3 that produced, under microwave irradiation for 1 h and in very high yields, twenty-six derivatives, out of which only two were previously known. The methodology afforded the assembly via the condensation and dehydration reactions of AA, SA and PBA. SA, as one component of the triadic system, tolerated a wide variety of substituents on AA, such as electron-withdrawing and electron-donating groups, probably giving the initial in situ formation of the intermediate imine, which was further condensed with PBA. An exception was the 5-NO_2_ AA, where the -I and -R substituents gave very unstable or no products. In total, three SAs as well as three ABAs, free of substituents or as their Cl and Br derivatives, have been used as counterparts of ten AAs. With the less-activated Cl- and Br-substituted SAs and ABAs, the reactions did not work with 5-NO_2_ and 5-OH AAs, and thus, the corresponding BASAN-Cl and BASAN-Br derivatives were not formed.

To the best of our knowledge, for the first time, the reaction of *o*-hydroxyacetophenone instead of SA with the same counterparts has been attempted, and the synthesis required the use of toluene and MSs in order to afford the related boron heterocycle under MWI. Nevertheless, highly electron-withdrawing groups, such as NO_2_ on AAs, were not tolerated. The reason is probably the limited nucleophilicity of the NH_2_ of an AA being in the *m*- and, most importantly, in the *p*-positions of the NO_2_ group. This less nucleophilic NH_2_ group had to compete with the bulkiness of the Me group for its reaction with a carbonyl group. The reactions were sluggish with hydroxyl- and iodo-AA as well. Therefore, based on the synthetic observations and the three proposed Methods, A, B and C, these approaches allow for the use of a wide variety of functionalities and the preparation of good to excellent yields of bridgehead bicyclo[4.4.0]boron heterocycles.

All compounds were studied for their affinity to calf thymus DNA using proper techniques like viscosity and UV–vis spectroscopy, where K_b_ values were found in the range 2.83 × 10^4^–8.41 × 10^6^ M^−1^. Additionally, the EB-displacement experiment using fluorescence spectroscopy indicated K_SV_ values between 1.49 × 10^4^ and 5.36 × 10^4^ M^−1^, whereas the quenching constants (K_q_) of the compounds were calculated to be between 6.46 × 10^11^ and 2.33 × 10^12^ M^−1^ s^−1^. All derivatives showed a good intercalation profile towards CT DNA, better than the reference compound EB. Although the list of the prepared derivatives and their substituents was not exhaustive, it may be rather safe to conclude that based on the stability experiments in solution or upon heating, it seems that highly electron-deficient components should be avoided in the assembly and, in particular, those containing nitro groups. Other than that, the efficient synthesis and the good intercalative properties of the bridgehead boron[4.4.0]heterocycles and, in particular cases, their slow or controlled disassembly give promise for their use as lead compounds for possible pharmaceuticals with applications for DNA-related diseases and/or as carriers of drugs targeting DNA, respectively.

## Figures and Tables

**Table 1:** UV–vis spectral features of the interaction of the compounds **18**–**24** and **26**–**48** with increasing amounts of CT DNA. UV band (λ, in nm) (percentage of the observed hyper-/hypo-chromism (ΔA/A_0_, %), blue-/red-shift of the λ_max_ (Δλ, nm)) and DNA-binding constants (K_b_, in M^−1^).

	Compound	λ_max_(nm) ((ΔA/A_0_) ^a^ (in %), Δλ (in nm) ^b^)	Κ_b_ (M^−1^)
	**18**	316 (<−50 ^c^, +3); 408 (<−50, −3)	3.89 (± 0.35) × 10^5^
BASAN COMPOUNDS	**19**	321 (−26 ^a^, +1 ^b^); 408 (−25, −5 ^b^)	1.63 (± 0.06) × 10^6^
	**20**	335 (−19, +2); 411 (−18, +2)	1.75 (± 0.31) × 10^6^
	**21**	318 (<−50, +5); 405 (−45, −17)	3.37 (± 0.27) × 10^5^
	**22**	321 (<−50, +3); 409 (<−50, −31)	4.17 (± 0.28) × 10^5^
	**23**	319 (<−50, +3/Elim ^d^); 410 (<−50, −33)	6.38 (± 0.27) × 10^5^
	**24**	295 (+9 ^a^, −1); 326 (−41, +1); 408 (−46, −15)	2.83 (± 0.12) × 10^4^
	**26**	313 (−11, −30); 399 (<−50, Elim)	1.59 (± 0.07) × 10^6^
	**27**	309 (<−50, Elim); 408 (<−50, +42)	1.10 (± 0.06) × 10^6^
BACAN COMPOUNDS	**28**	302 (−36, +12); 387 (−25, −22)	1.24 (± 0.08) × 10^6^
	**29**	300 (sh) ^e^ (−20, +10); 387 (−40, −7)	8.41 (± 0.13) × 10^6^
	**30**	303 (−16, +4); 387 (<−50, −3)	1.68 (± 0.11) × 10^5^
	**31**	308 (−30, +11); 388 (−43, −5)	4.17 (± 0.24) × 10^5^
	**32**	307 (−31, +10); 388 (<−50, −4)	2.73 (± 0.07) × 10^5^
	**33**	310 (−37, −15); 413 (−43, −51)	2.97 (± 0.07) × 10^6^
BASAN-Cl COMPOUNDS	**34**	315 (−20, −18); 414 (<−50, −45/Elim)	1.54 (± 0.06) × 10^6^
	**35**	307 (−36, −14); 412 (−39, −36)	5.68 (± 0.06) × 10^6^
	**36**	312 (−10, −20); 416 (<−50, −43/Elim)	2.64 (± 0.31) × 10^6^
	**37**	313 (−12, −18); 417 (<−50, −40/Elim)	3.12 (± 0.32) × 10^5^
	**38**	326 (+3, −28); 420 (<−50, −50)	1.58 (± 0.07) × 10^6^
	**39**	307 (−30, −15); 417 (−40, −52)	2.08 (± 0.08) × 10^6^
	**40**	297 (−5, −6); 418 (<−5, +27)	3.74 (± 0.26) × 10^5^
	**41**	268 (+8, +1); 312 (−45, −15); 409 (<−50, −6)	1.39 (± 0.07) × 10^6^
BASAN-Br COMPOUNDS	**42**	267 (+6, 0); 324 (<−50, −15); 415 (−45, +6)	3.38 (± 0.10) × 10^6^
	**43**	268 (+22, +2); 318 (<−50, −18); 412 (−40, −20)	1.78 (± 0.09) × 10^6^
	**44**	317 (−25, −25); 416 (<−50, Elim)	1.53 (± 0.61) × 10^6^
	**45**	276 (+18, +9); 314 (<−50, +40); 417 (<−50, Elim)	3.81 (± 0.39) × 10^5^
	**46**	278 (+12, +14); 328 (<−50, +25); 418 (<−50, +10)	1.11 (± 0.06) × 10^6^
	**47**	272 (+16, +2); 330 (−39, +4); 404 (−13, +20)	2.67 (± 0.09) × 10^6^
	**48**	274 (+14, 0); 302 (−30, Elim); 420 (<−50, +30)	1.02 (± 0.07) × 10^6^

^a^ “+” denotes hyperchromism, and “−” denotes hypochromism; ^b^ “+” denotes red-shift, and “−” denotes blue-shift; ^c^ “<−50” = intense hypochromism, and “>+50” = intense hyperchromism; ^d^ “Elim” = eliminated/disappeared; ^e^ “sh” = shoulder.

**Table 2:** Data from the EB-DNA competitive studies for compounds **18**–**24** and **26**–**48**. Percentage of EB-DNA fluorescence quenching (ΔI/I_o_, %), EB-DNA Stern–Volmer constants (K_SV_, M^−1^) and EB-DNA quenching constants (K_q_, M^−1^ s^−1^) for compounds **18**–**24** and **26**–**48**.

	Compound	∆I/I_o_ (%)	K_SV_ (M^−1^)	k_q_, M^−1^ s^−1^
BASAN COMPOUNDS	**18**	30.5	1.84 (± 0.03) × 10^4^	8.00 (± 0.11) × 10^11^
	**19**	29.8	1.59 (± 0.03) × 10^4^	6.93 (± 0.13) × 10^11^
	**20**	26.5	1.49 (± 0.04) × 10^4^	6.46 (± 0.17) × 10^11^
	**21**	29.5	1.61 (± 0.04) × 10^4^	6.98 (± 0.18) × 10^11^
	**22**	50.7	4.00 (± 0.11) × 10^4^	1.74 (± 0.05) × 10^12^
	**23**	51.9	4.55 (± 0.09) × 10^4^	1.98 (± 0.04) × 10^12^
	**24**	54.9	4.88 (± 0.13) × 10^4^	2.12 (± 0.06) × 10^12^
	**26**	52.9	4.70 (± 0.07) × 10^4^	2.04 (± 0.03) × 10^12^
	**27**	55.8	4.78 (± 0.13) × 10^4^	2.08 (± 0.06) × 10^12^
BACAN COMPOUNDS	**28**	40.1	2.82 (± 0.07) × 10^4^	1.23 (± 0.03) × 10^12^
	**29**	52.5	4.18 (± 0.10) × 10^4^	1.82 (± 0.04) × 10^12^
	**30**	56.9	5.13 (± 0.08) × 10^4^	2.23 (± 0.04) × 10^12^
	**31**	48.7	3.74 (± 0.06) × 10^4^	1.62 (± 0.03) × 10^12^
	**32**	52.1	4.68 (± 0.08) × 10^4^	2.04 (± 0.03) × 10^12^
BASAN-Cl COMPOUNDS	**33**	51.4	4.11 (± 0.07) × 10^4^	1.79 (± 0.03) × 10^12^
	**34**	42.0	3.02 (± 0.06) × 10^4^	1.31 (± 0.03) × 10^12^
	**35**	53.9	4.72 (± 0.14) × 10^4^	2.05 (± 0.06) × 10^12^
	**36**	55.7	4.96 (± 0.08) × 10^4^	2.16 (± 0.03) × 10^12^
	**37**	46.4	3.44 (± 0.07) × 10^4^	1.50 (± 0.03) × 10^12^
	**38**	53.7	4.46 (± 0.10) × 10^4^	1.94 (± 0.04) × 10^12^
	**39**	54.5	4.81 (± 0.05) × 10^4^	2.09 (± 0.02) × 10^12^
	**40**	54.5	4.80 (± 0.09) × 10^4^	2.09 (± 0.04) × 10^12^
BASAN-Br COMPOUNDS	**41**	55.7	4.60 (± 0.06) × 10^4^	2.00 (± 0.03) × 10^12^
	**42**	57.2	5.28 (± 0.06) × 10^4^	2.29 (± 0.02) × 10^12^
	**43**	55.8	5.12 (± 0.09) × 10^4^	2.22 (± 0.04) × 10^12^
	**44**	52.6	4.22 (± 0.11) × 10^4^	1.84 (± 0.05) × 10^12^
	**45**	58.3	5.13 (± 0.12) × 10^4^	2.23 (± 0.05) × 10^12^
	**46**	53.1	4.46 (± 0.07) × 10^4^	1.94 (± 0.03) × 10^12^
	**47**	57.0	5.36 (± 0.13) × 10^4^	2.33 (± 0.06) × 10^12^
	**48**	53.0	4.23 (± 0.11) × 10^4^	1.84 (± 0.05) × 10^12^

## Data Availability

Data is contained within the article and Supplementary Material.

## Supplementary Materials

The following supporting information can be downloaded at: https://www.mdpi.com/article/10.3390/ijms25189842/s1.
