# Supplementary material for: One-Pot, Multi-Component Green Microwave-Assisted Synthesis of Bridgehead Bicyclo[4.4.0]boron Heterocycles and DNA Affinity Studies
Source: Int J Mol Sci. 2024 Sep 12;25(18):9842. doi: 10.3390/ijms25189842 (PMC11432172; doi:10.3390/ijms25189842)

Supporting Information part 1

One-Pot Multi-Component Green Microwave Assisted Synthesis of Bridgehead Bicyclo[4.4.0]boron Heterocycles and DNA Affinity Studies

Polinikis Paisidis,[1] Maroula G. Kokotou,[2] Antigoni Kotali,[3] George Psomas,[4] Konstantina C. Fylaktakidou[1,*]

[1] Laboratory of Organic Chemistry, Aristotle University of Thessaloniki, Chemistry Department of Chemistry, 54124 Thessaloniki, Greece, email: ppaisidis@chem.auth.gr
[2] Laboratory of Chemistry, Department of Food Science and Human Nutrition, Agricultural University of Athens, Iera Odos 75, Athens 11855, Greece, email: mkokotou@aua.gr
[3] Laboratory of Organic Chemistry, Aristotle University of Thessaloniki, Department of Chemical Engineering, 54124 Thessaloniki, Greece, email: kotali@cheng.auth.gr
[4] Laboratory of Inorganic Chemistry, Aristotle University of Thessaloniki, Department of Chemistry, 54124 Thessaloniki, Greece, email: gepsomas@chem.auth.gr
Corresponding Author: email: kfylakta@chem.auth.gr

Table of contents

S.1. Copies of NMR spectra of compounds 18-48 3

S.2. Copies of NMR spectra of compounds 18-24,26-48 at 0, 12, 24, 48 and 72 h intervals……………………………….......64

S.3. Time dependent comparative NMR spectra of BASAN (18-24,26-27), BASAN-Cl (33-40) and BASAN-Br (41-48) derived from the same anthranilic acid……81

S.4. Time dependent comparative NMR spectra of BASAN and BACAN derived from the same anthranilic acid…..…84

S.1. Copies of NMR spectra of compounds 18-48

7-phenyl-5H,7H-7λ[4],14λ[4]-benzo[d]benzo[5,6][1,3,2]oxazaborinino[2,3-b][1,3,2]oxa-zaborinin-5-one **(18)** **^{1}H-NMR spectrum**

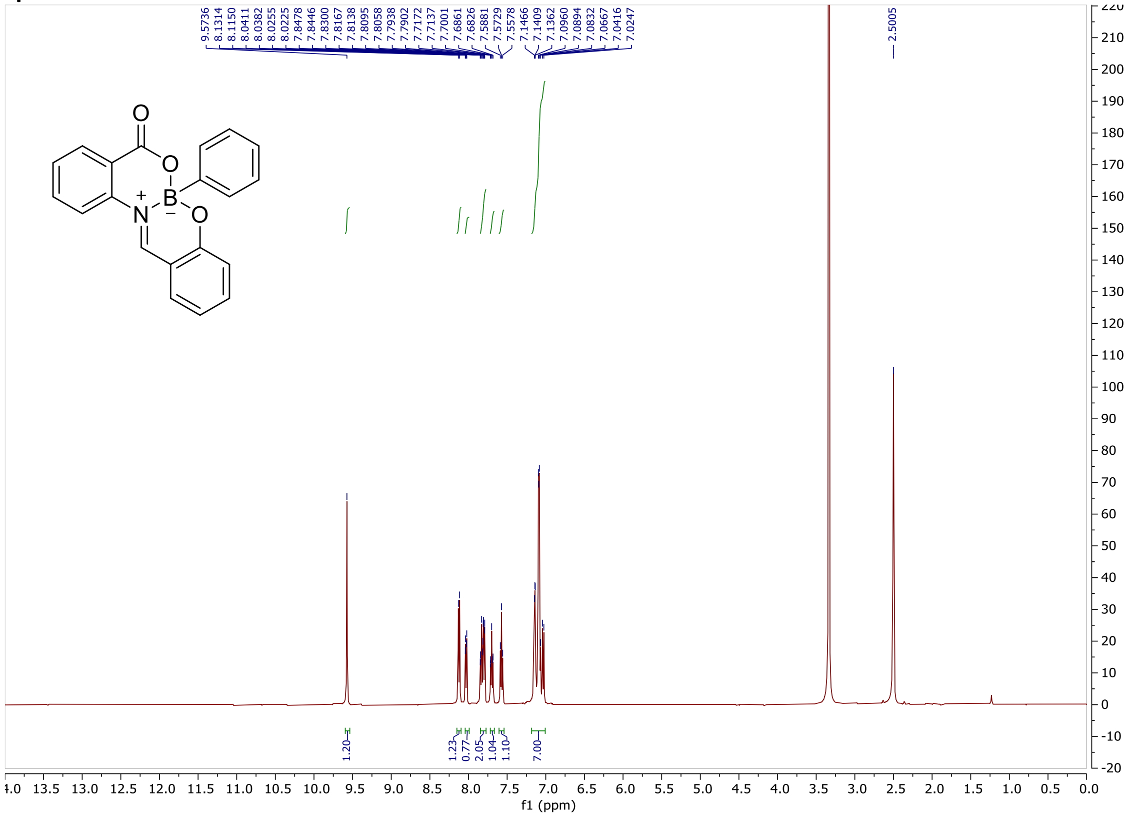

^{13}C-NMR spectrum

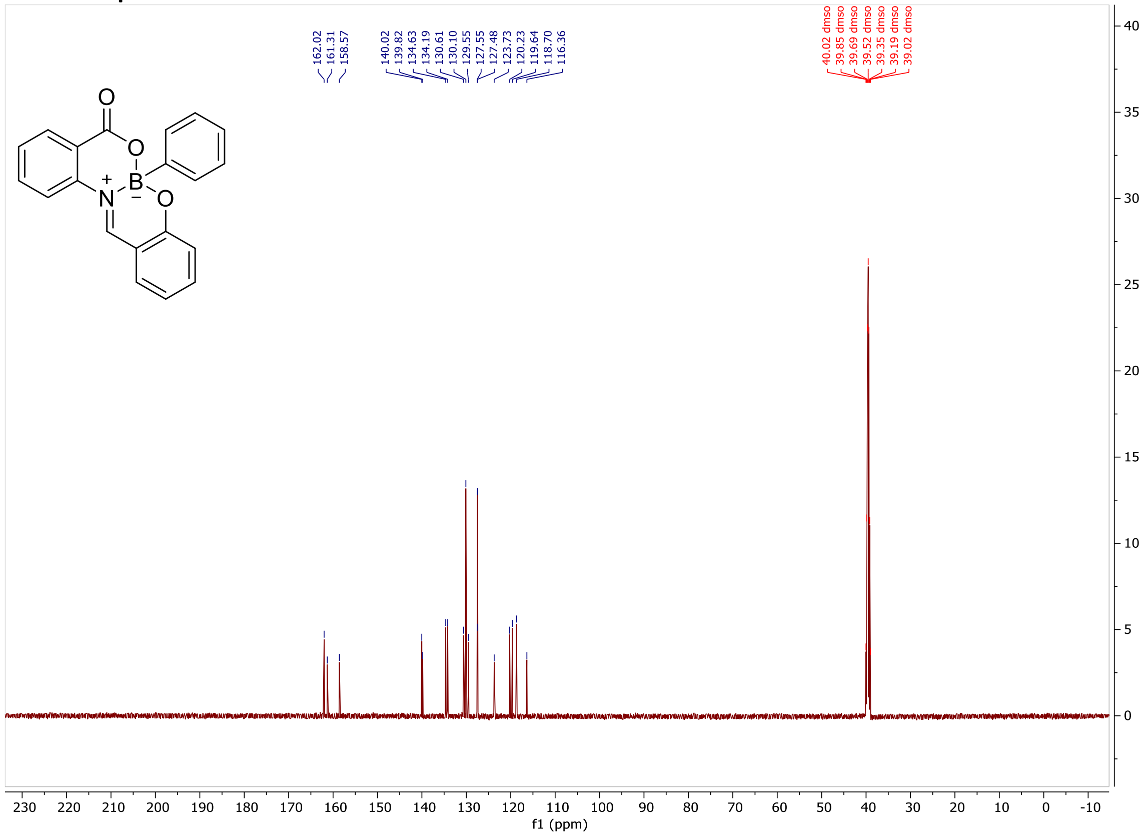

(18) ^{1}H-NMR spectrum Bottom: crude from method C**; Top:** recrystallized from PS/Ethyl acetate

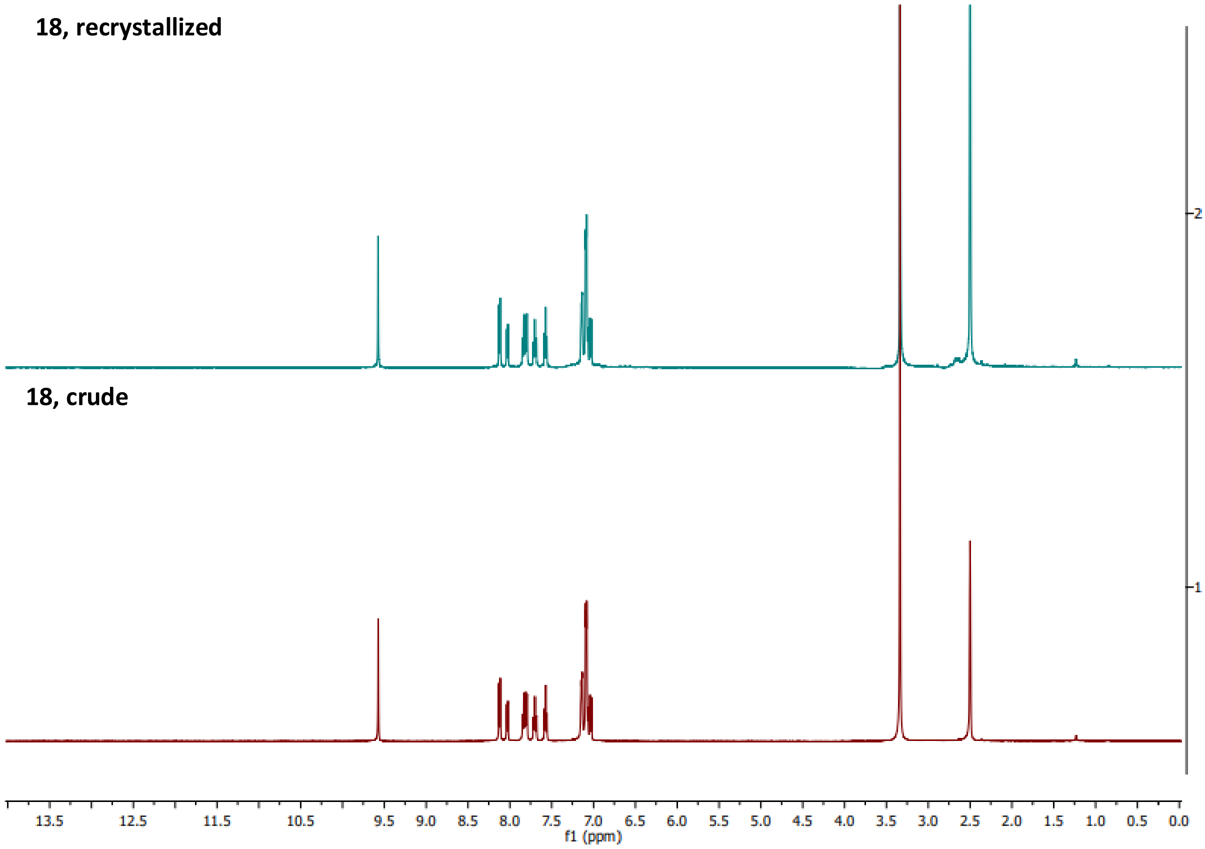

(18) HRMS m/z 353 and 381 is Eppendorf plasticizer

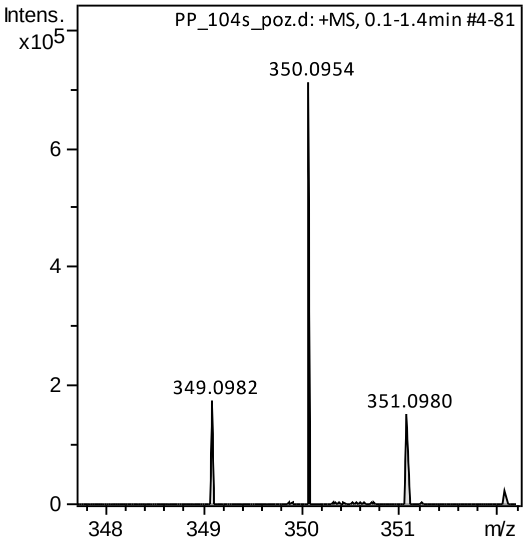

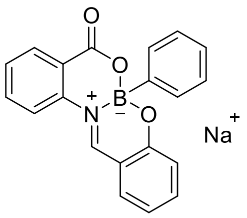

Chemical Formula: $C_{20}H_{14}BNNaO_3^+$
Exact Mass: 350.0959

3-methyl-7-phenyl-5H,7H-7λ4,14λ4-benzo[d]benzo[5,6][1,3,2]oxazaborinino[2,3-b][1,3,2]oxazaborinin-5-one **(19)** **^{1}H-NMR spectrum**

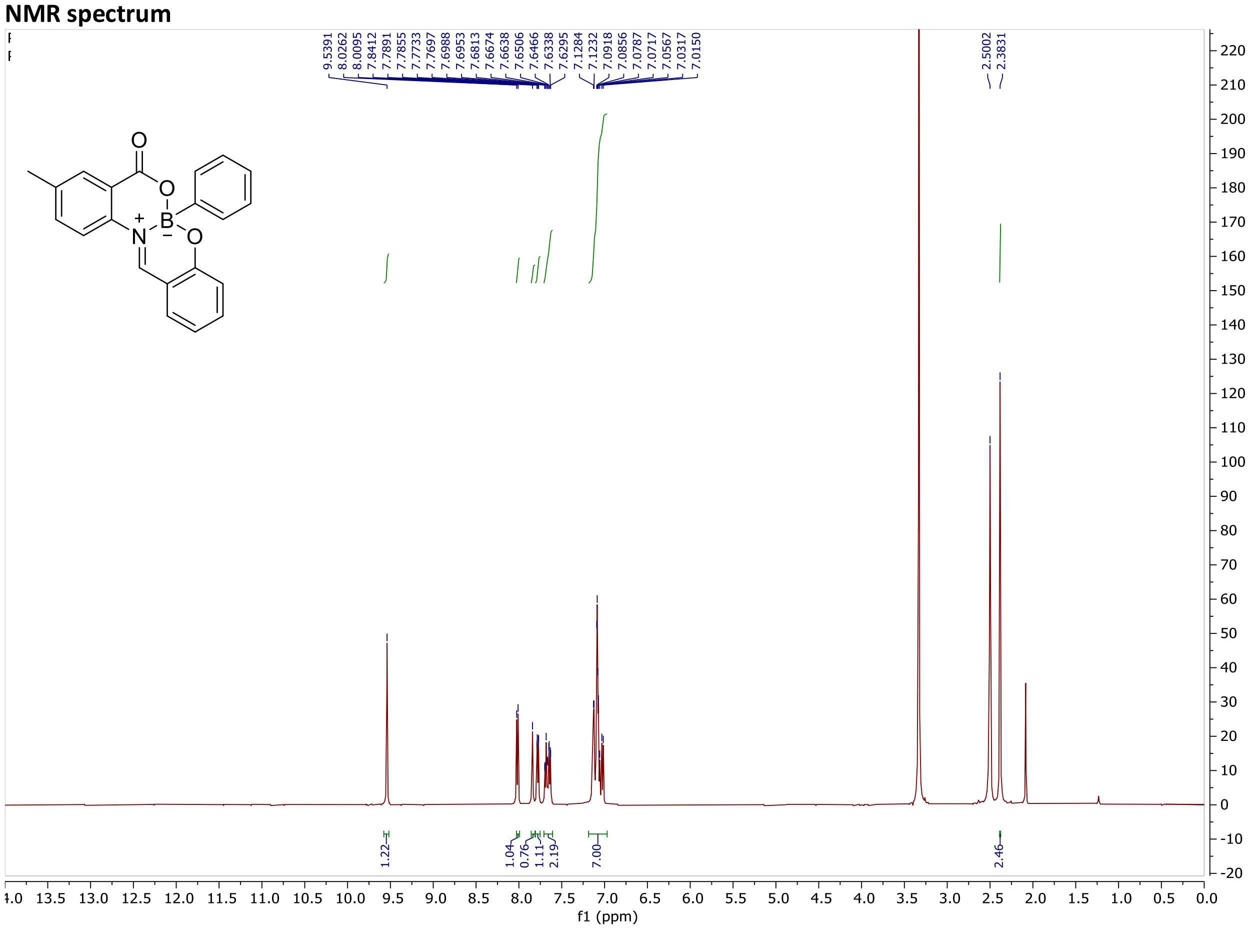

^{13}C-NMR spectrum

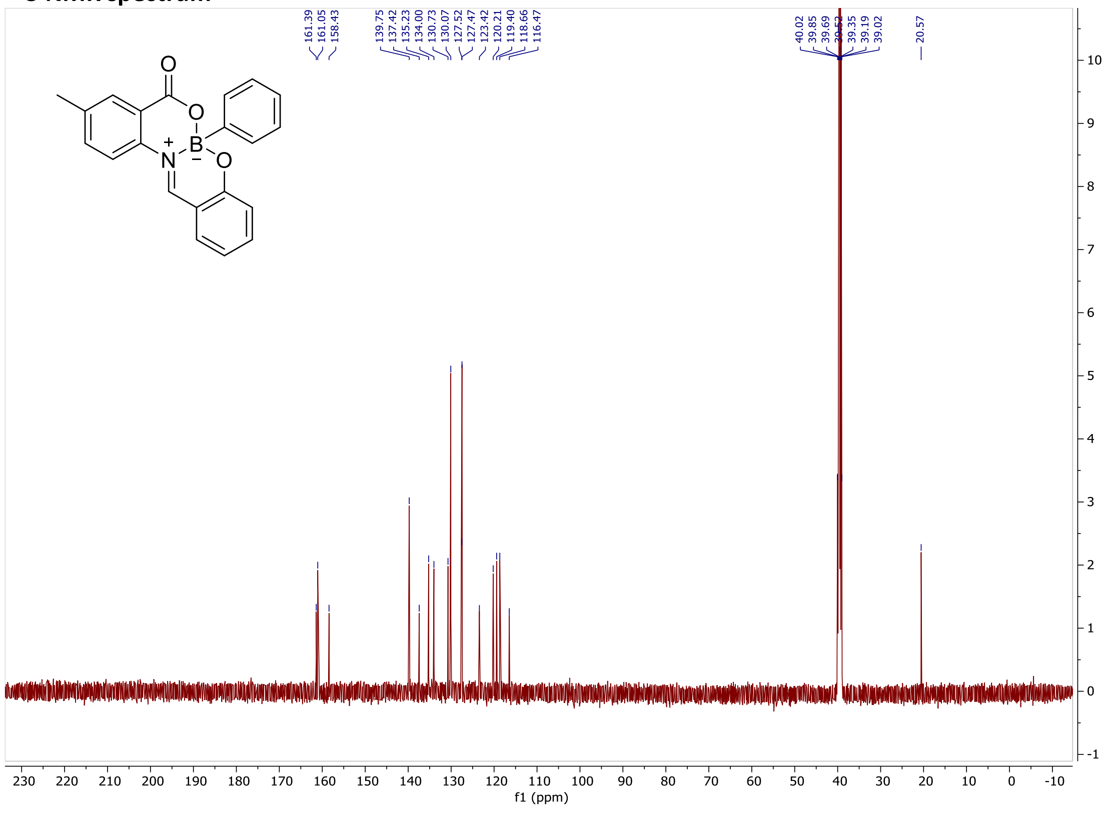

(19) HRMS

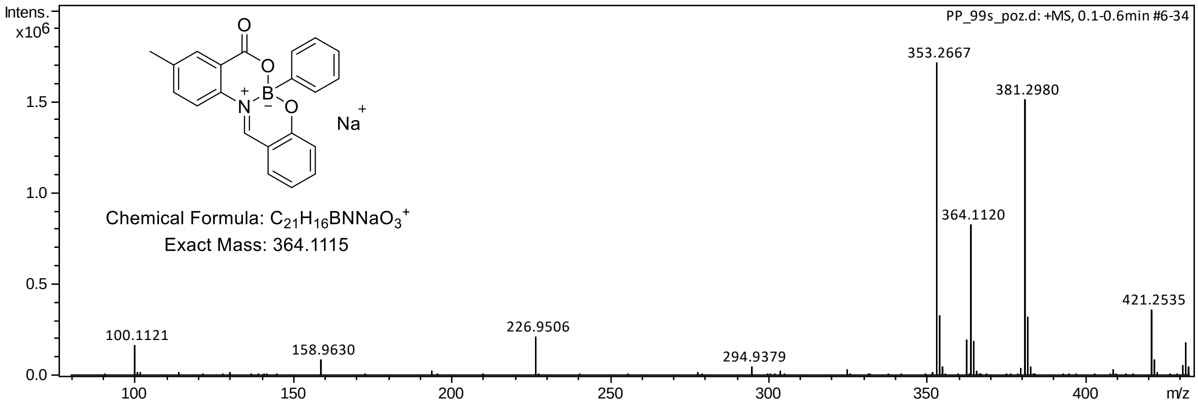
Intens.
x10^6
PP_99s_poz.d: +MS, 0.1-0.6min #6-34
Na+
Chemical Formula: C21H16BNNaO3+
Exact Mass: 364.1115
353.2667
381.2980
364.1120
421.2535
226.9506
100.1121
158.9630
294.9379
1.5
1.0
0.5
0.0
100
150
200
250
300
350
400
m/z

3-hydroxy-7-phenyl-5H,7H-7λ^4,14λ^4-benzo[d]benzo[5,6][1,3,2]oxazaborinino[2,3-b][1,3,2]oxazaborinin-5-one **(20)** **^{1}H-NMR spectrum**

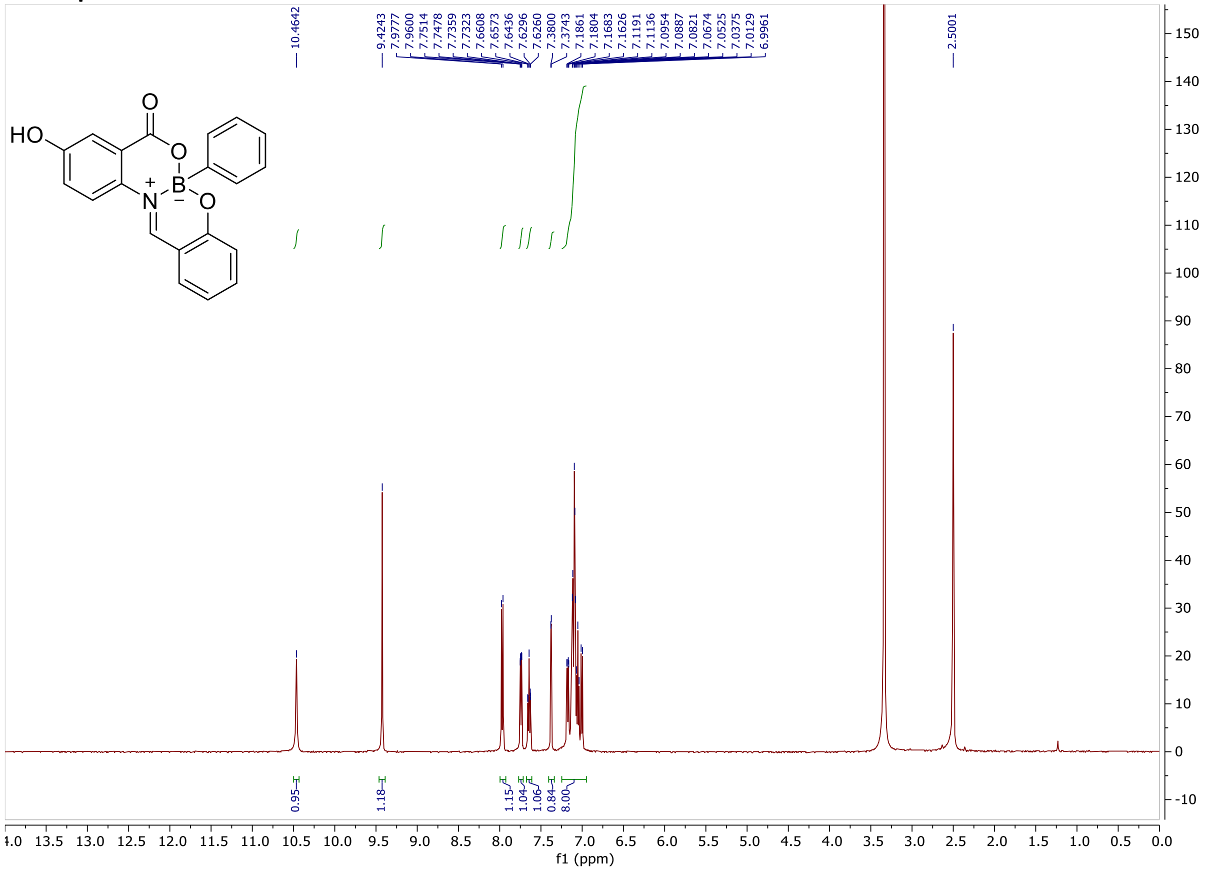

^{13}C-NMR spectrum

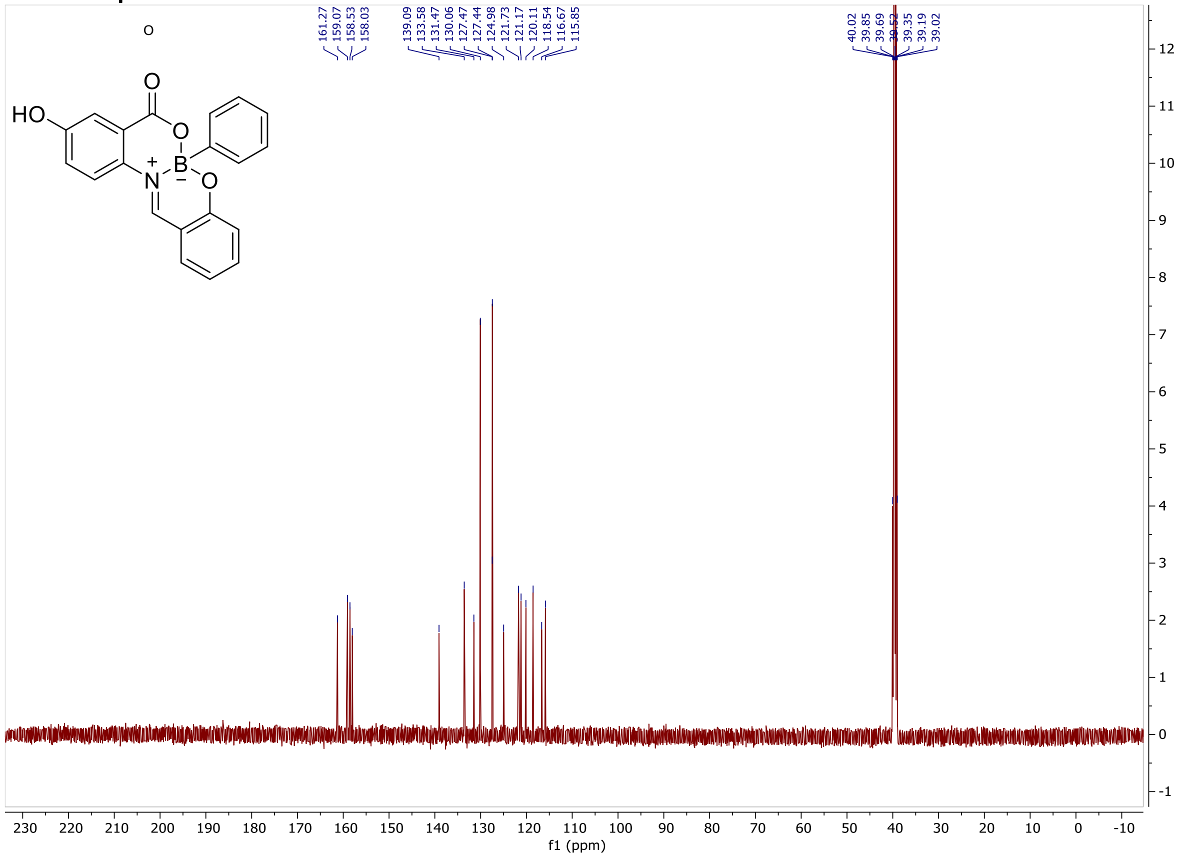

(20) HRMS

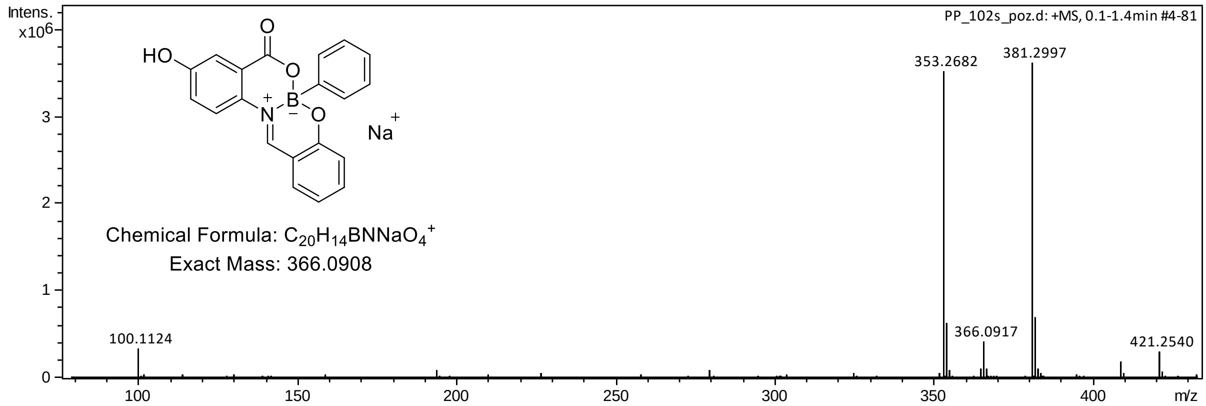
PP_102s_poz.d: +MS, 0.1-1.4min #4-81
Intens.
x10^6
353.2682
381.2997
366.0917
421.2540
100.1124
HO
O
O
B
N
O
Na+
Chemical Formula: C20H14BNNaO4+
Exact Mass: 366.0908
m/z

3-fluoro-7-phenyl-5H,7H-7λ^4,14λ^4-benzo[d]benzo[5,6][1,3,2]oxazaborinino[2,3-b][1,3,2]oxazaborinin-5-one **(21)** **^{1}H-NMR spectrum**

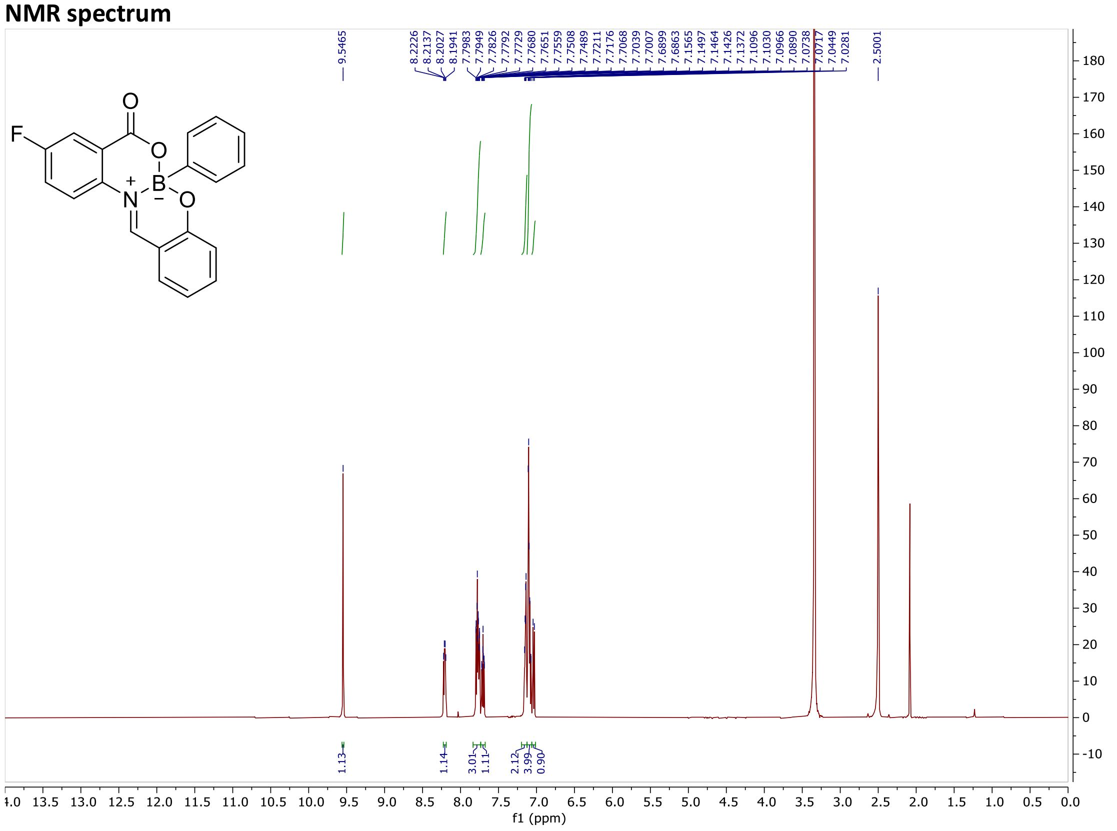

^{13}C-NMR spectrum

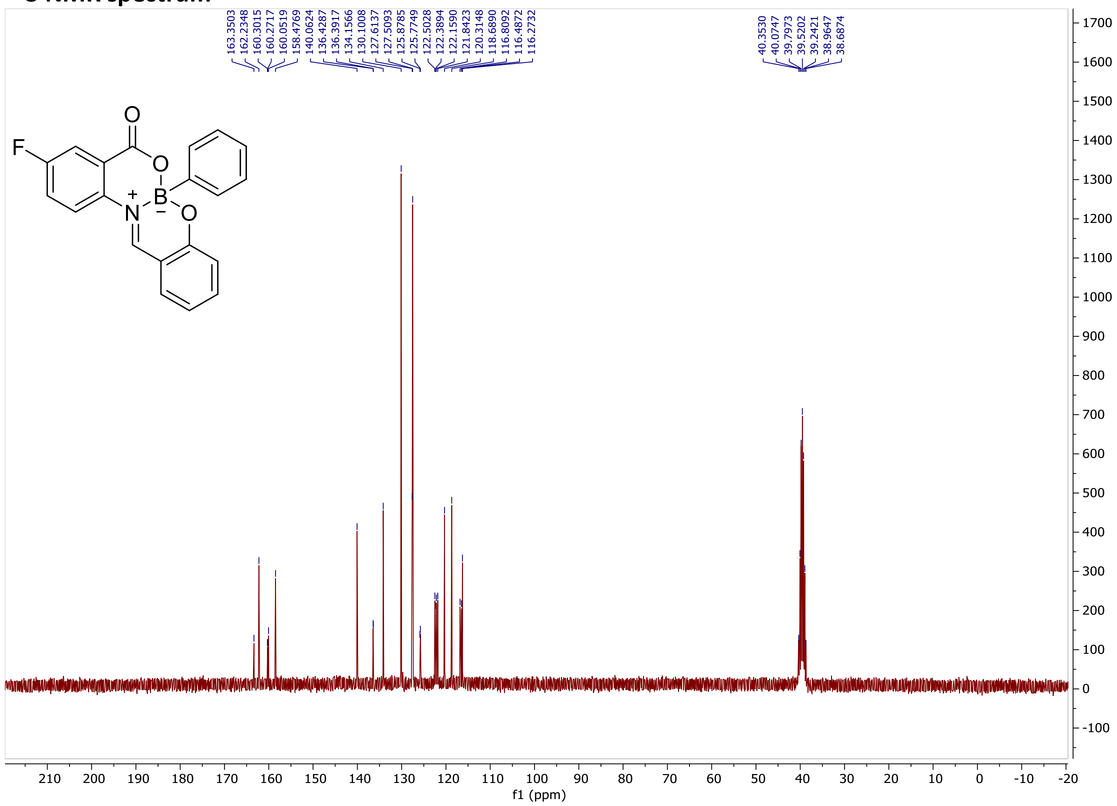

(21) ^{1}H-NMR spectrum Bottom: crude from method C**; Top:** recrystallized from PS and Ethyl acetate

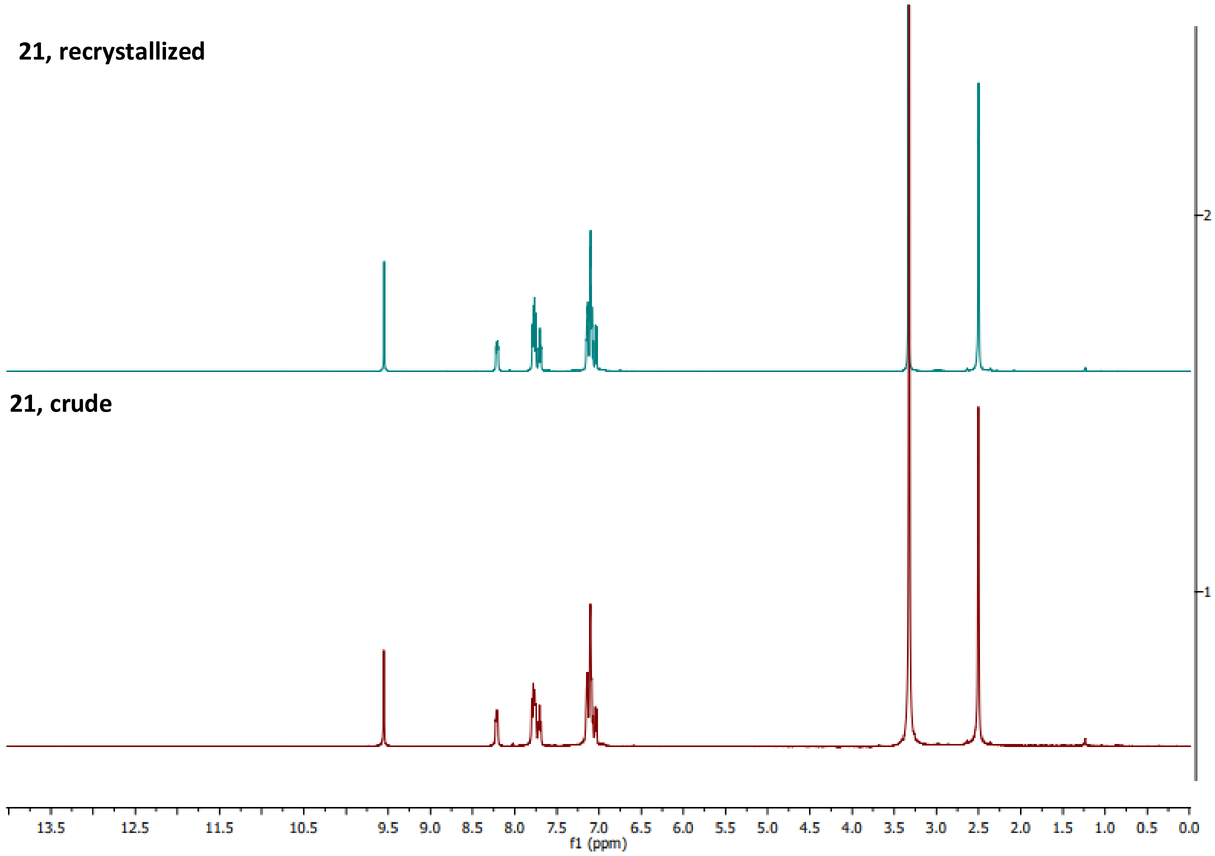

(21) HRMS

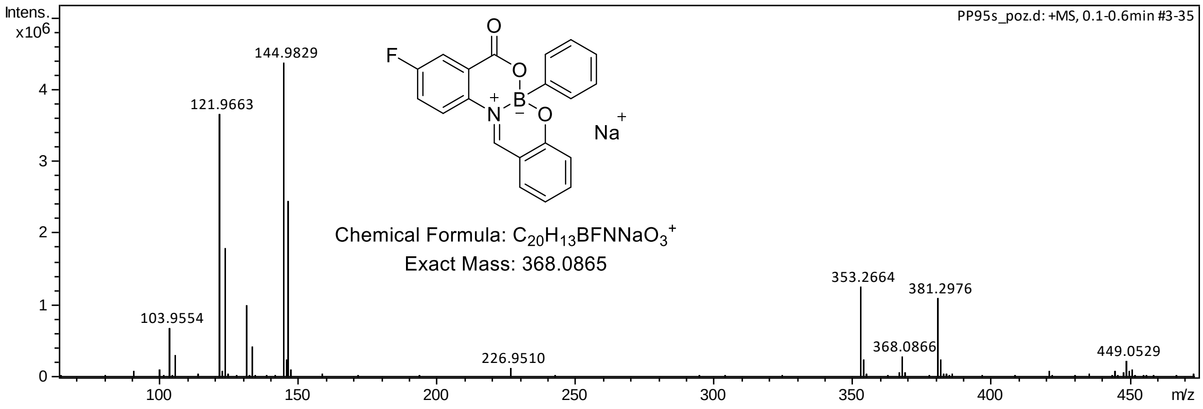

3-chloro-7-phenyl-5H,7H-7λ^4,14λ^4-benzo[d]benzo[5,6][1,3,2]oxazaborinino[2,3-b][1,3,2]oxazaborinin-5-one **(22)** **^{1}H-NMR spectrum**

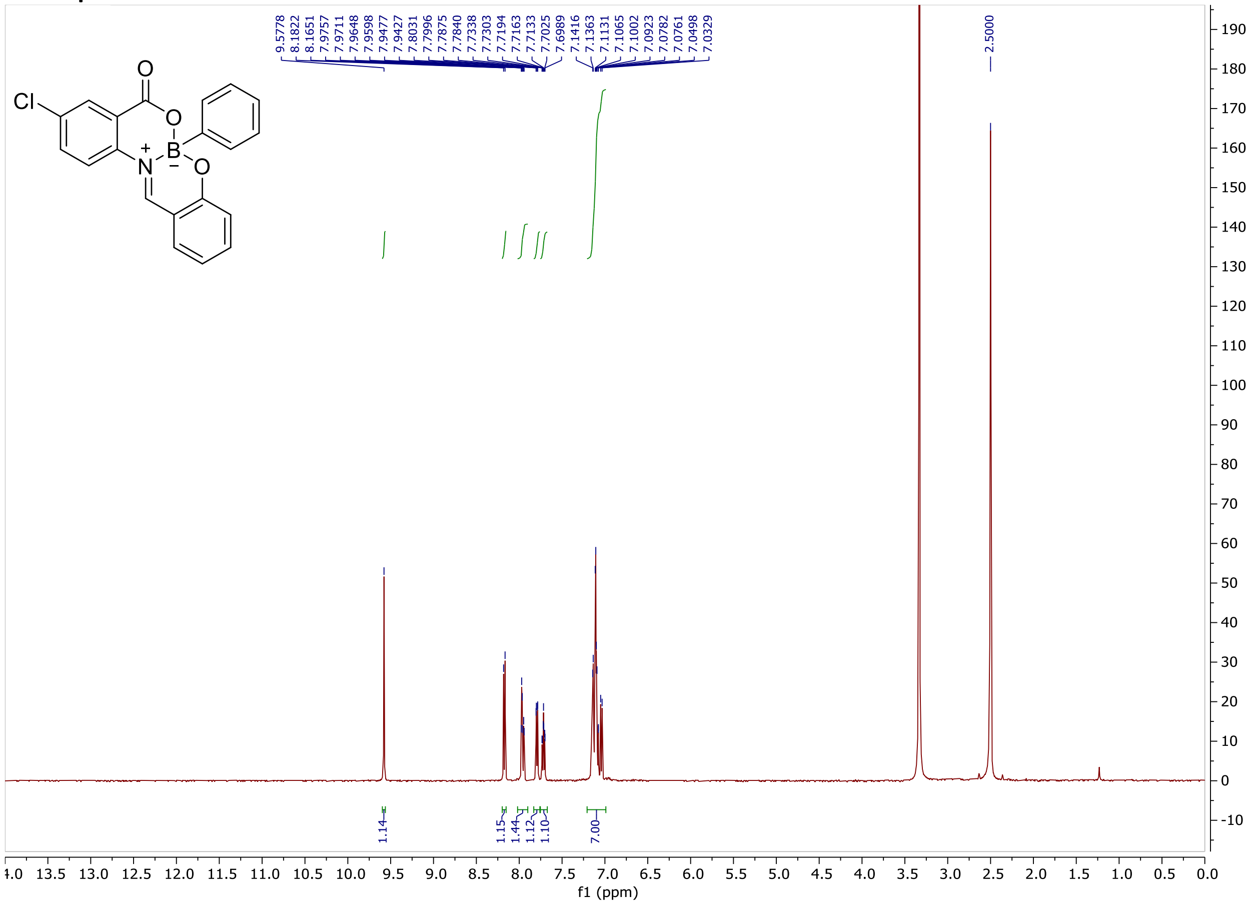

^{13}C-NMR spectrum

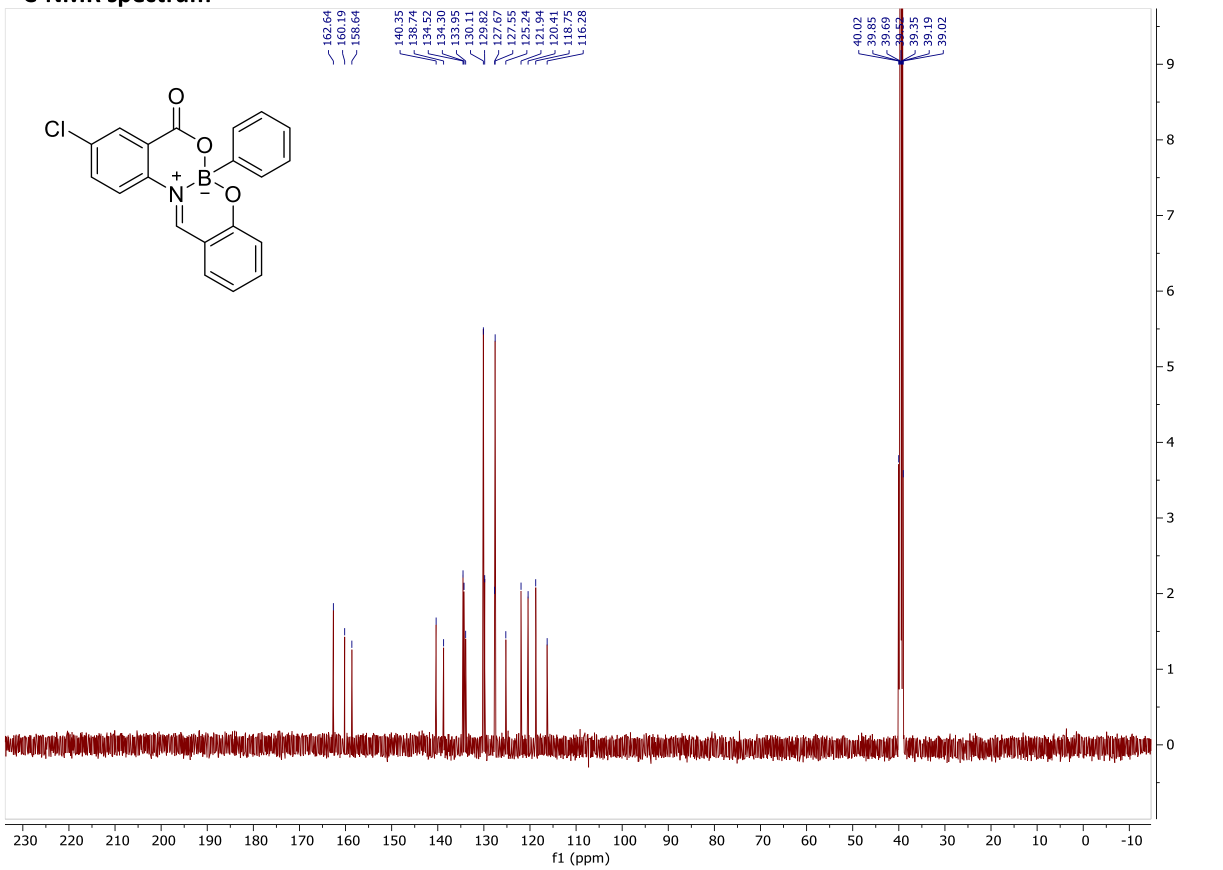

(22) ^{1}**H-NMR spectrum Bottom:** crude from method C**; Top:** recrystallized from PS and Ethyl acetate

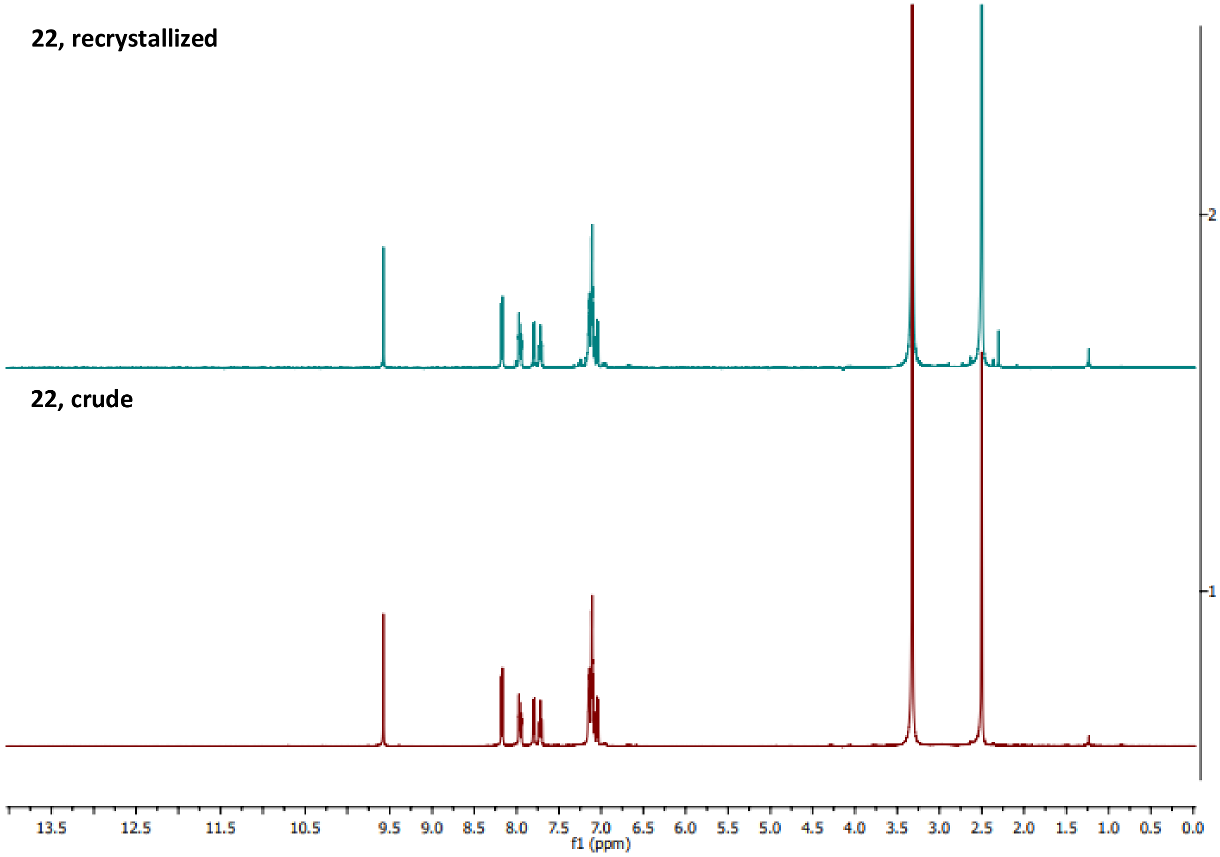

(22) HRMS

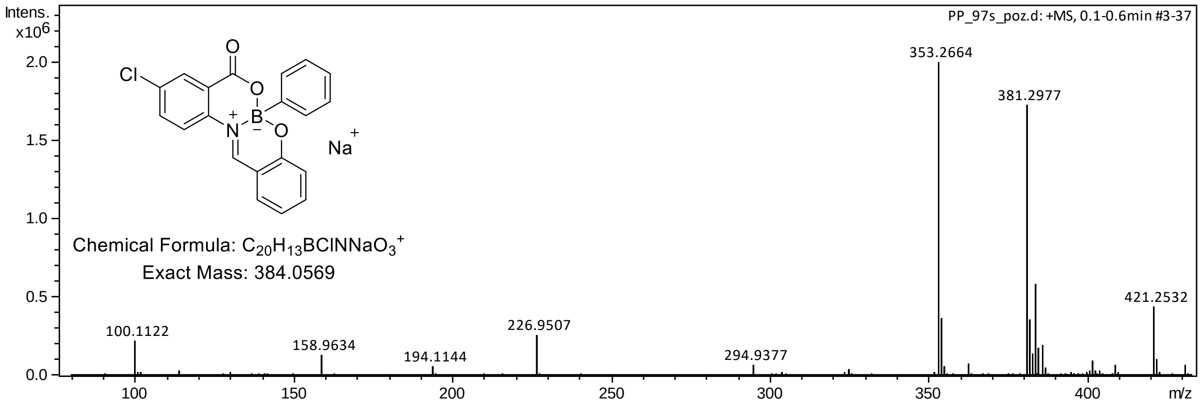

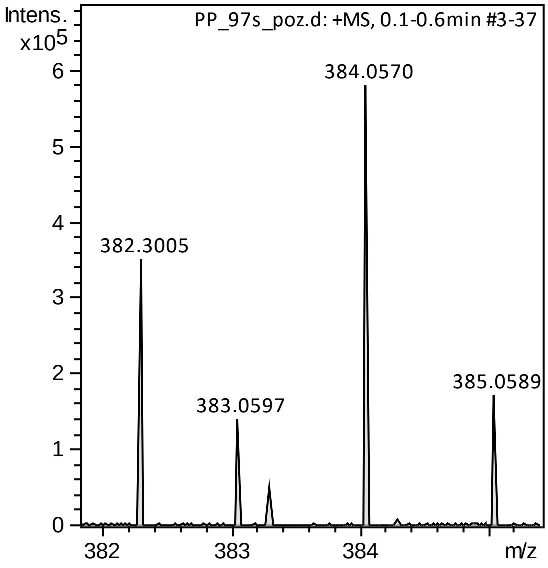
Intens.
x10^5
PP_97s_poz.d: +MS, 0.1-0.6min #3-37
384.0570
382.3005
385.0589
383.0597
6
5
4
3
2
1
0
382
383
384
m/z

3-bromo-7-phenyl-5H,7H-7λ^4,14λ^4-benzo[d]benzo[5,6][1,3,2]oxazaborinino[2,3-b][1,3,2]oxazaborinin-5-one **(23)** **^{1}H-NMR spectrum**

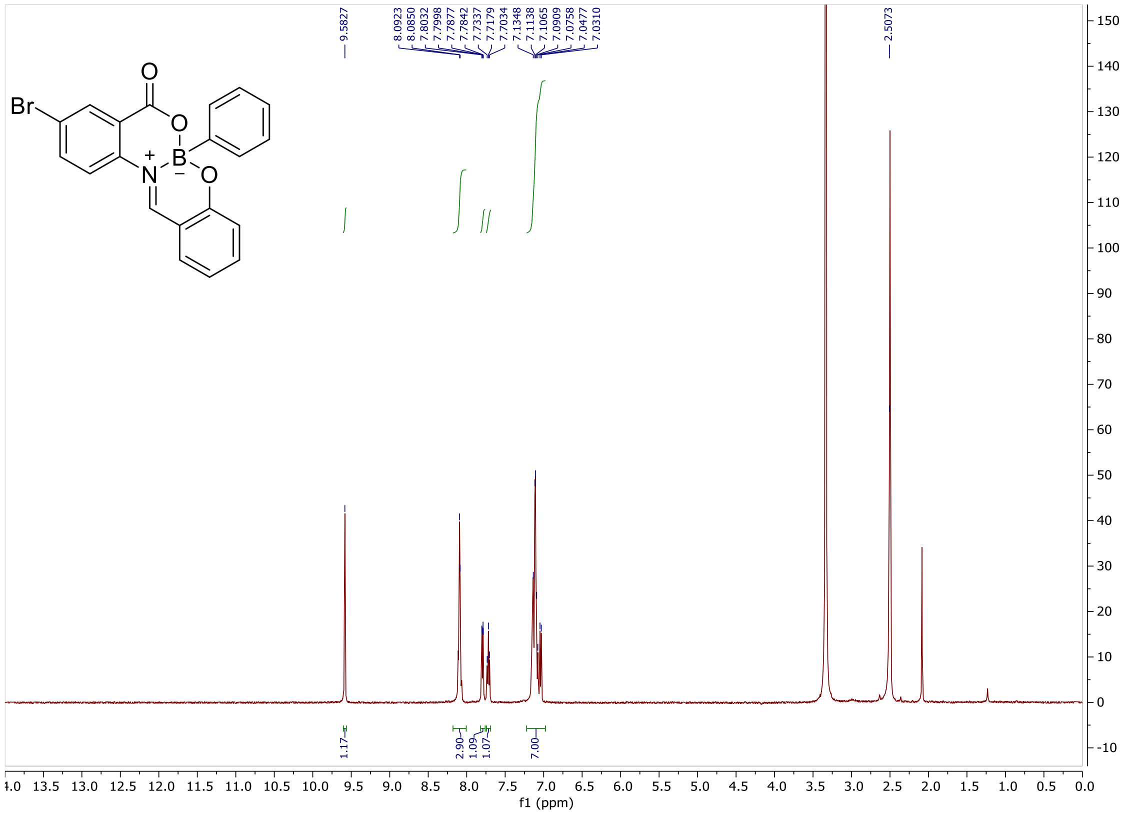

^{13}C-NMR spectrum

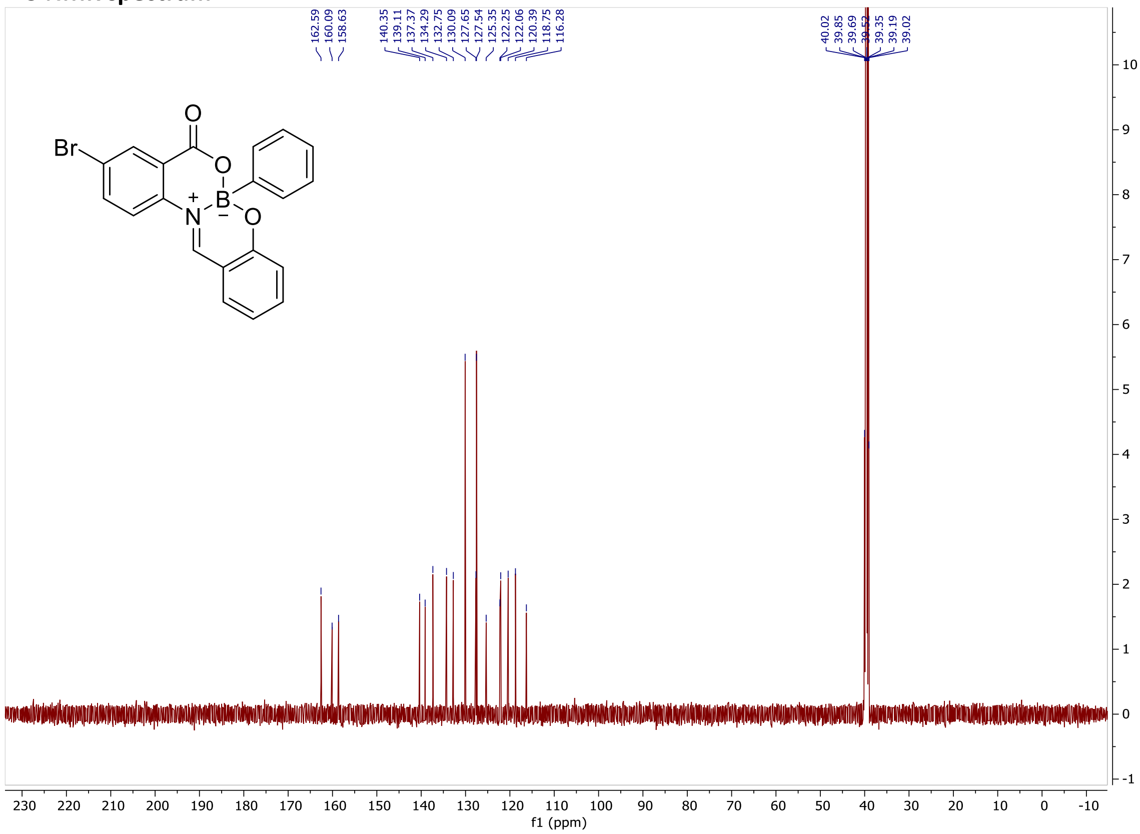

(23) ^{1}**H-NMR spectrum Bottom:** crude from method C**; Top:** recrystallized from PS and Ethyl acetate

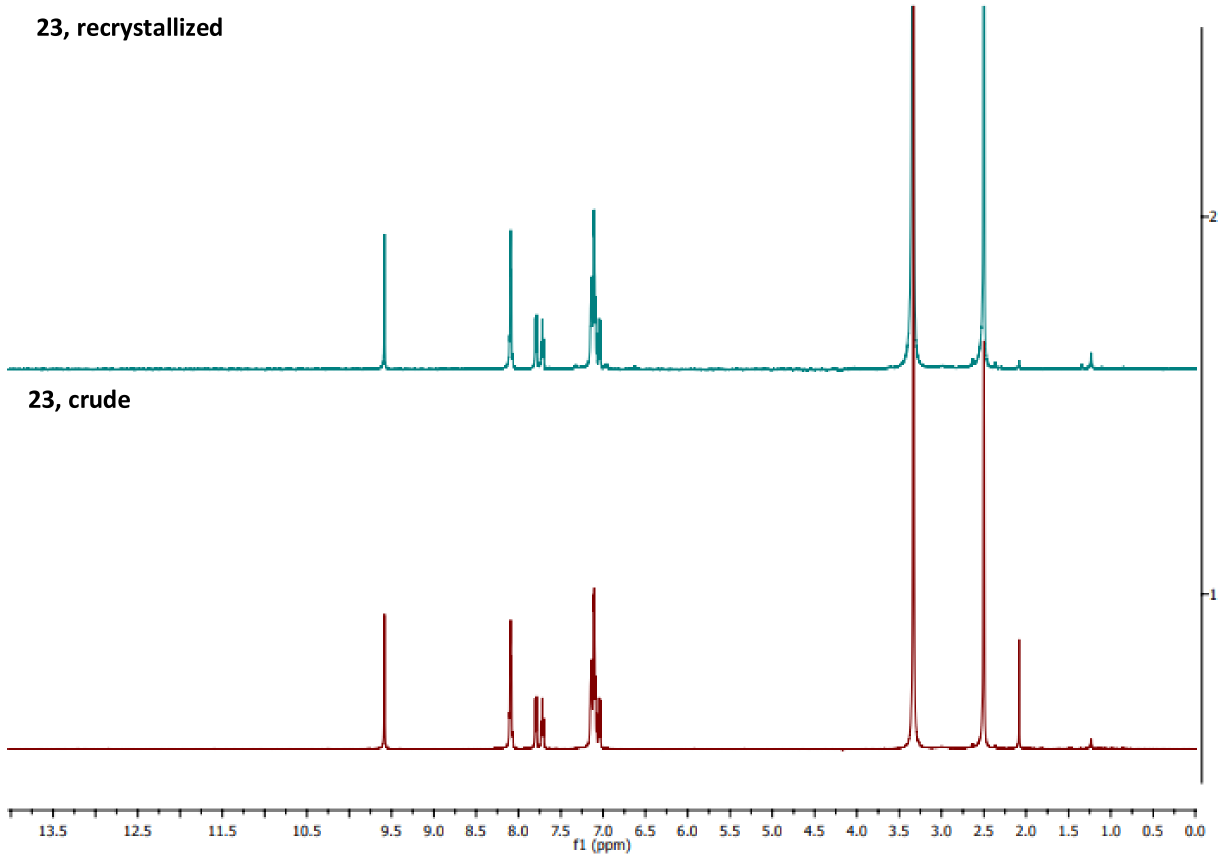

(23) HRMS

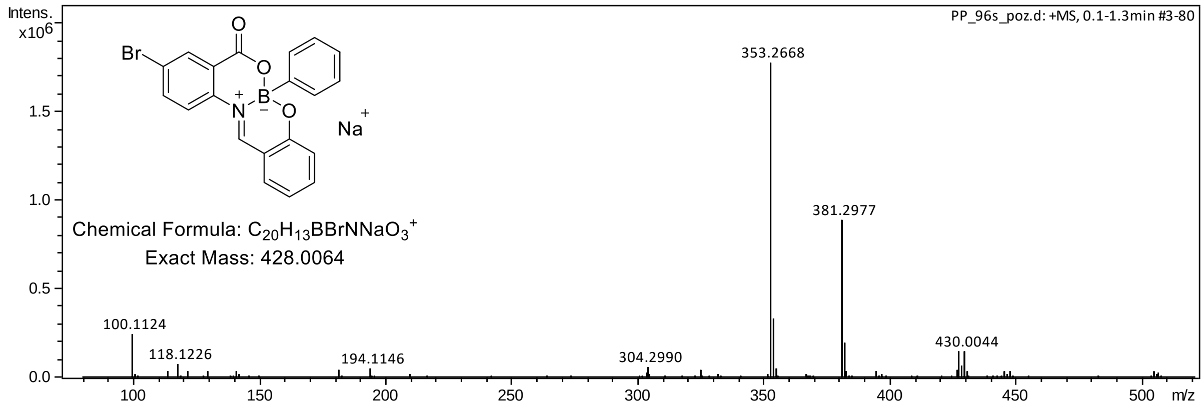

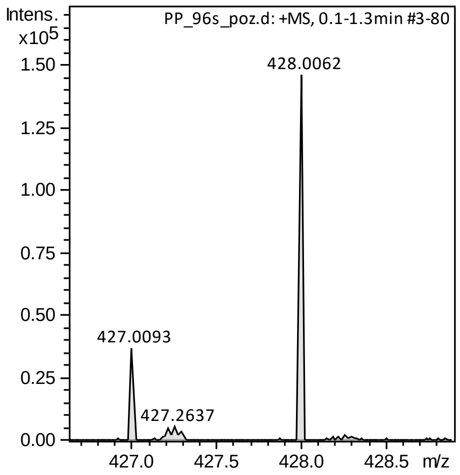

3-iodo-7-phenyl-5H,7H-7λ[4],14λ[4]-benzo[d]benzo[5,6][1,3,2]oxazaborinino[2,3-b][1,3,2]oxazaborinin-5-one **(24)** **^{1}H-NMR spectrum**

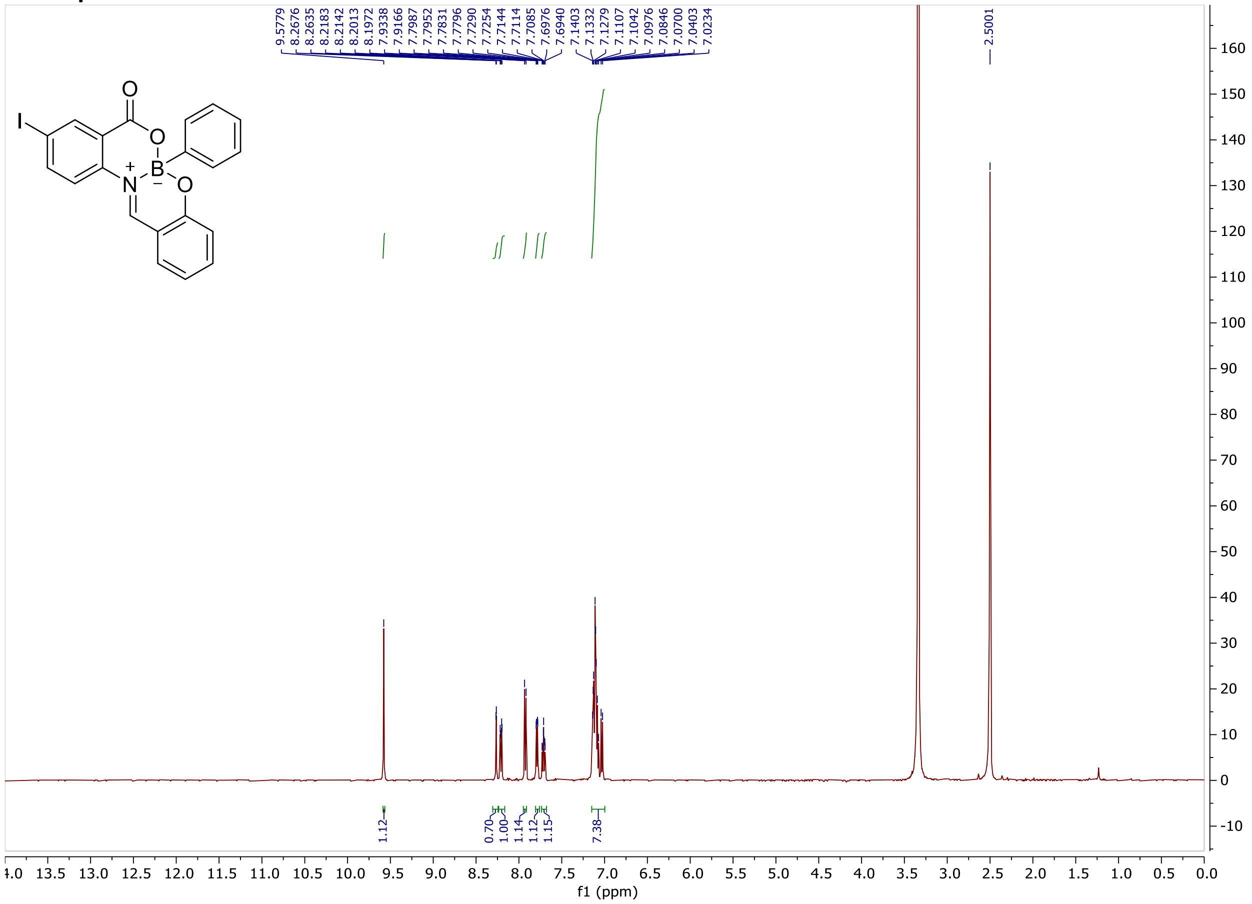

^{13}C-NMR spectrum

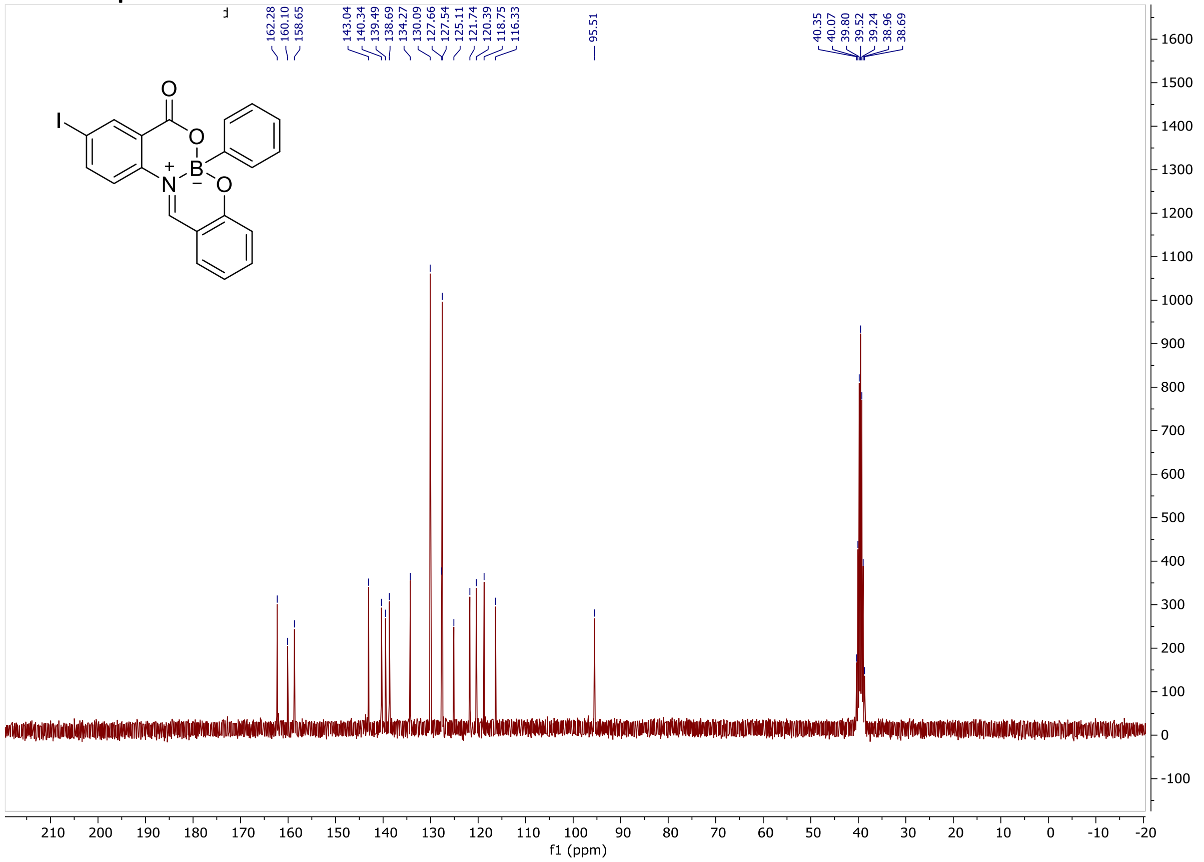

(24) HRMS

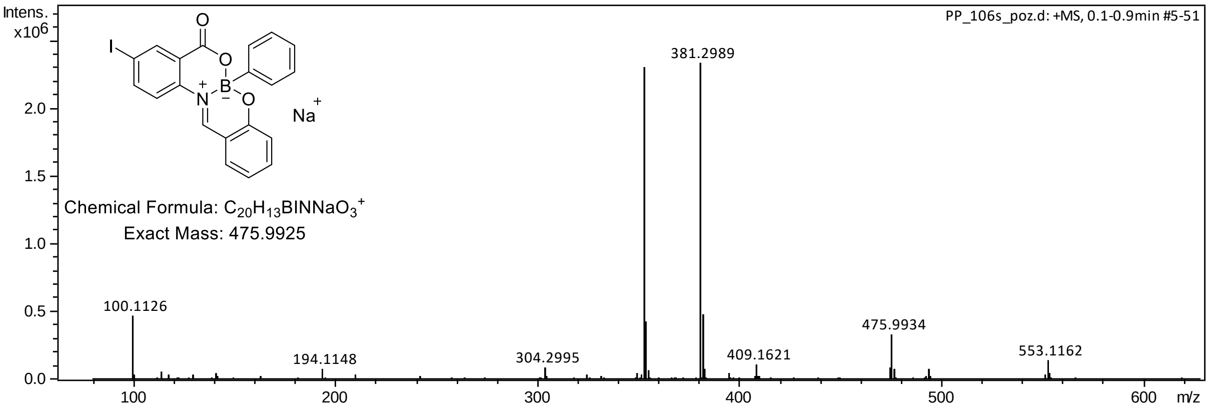

Intens.
x10^6
2.0
1.5
1.0
0.5
0.0
PP_106s_poz.d: +MS, 0.1-0.9min #5-51
I
O
O
B
N
O
Na+
Chemical Formula: C20H13BINNaO3+
Exact Mass: 475.9925
381.2989
100.1126
194.1148
304.2995
409.1621
475.9934
553.1162
100
200
300
400
500
600
m/z

3-nitro-7-phenyl-5H,7H-7 λ[4],14 λ[4]-benzo[d]benzo[5,6][1,3,2]oxazaborinino[2,3-b][1,3,2]oxazaborinin-5-one **(25)** **^{1}H-NMR spectrum**

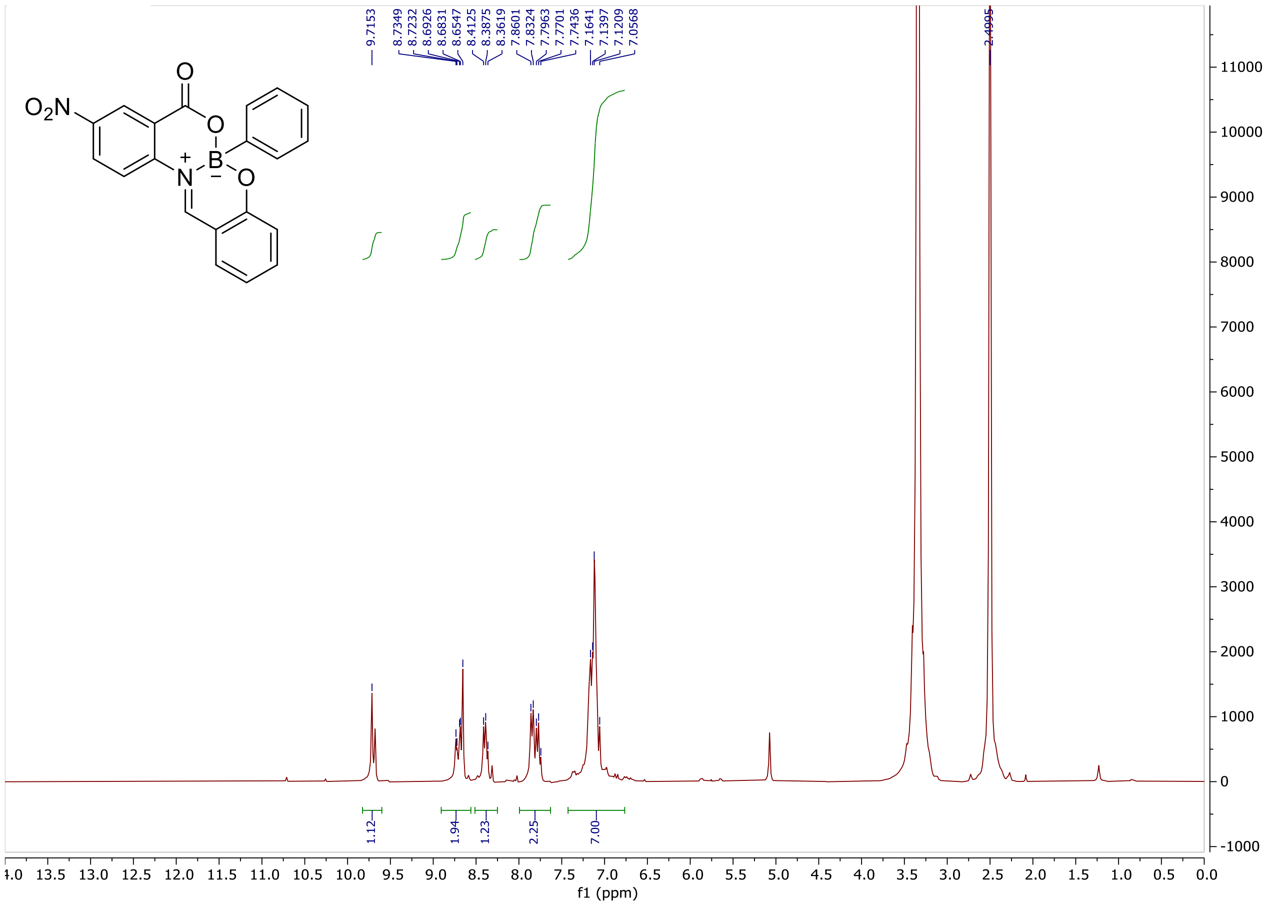

2-chloro-7-phenyl-5H,7H-7λ[4],14λ[4]-benzo[d]benzo[5,6][1,3,2]oxazaborinino[2,3-b][1,3,2]oxazaborinin-5-one **(26)** **^{1}H-NMR spectrum**

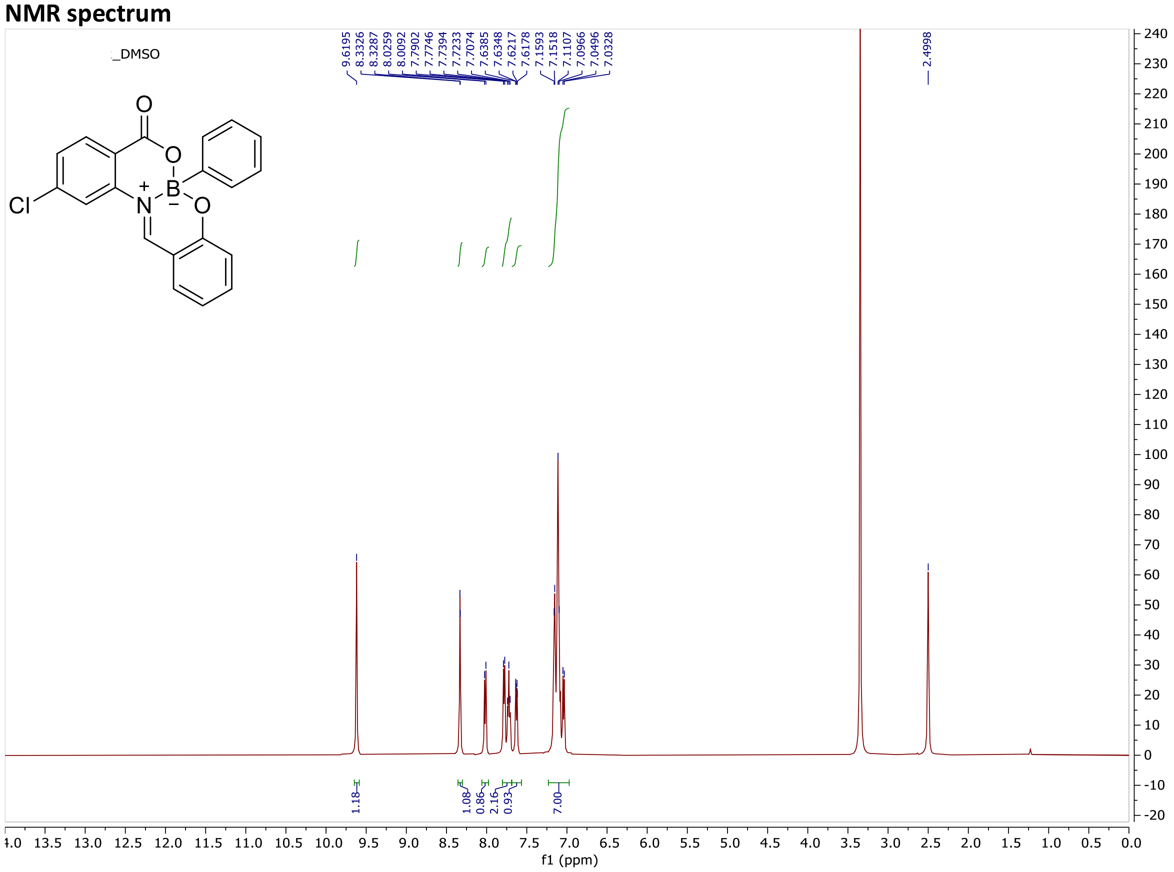

^{13}C-NMR spectrum

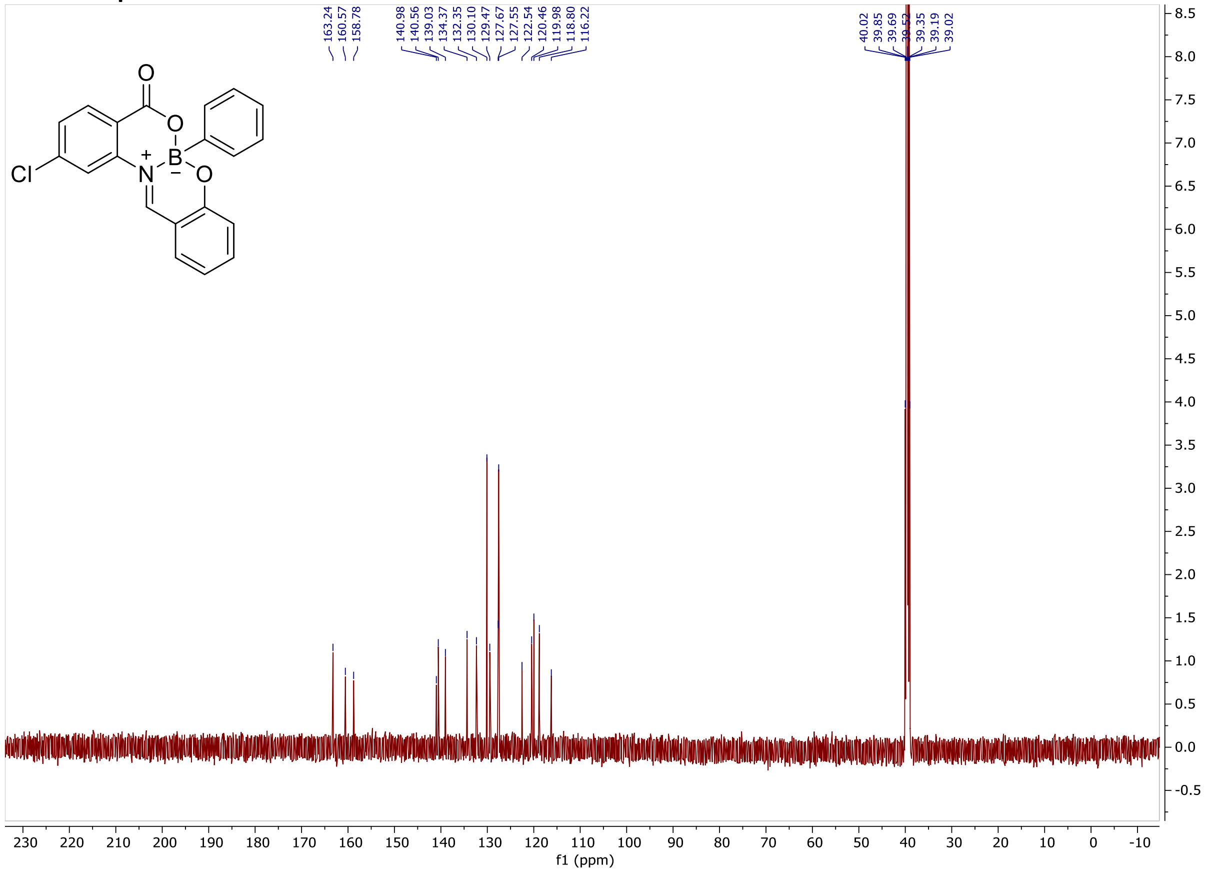

(26) HRMS

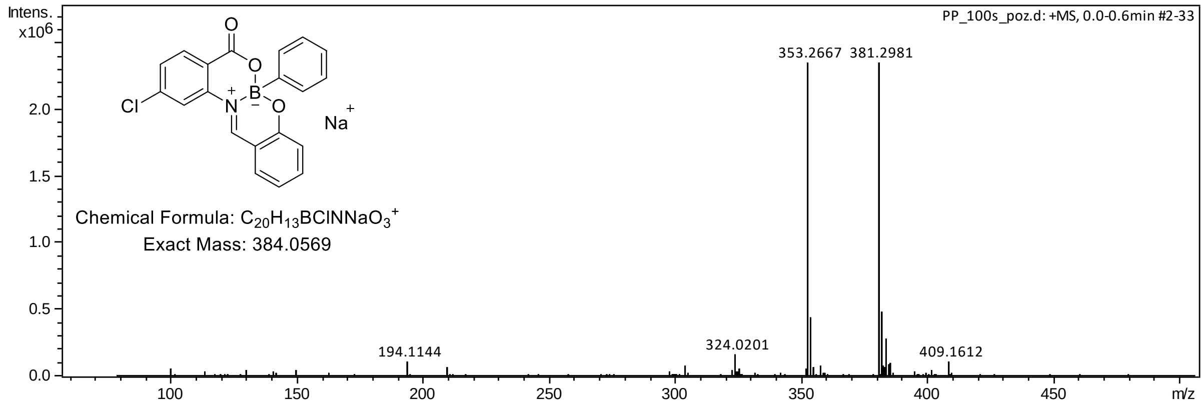
Intens.
x10^6
PP_100s_poz.d: +MS, 0.0-0.6min #2-33
Chemical Formula: $C_{20}H_{13}BClNNaO_3^+$
Exact Mass: 384.0569
Cl
Na+
353.2667
381.2981
194.1144
324.0201
409.1612
m/z

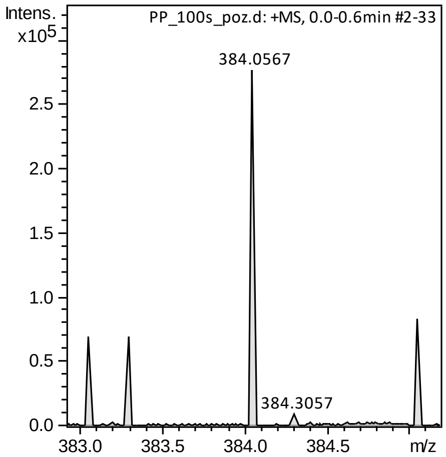
Intens.
x10^5
PP_100s_poz.d: +MS, 0.0-0.6min #2-33
384.0567
384.3057
m/z

2-nitro-7-phenyl-5H,7H-7λ[4],14λ[4]-benzo[d]benzo[5,6][1,3,2]oxazaborinino[2,3-b][1,3,2]oxazaborinin-5-one **(27)** **^{1}H-NMR spectrum**

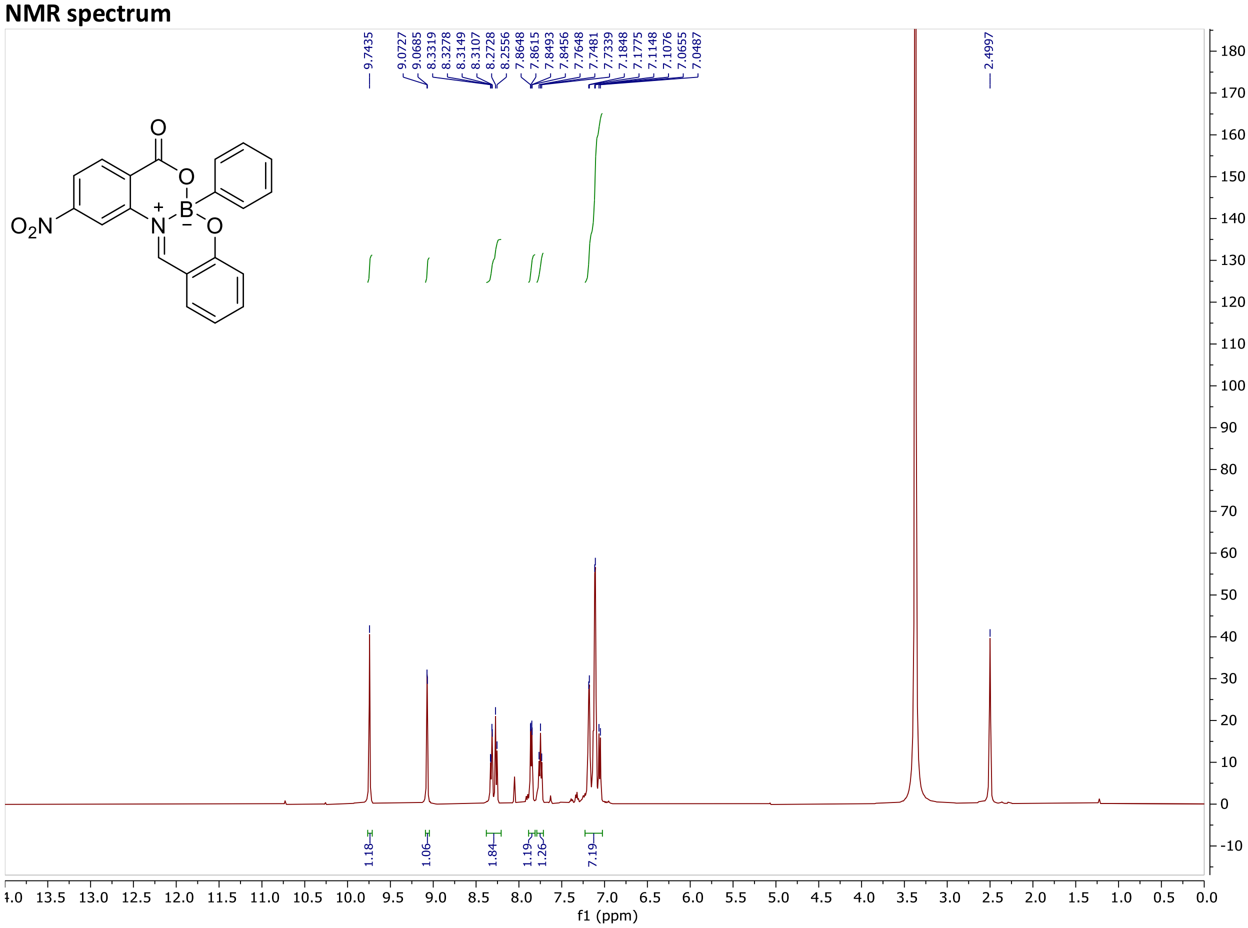

^{13}C-NMR spectrum

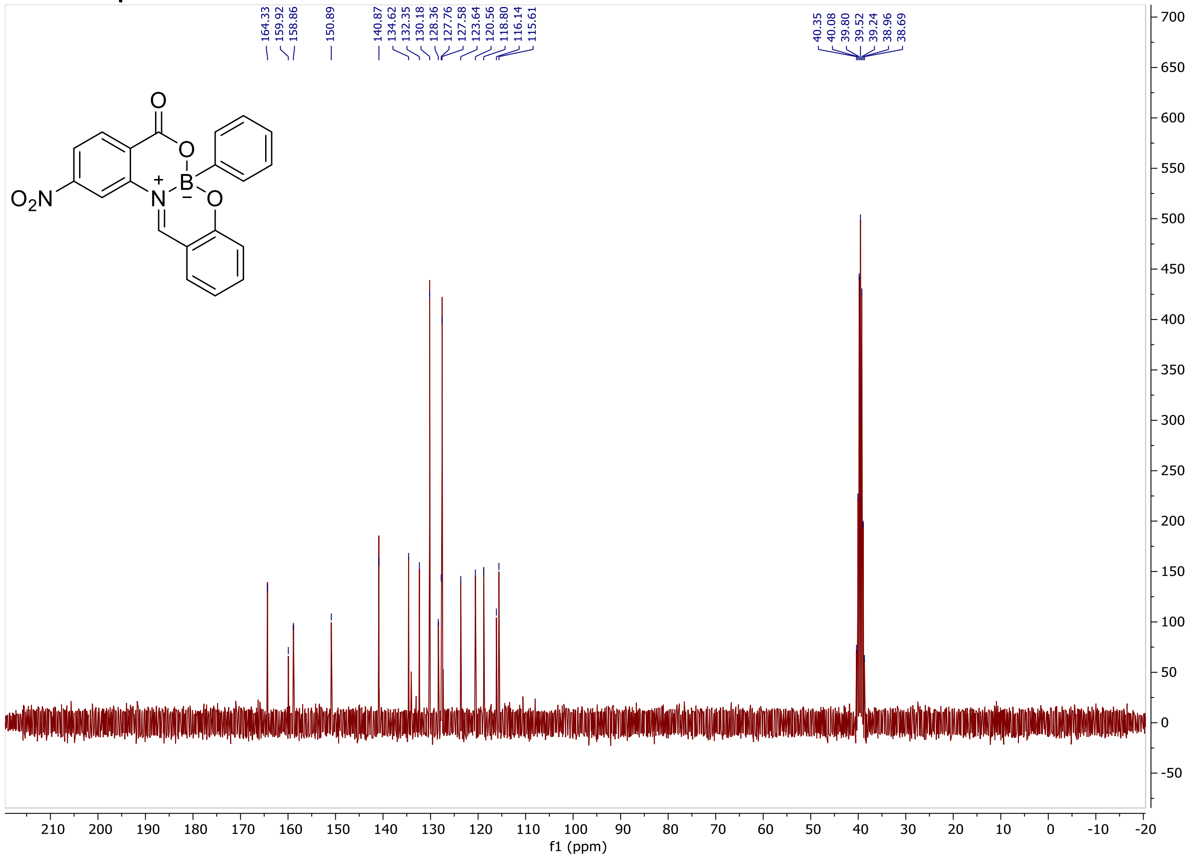

(27) HRMS

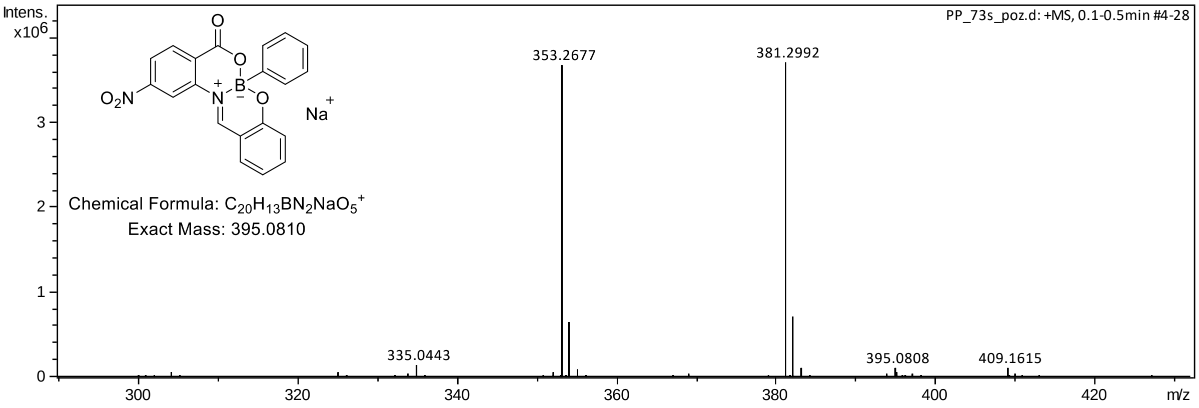

Intens.
x10^6
PP_73s_poz.d: +MS, 0.1-0.5min #4-28
O
O
B
O
N
O2N
Na+
Chemical Formula: C20H13BN2NaO5+
Exact Mass: 395.0810
353.2677
381.2992
335.0443
395.0808
409.1615
3
2
1
0
300
320
340
360
380
400
420
m/z

13-methyl-7-phenyl-5H,7H-7λ⁴,14λ⁴-benzo[d]benzo[5,6][1,3,2]oxazaborinino[2,3-b][1,3,2]oxazaborinin-5-one **(28)** **^{1}H-NMR spectrum**

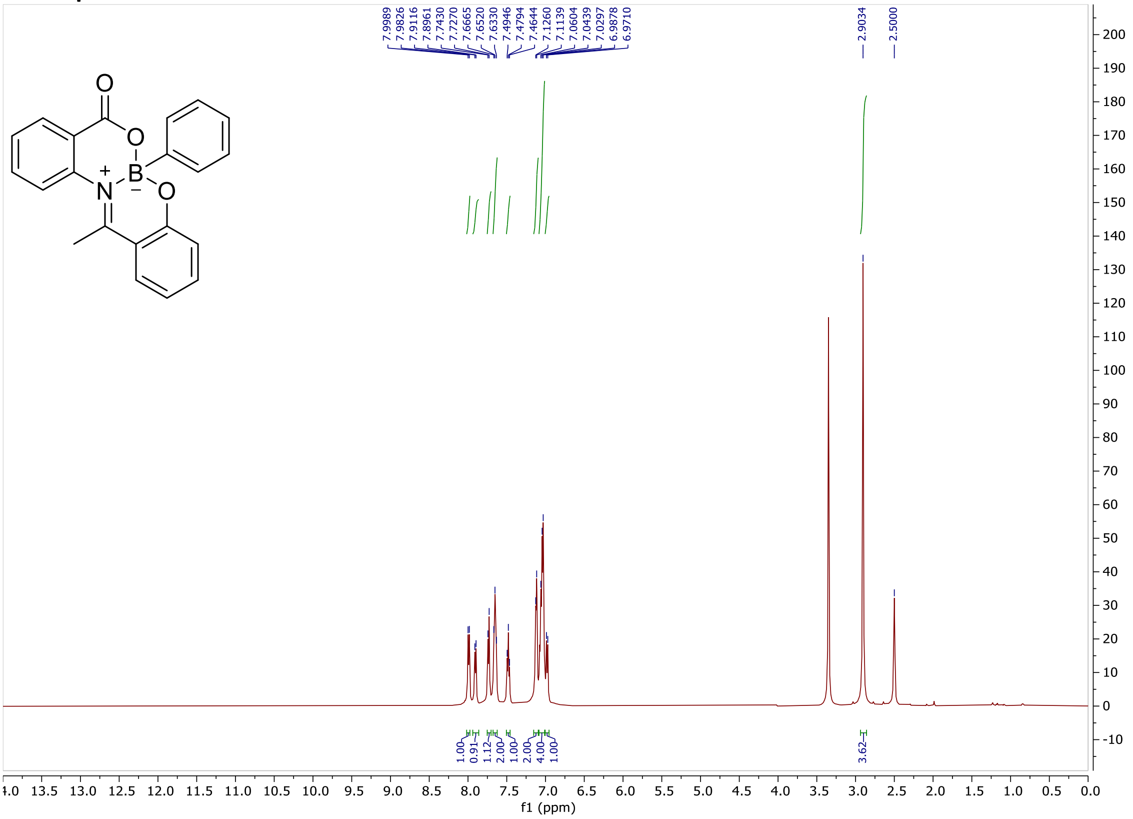

^{13}C-NMR spectrum

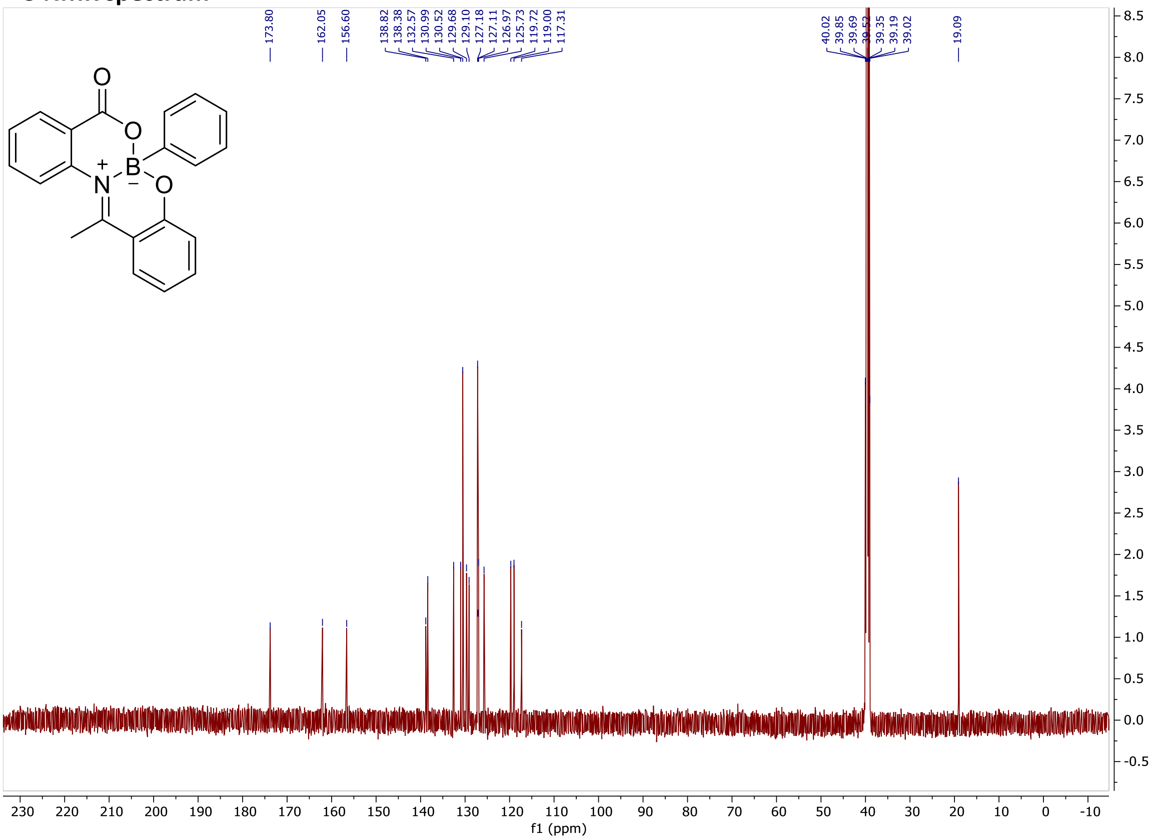

(28) HRMS

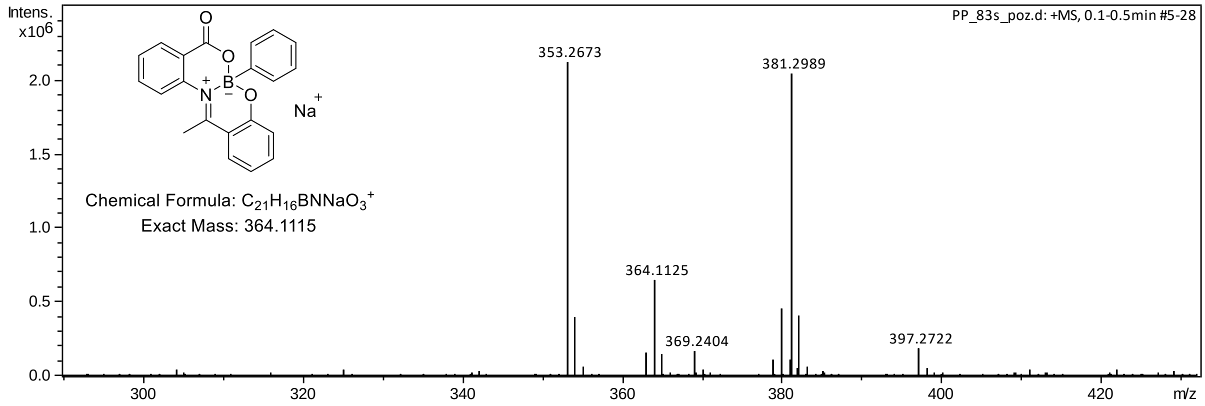

PP_83s_poz.d: +MS, 0.1-0.5min #5-28
Intens.
x10^6
Na+
Chemical Formula: C21H16BNNaO3+
Exact Mass: 364.1115
353.2673
381.2989
364.1125
369.2404
397.2722
m/z

3,13-dimethyl-7-phenyl-5H,7H-7λ[4],14λ[4]-benzo[d]benzo[5,6][1,3,2]oxazaborinino [2,3-b][1,3,2]oxazaborinin-5-one **(29)**

^{1}H-NMR spectrum

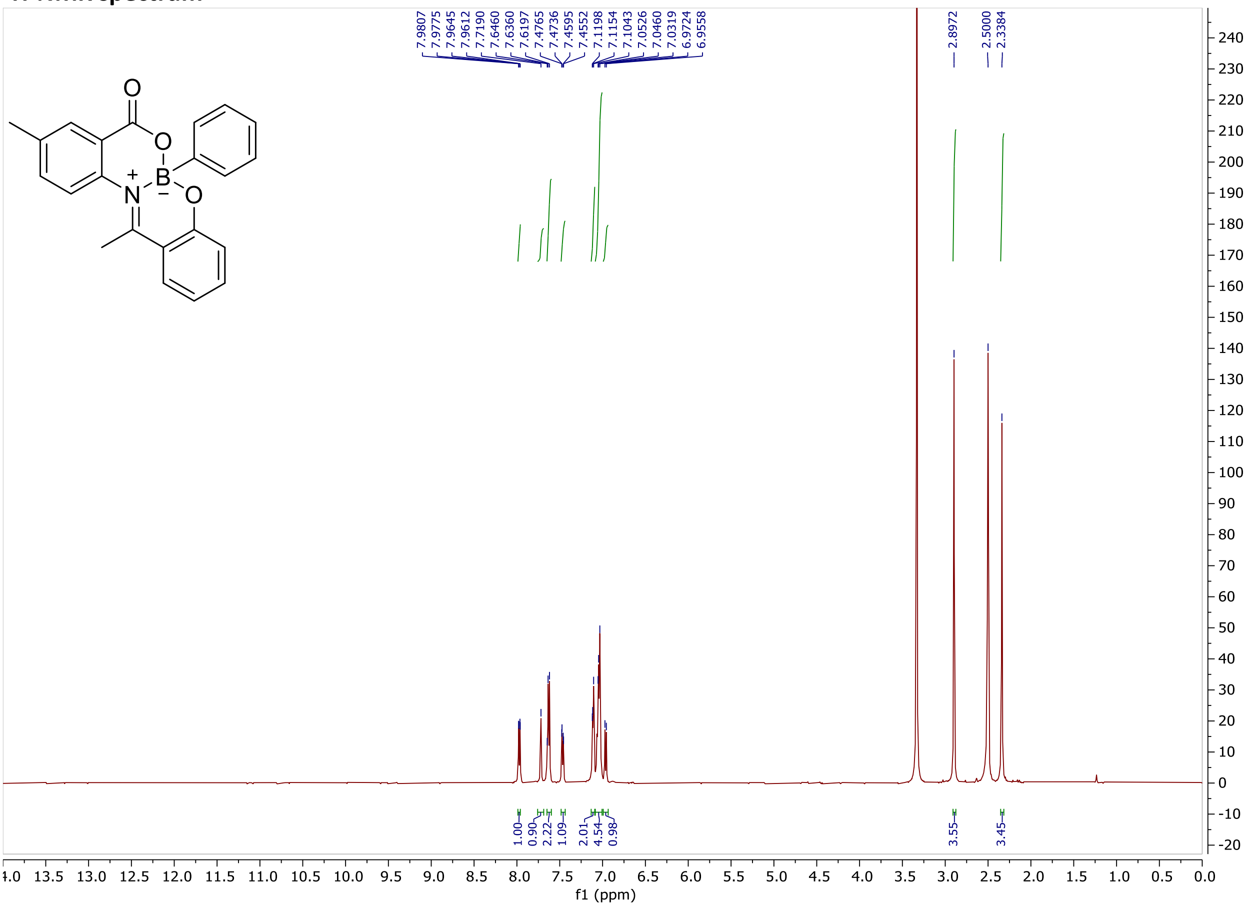

^{13}C-NMR spectrum

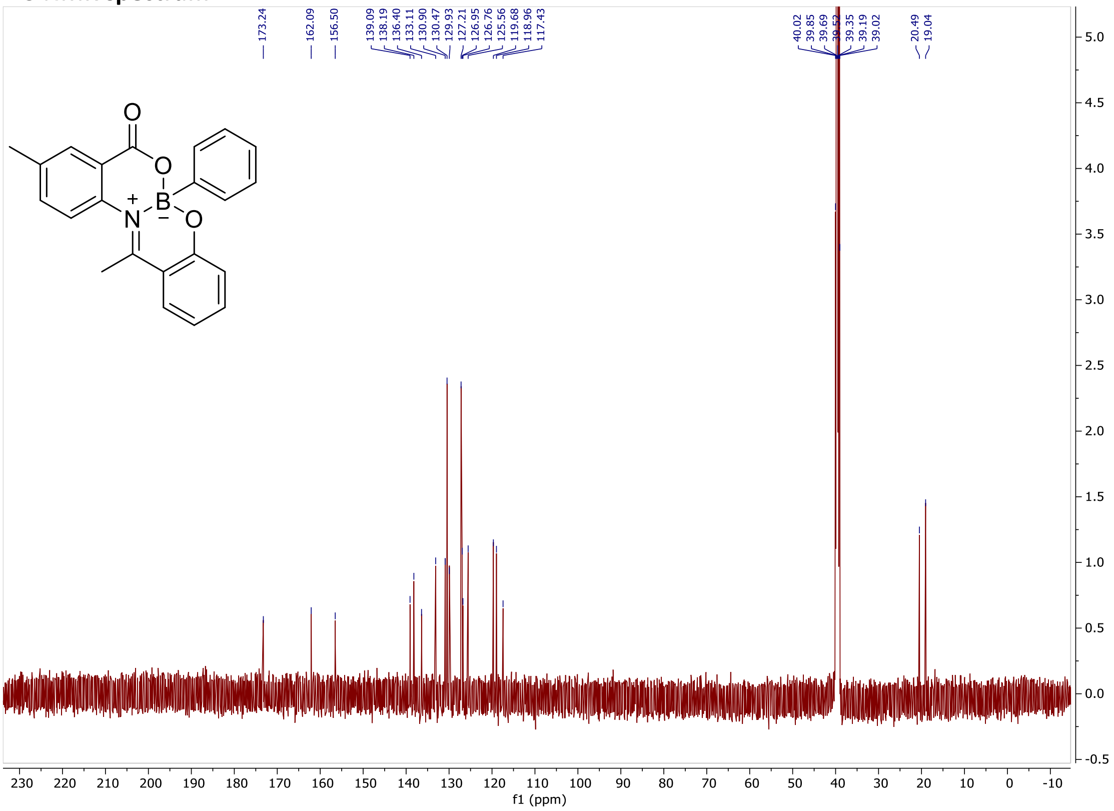

(29) HRMS

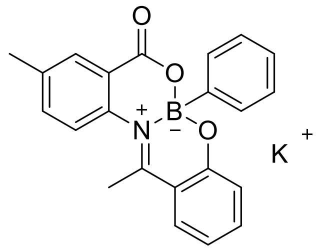

Chemical Formula: $C_{22}H_{18}BKNO_3^+$

Exact Mass: 394,1011

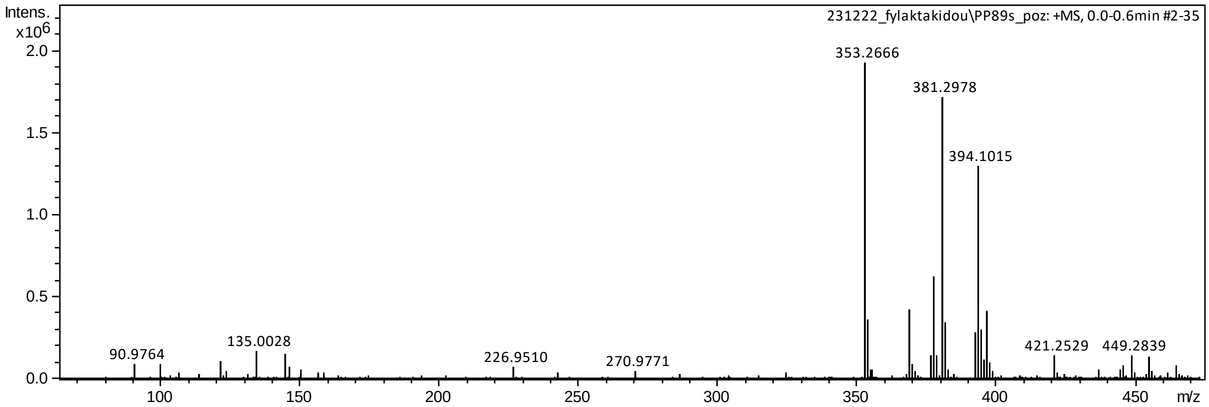

3-fluoro-13-methyl-7-phenyl-5H,7H-7λ[4],14λ[4]-benzo[d]benzo[5,6][1,3,2]oxazabo-rinino[2,3-b][1,3,2]oxazaborinin-5-one **(30)** **^{1}H-NMR spectrum**

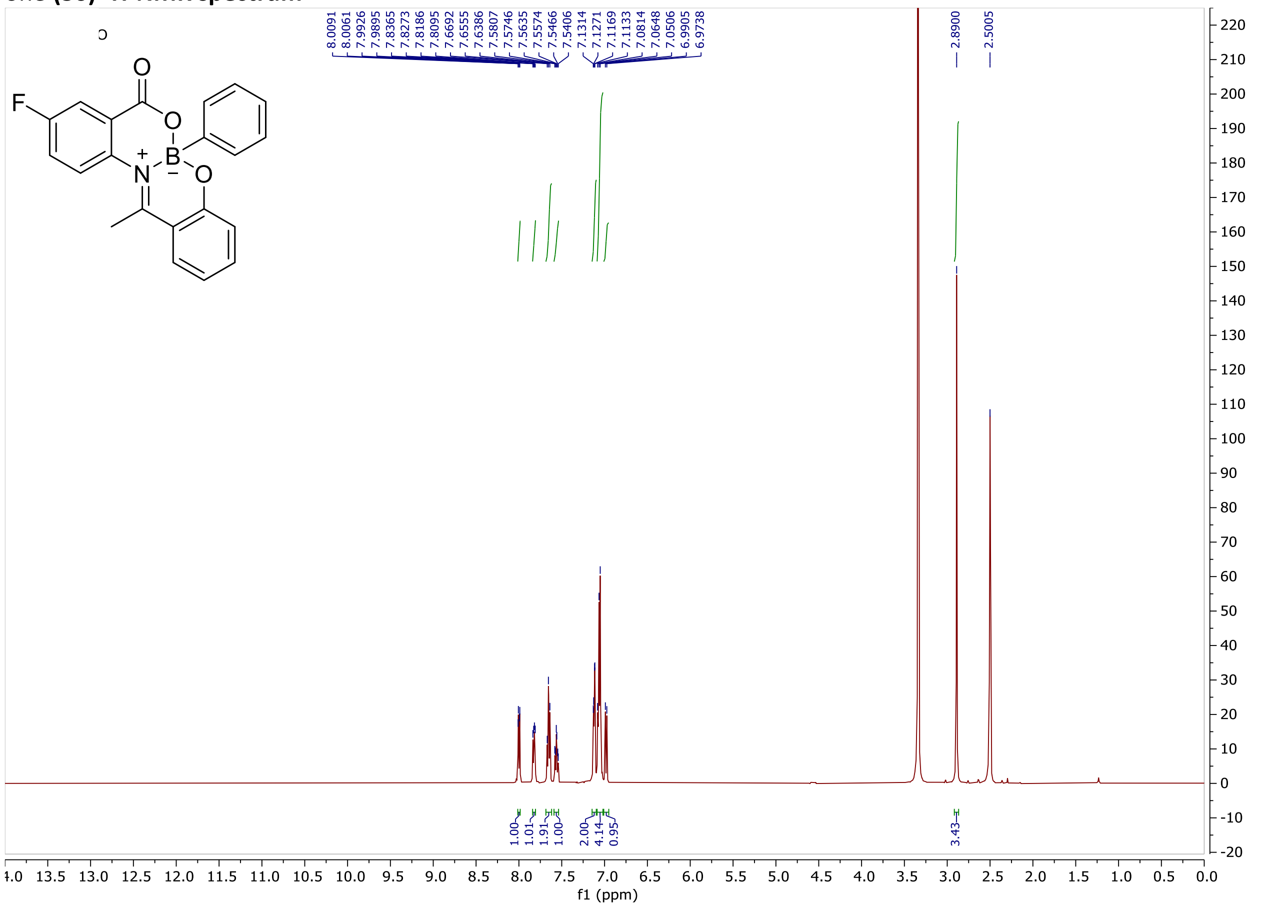

^{13}C-NMR spectrum

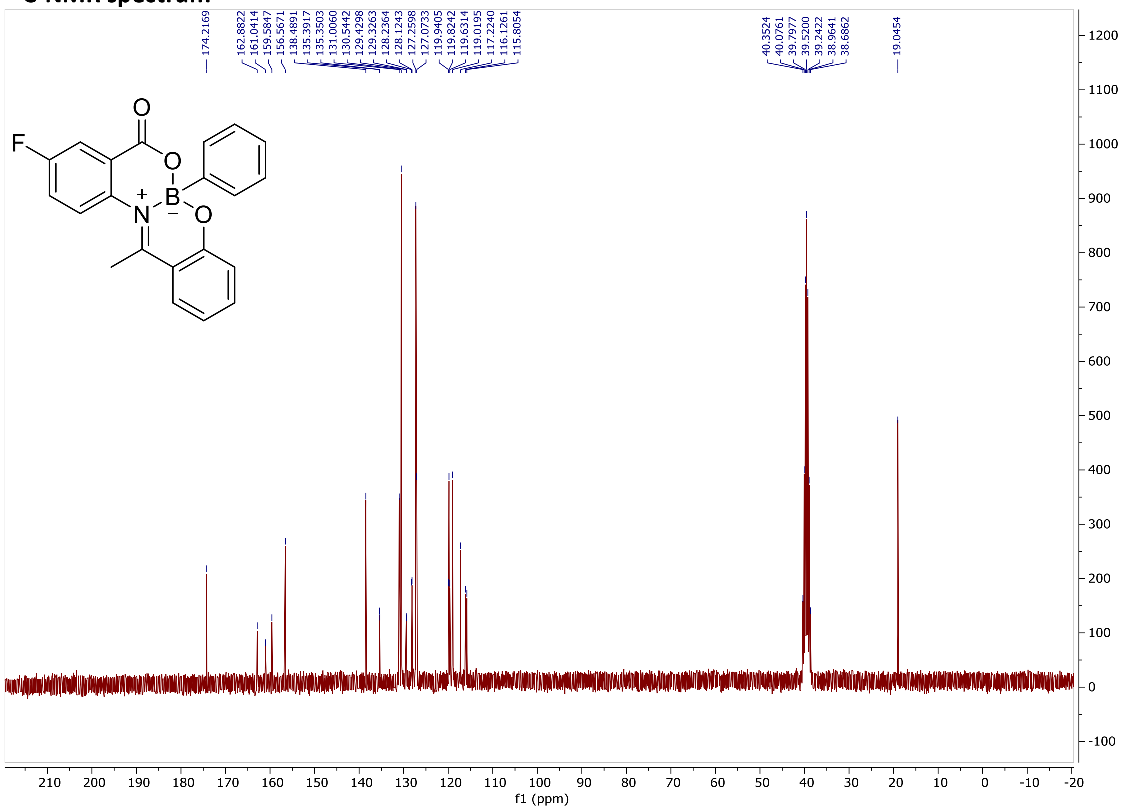

(30) HRMS

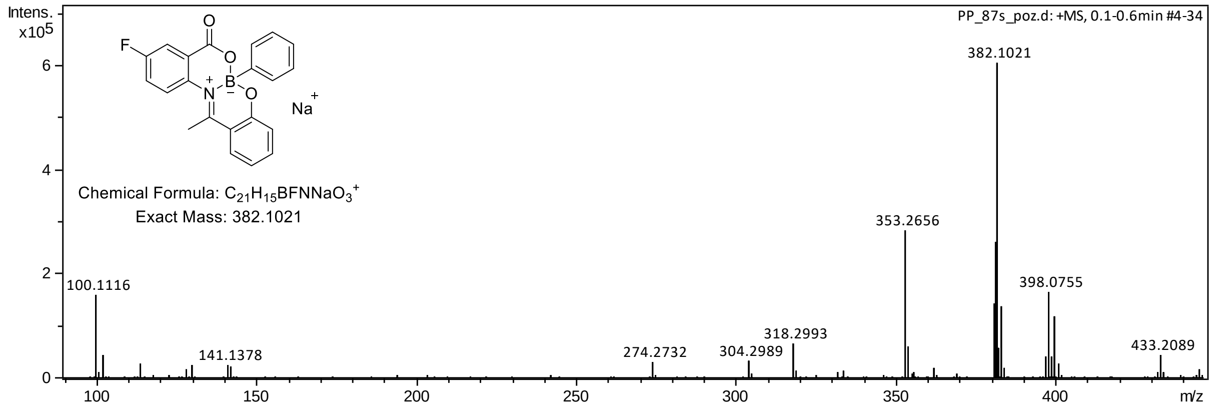

Intens.
x10^5
PP_87s_poz.d: +MS, 0.1-0.6min #4-34
Na+
Chemical Formula: C21H15BFNNaO3+
Exact Mass: 382.1021
382.1021
353.2656
398.0755
100.1116
433.2089
318.2993
141.1378
274.2732
304.2989
m/z

3-chloro-13-methyl-7-phenyl-5H,7H-7λ[4],14λ[4]-benzo[d]benzo[5,6][1,3,2]oxazabo-rinino[2,3-b][1,3,2]oxazaborinin-5-one **(31)** **^{1}H-NMR spectrum**

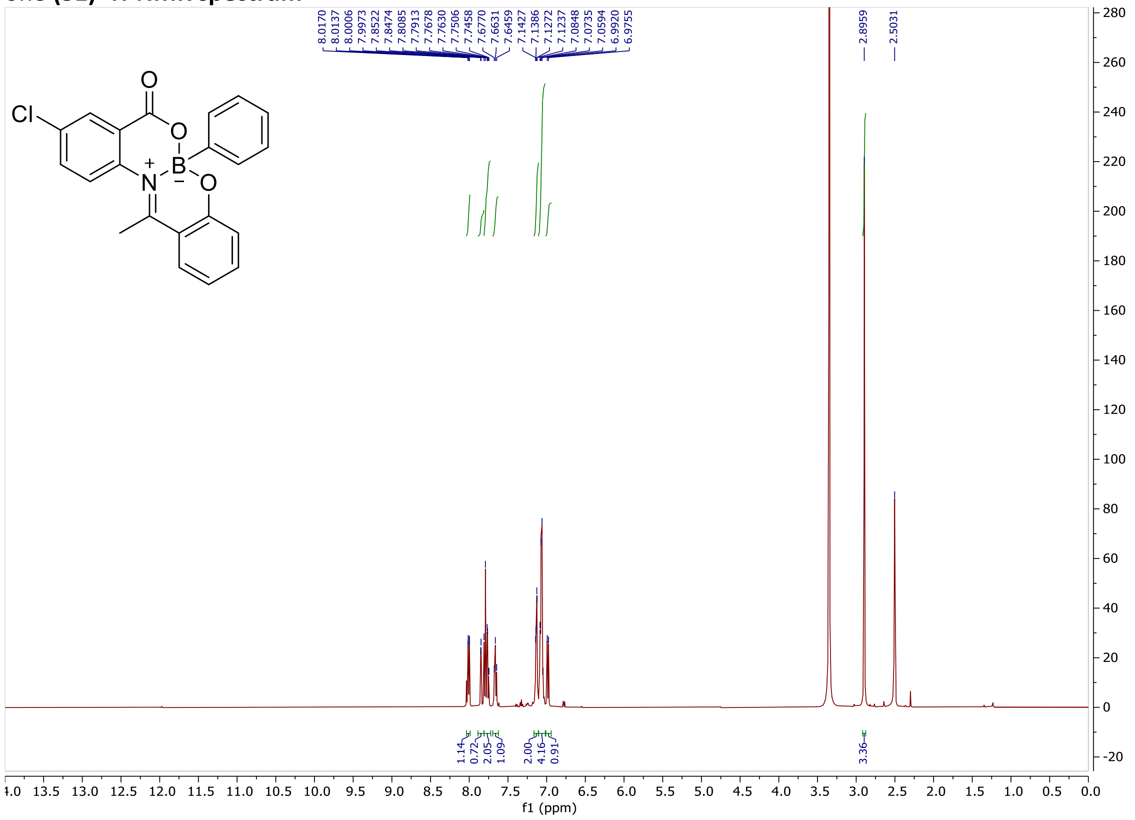

^{13}C-NMR spectrum

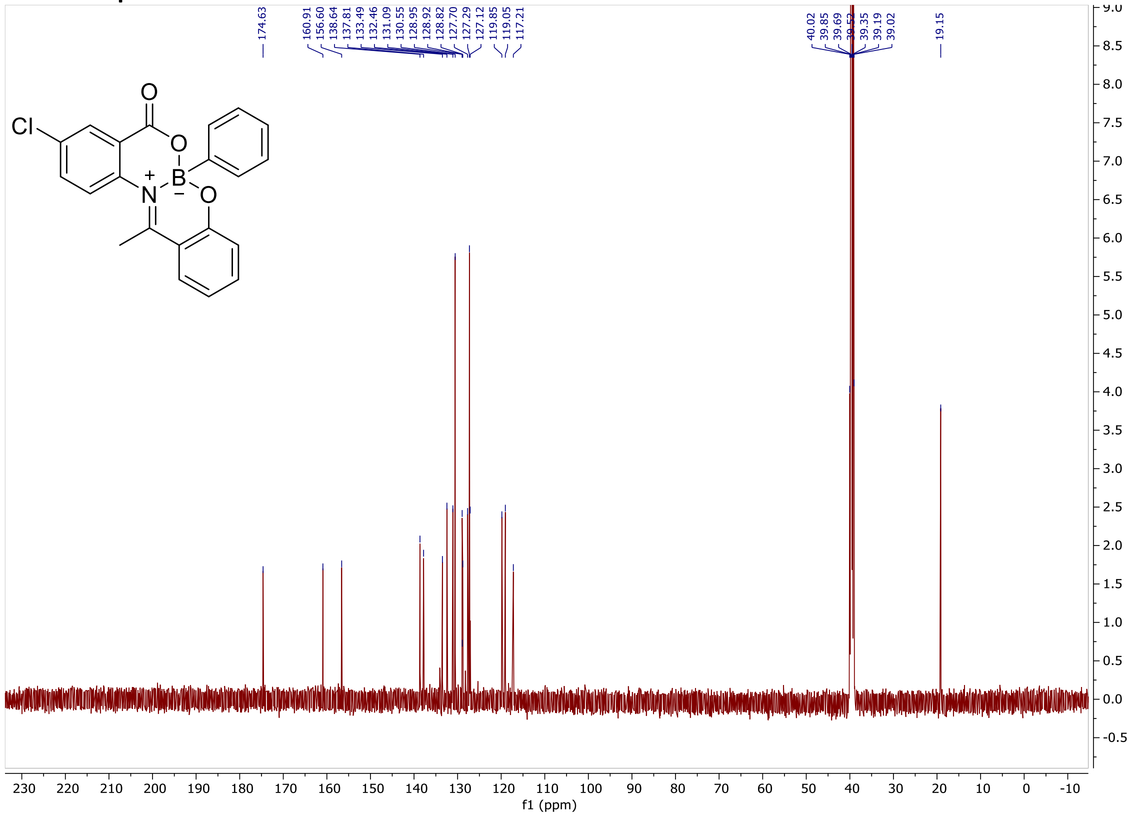

(31) HRMS

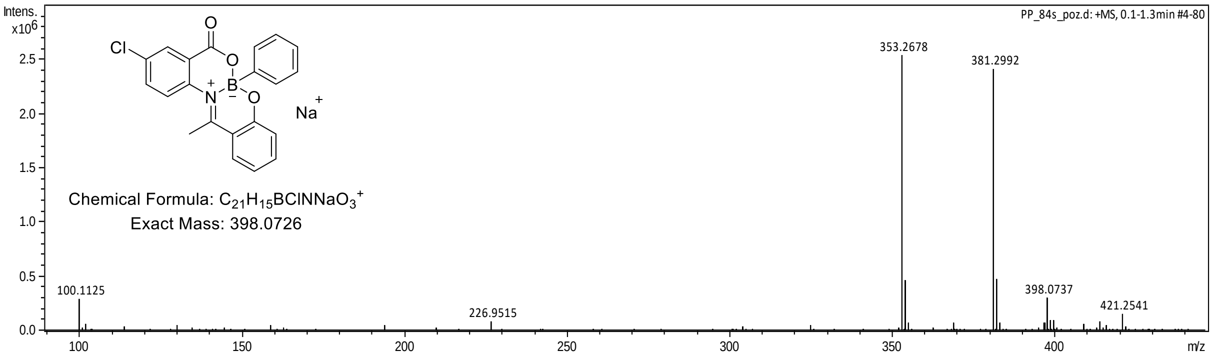
Intens.
x10^6
PP_84s_poz.d: +MS, 0.1-1.3min #4-80
Cl
O
O
B
N
O
Na+
Chemical Formula: C21H15BClNNaO3+
Exact Mass: 398.0726
353.2678
381.2992
100.1125
226.9515
398.0737
421.2541
m/z

3-bromo-13-methyl-7-phenyl-5H,7H-7λ4,14λ4-benzo[d]benzo[5,6][1,3,2]oxazabori-nino[2,3-b][1,3,2]oxazaborinin-5-one **(32)** **^{1}H-NMR spectrum**

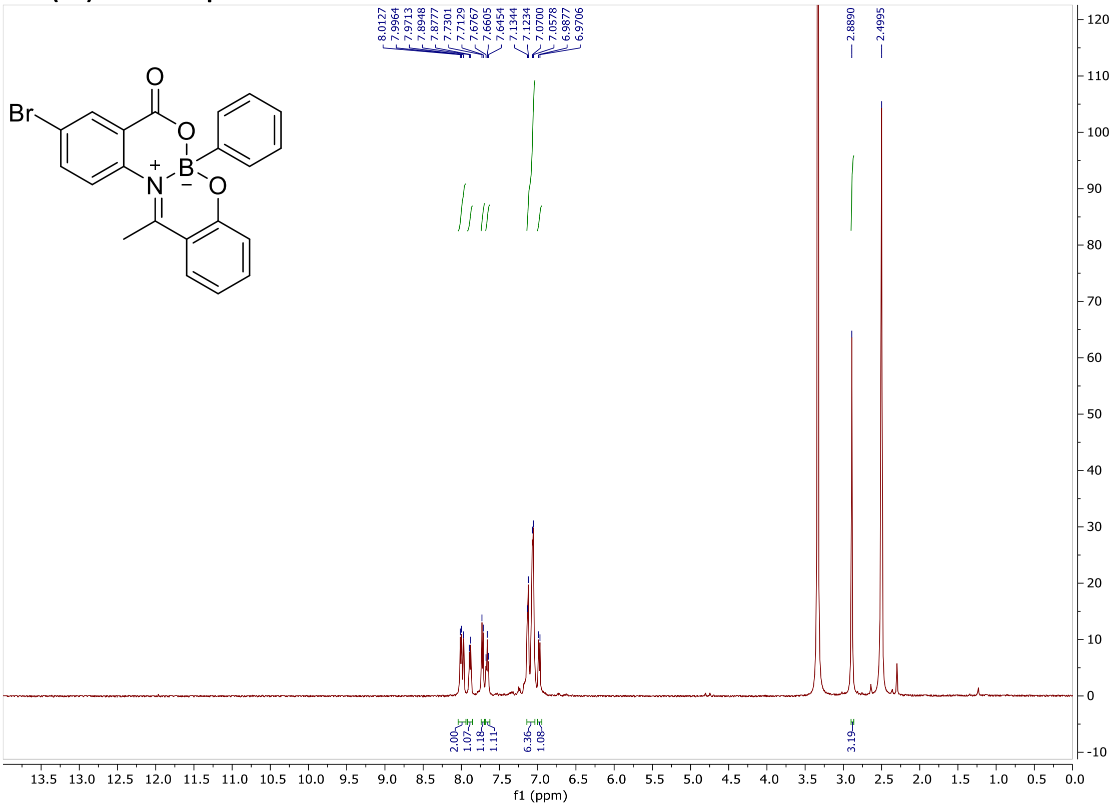

^{13}C-NMR spectrum

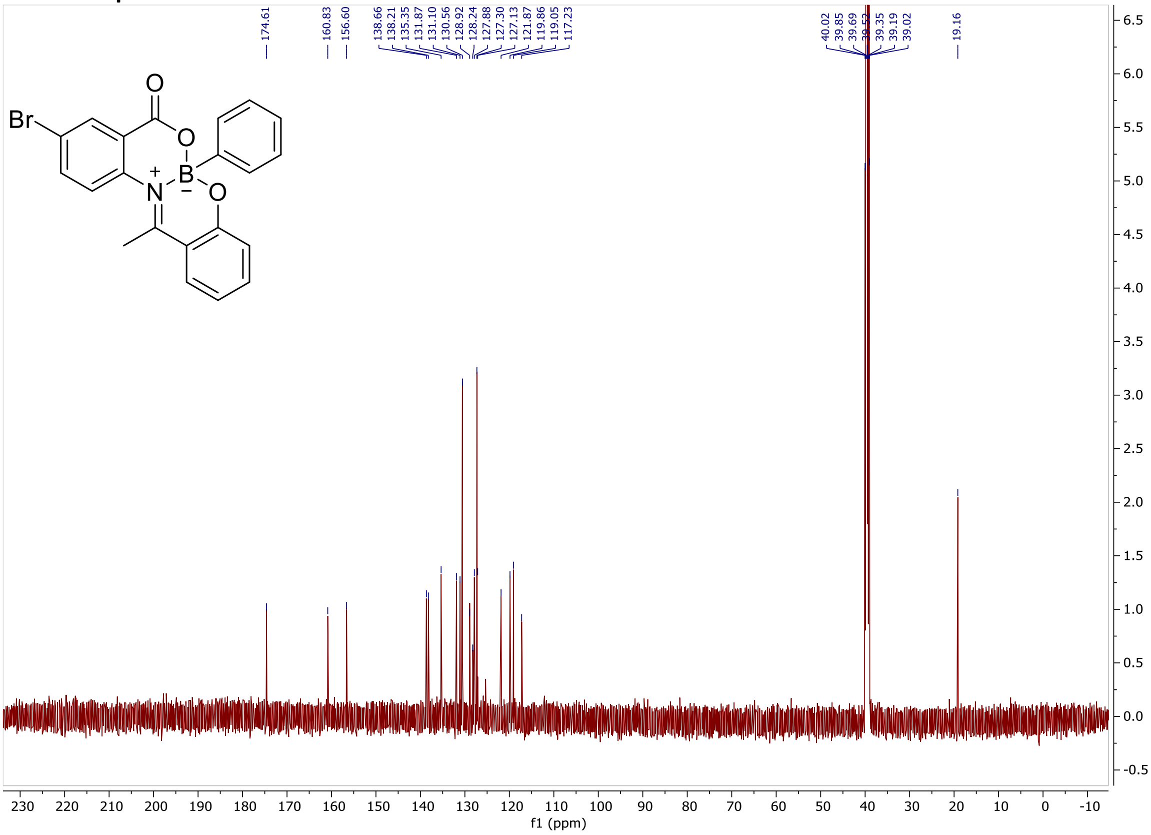

(32) HRMS

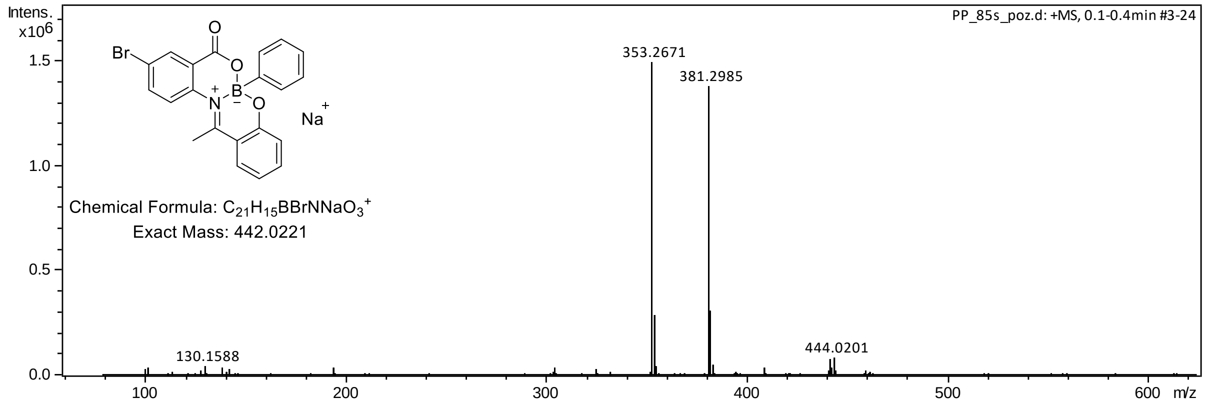
Intens.
x10^6
PP_85s_poz.d: +MS, 0.1-0.4min #3-24
Br
O
O
B
N
O
Na+
Chemical Formula: C21H15BBrNNaO3+
Exact Mass: 442.0221
353.2671
381.2985
130.1588
444.0201
1.5
1.0
0.5
0.0
100
200
300
400
500
600
m/z

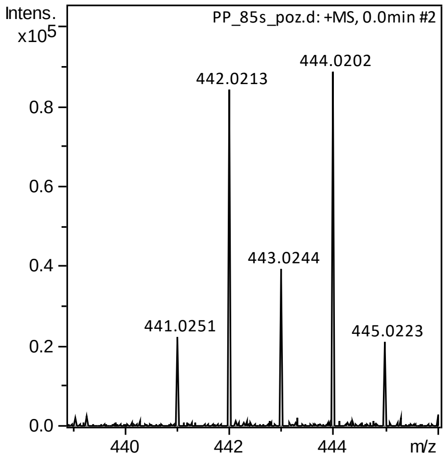
Intens.
x10^5
PP_85s_poz.d: +MS, 0.0min #2
442.0213
444.0202
443.0244
441.0251
445.0223
0.8
0.6
0.4
0.2
0.0
440
442
444
m/z

11-chloro-7-(4-chlorophenyl)-5H,7H-7λ[4],14λ[4]-benzo[d]benzo[5,6][1,3,2]oxaza-borinino[2,3-b][1,3,2]oxazaborinin-5-one **(33)** **^{1}H-NMR spectrum**

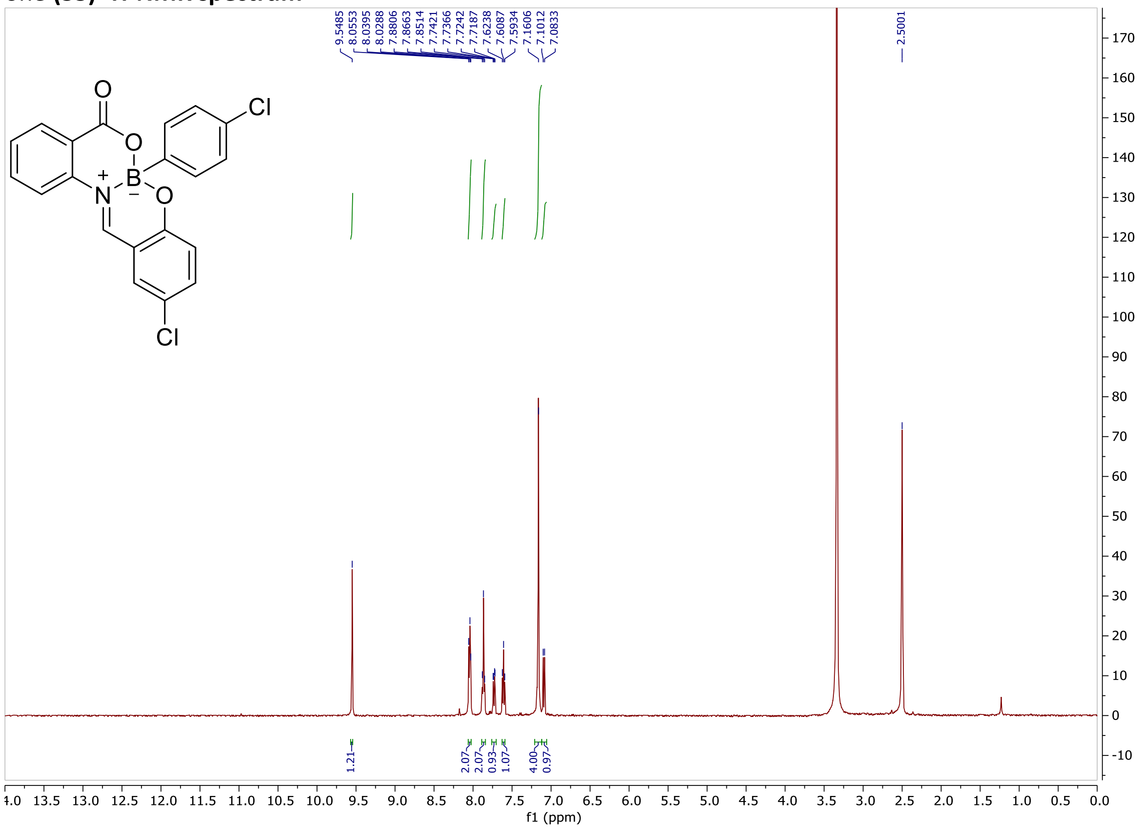

^{13}C-NMR spectrum

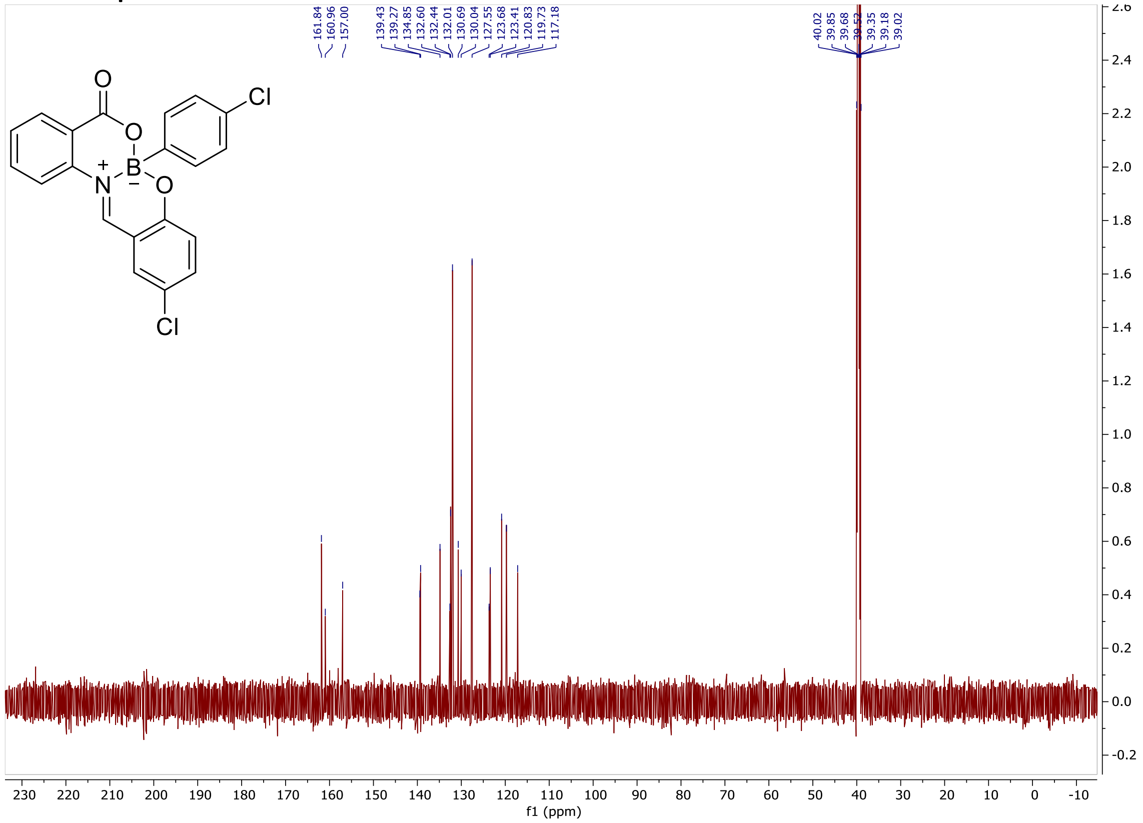

(33) HRMS

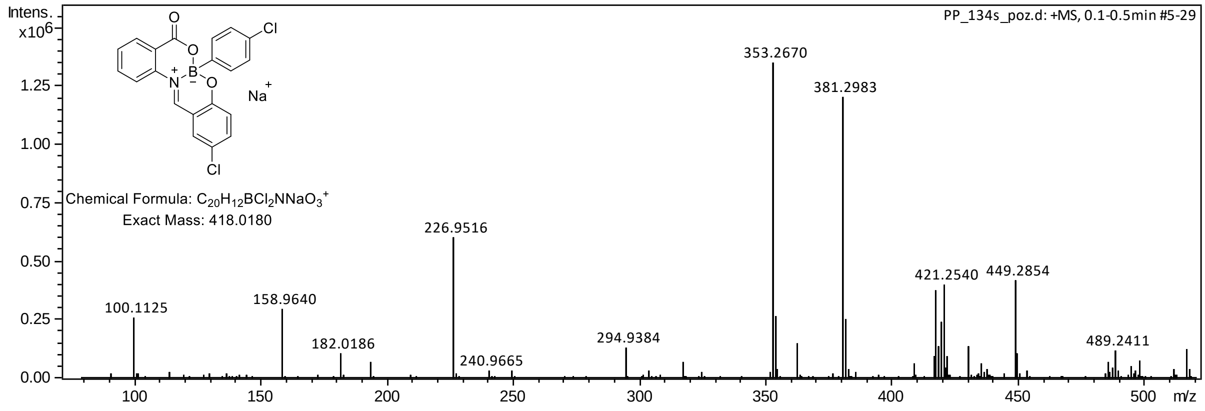

PP_134s_poz.d: +MS, 0.1-0.5min #5-29
Intens. x10^6
Chemical Formula: $C_{20}H_{12}BCl_2NNaO_3^+$
Exact Mass: 418.0180
Na+
Cl
353.2670
381.2983
226.9516
421.2540
449.2854
158.9640
100.1125
182.0186
294.9384
240.9665
489.2411
m/z

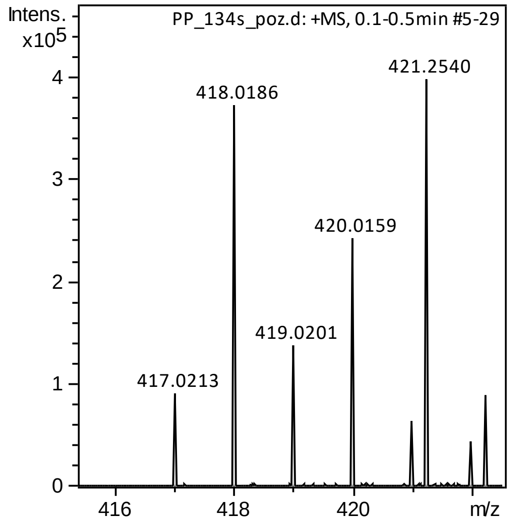

PP_134s_poz.d: +MS, 0.1-0.5min #5-29
Intens. x10^5
421.2540
418.0186
420.0159
419.0201
417.0213
m/z

11-chloro-7-(4-chlorophenyl)-3-methyl-5H,7H-7λ[4],14λ[4]-benzo[d]benzo[5,6][1,3,2] oxazaborinino[2,3-b][1,3,2]oxazaborinin-5-one **(34)** **^{1}H-NMR spectrum**

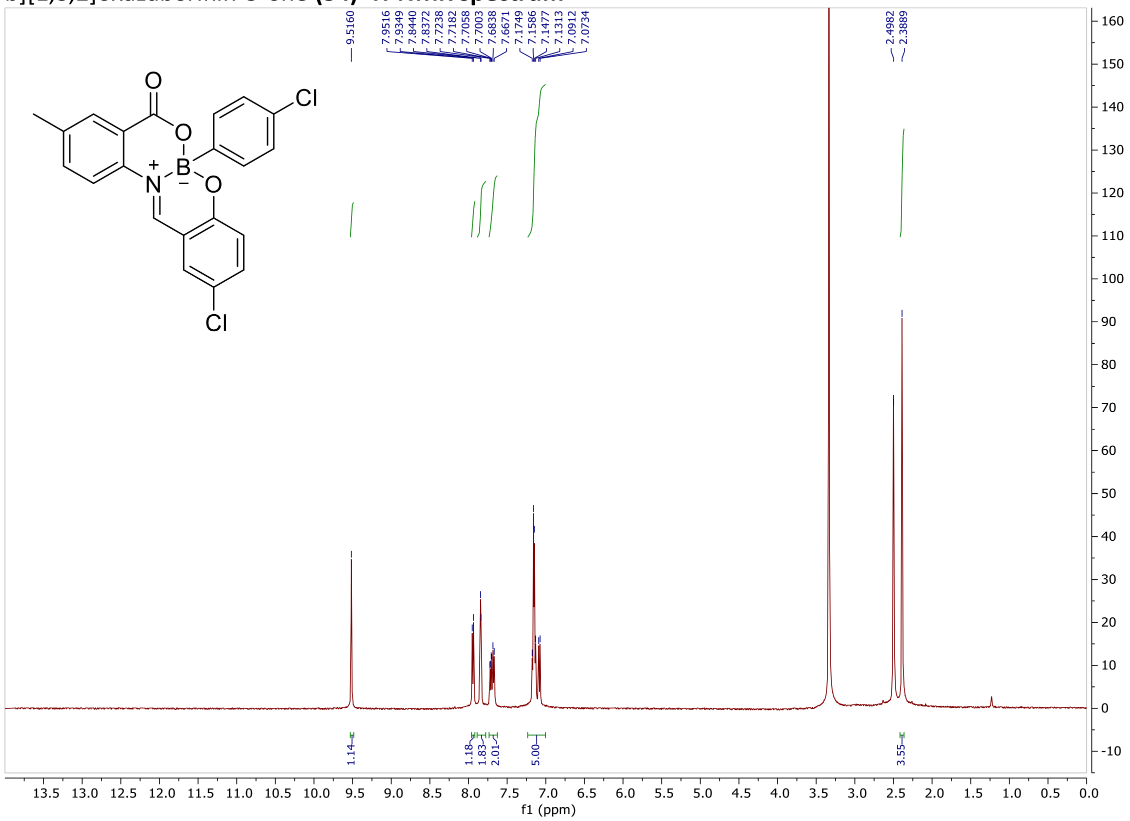

^{13}C-NMR spectrum

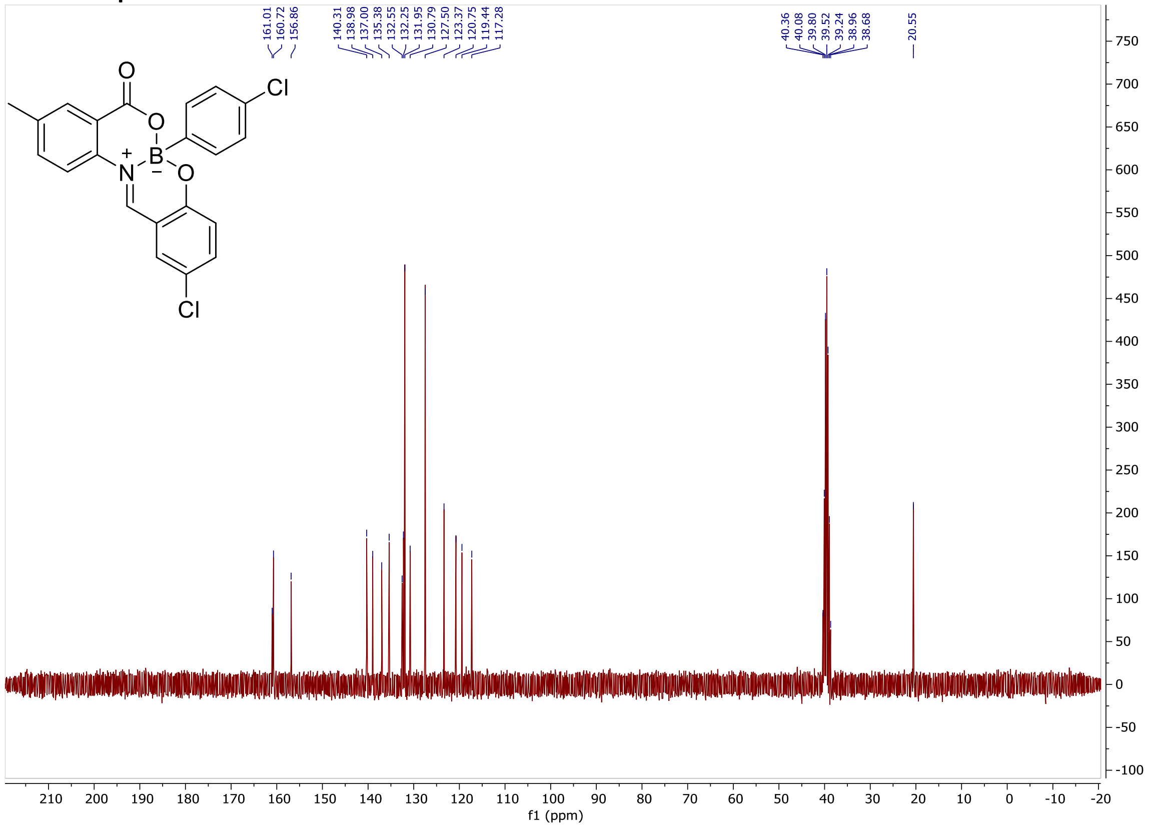

(34) HRMS

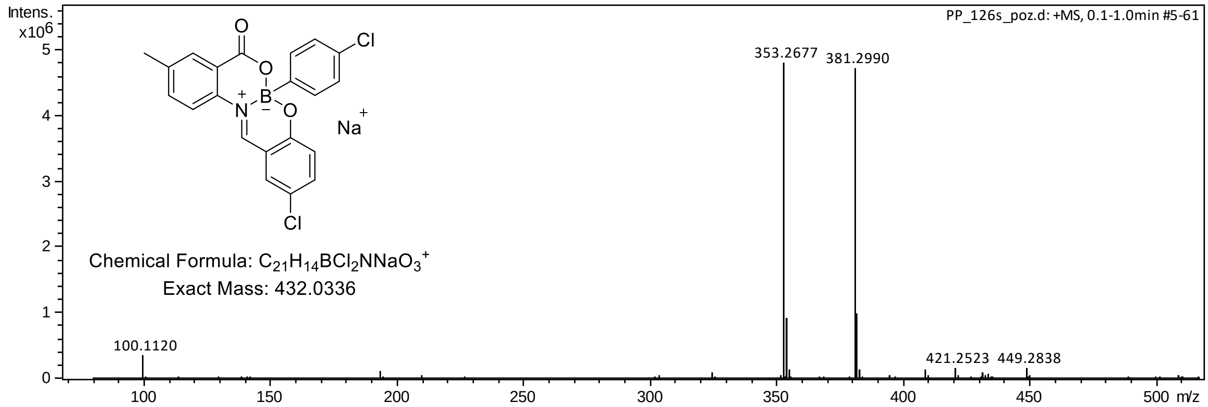
Intens.
x10^6
PP_126s_poz.d: +MS, 0.1-1.0min #5-61
Cl
O
Na+
Cl
Chemical Formula: C21H14BCl2NNaO3+
Exact Mass: 432.0336
353.2677
381.2990
100.1120
421.2523
449.2838
m/z

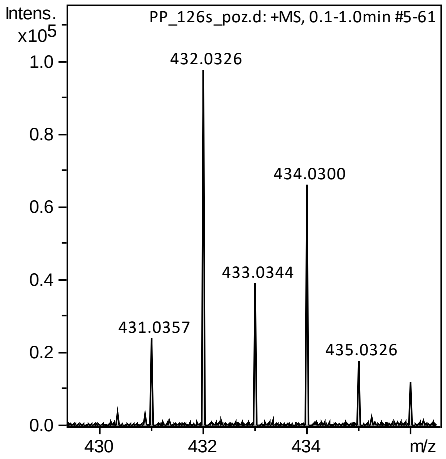
Intens.
x10^5
PP_126s_poz.d: +MS, 0.1-1.0min #5-61
432.0326
434.0300
433.0344
431.0357
435.0326
m/z

11-chloro-7-(4-chlorophenyl)-3-fluoro-5H,7H-7λ[4],14λ[4]-benzo[d]benzo[5,6][1,3,2] oxazaborinino[2,3-b][1,3,2]oxazaborinin-5-one **(35)** ^{1}**H-NMR spectrum**

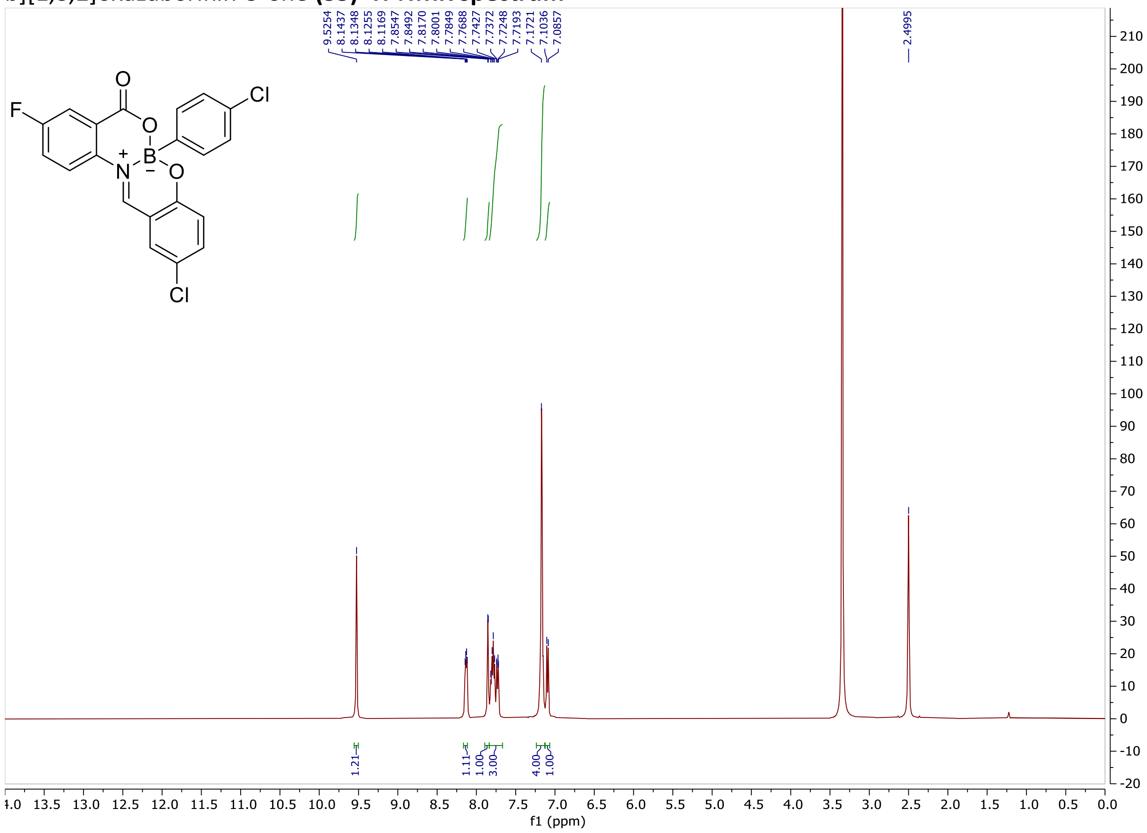

^{13}**C-NMR spectrum**

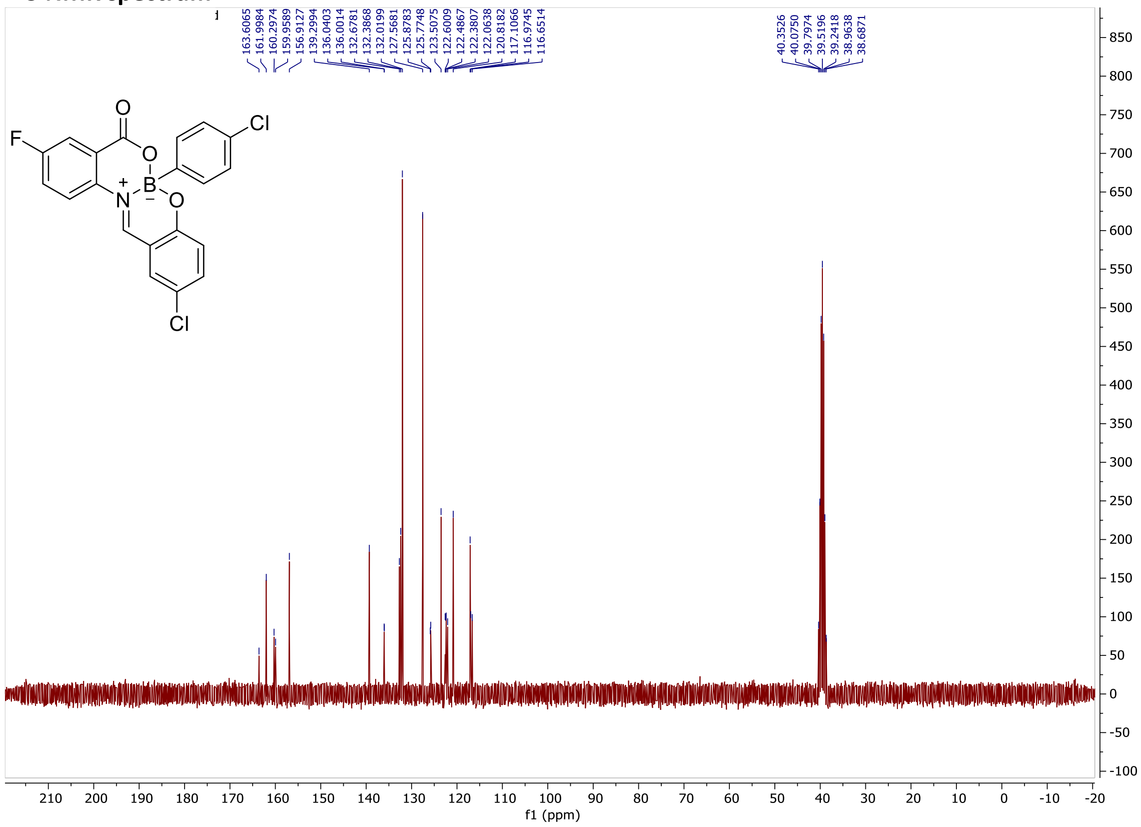

(35) HRMS

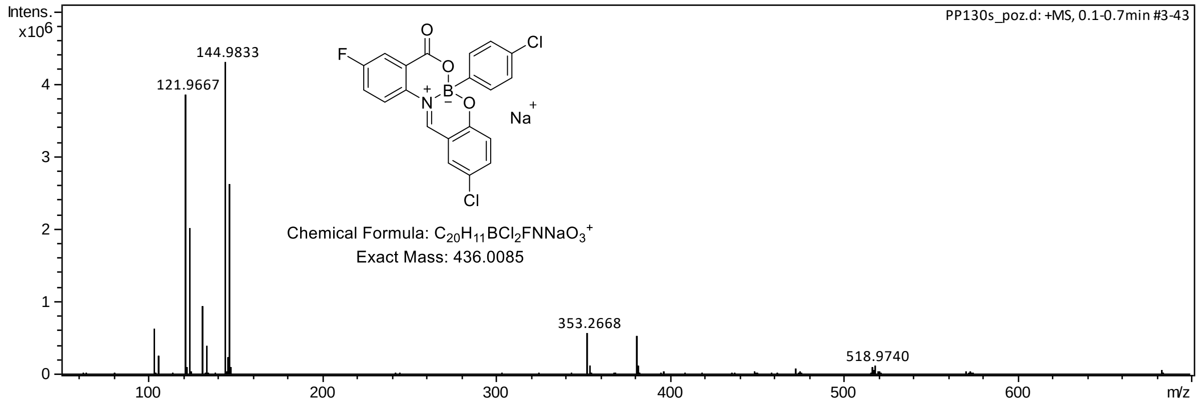

Intens.
x10^6
PP130s_poz.d: +MS, 0.1-0.7min #3-43
144.9833
121.9667
F
O
O
Cl
N
B
O
Na+
Cl
Chemical Formula: C20H11BCl2FNNaO3+
Exact Mass: 436.0085
353.2668
518.9740
100
200
300
400
500
600
m/z

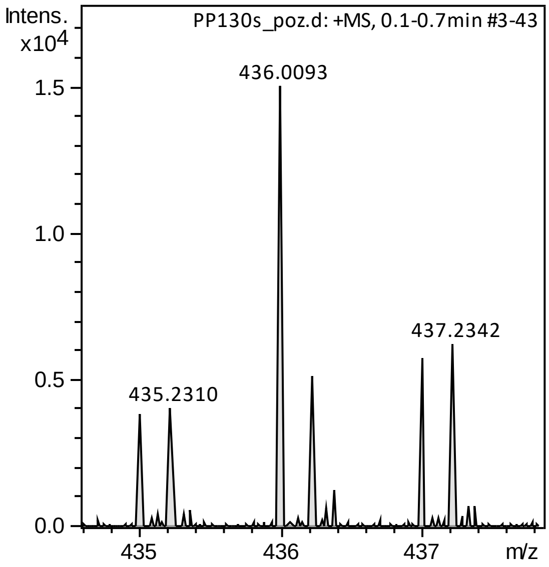

Intens.
x10^4
PP130s_poz.d: +MS, 0.1-0.7min #3-43
436.0093
437.2342
435.2310
435
436
437
m/z

3,11-dichloro-7-(4-chlorophenyl)-5H,7H-7λ[4],14λ[4]-benzo[d]benzo[5,6][1,3,2] oxazaborinino[2,3-b][1,3,2]oxazaborinin-5-one **(36)** **^{1}H-NMR spectrum**

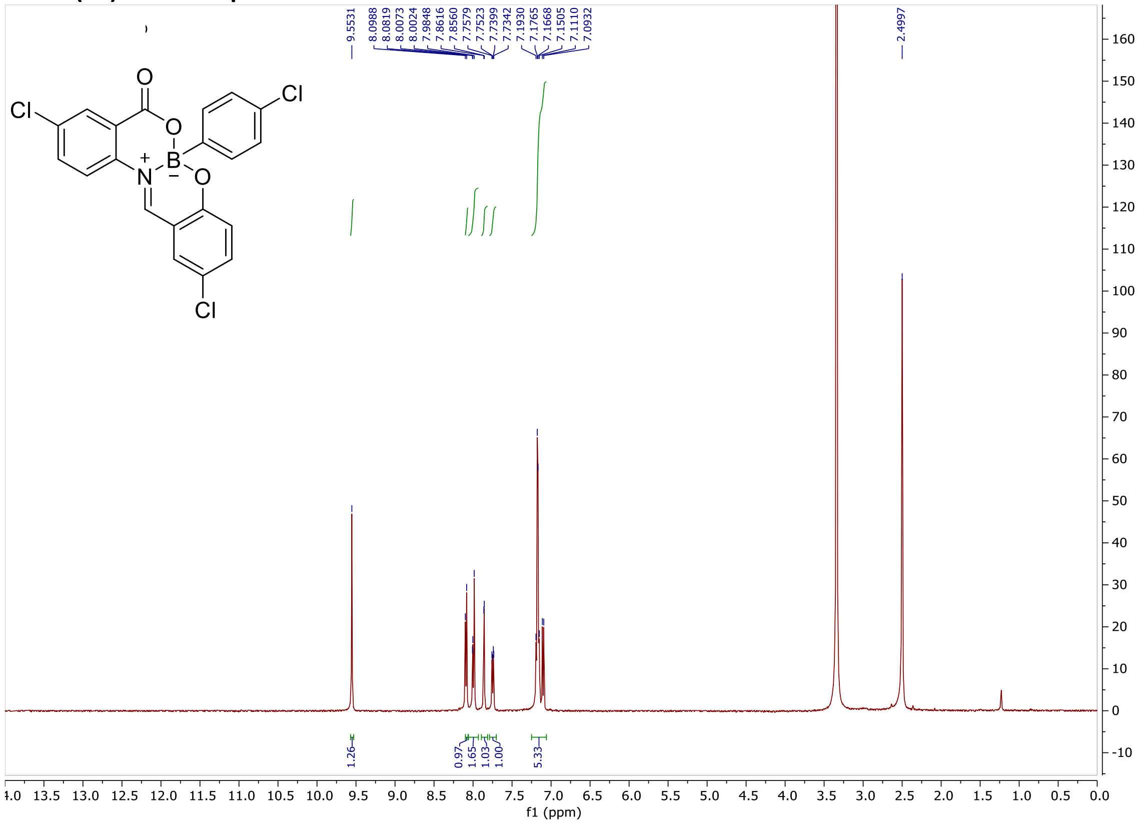

^{13}C-NMR spectrum

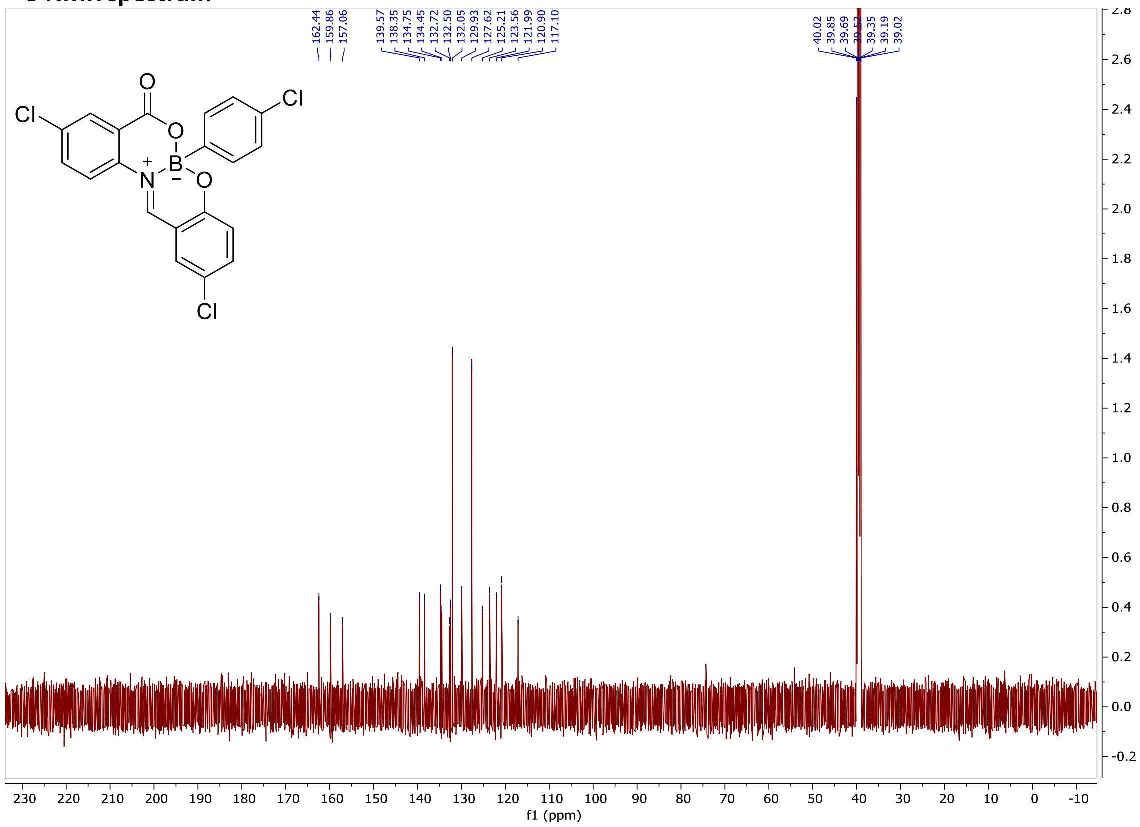

(36) HRMS

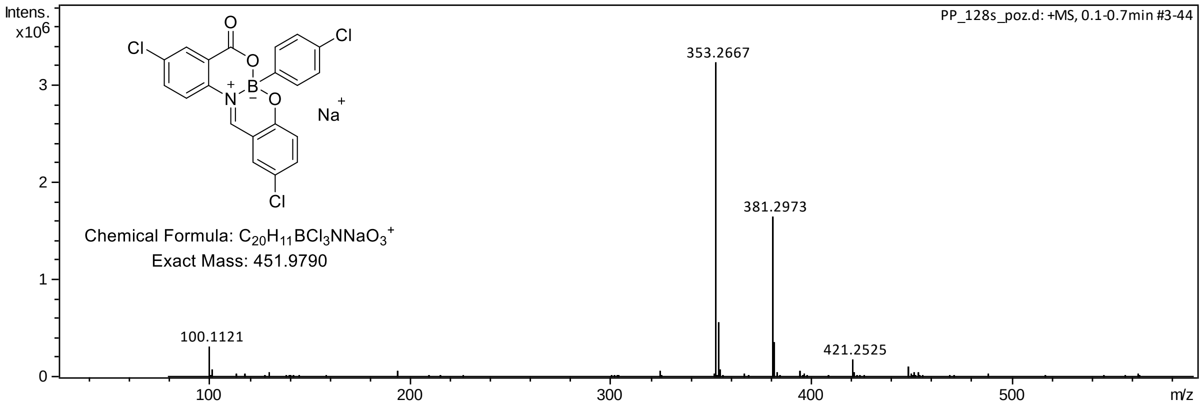
Intens.
x10^6
PP_128s_poz.d: +MS, 0.1-0.7min #3-44
Chemical Formula: $C_{20}H_{11}BCl_3NNaO_3^+$
Exact Mass: 451.9790
353.2667
381.2973
421.2525
100.1121
m/z

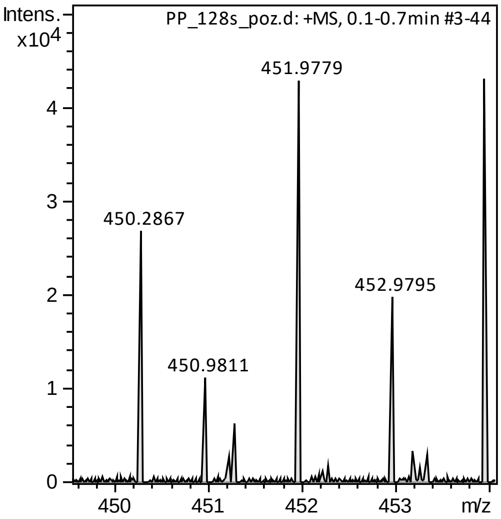
Intens.
x10^4
PP_128s_poz.d: +MS, 0.1-0.7min #3-44
451.9779
450.2867
452.9795
450.9811
m/z

3-bromo-11-chloro-7-(4-chlorophenyl)-5H,7H-7λ^4,14 λ^4-benzo[d]benzo[5,6][1,3,2] oxazaborinino[2,3-b][1,3,2]oxazaborinin-5-one **(37)** ^{1}**H-NMR spectrum**

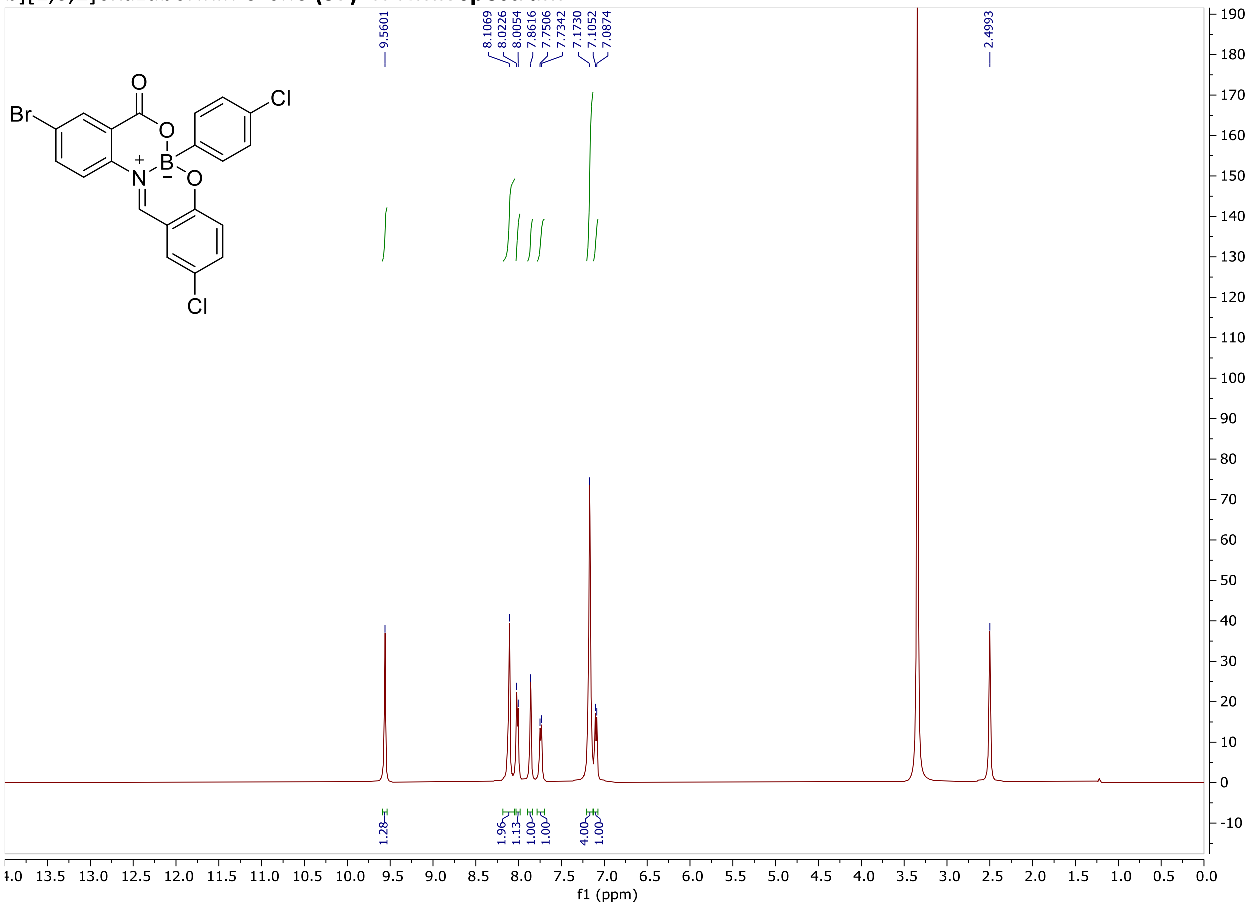

^{13}**C-NMR spectrum**

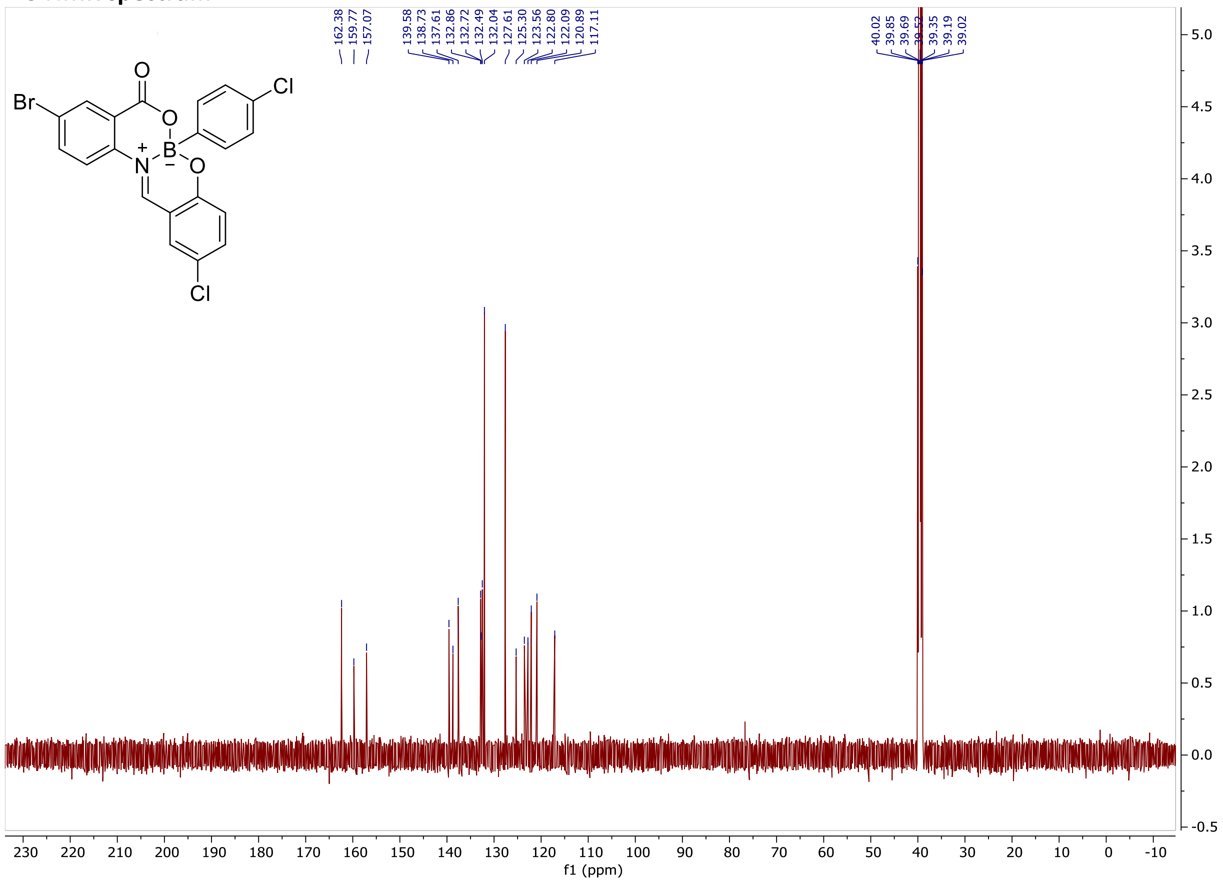

(37) HRMS

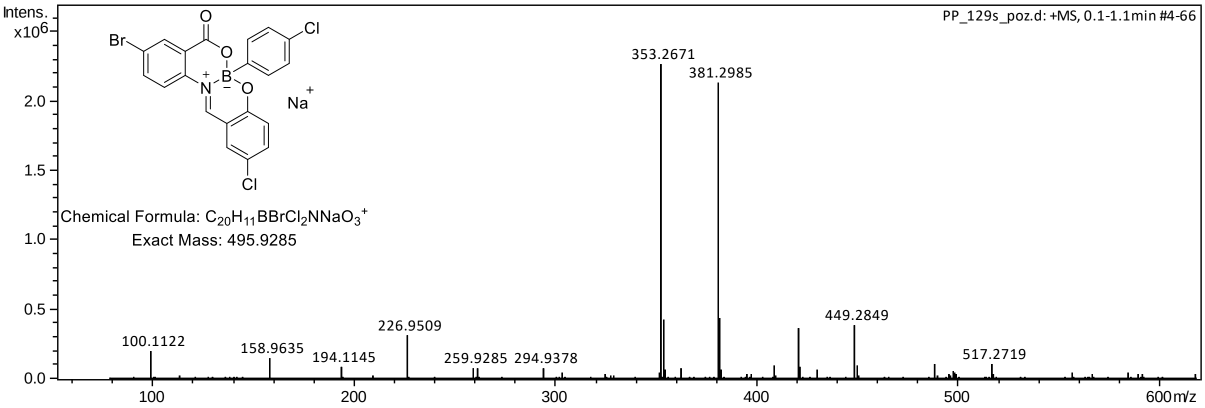
Intens.
x10^6
PP_129s_poz.d: +MS, 0.1-1.1min #4-66
Br
O
O
B
N
O
Cl
Na+
Cl
Chemical Formula: $C_{20}H_{11}BBrCl_2NNaO_3^+$
Exact Mass: 495.9285
353.2671
381.2985
449.2849
226.9509
100.1122
158.9635
194.1145
259.9285
294.9378
517.2719
2.0
1.5
1.0
0.5
0.0
100
200
300
400
500
600 m/z

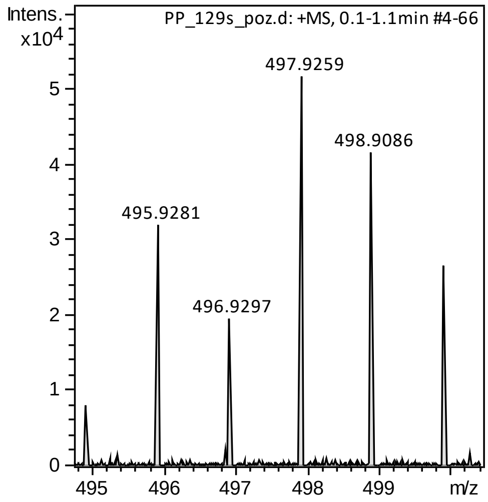
Intens.
x10^4
PP_129s_poz.d: +MS, 0.1-1.1min #4-66
497.9259
498.9086
495.9281
496.9297
5
4
3
2
1
0
495
496
497
498
499
m/z

11-chloro-7-(4-chlorophenyl)-3-iodo-5H,7H-7λ^4,14λ^4-benzo[d]benzo[5,6][1,3,2] oxazaborinino[2,3-b][1,3,2]oxazaborinin-5-one **(38)** **^{1}H-NMR spectrum**

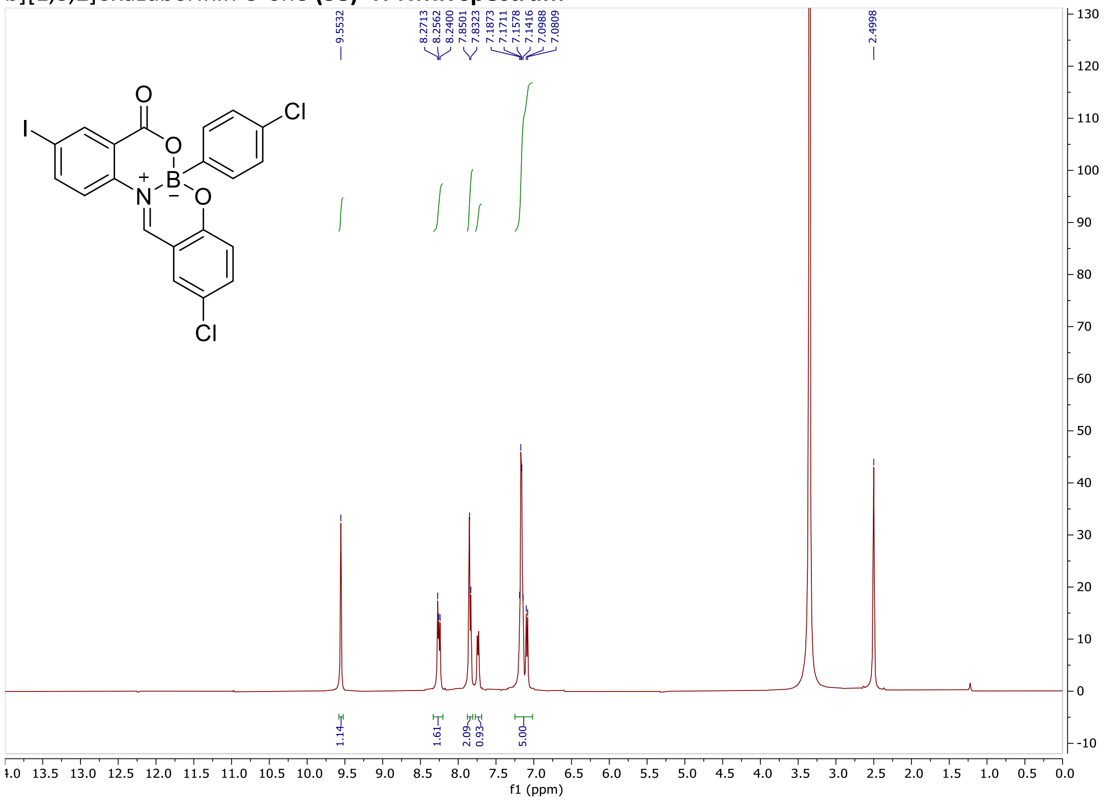

^{13}C-NMR spectrum

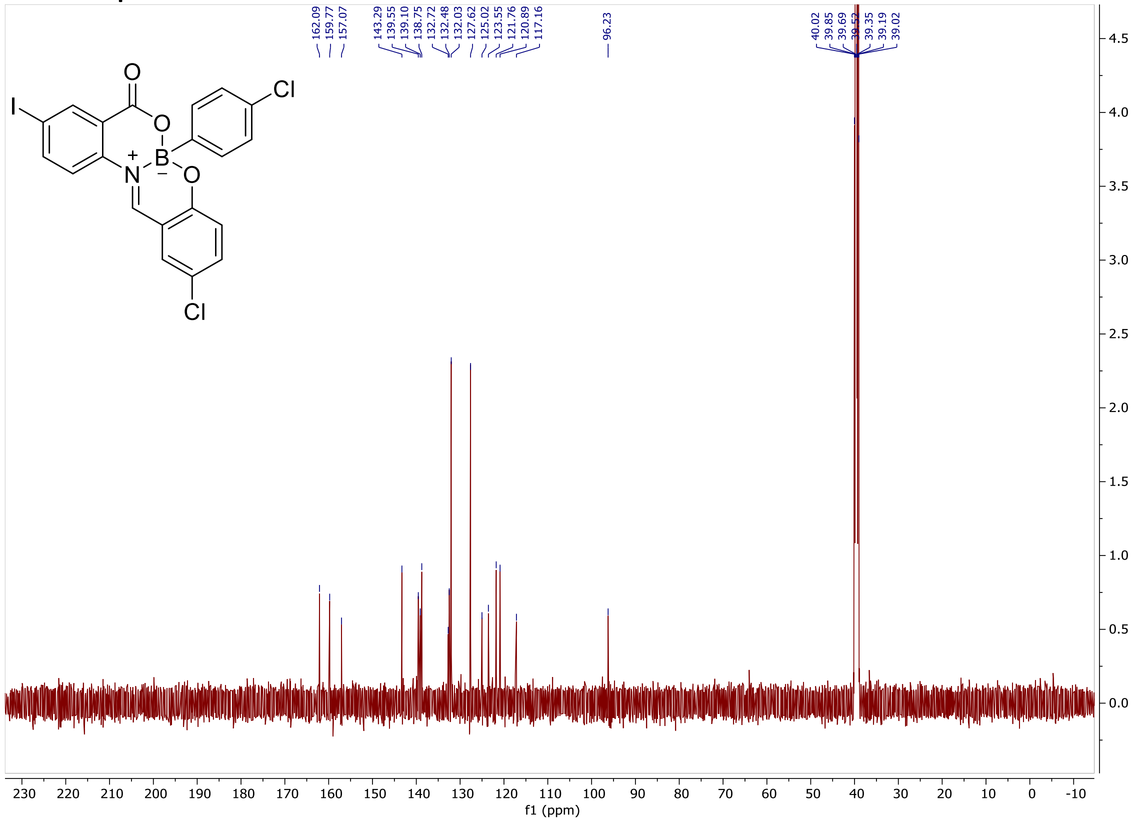

(38) HRMS

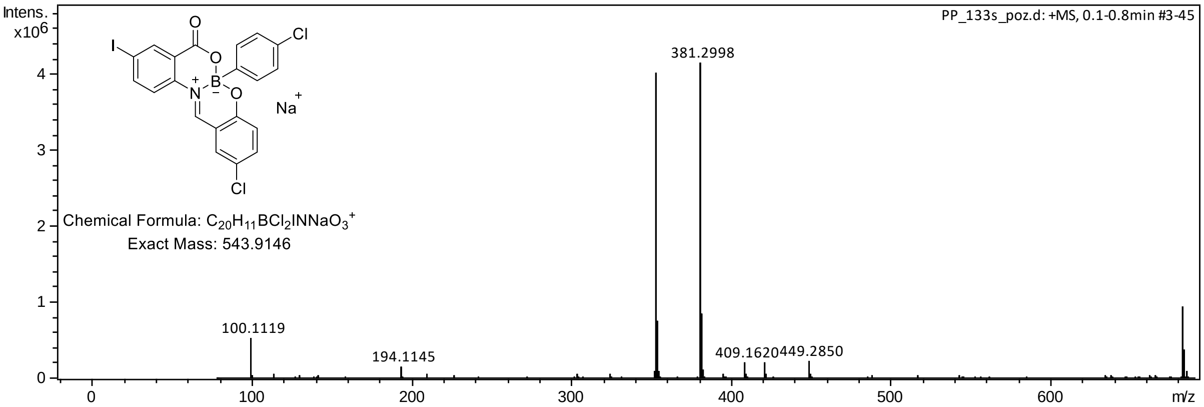

Intens.
x10^6
PP_133s_poz.d: +MS, 0.1-0.8min #3-45
Chemical Formula: C20H11BCl2INNaO3+
Exact Mass: 543.9146
381.2998
100.1119
194.1145
409.1620
449.2850
m/z

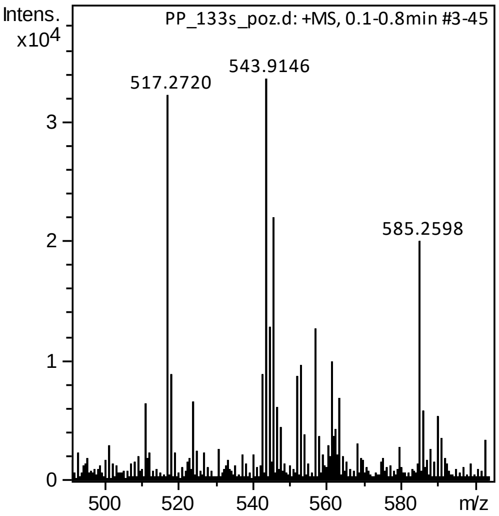

Intens.
x10^4
PP_133s_poz.d: +MS, 0.1-0.8min #3-45
517.2720
543.9146
585.2598
m/z

2,11-dichloro-7-(4-chlorophenyl)-5H,7H-7λ[4],14λ[4]-benzo[d]benzo[5,6][1,3,2] oxazaborinino[2,3-b][1,3,2]oxazaborinin-5-one **(39)** **^{1}H-NMR spectrum**

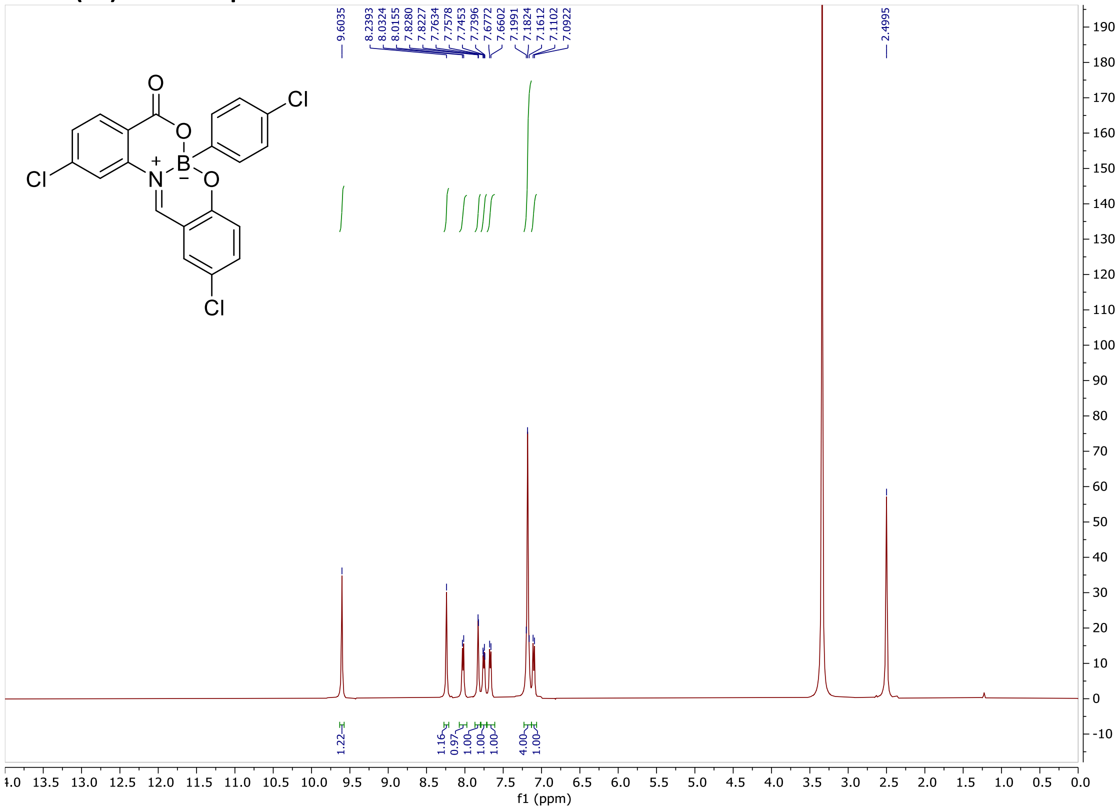

^{13}C-NMR spectrum

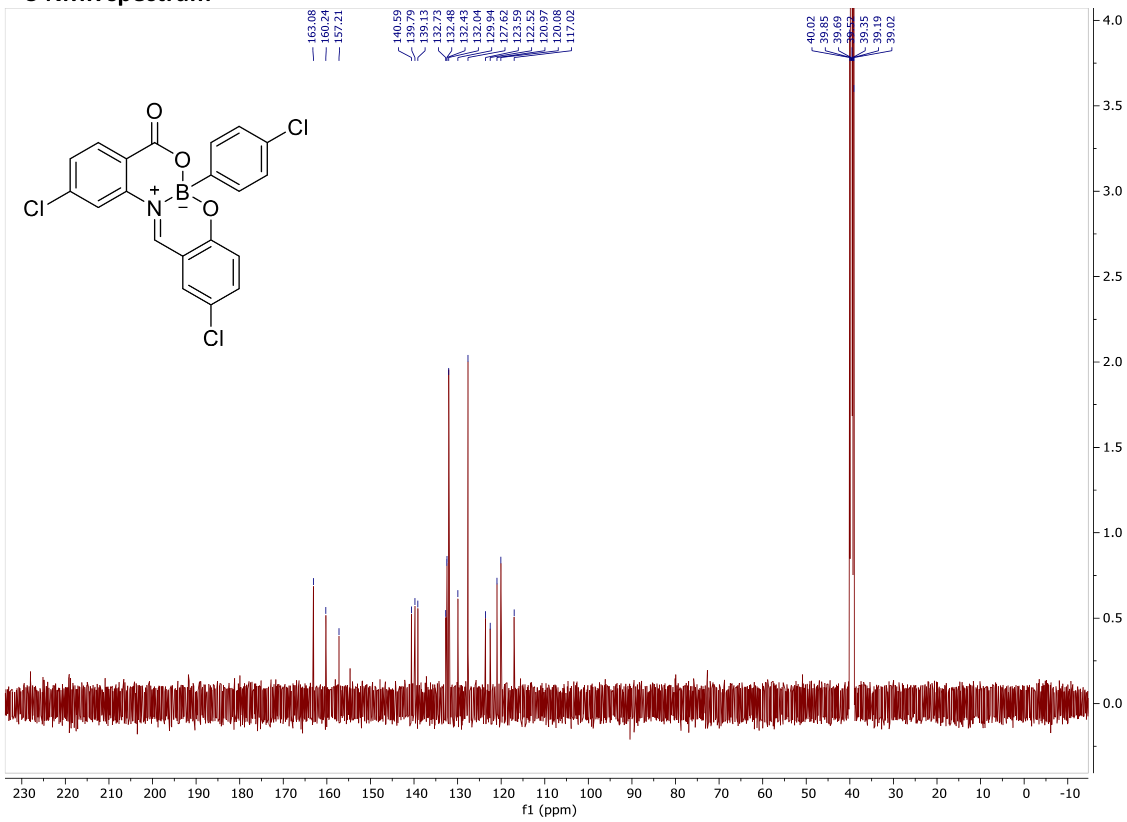

(39) HRMS

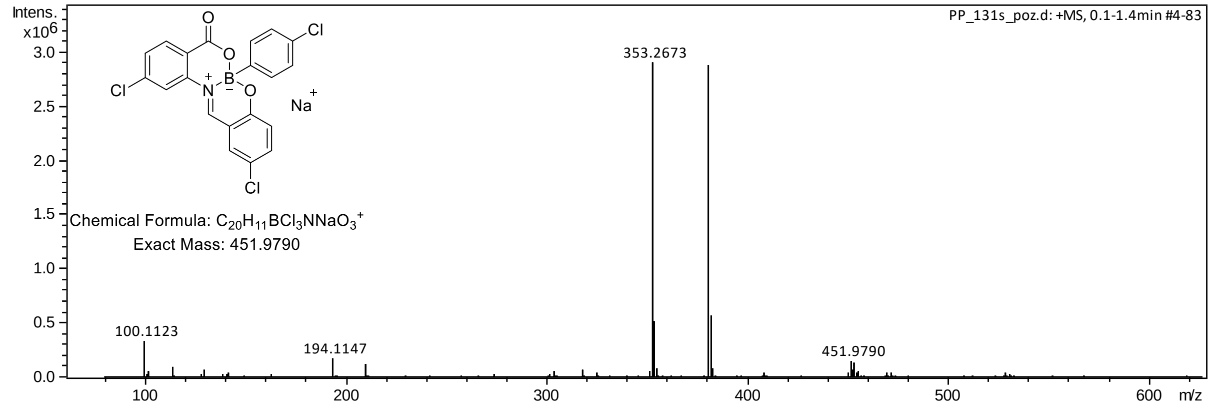
Intens.
x10^6
PP_131s_poz.d: +MS, 0.1-1.4min #4-83
353.2673
100.1123
194.1147
451.9790
Chemical Formula: C20H11BCl3NNaO3+
Exact Mass: 451.9790
Na+
m/z

11-chloro-7-(4-chlorophenyl)-2-nitro-5H,7H-7λ[4],14λ[4]-benzo[d]benzo[5,6][1,3,2] oxazaborinino[2,3-b][1,3,2]oxazaborinin-5-one **(40)** ^{1}**H-NMR spectrum**

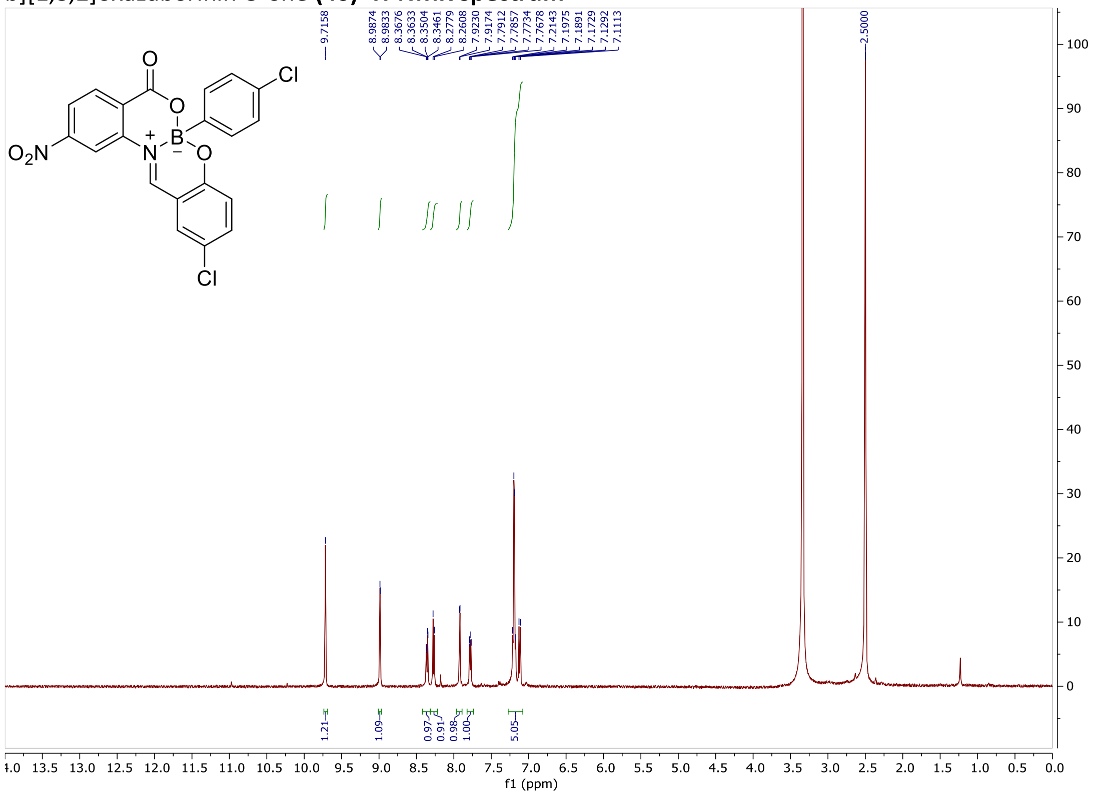

^{13}**C-NMR spectrum**

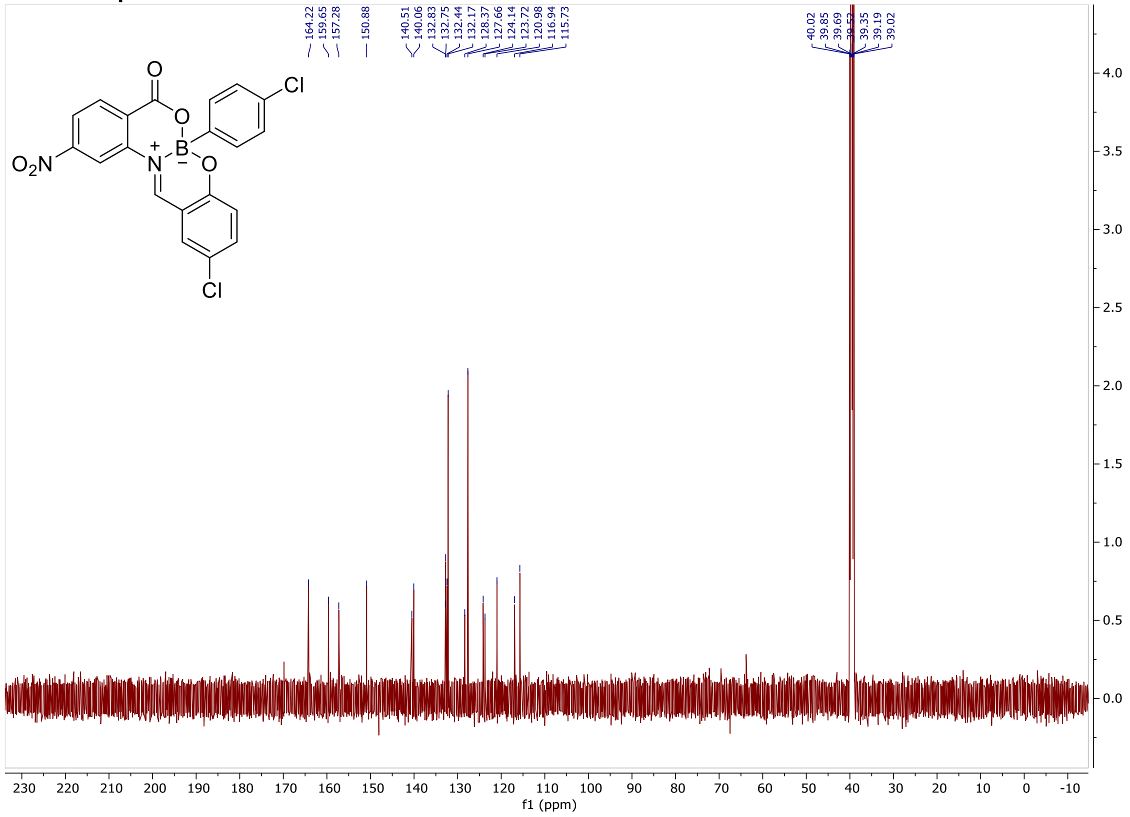

(40) HRMS

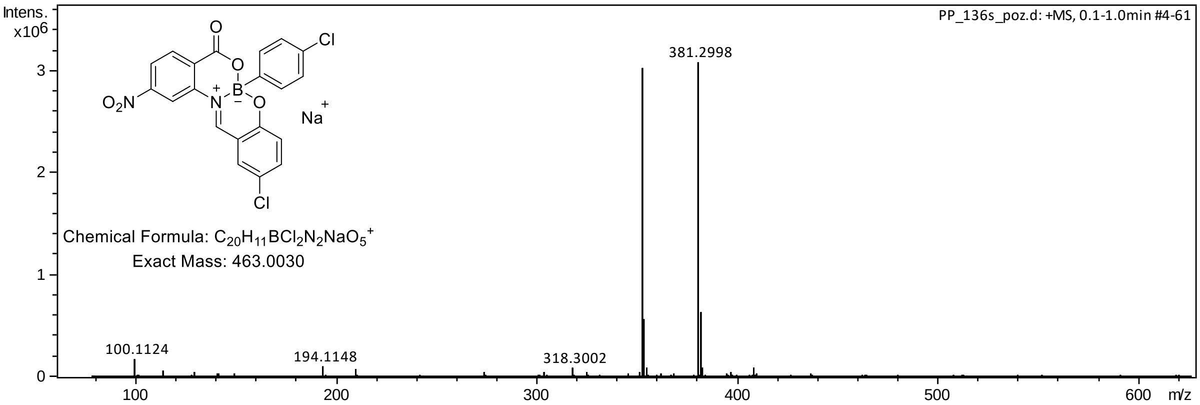
Intens.
x10^6
PP_136s_poz.d: +MS, 0.1-1.0min #4-61
381.2998
O2N
Cl
Cl
Na+
Chemical Formula: C20H11BCl2N2NaO5+
Exact Mass: 463.0030
100.1124
194.1148
318.3002
m/z

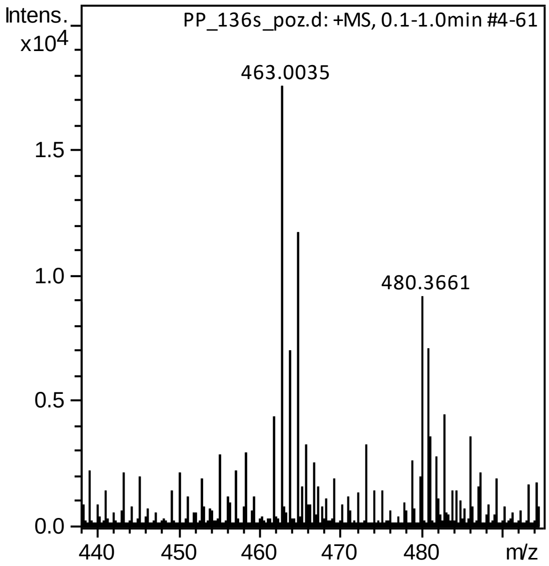
Intens.
x10^4
PP_136s_poz.d: +MS, 0.1-1.0min #4-61
463.0035
480.3661
m/z

11-bromo-7-(4-bromophenyl)-5H,7H-7λ[4],14λ[4]-benzo[d]benzo[5,6][1,3,2]oxazabo-rinino[2,3-b][1,3,2]oxazaborinin-5-one **(41)** **^{1}H-NMR spectrum**

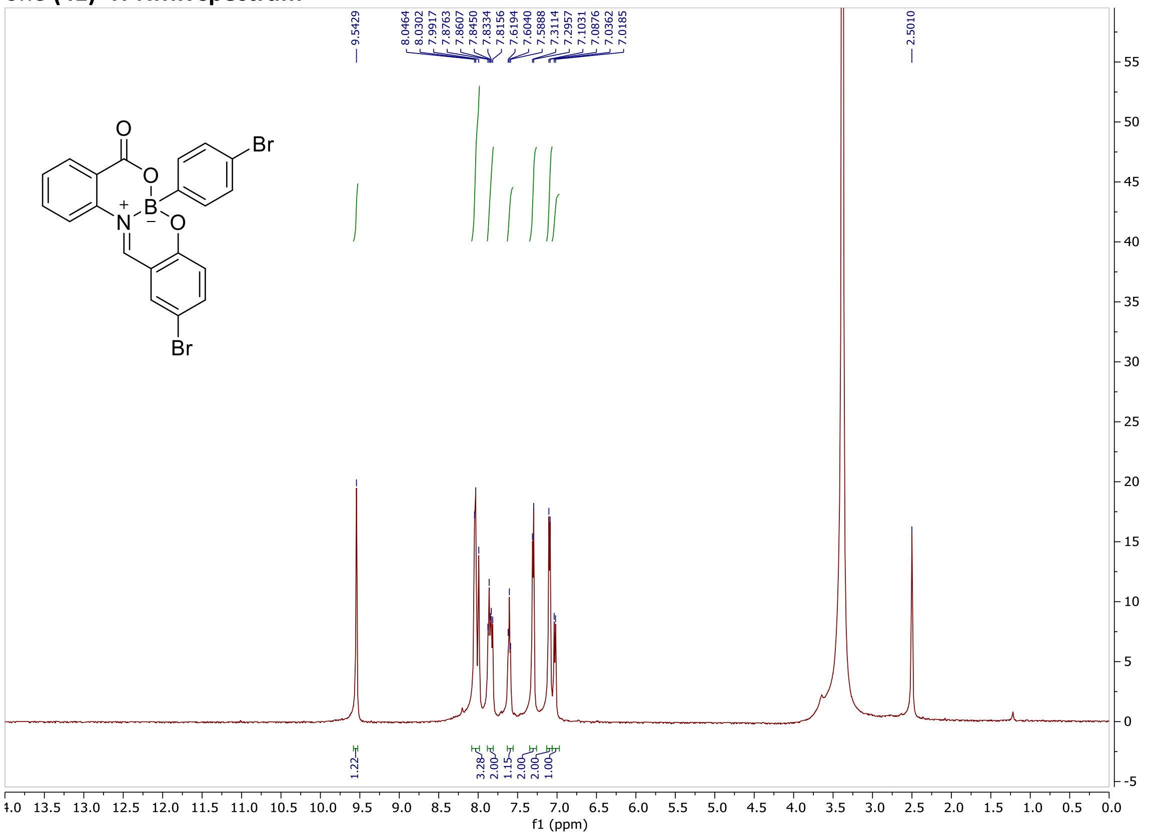

^{13}C-NMR spectrum

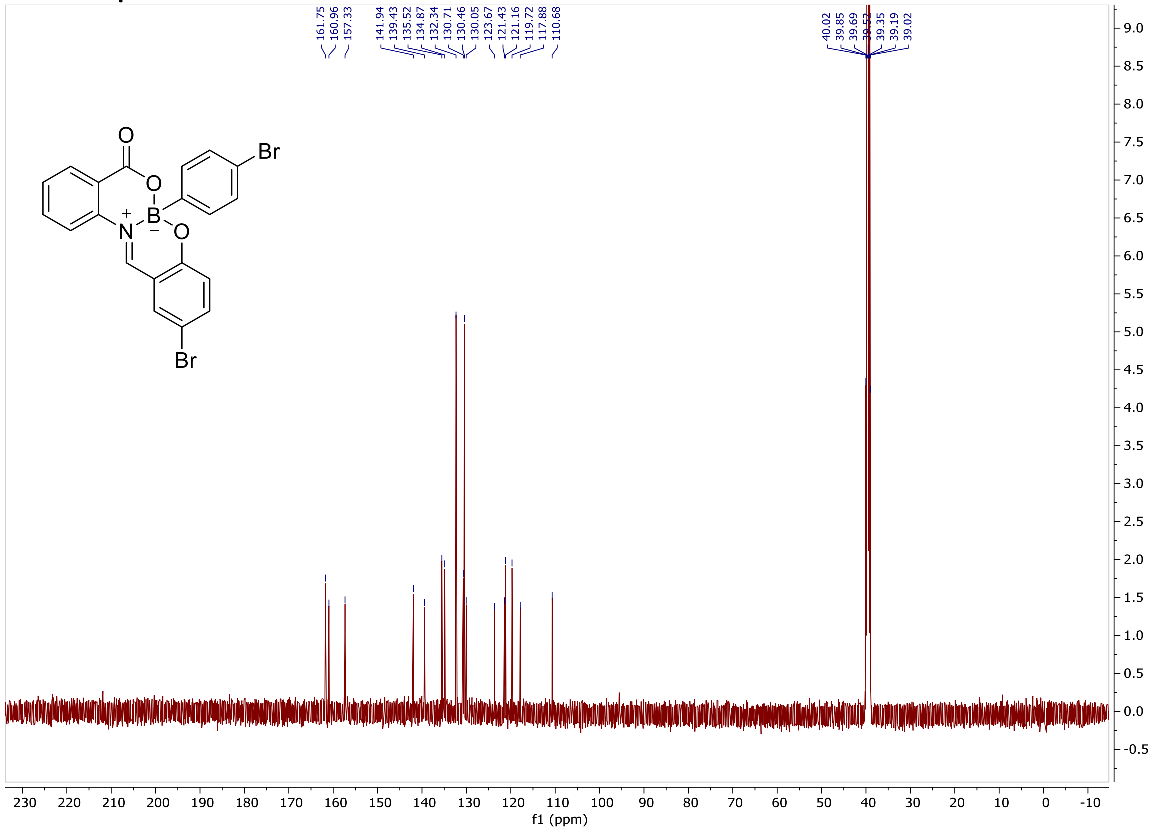

(41) HRMS

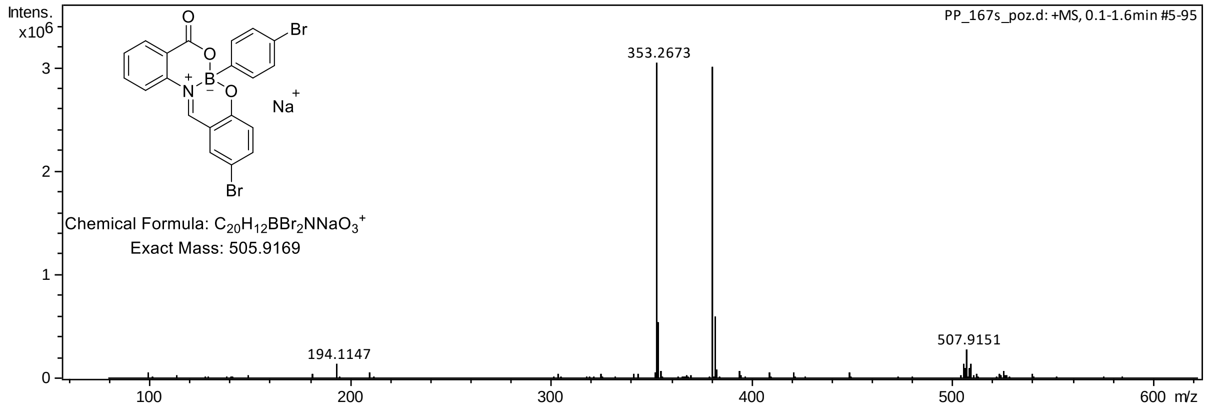

Intens.
x10^6
PP_167s_poz.d: +MS, 0.1-1.6min #5-95
O
O
Br
B
N
O
Na+
Br
Chemical Formula: C20H12BBr2NNaO3+
Exact Mass: 505.9169
353.2673
194.1147
507.9151
0
1
2
3
100
200
300
400
500
600 m/z

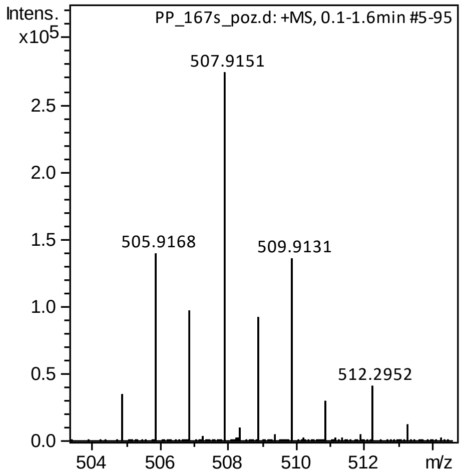

Intens.
x10^5
PP_167s_poz.d: +MS, 0.1-1.6min #5-95
507.9151
505.9168
509.9131
512.2952
0.0
0.5
1.0
1.5
2.0
2.5
504
506
508
510
512
m/z

11-bromo-7-(4-bromophenyl)-3-methyl-5H,7H-7λ4,14λ4-benzo[d]benzo[5,6][1,3,2] oxazaborinino[2,3-b][1,3,2]oxazaborinin-5-one **(42)** **^{1}H-NMR spectrum**

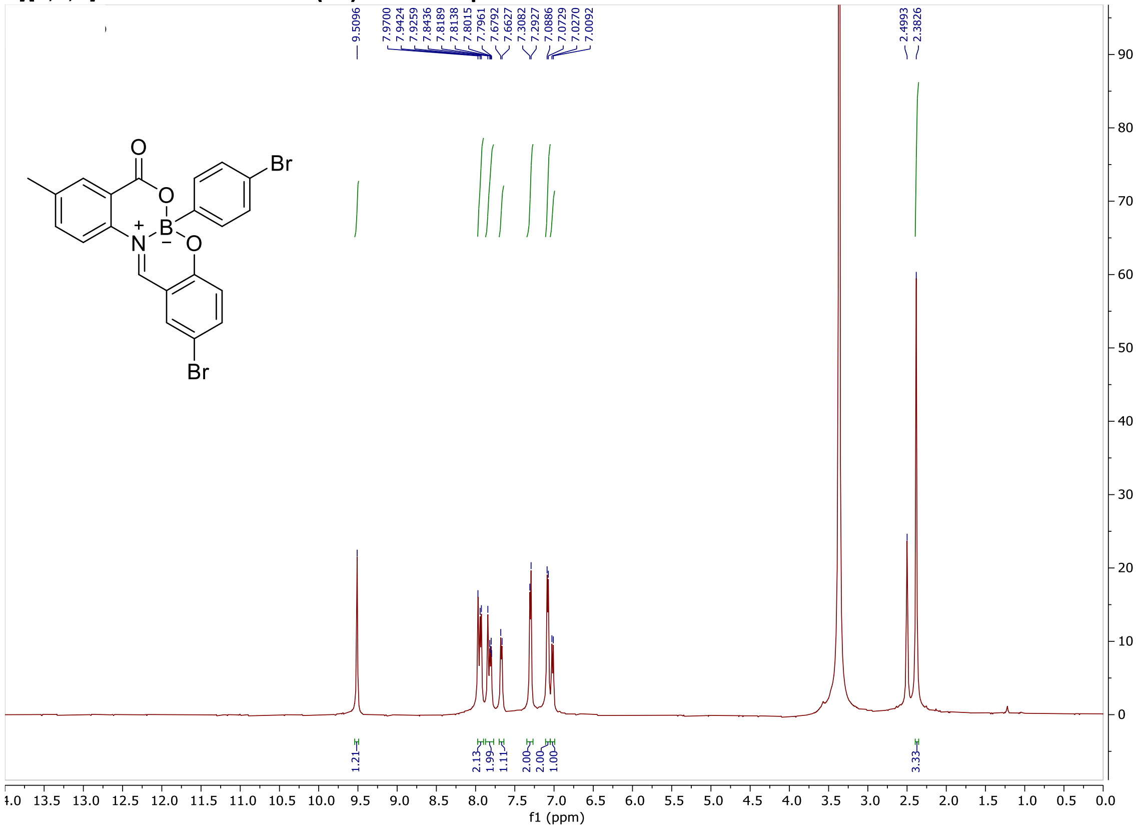

^{13}C-NMR spectrum

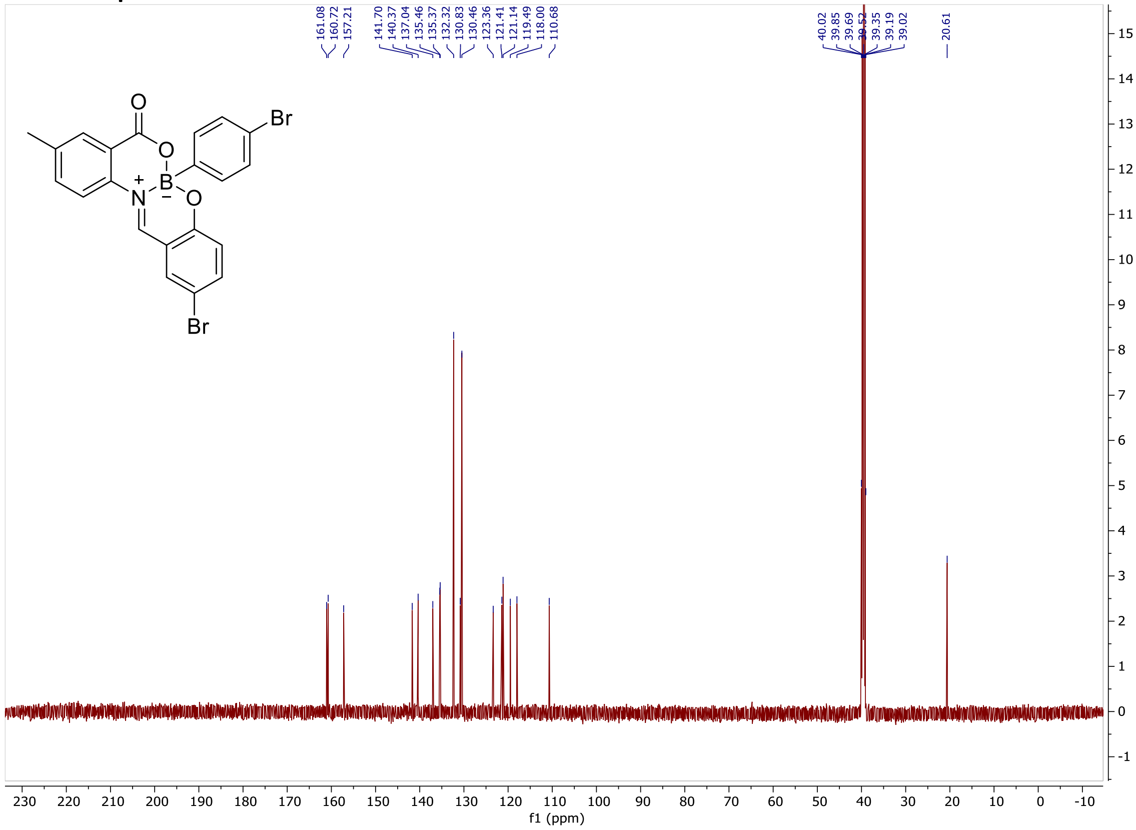

(42) HRMS

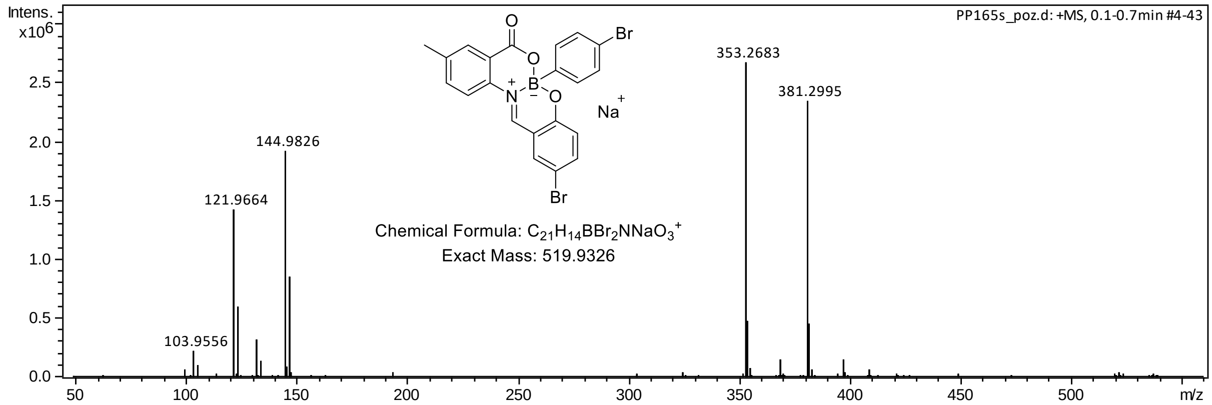

Intens.
x10^6
PP165s_poz.d: +MS, 0.1-0.7min #4-43
353.2683
381.2995
144.9826
121.9664
103.9556
Br
O
O
B
N
O
Na
Br
Chemical Formula: C21H14BBr2NNaO3+
Exact Mass: 519.9326
m/z

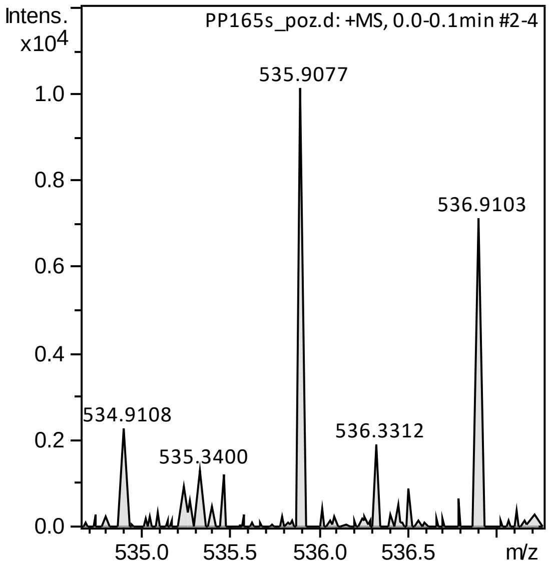

Intens.
x10^4
PP165s_poz.d: +MS, 0.0-0.1min #2-4
535.9077
536.9103
534.9108
536.3312
535.3400
m/z

11-bromo-7-(4-bromophenyl)-3-fluoro-5H,7H-7λ^4,14λ^4-benzo[d]benzo[5,6][1,3,2] oxazaborinino[2,3-b][1,3,2]oxazaborinin-5-one **(43)** **^{1}H-NMR spectrum**

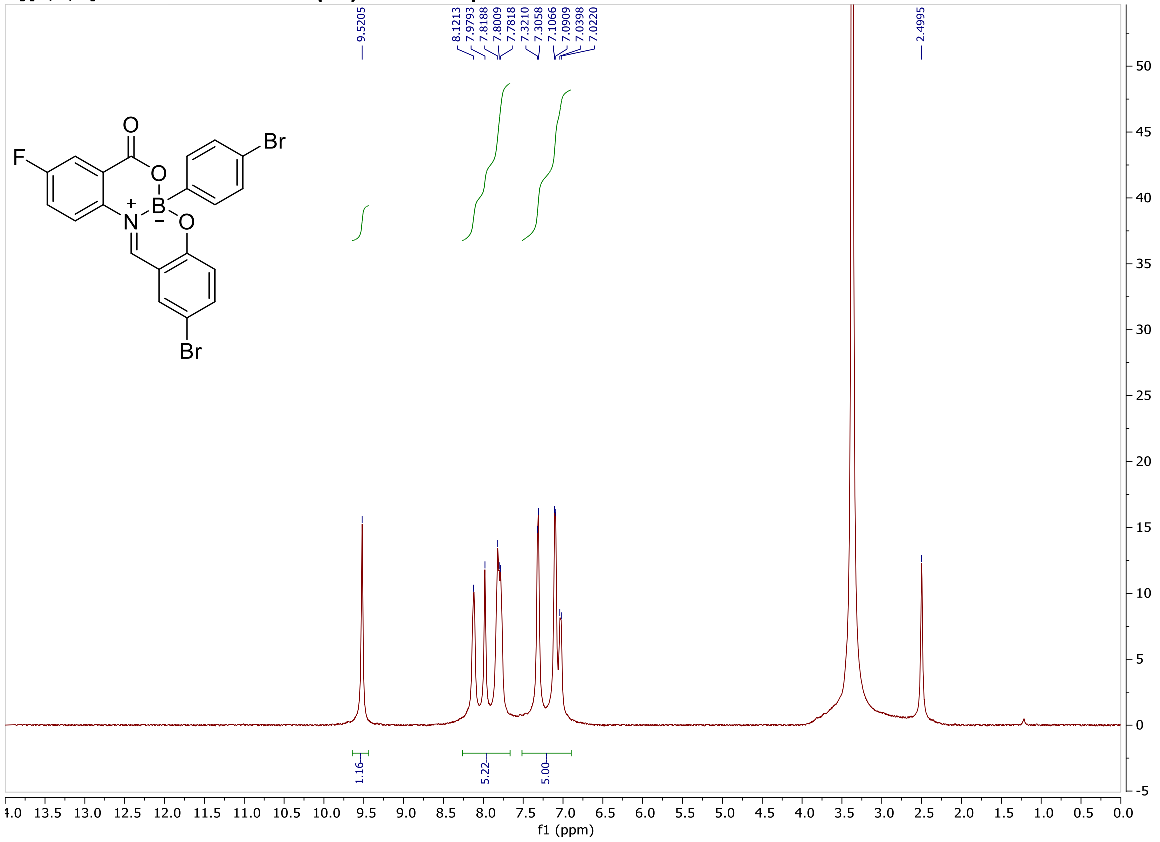

^{13}C-NMR spectrum

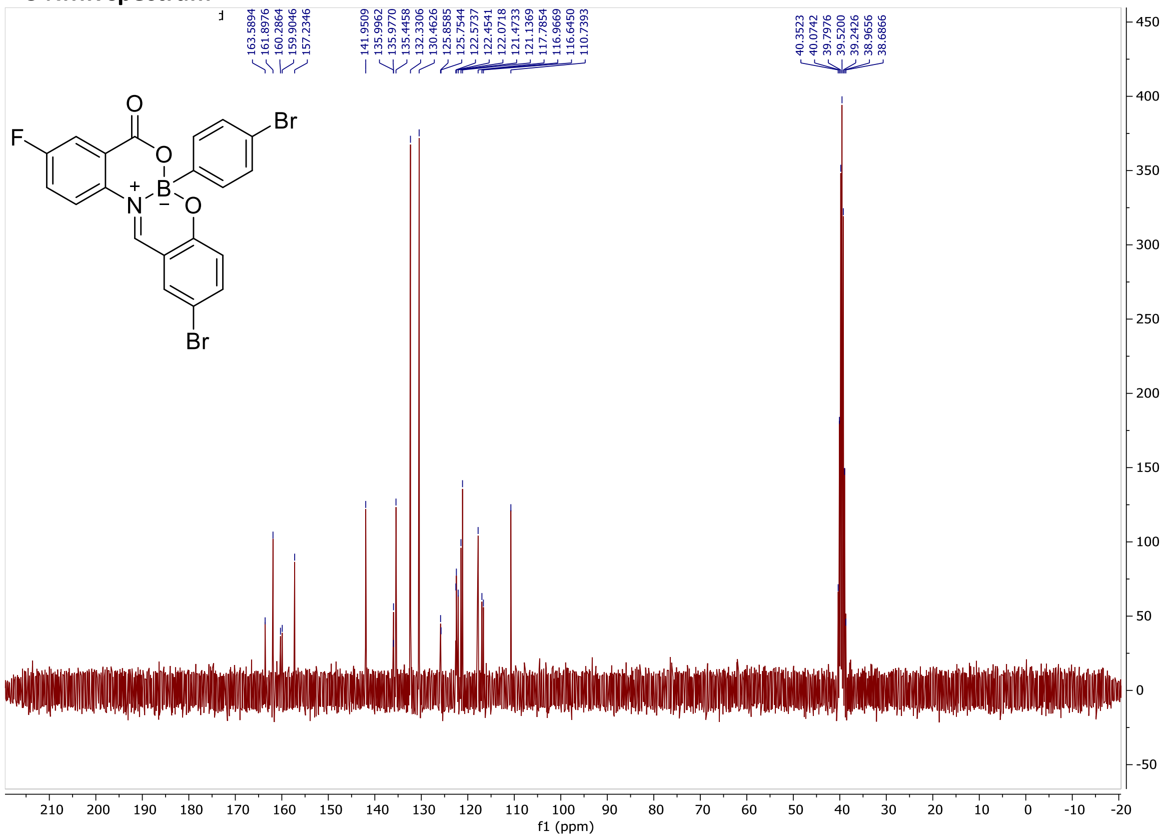

(43) HRMS

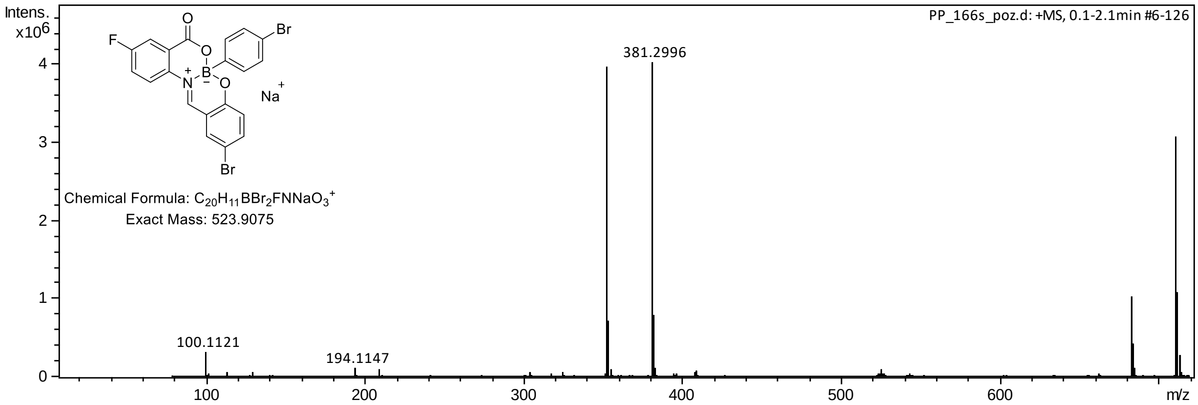

Intens.
x10^6
PP_166s_poz.d: +MS, 0.1-2.1min #6-126
Br
F
O
O
B
N
O
Na+
Br
381.2996
Chemical Formula: C20H11BBr2FNNaO3+
Exact Mass: 523.9075
100.1121
194.1147
100
200
300
400
500
600
m/z

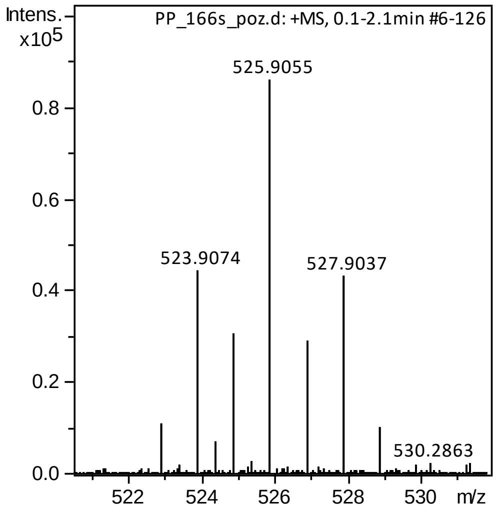

Intens.
x10^5
PP_166s_poz.d: +MS, 0.1-2.1min #6-126
525.9055
523.9074
527.9037
530.2863
522
524
526
528
530
m/z

11-bromo-7-(4-bromophenyl)-3-chloro-5H,7H-7λ[4],14λ[4]-benzo[d]benzo[5,6][1,3,2] oxazaborinino[2,3-b][1,3,2]oxazaborinin-5-one **(44)** **^{1}H-NMR spectrum**

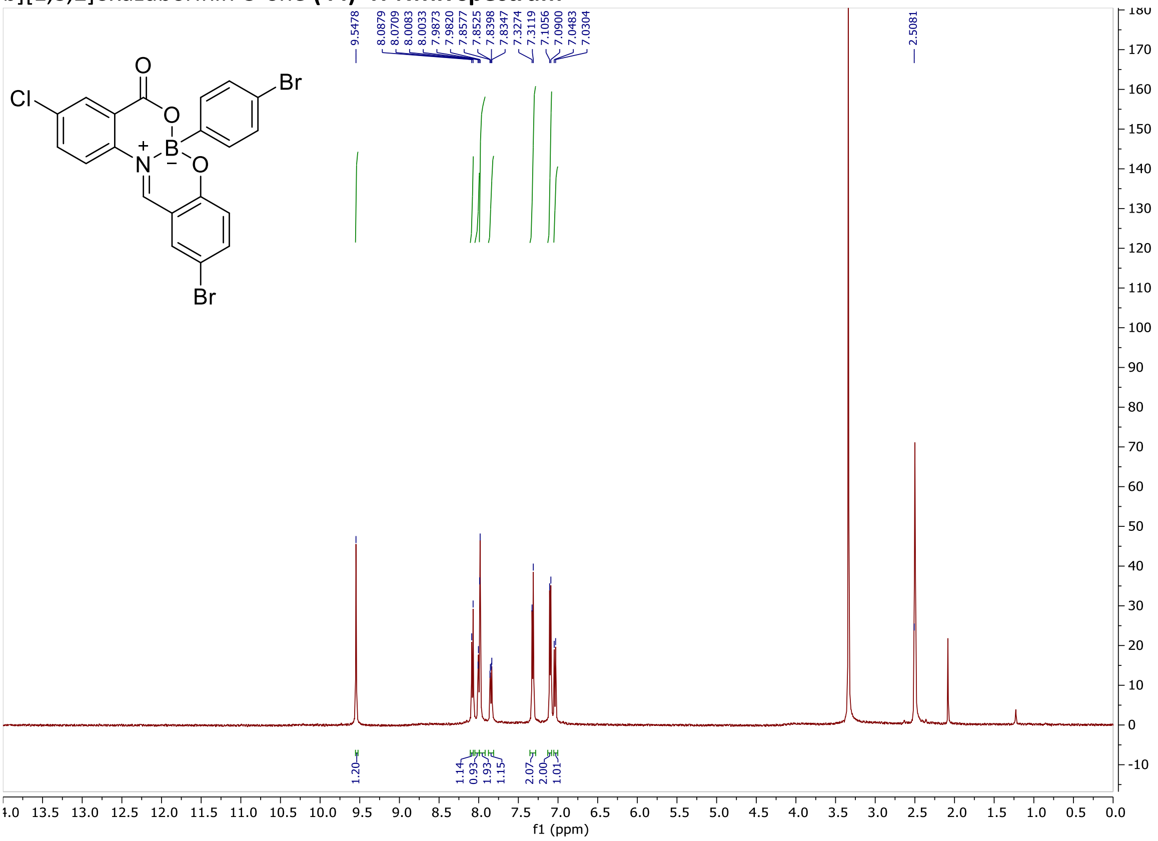

^{13}C-NMR spectrum

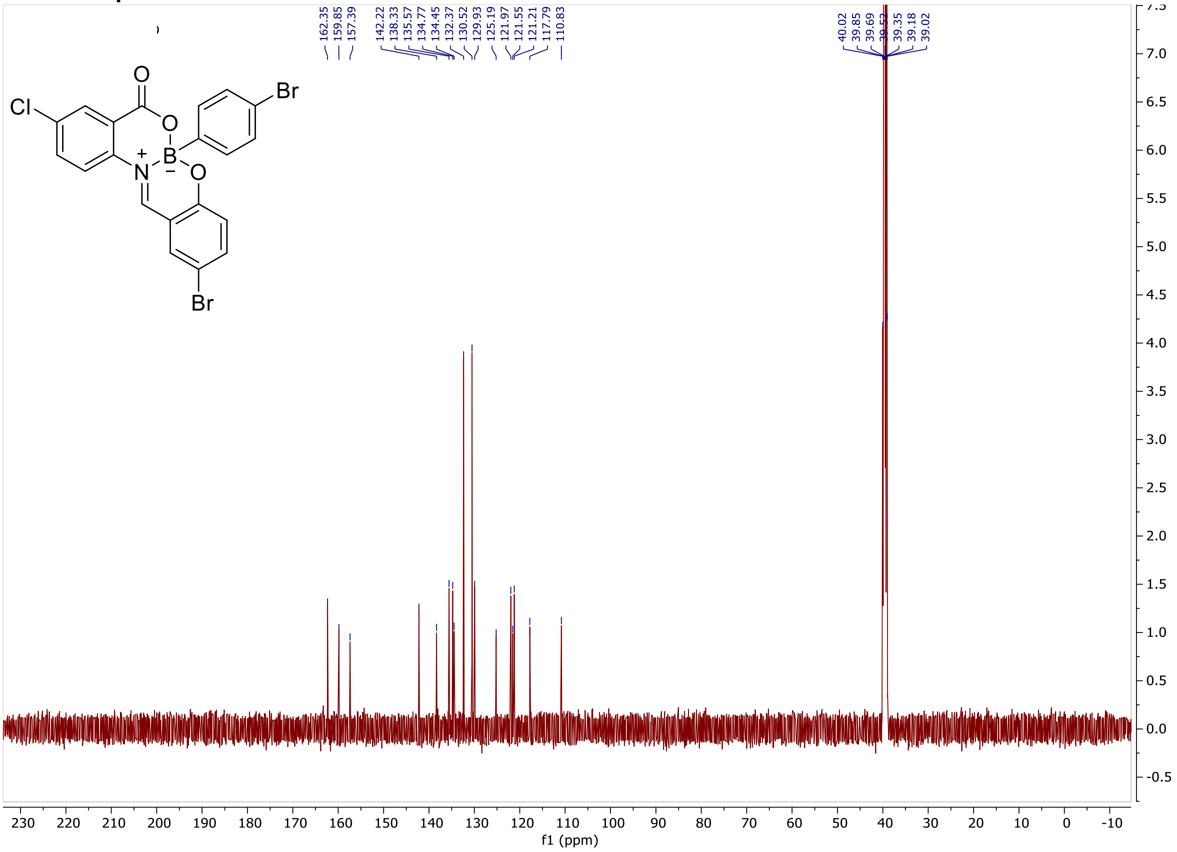

(44) HRMS

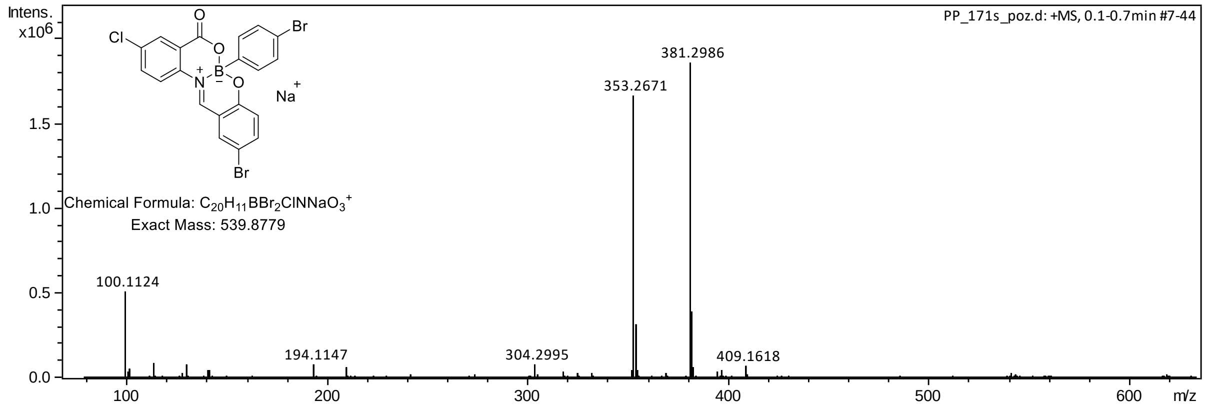
PP_171s_poz.d: +MS, 0.1-0.7min #7-44
Intens.
x10^6
Chemical Formula: C20H11BBr2ClNNaO3+
Exact Mass: 539.8779
100.1124
194.1147
304.2995
353.2671
381.2986
409.1618
m/z

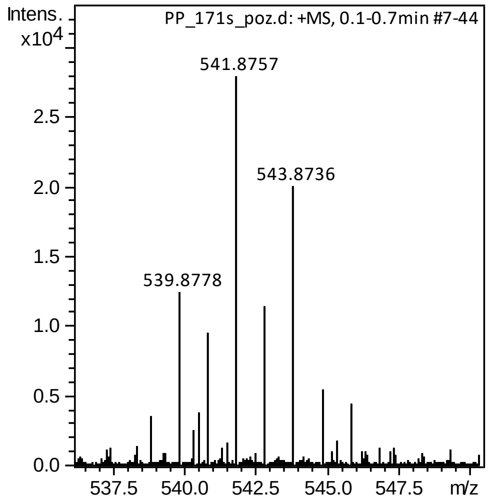
PP_171s_poz.d: +MS, 0.1-0.7min #7-44
Intens.
x10^4
539.8778
541.8757
543.8736
m/z

3,11-dibromo-7-(4-bromophenyl)-5H,7H-7λ[4],14λ[4]-benzo[d]benzo[5,6][1,3,2] oxazaborinino[2,3-b][1,3,2]oxazaborinin-5-one **(45)** **^{1}H-NMR spectrum**

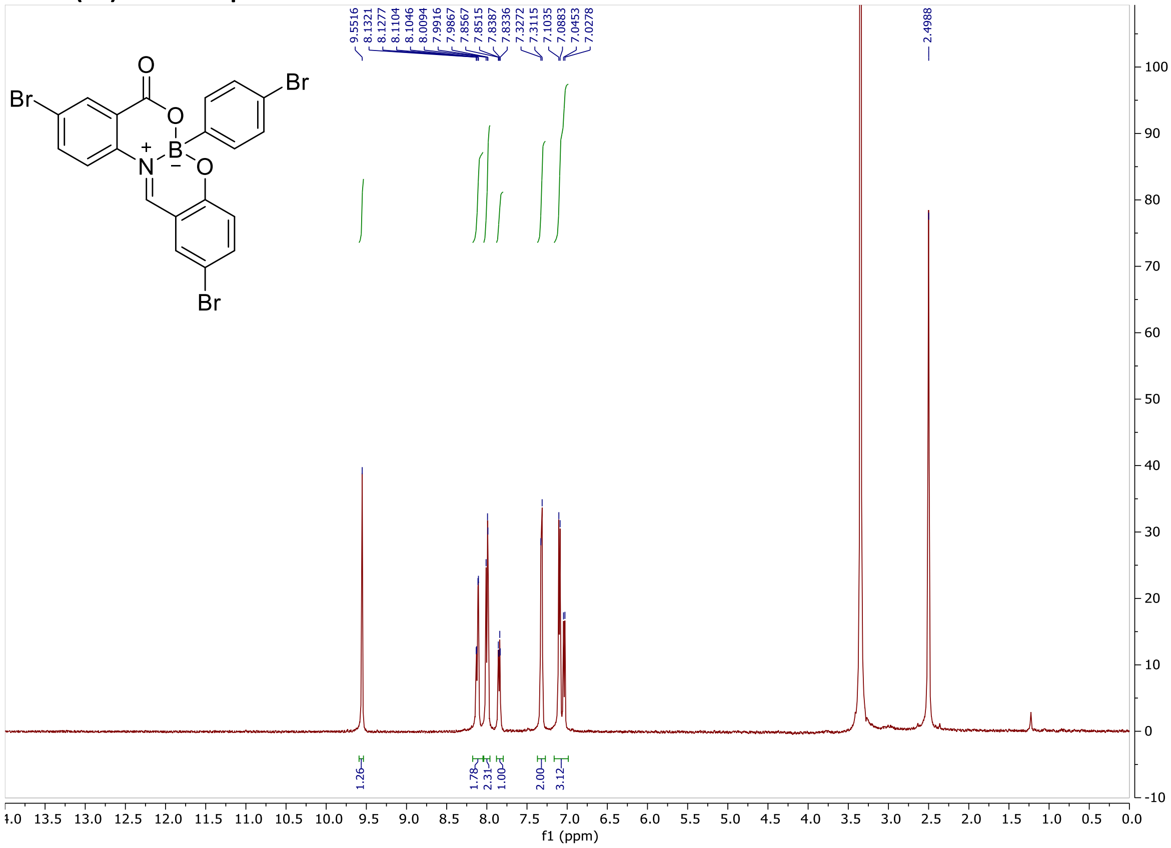

^{13}C-NMR spectrum

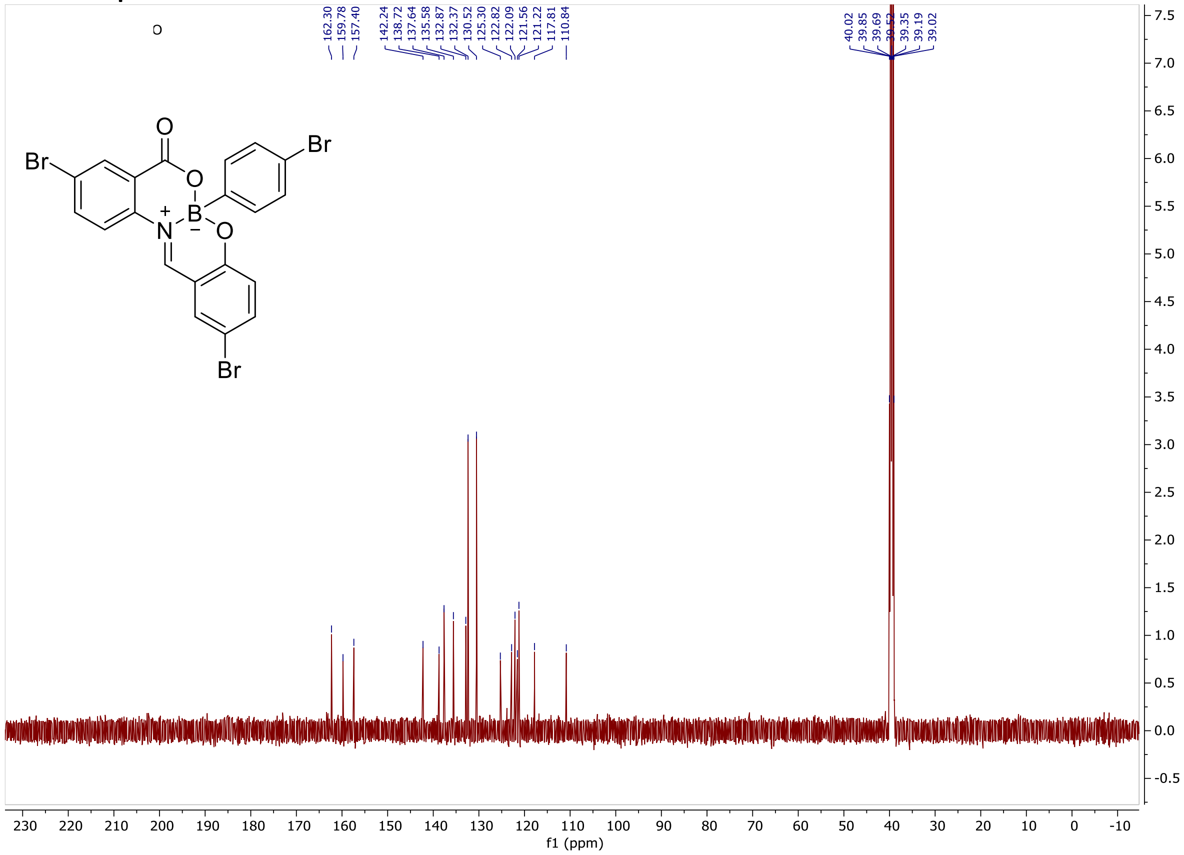

(45) HRMS

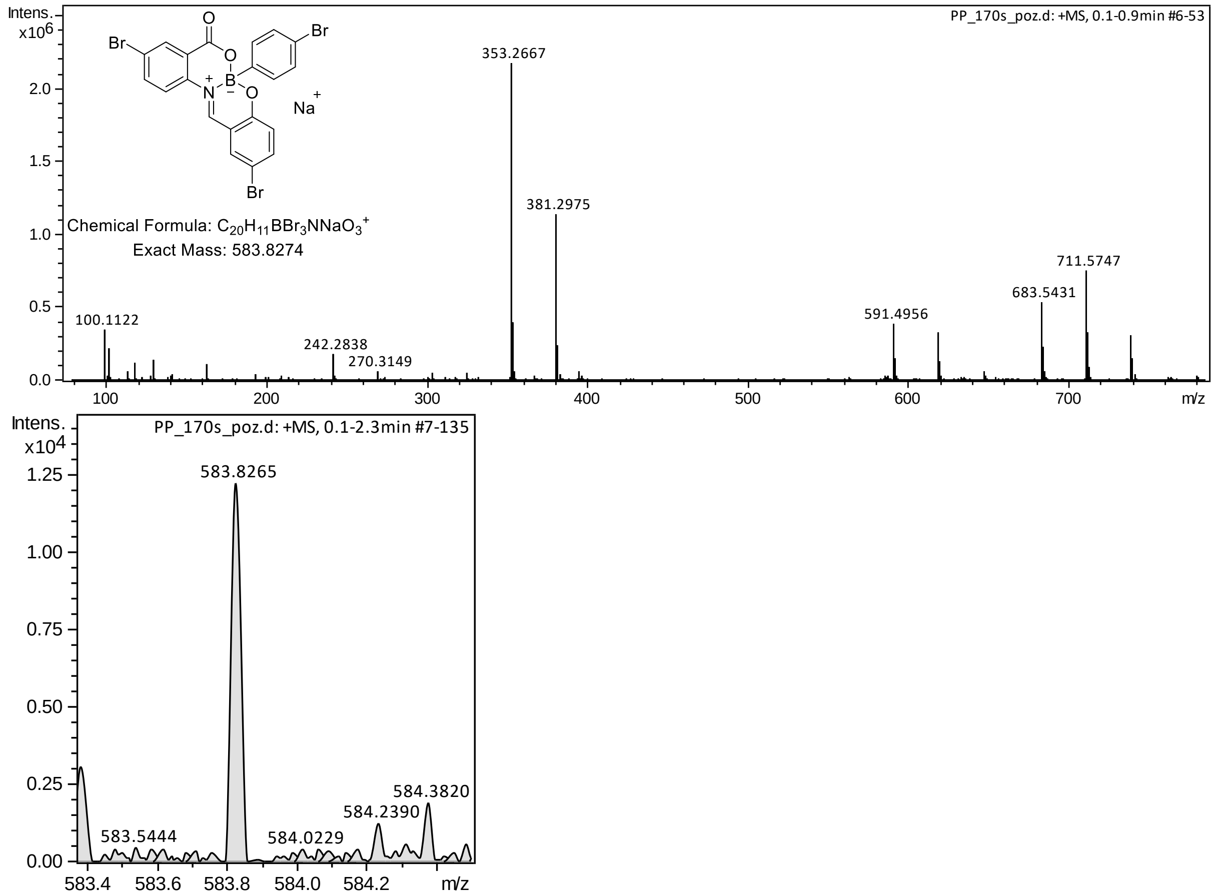
Intens.
x10^6
PP_170s_poz.d: +MS, 0.1-0.9min #6-53
Chemical Formula: C20H11BBr3NNaO3+
Exact Mass: 583.8274
353.2667
381.2975
711.5747
683.5431
591.4956
100.1122
242.2838
270.3149
m/z
Intens.
x10^4
PP_170s_poz.d: +MS, 0.1-2.3min #7-135
583.8265
584.3820
584.2390
583.5444
584.0229
m/z

11-bromo-7-(4-bromophenyl)-3-iodo-5H,7H-7λ4,14λ4-benzo[d]benzo[5,6][1,3,2] oxazaborinino[2,3-b][1,3,2]oxazaborinin-5-one **(46)** **^{1}H-NMR spectrum**

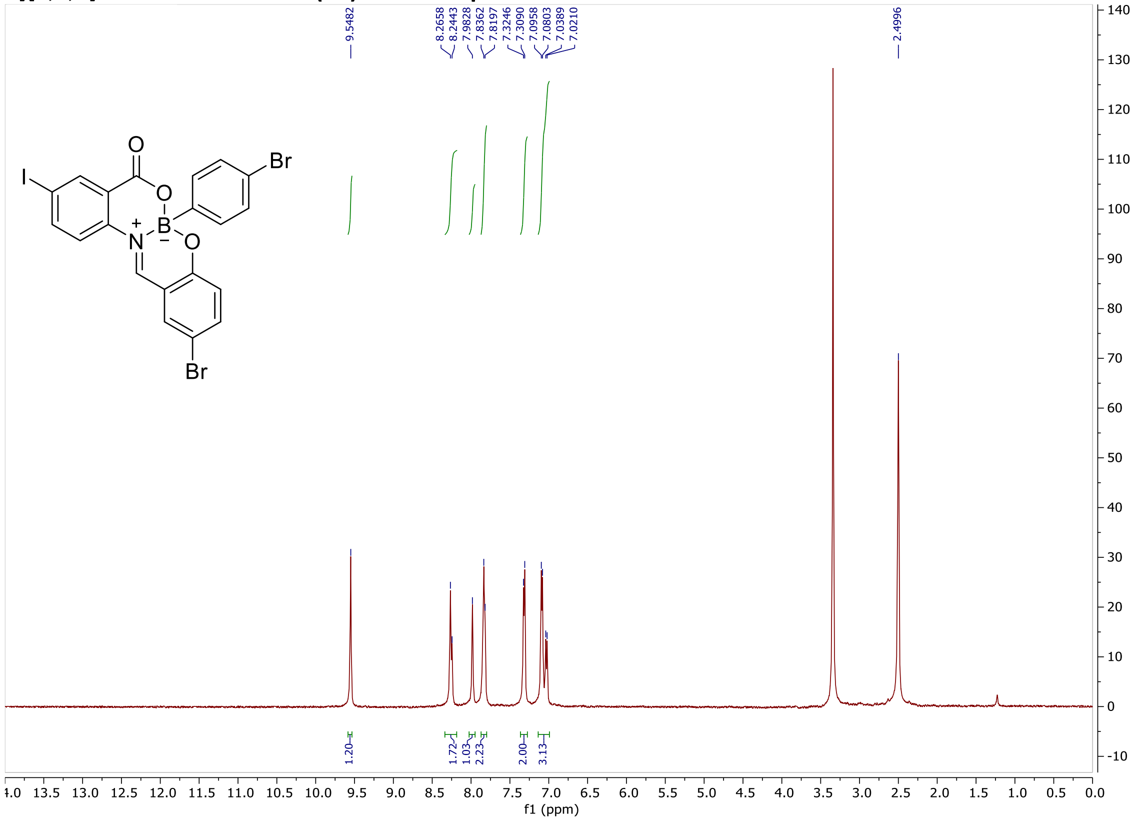

^{13}C-NMR spectrum

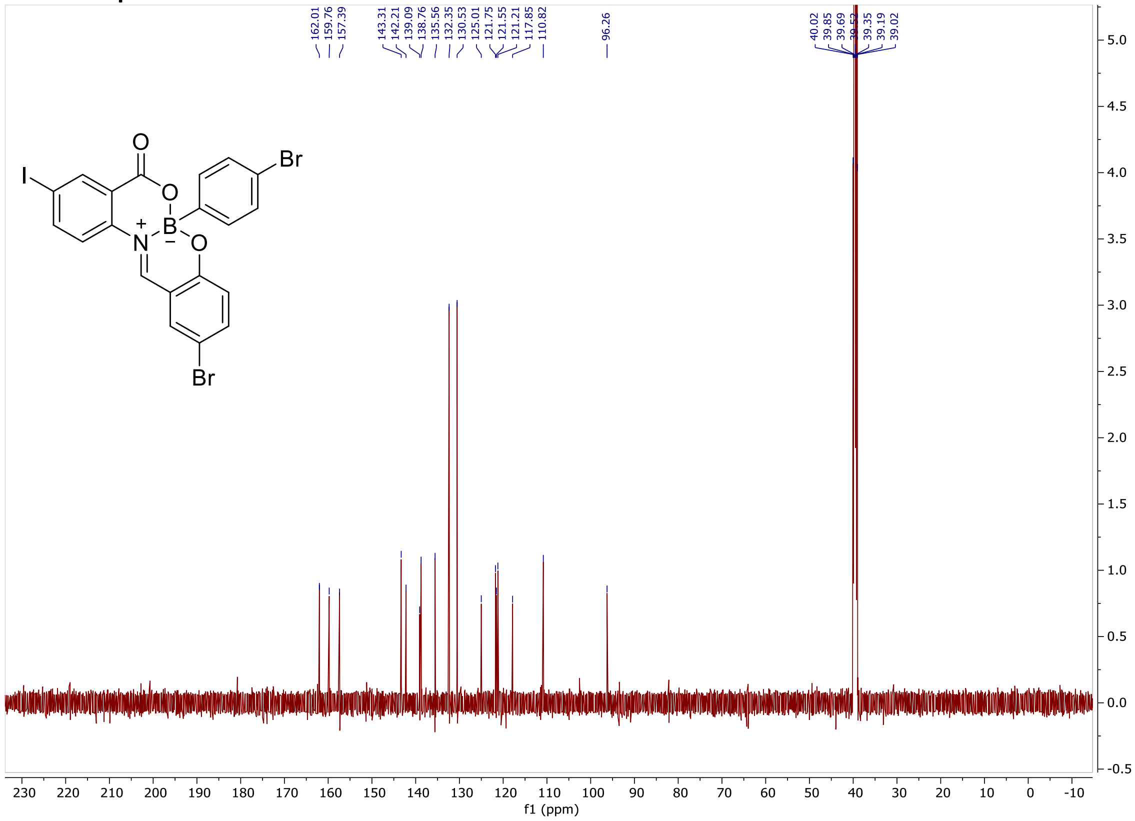

(46) HRMS

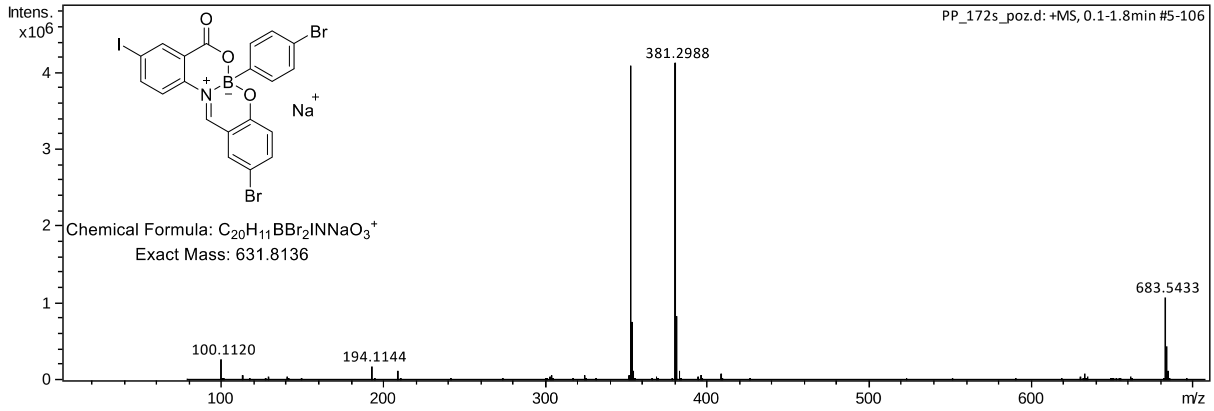

Intens.
x10^6
PP_172s_poz.d: +MS, 0.1-1.8min #5-106
381.2988
683.5433
100.1120
194.1144
Chemical Formula: C20H11BBr2INNaO3+
Exact Mass: 631.8136
m/z

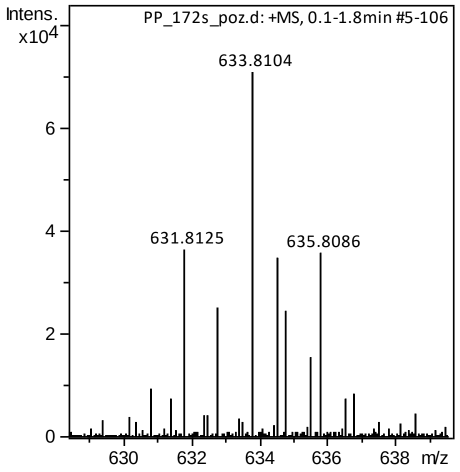

Intens.
x10^4
PP_172s_poz.d: +MS, 0.1-1.8min #5-106
633.8104
631.8125
635.8086
m/z

11-bromo-7-(4-bromophenyl)-2-chloro-5H,7H-7λ[4],14λ[4]-benzo[d]benzo[5,6][1,3,2] oxazaborinino[2,3-b][1,3,2]oxazaborinin-5-one (**47**) **^{1}H-NMR spectrum**

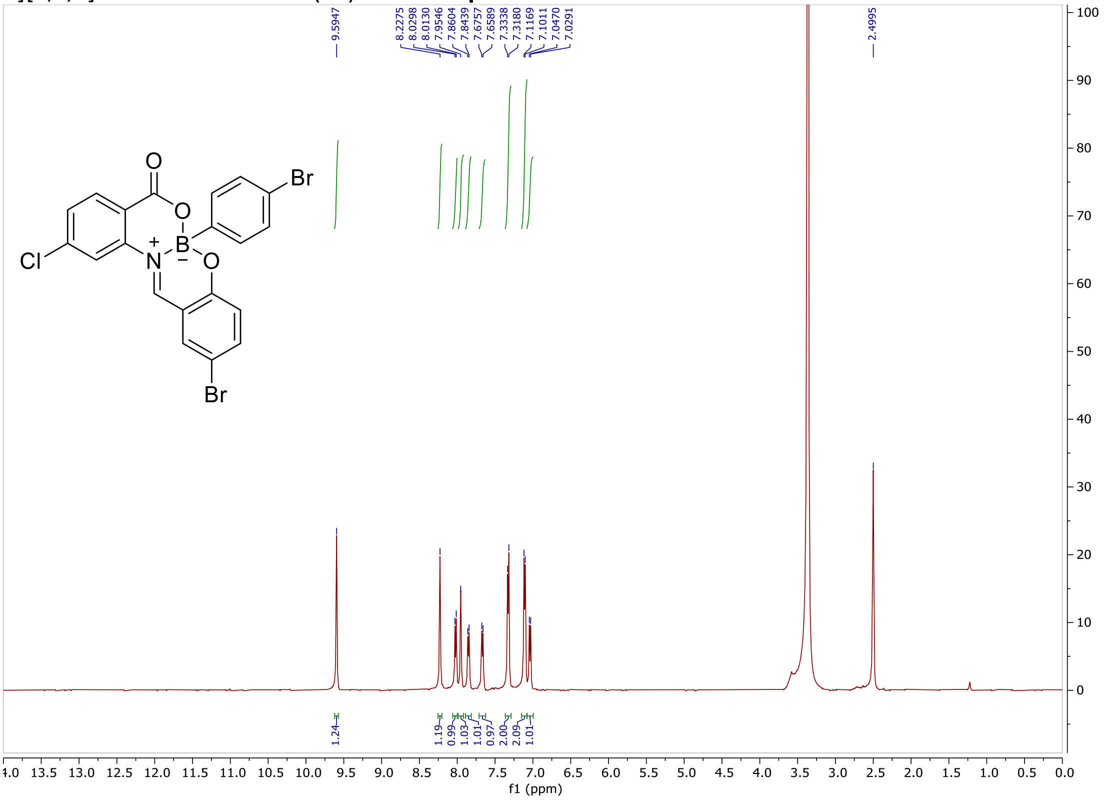

^{13}C-NMR spectrum

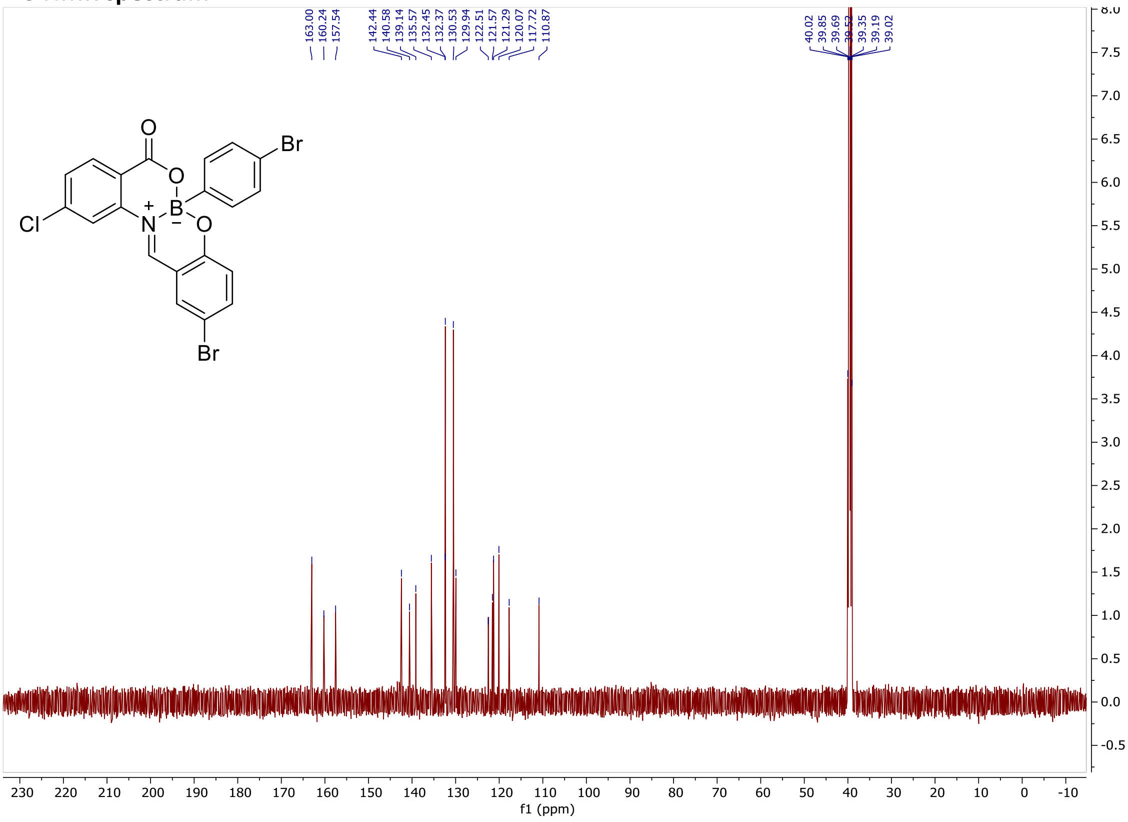

(47) HRMS

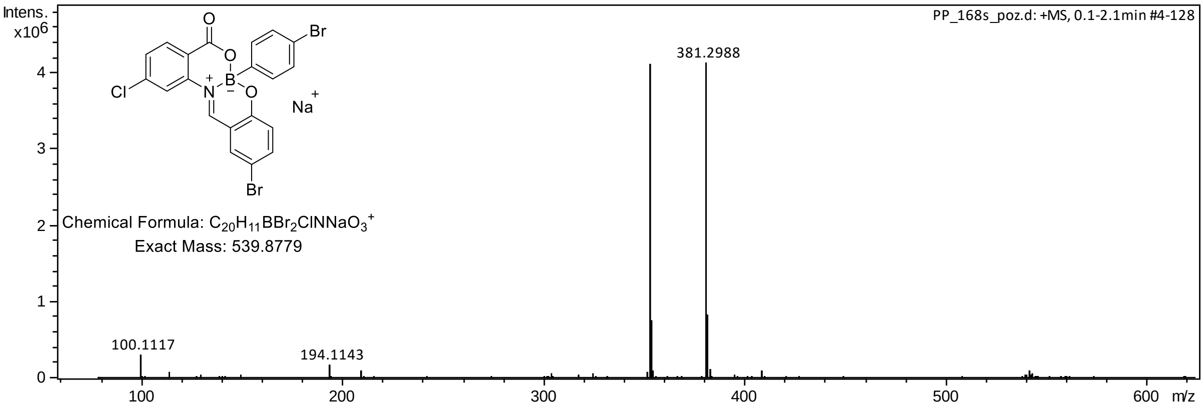
Intens.
x10^6
PP_168s_poz.d: +MS, 0.1-2.1min #4-128
381.2988
100.1117
194.1143
Cl
O
O
B
N
O
Br
Na+
Br
Chemical Formula: $C_{20}H_{11}BBr_2ClNNaO_3^+$
Exact Mass: 539.8779
m/z

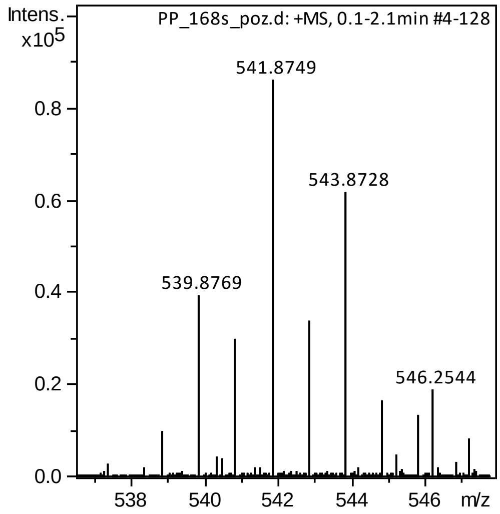
Intens.
x10^5
PP_168s_poz.d: +MS, 0.1-2.1min #4-128
541.8749
543.8728
539.8769
546.2544
m/z

11-bromo-7-(4-bromophenyl)-2-nitro-5H,7H-7λ[4],14λ[4]-benzo[d]benzo[5,6][1,3,2] oxazaborinino[2,3-b][1,3,2]oxazaborinin-5-one **(48)** **^{1}H-NMR spectrum**

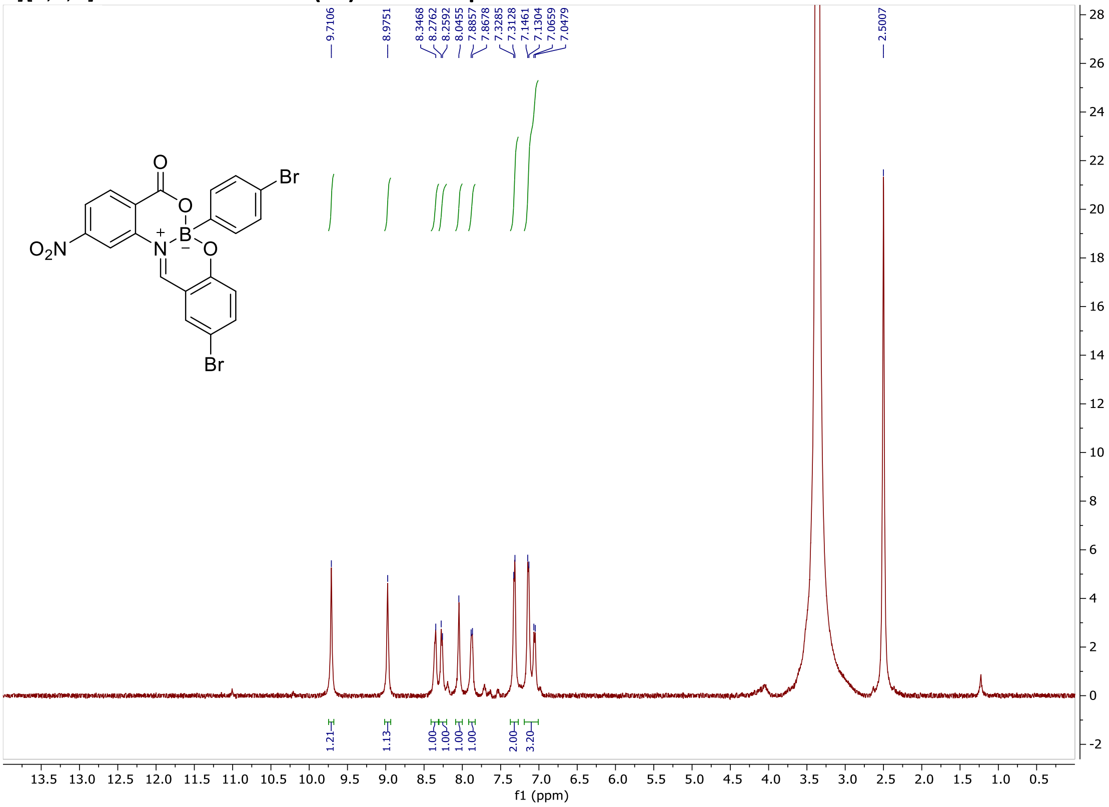

^{13}C-NMR spectrum

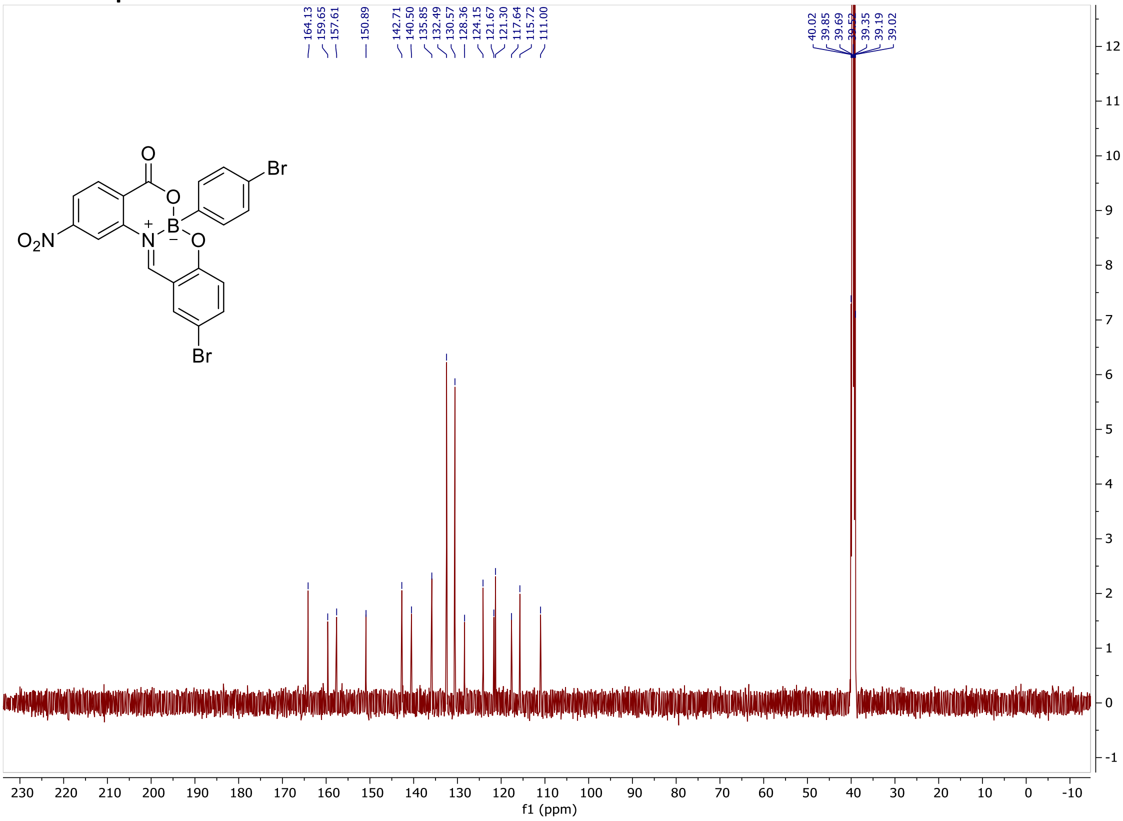

(48) HRMS

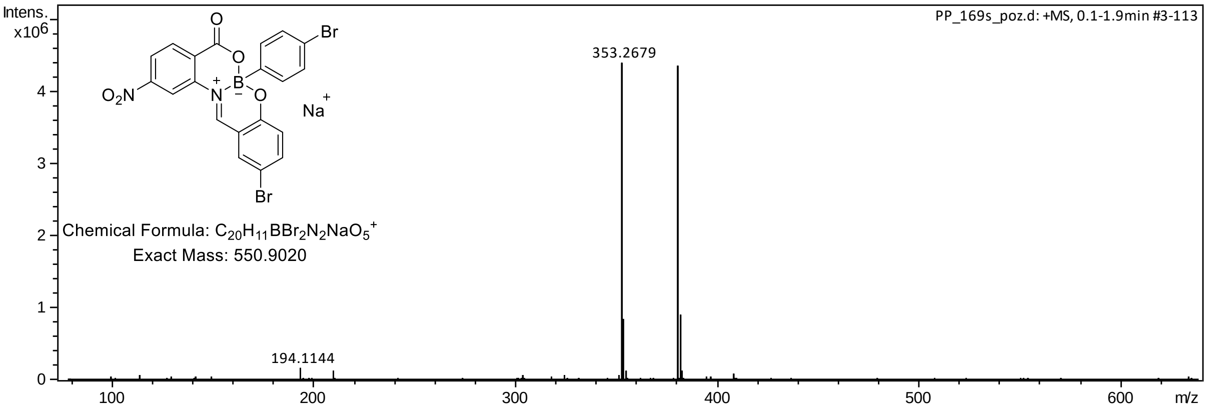

PP_169s_poz.d: +MS, 0.1-1.9min #3-113
Intens.
x10^6
Br
O
O
O₂N
N
B
O
Na+
Br
Chemical Formula: $C_{20}H_{11}BBr_2N_2NaO_5^+$
Exact Mass: 550.9020
353.2679
194.1144
100
200
300
400
500
600
m/z

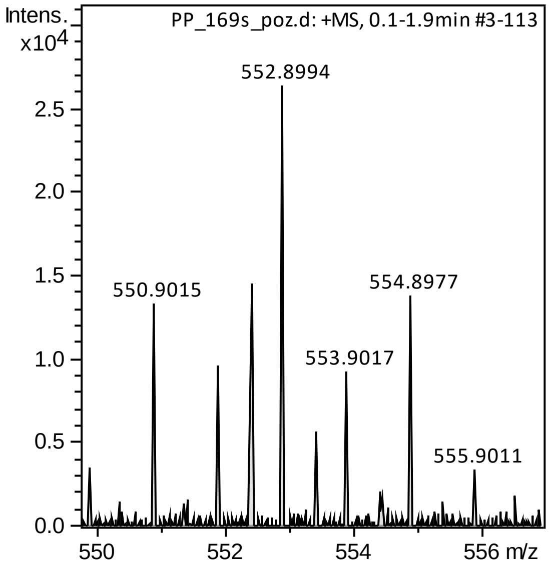

Intens.
x10^4
PP_169s_poz.d: +MS, 0.1-1.9min #3-113
552.8994
550.9015
554.8977
553.9017
555.9011
550
552
554
556 m/z

S.2. Copies of NMR spectra of compounds 18-48 at 0, 12, 24, 48 and 72 h intervals

18

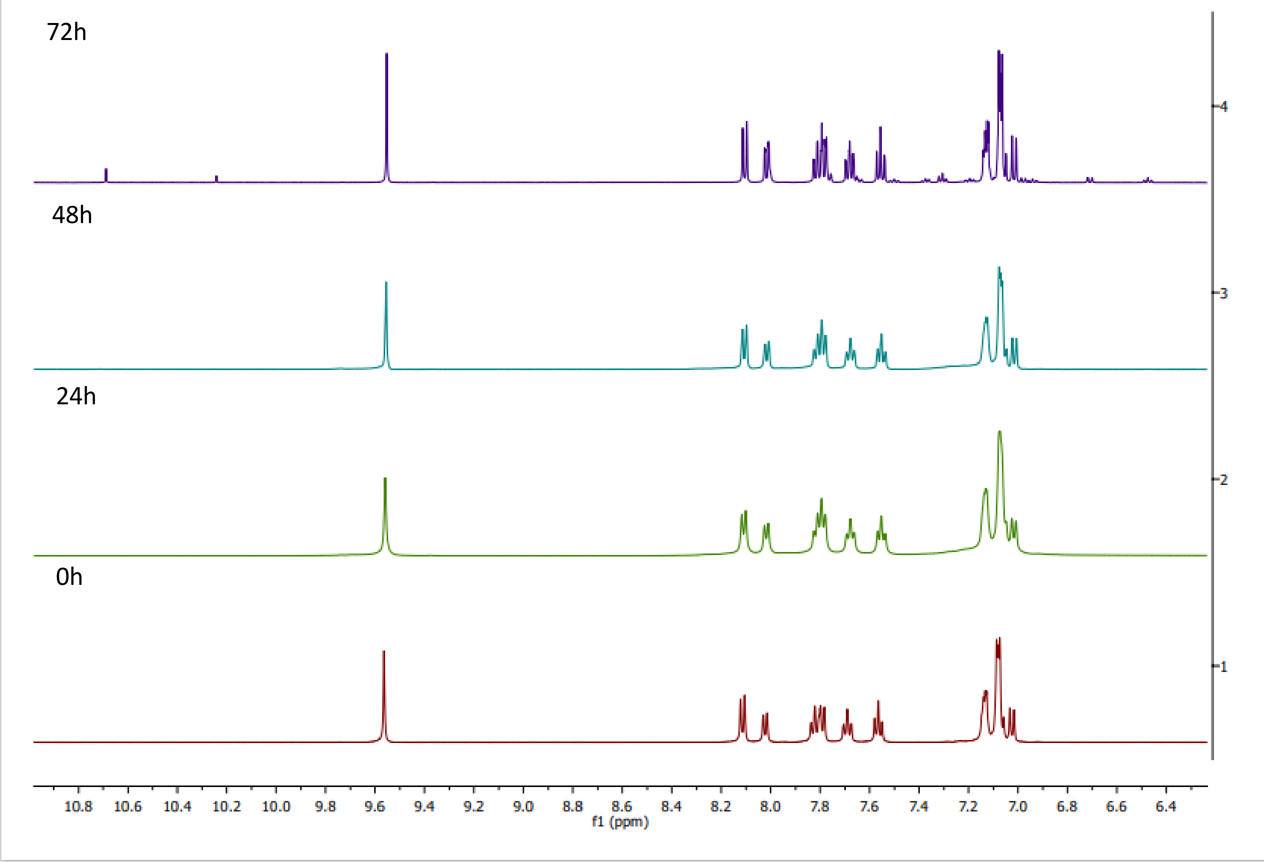

19

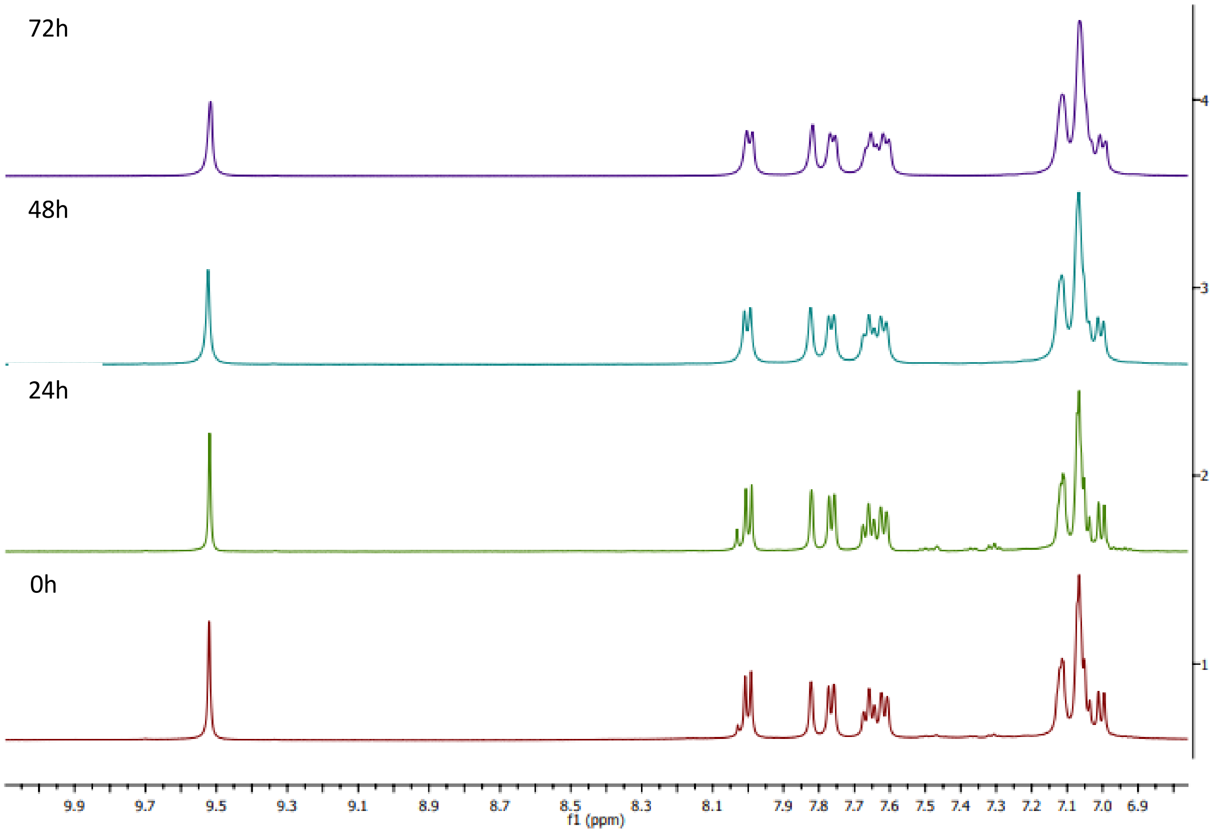

19

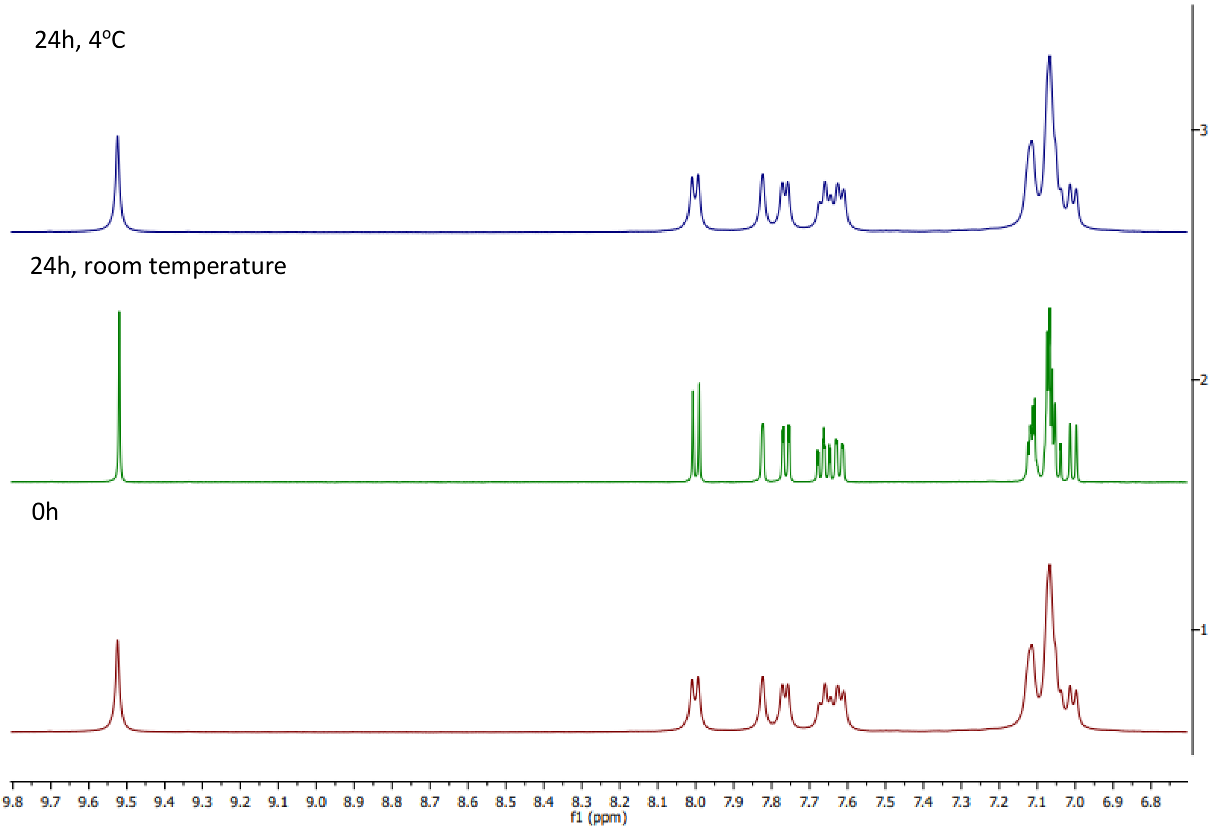
24h, 4ºC
24h, room temperature
0h
9.8 9.7 9.6 9.5 9.4 9.3 9.2 9.1 9.0 8.9 8.8 8.7 8.6 8.5 8.4 8.3 8.2 8.1 8.0 7.9 7.8 7.7 7.6 7.5 7.4 7.3 7.2 7.1 7.0 6.9 6.8
f1 (ppm)

20

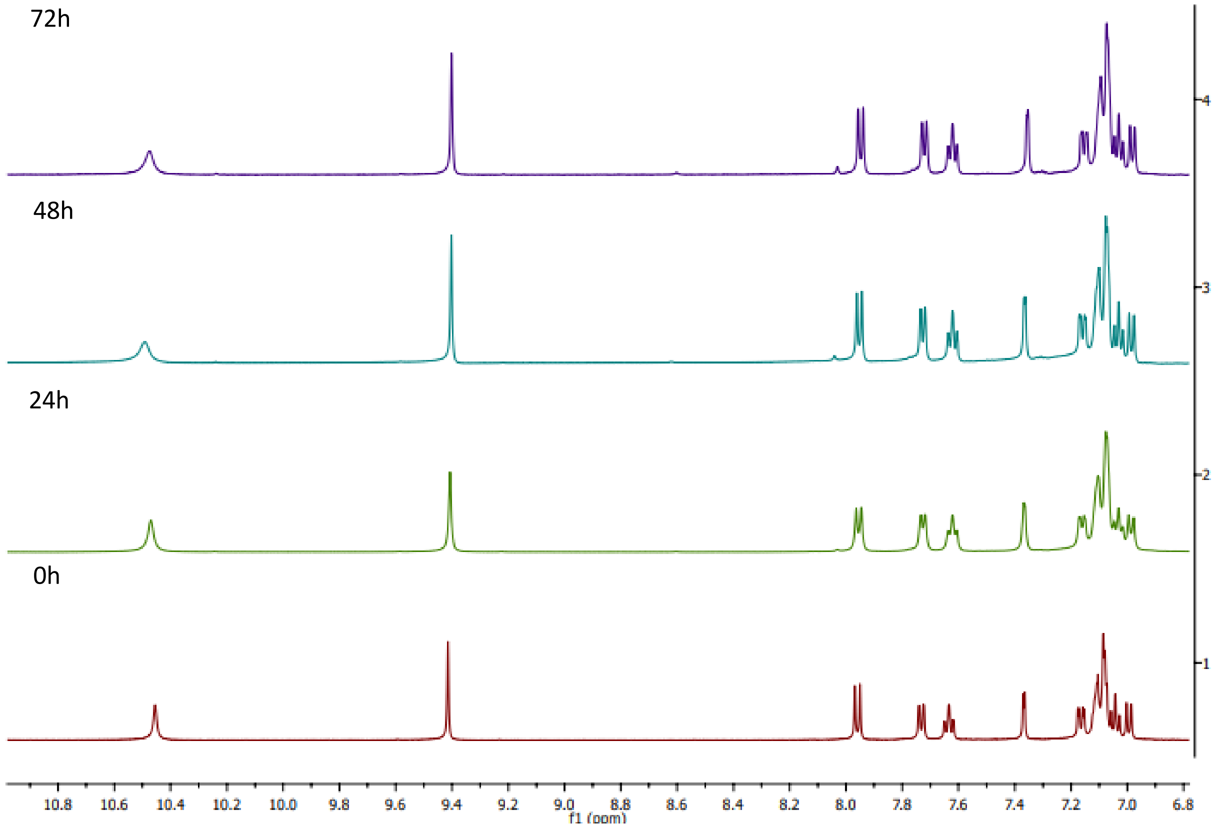
72h
48h
24h
0h
10.8 10.6 10.4 10.2 10.0 9.8 9.6 9.4 9.2 9.0 8.8 8.6 8.4 8.2 8.0 7.8 7.6 7.4 7.2 7.0 6.8
f1 (ppm)

21

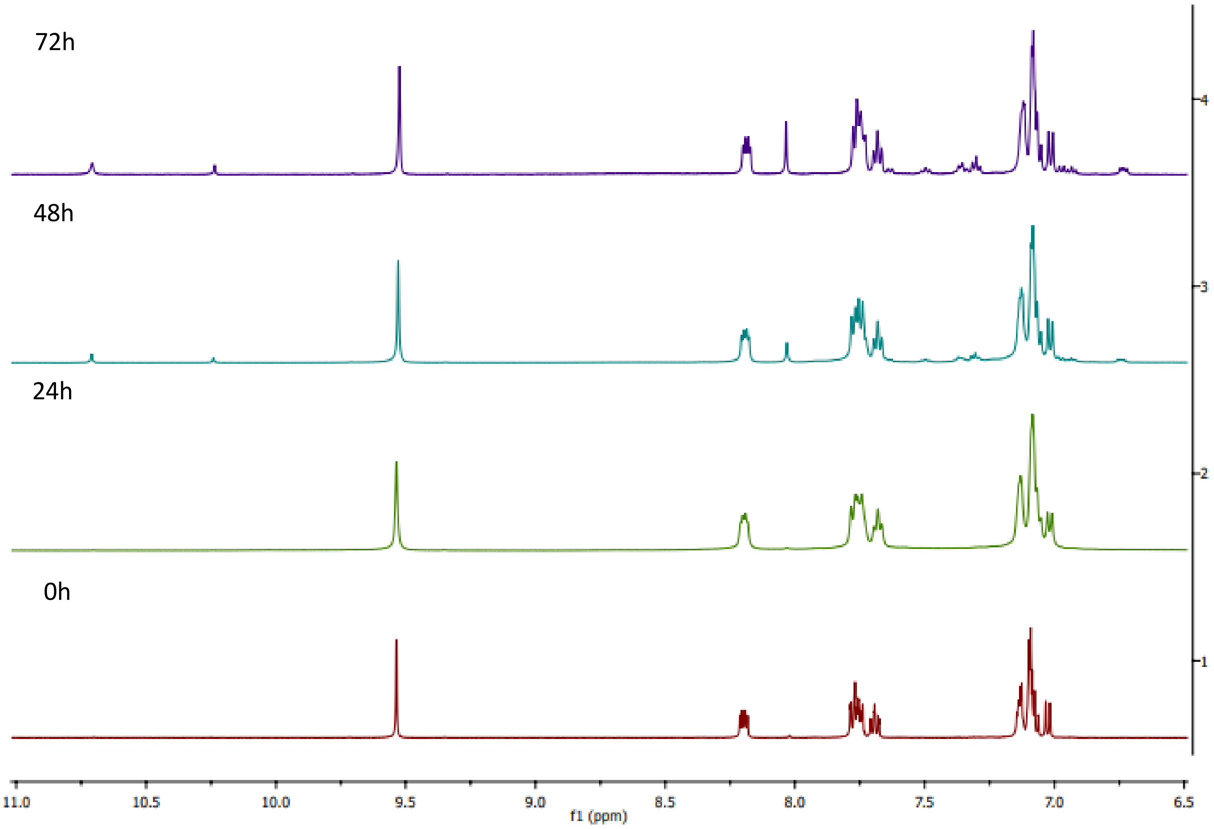
72h
48h
24h
0h
11.0
10.5
10.0
9.5
9.0
8.5
8.0
7.5
7.0
6.5
f1 (ppm)
4
3
2
1

22

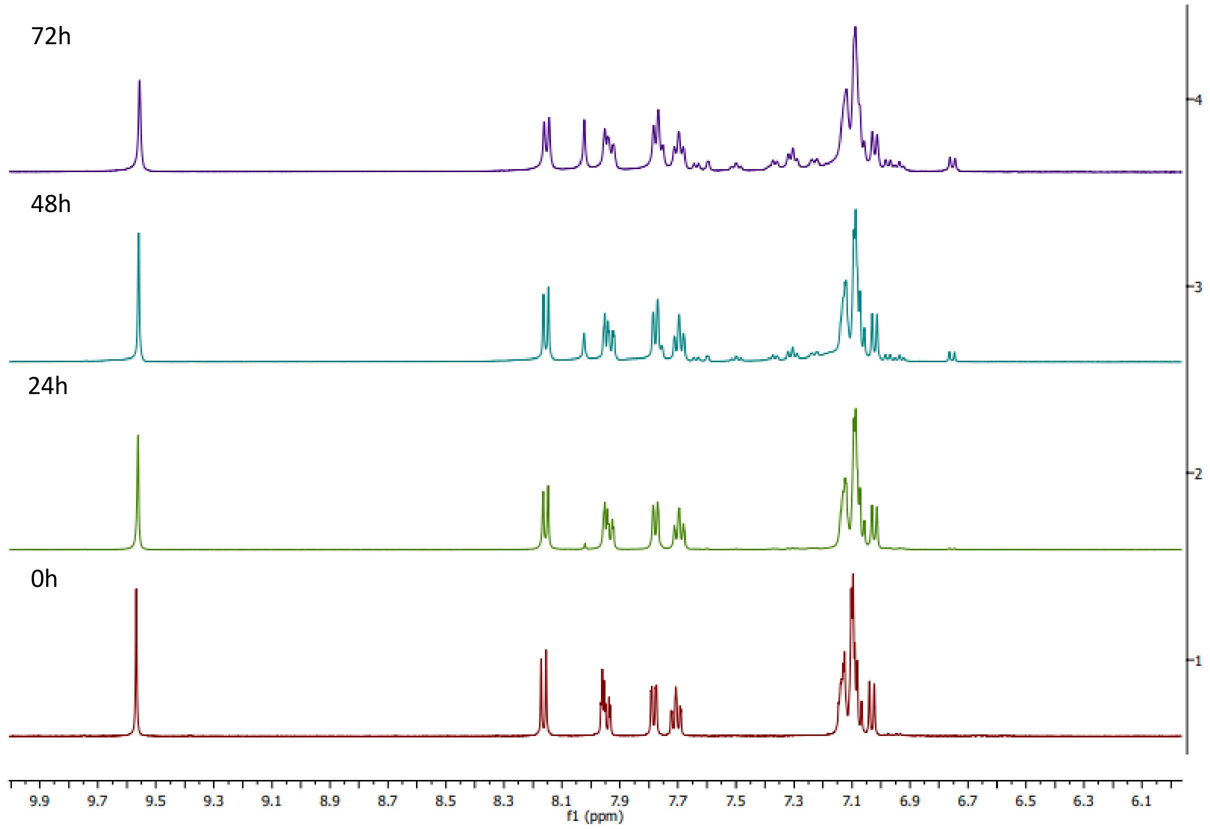
72h
48h
24h
0h
9.9
9.7
9.5
9.3
9.1
8.9
8.7
8.5
8.3
8.1
7.9
7.7
7.5
7.3
7.1
6.9
6.7
6.5
6.3
6.1
f1 (ppm)
4
3
2
1

23

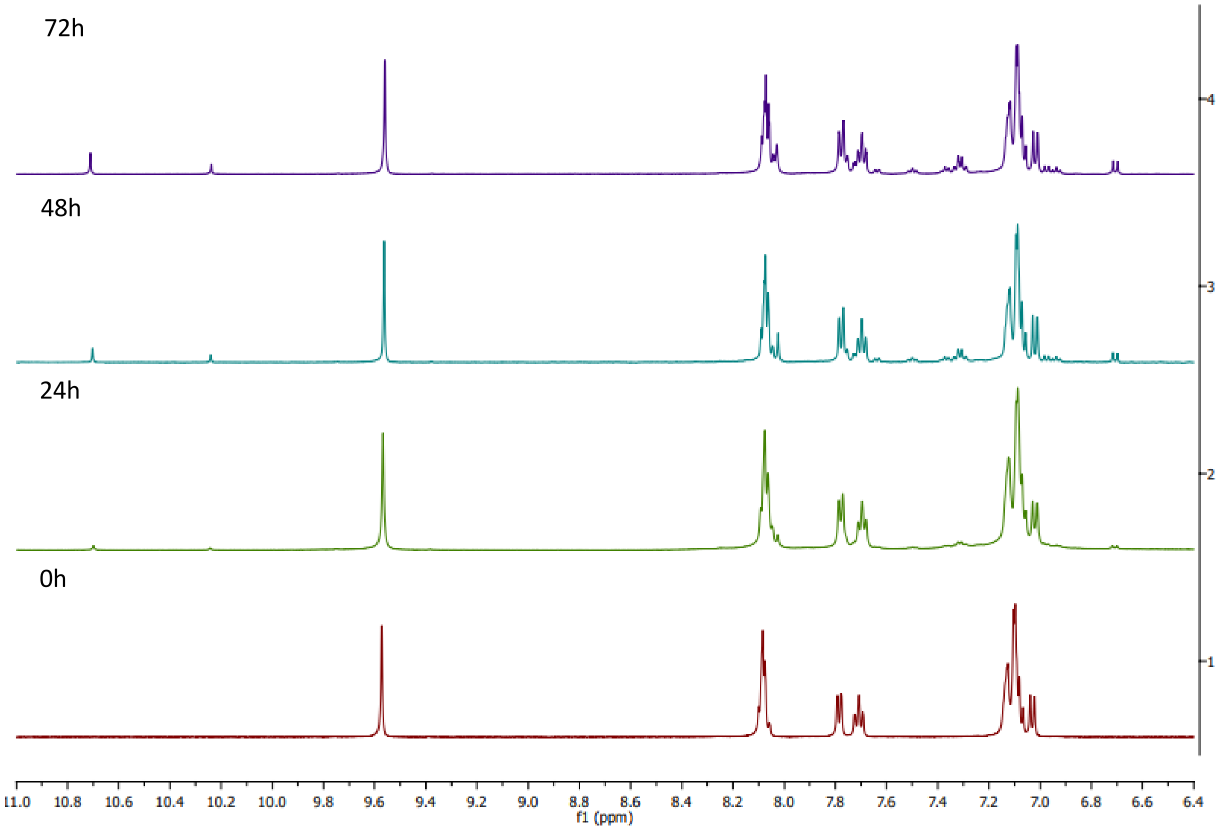

23 heat for 2 and 5 h

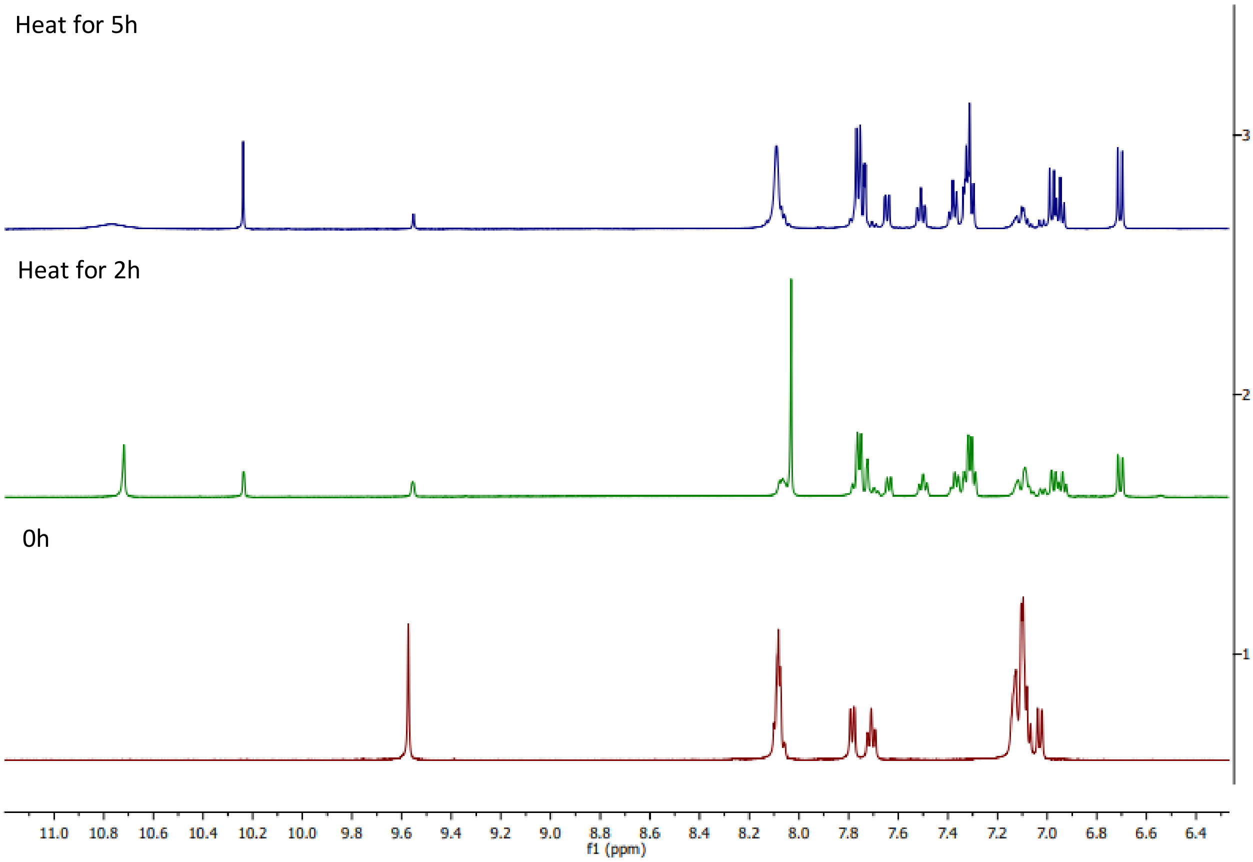

24

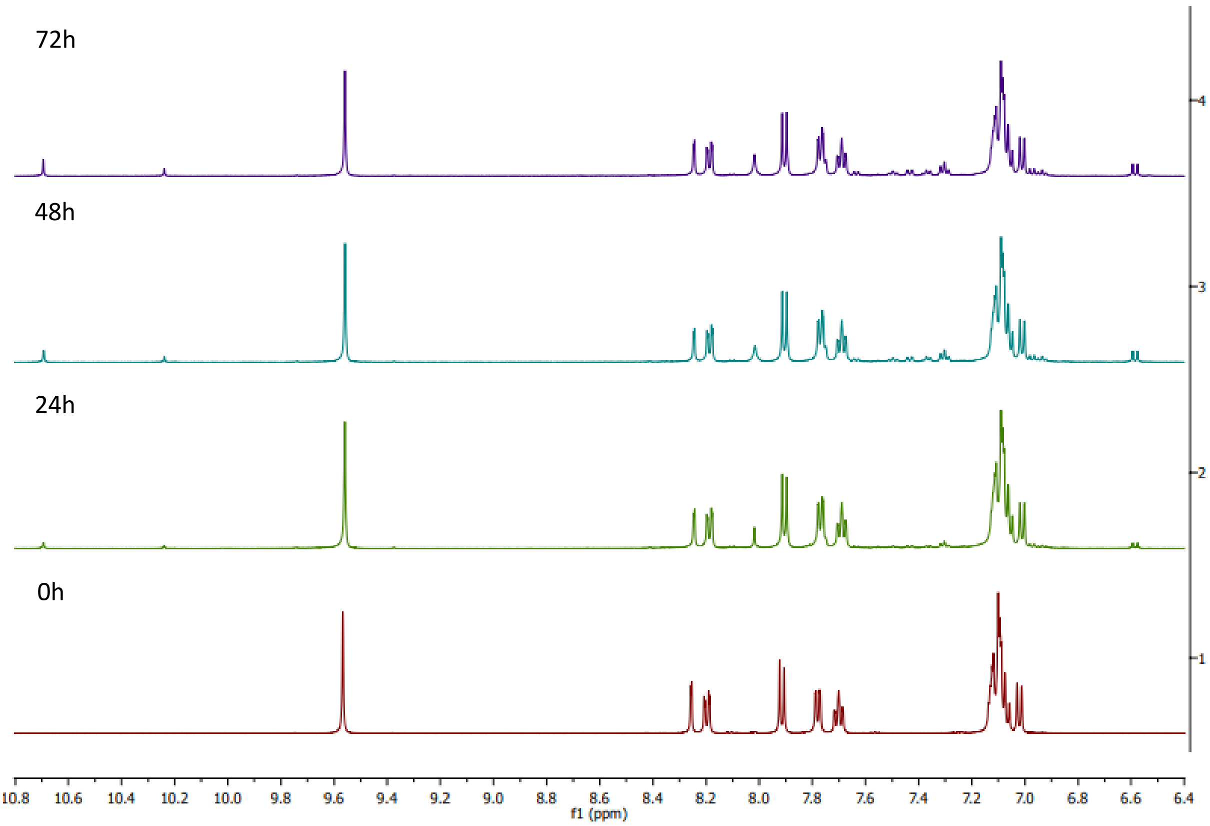
72h
48h
24h
0h
10.8 10.6 10.4 10.2 10.0 9.8 9.6 9.4 9.2 9.0 8.8 8.6 8.4 8.2 8.0 7.8 7.6 7.4 7.2 7.0 6.8 6.6 6.4
f1 (ppm)
4
3
2
1

26

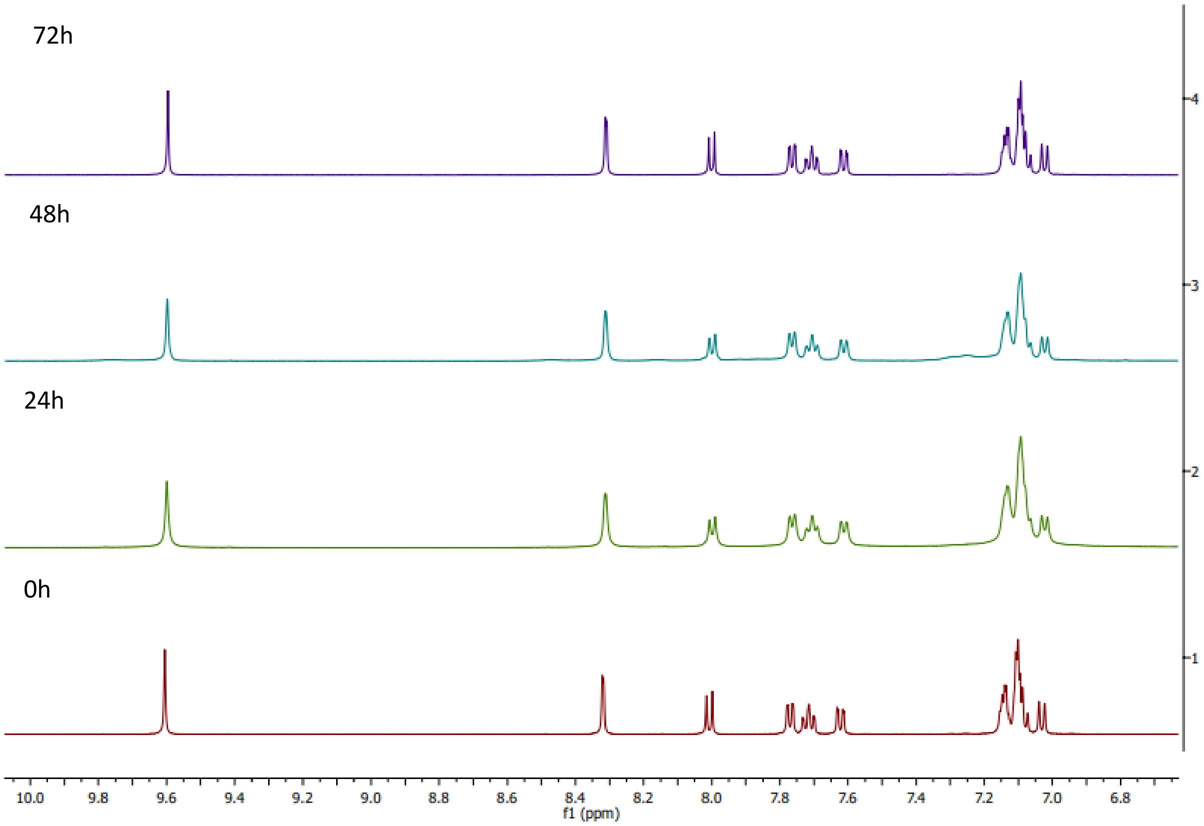
72h
48h
24h
0h
10.0 9.8 9.6 9.4 9.2 9.0 8.8 8.6 8.4 8.2 8.0 7.8 7.6 7.4 7.2 7.0 6.8
f1 (ppm)
4
3
2
1

27

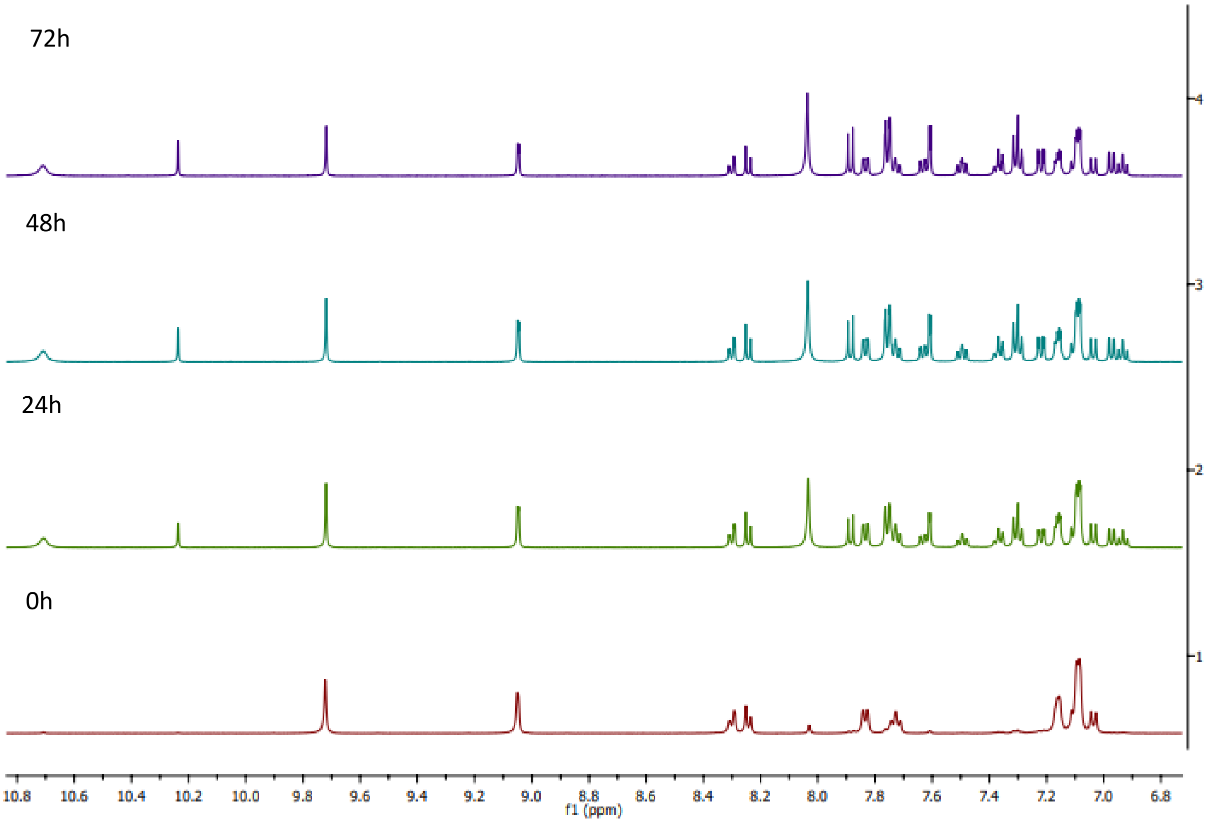

27 heat for 2 and 5 h

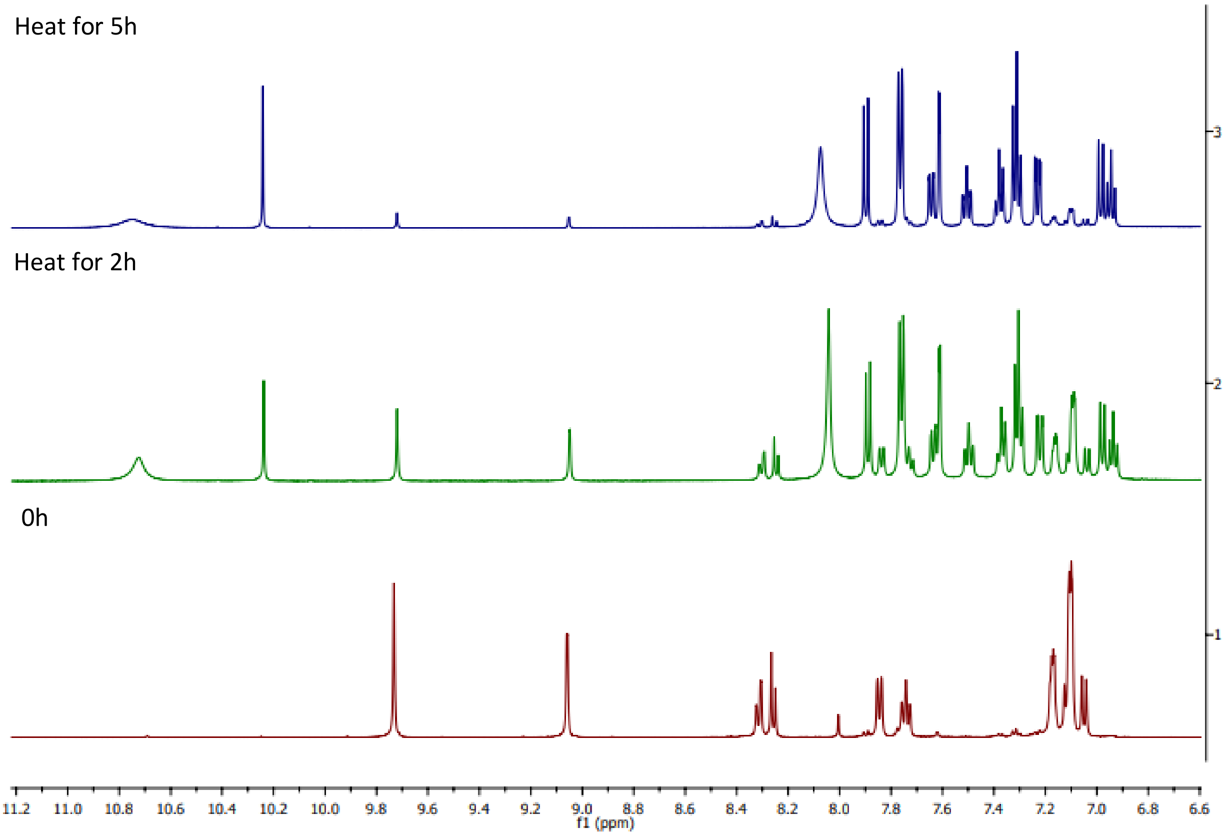

28

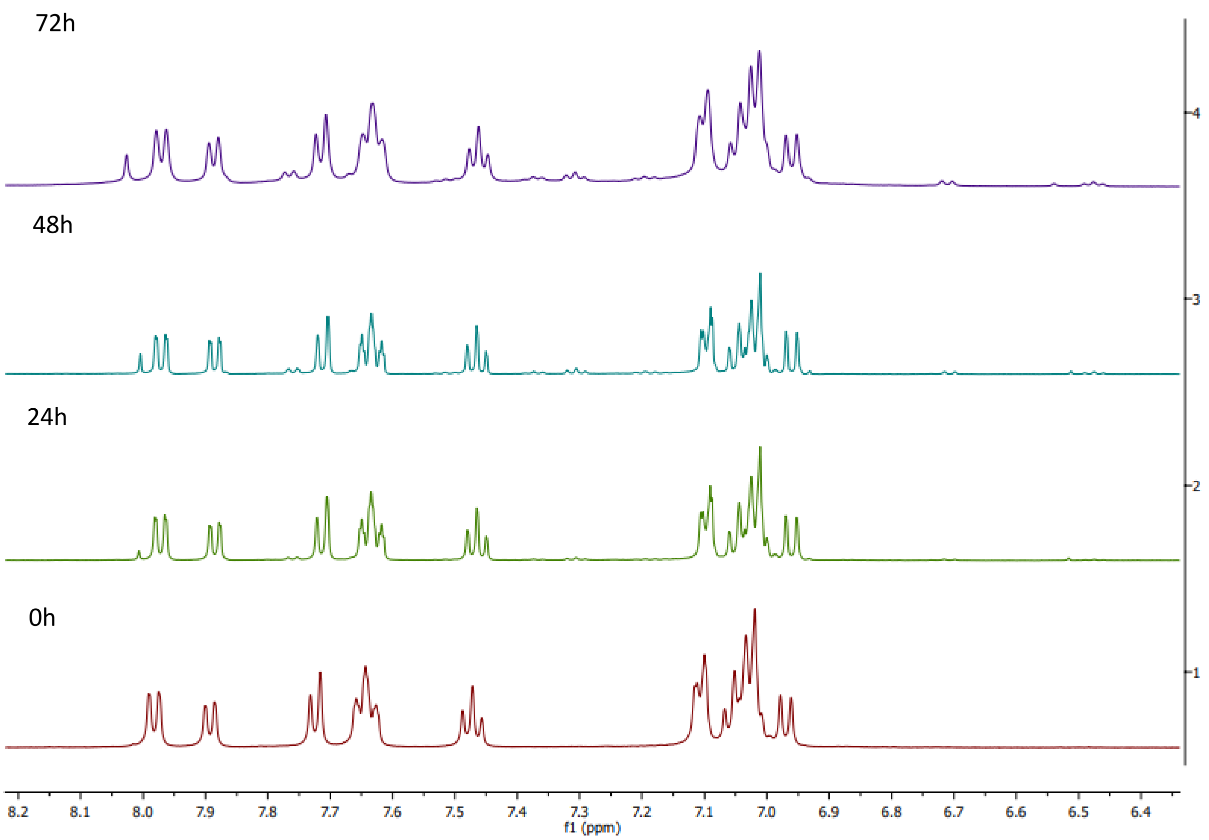

29

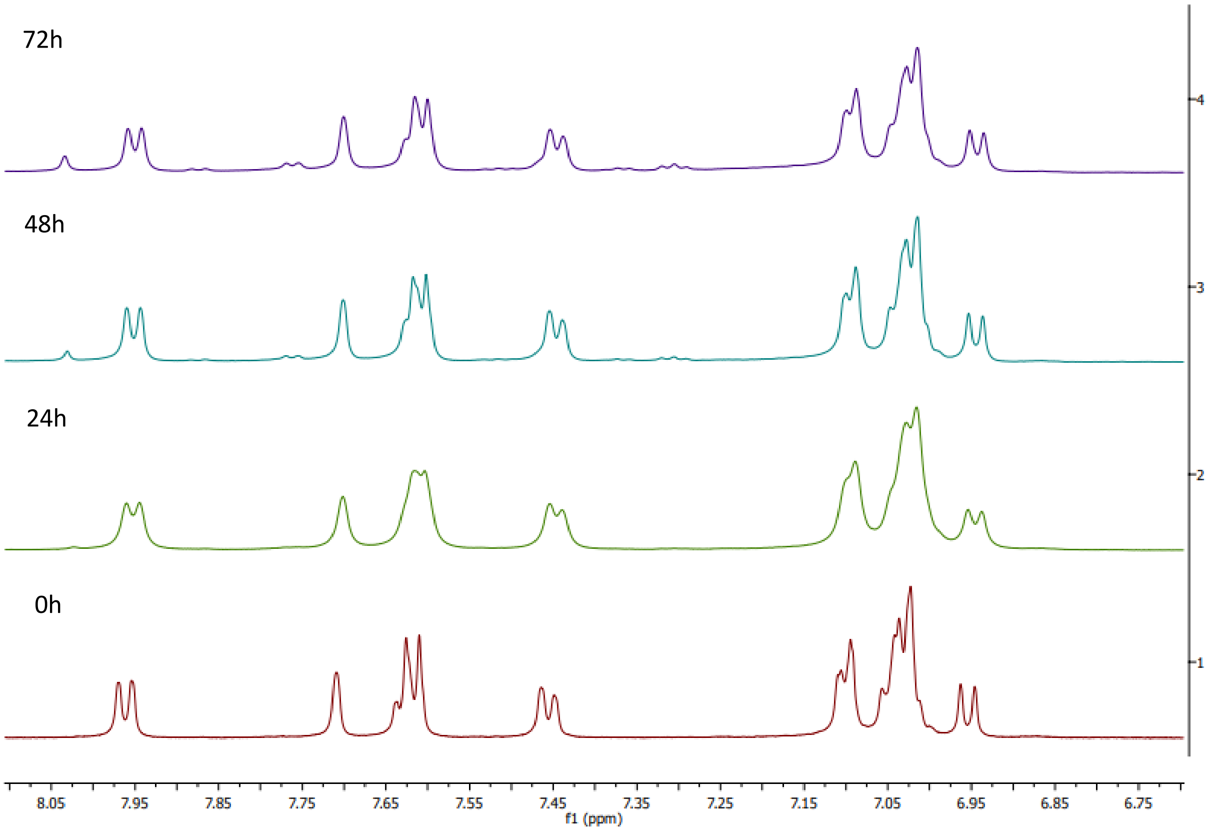

30

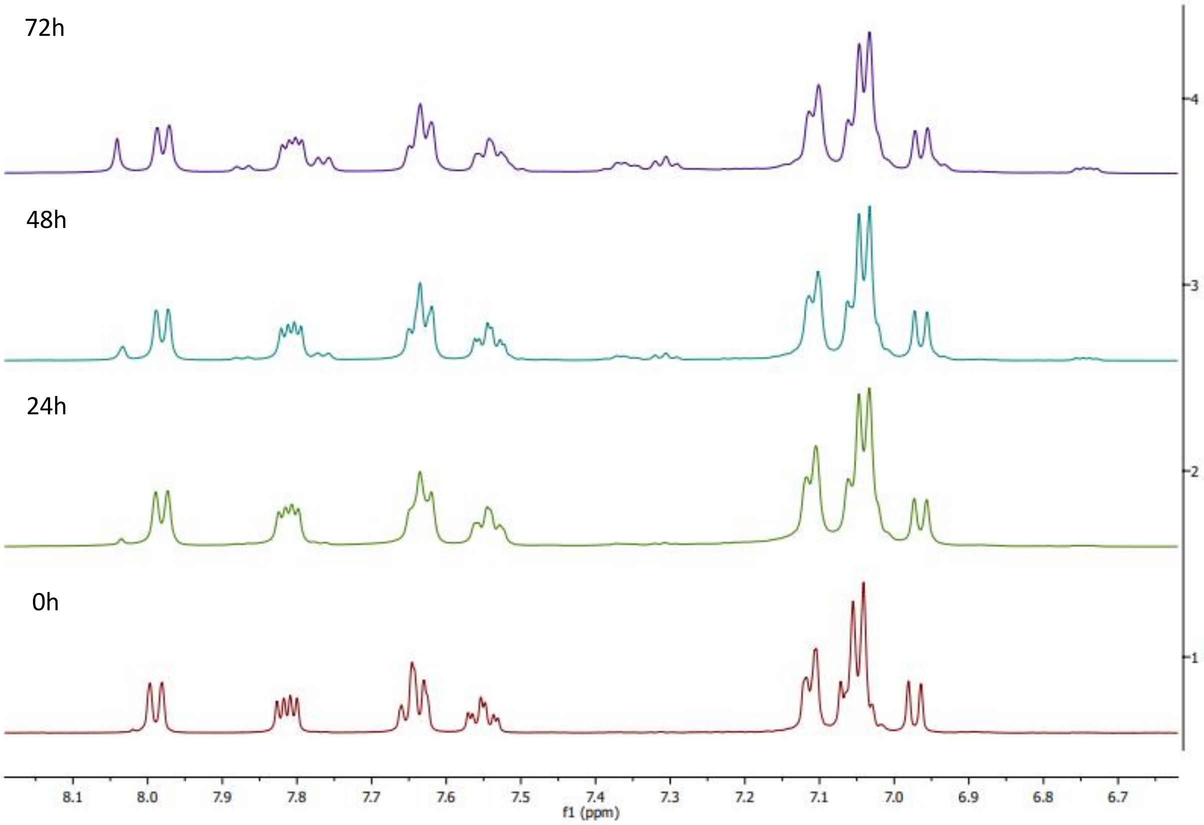
72h
48h
24h
0h
8.1 8.0 7.9 7.8 7.7 7.6 7.5 7.4 7.3 7.2 7.1 7.0 6.9 6.8 6.7
f1 (ppm)
1
2
3
4

31

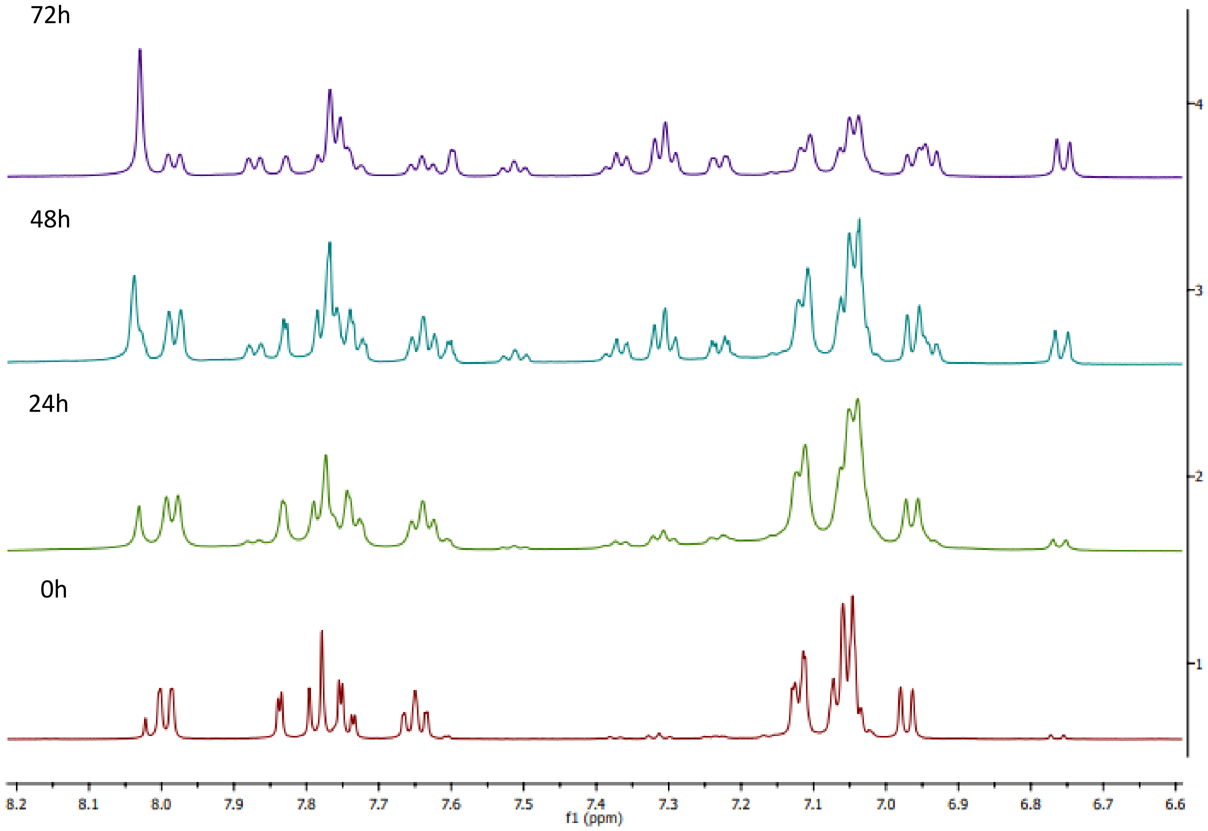
72h
48h
24h
0h
8.2 8.1 8.0 7.9 7.8 7.7 7.6 7.5 7.4 7.3 7.2 7.1 7.0 6.9 6.8 6.7 6.6
f1 (ppm)
1
2
3
4

32

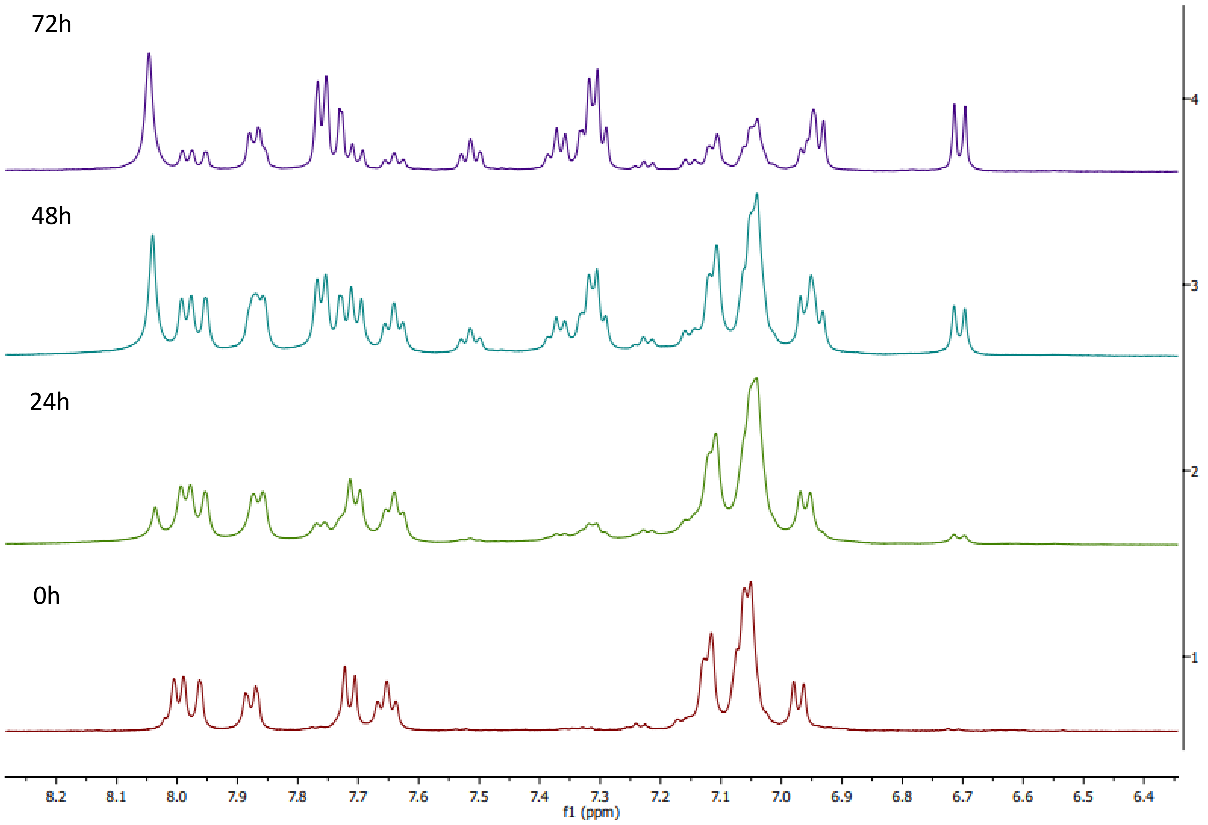
72h
48h
24h
0h
8.2
8.1
8.0
7.9
7.8
7.7
7.6
7.5
7.4
7.3
7.2
7.1
7.0
6.9
6.8
6.7
6.6
6.5
6.4
f1 (ppm)
4
3
2
1

33

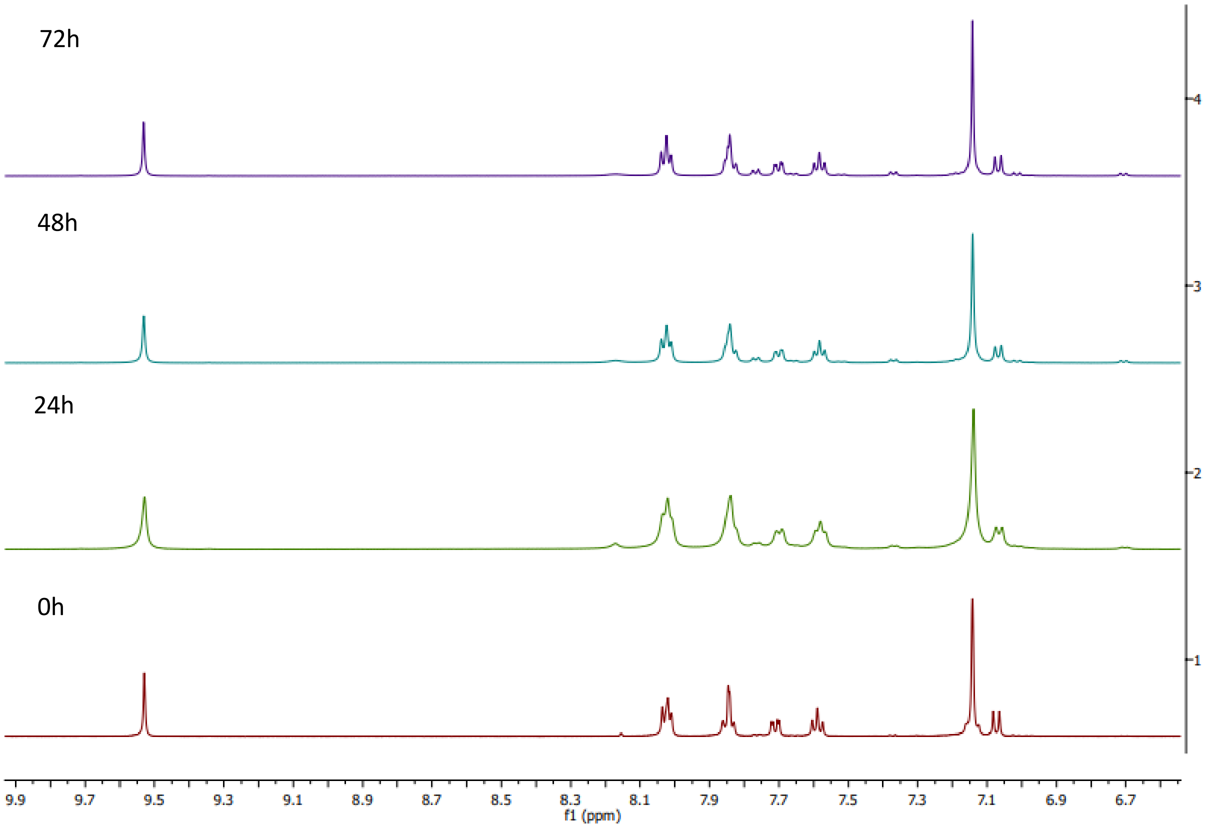
72h
48h
24h
0h
9.9
9.7
9.5
9.3
9.1
8.9
8.7
8.5
8.3
8.1
7.9
7.7
7.5
7.3
7.1
6.9
6.7
f1 (ppm)
4
3
2
1

34

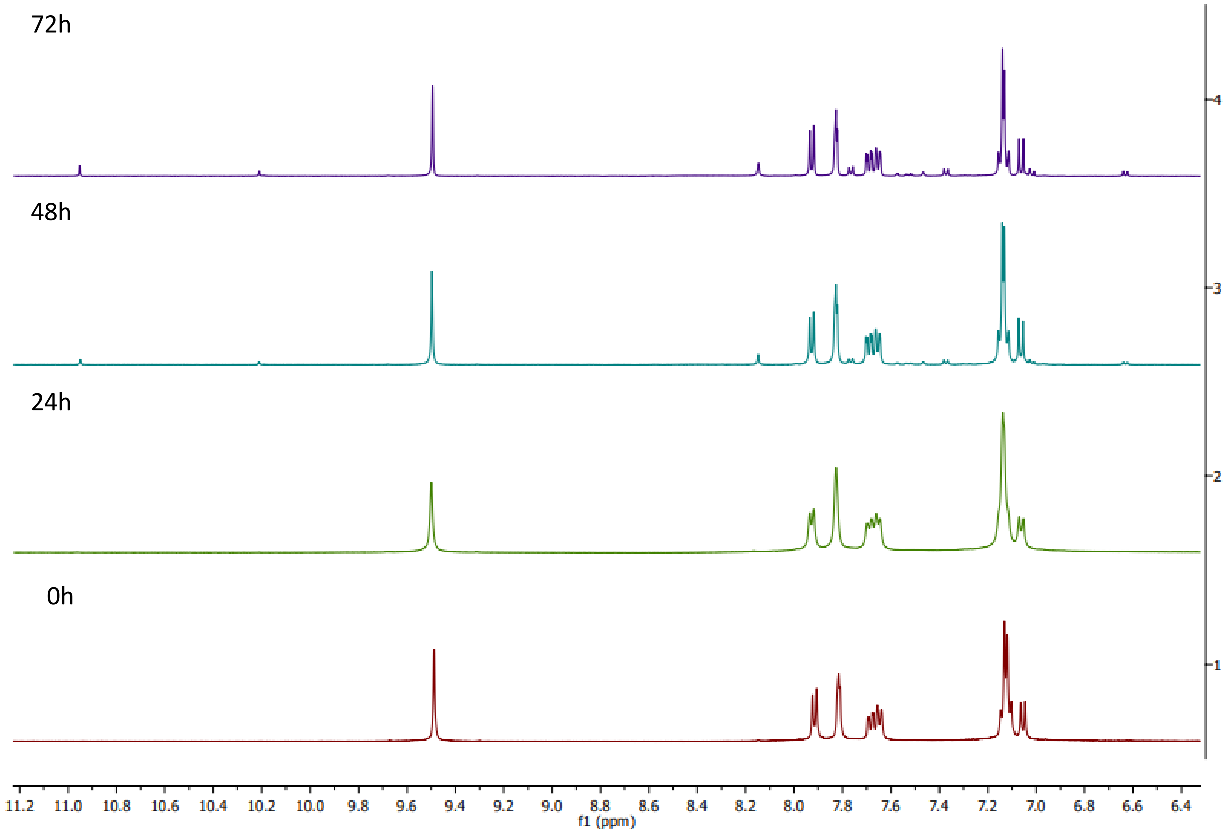

72h
48h
24h
0h
11.2 11.0 10.8 10.6 10.4 10.2 10.0 9.8 9.6 9.4 9.2 9.0 8.8 8.6 8.4 8.2 8.0 7.8 7.6 7.4 7.2 7.0 6.8 6.6 6.4
f1 (ppm)
4
3
2
1

35

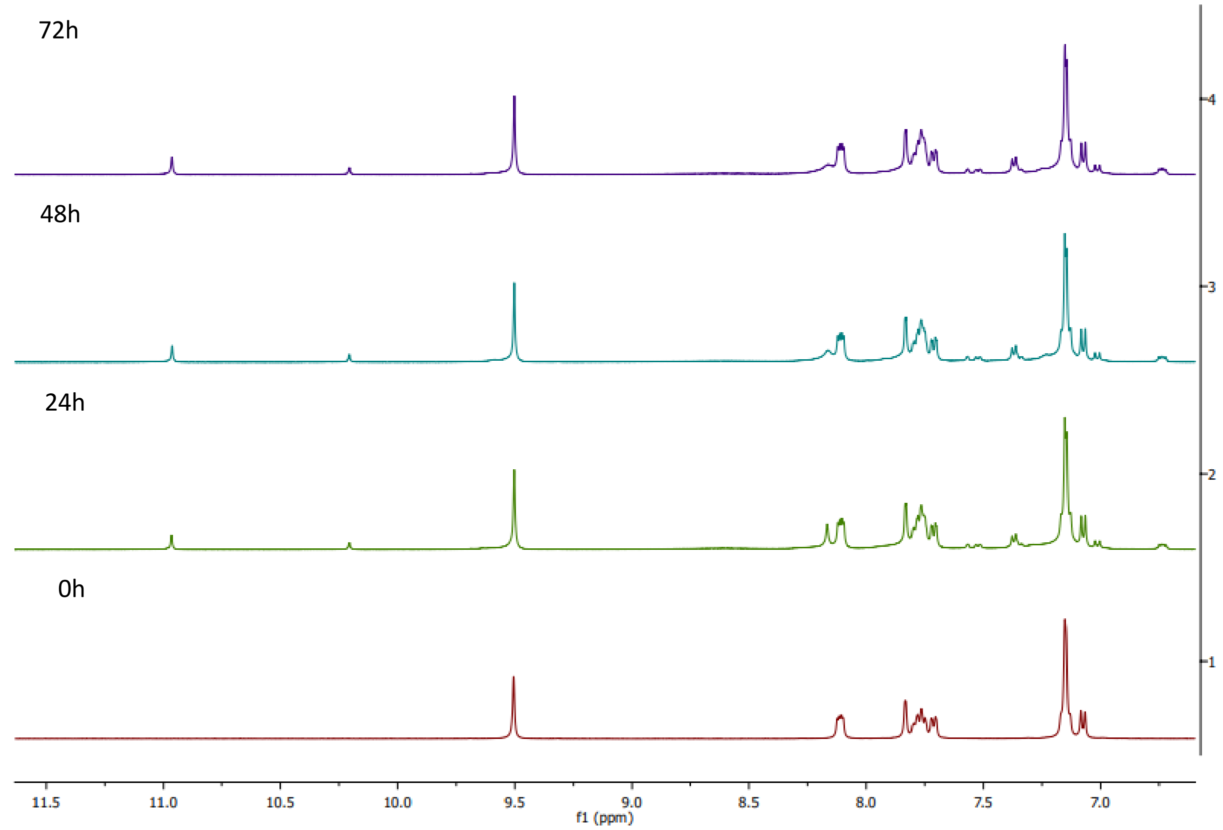

72h
48h
24h
0h
11.5 11.0 10.5 10.0 9.5 9.0 8.5 8.0 7.5 7.0
f1 (ppm)
4
3
2
1

36

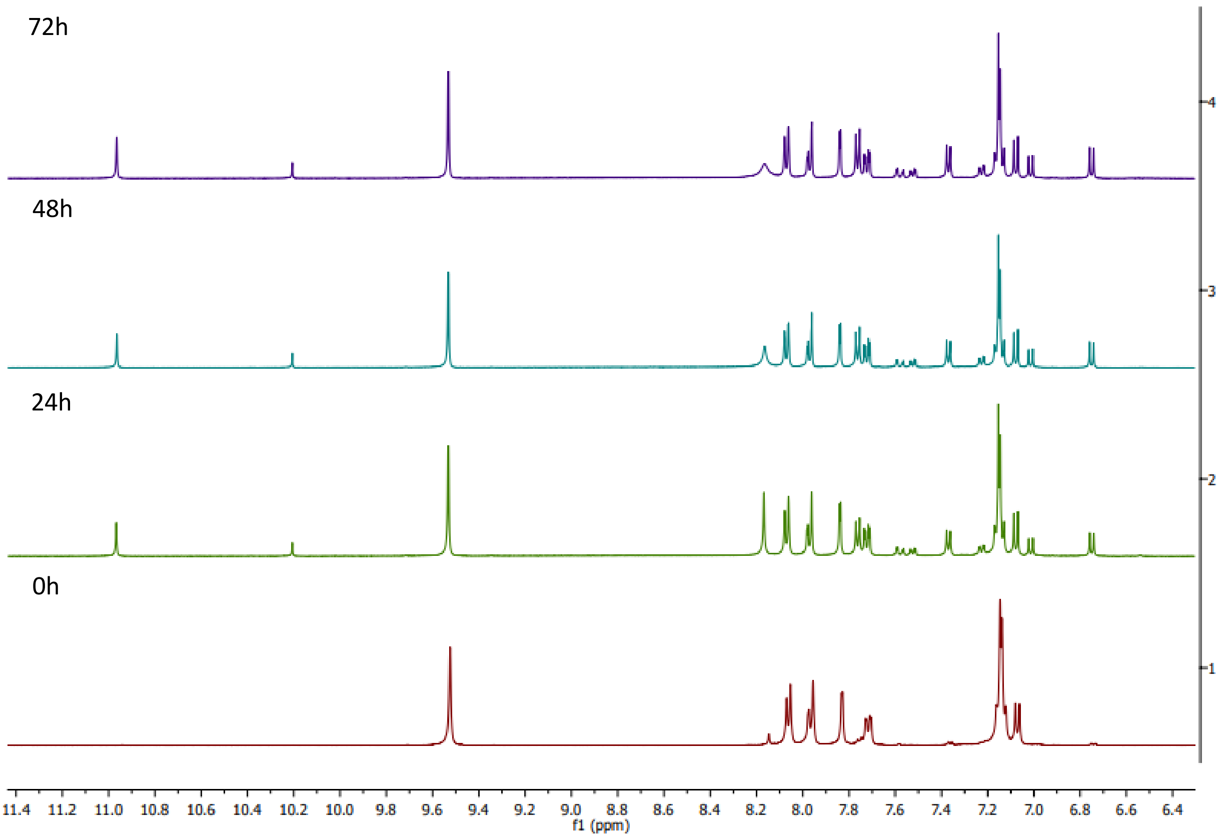
72h
48h
24h
0h
11.4 11.2 11.0 10.8 10.6 10.4 10.2 10.0 9.8 9.6 9.4 9.2 9.0 8.8 8.6 8.4 8.2 8.0 7.8 7.6 7.4 7.2 7.0 6.8 6.6 6.4
f1 (ppm)
4
3
2
1

37

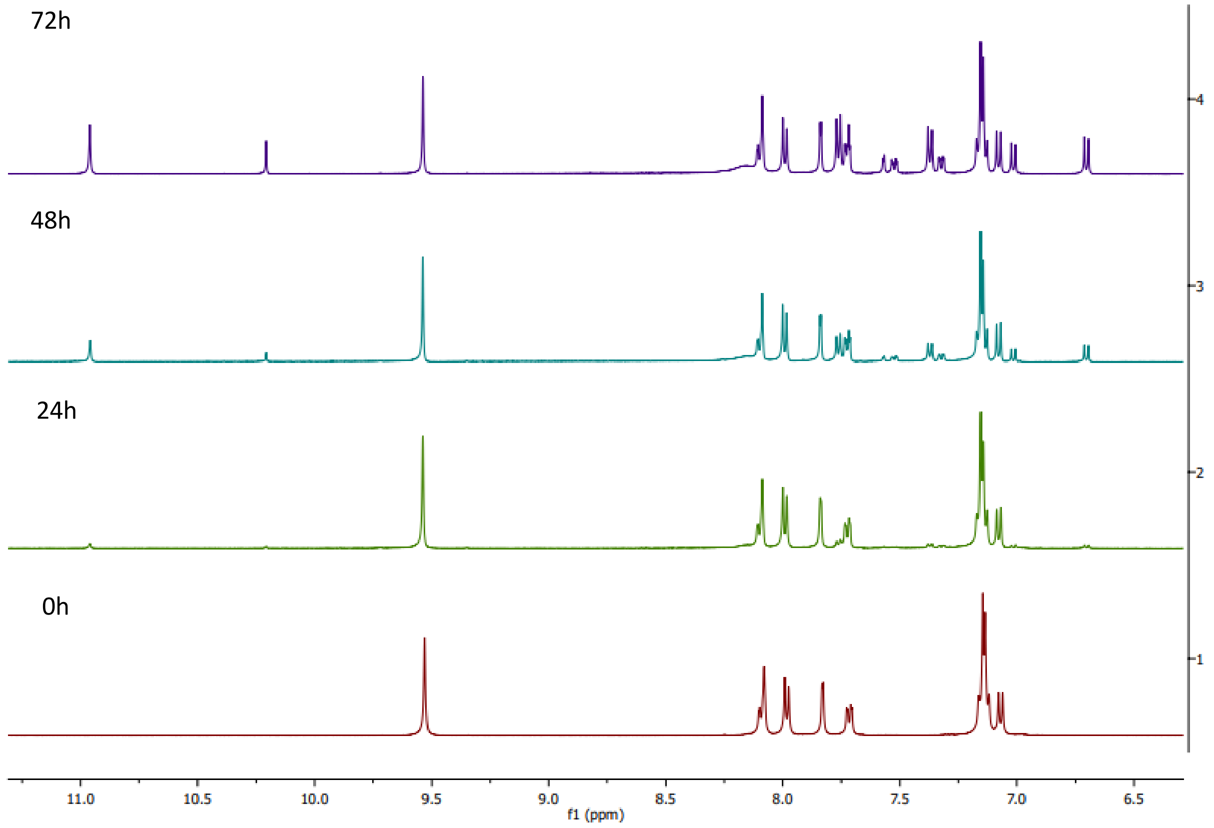
72h
48h
24h
0h
11.0 10.5 10.0 9.5 9.0 8.5 8.0 7.5 7.0 6.5
f1 (ppm)
4
3
2
1

38

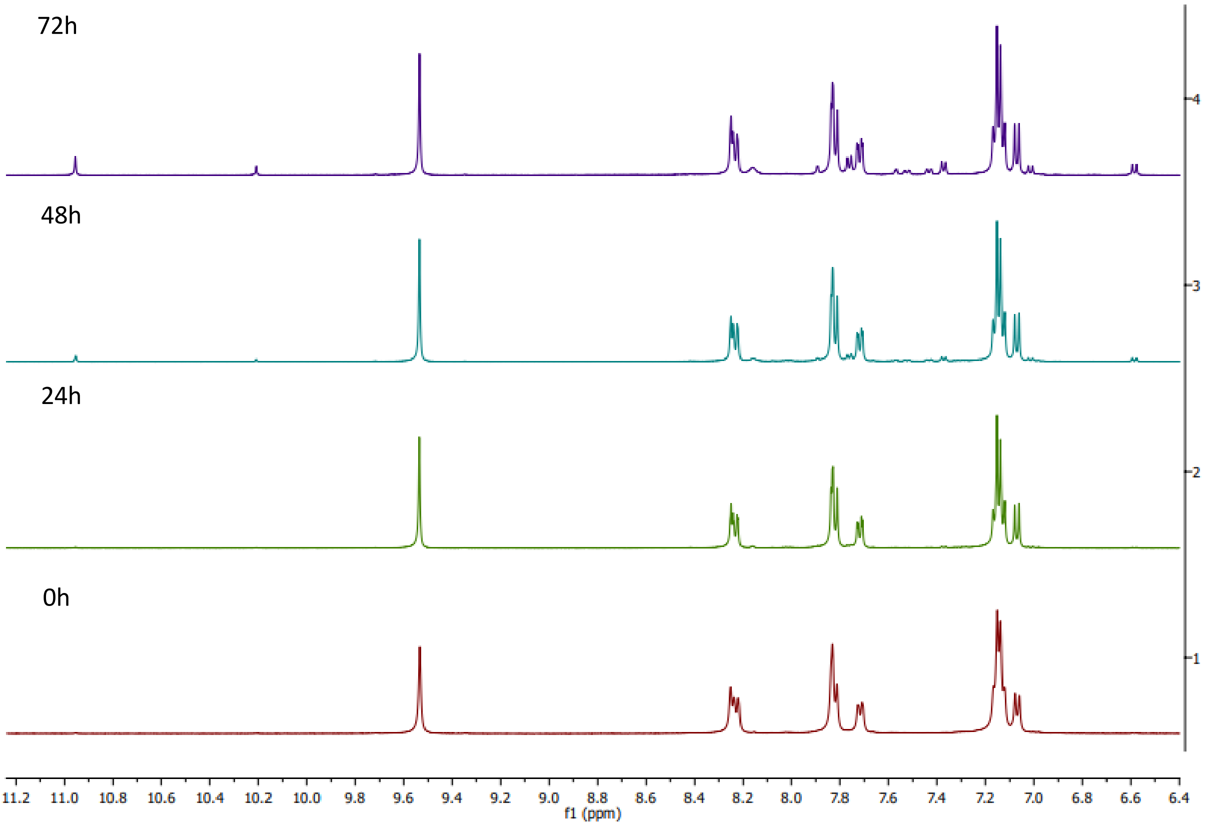
72h
48h
24h
0h
4
3
2
1
11.2 11.0 10.8 10.6 10.4 10.2 10.0 9.8 9.6 9.4 9.2 9.0 8.8 8.6 8.4 8.2 8.0 7.8 7.6 7.4 7.2 7.0 6.8 6.6 6.4
f1 (ppm)

39

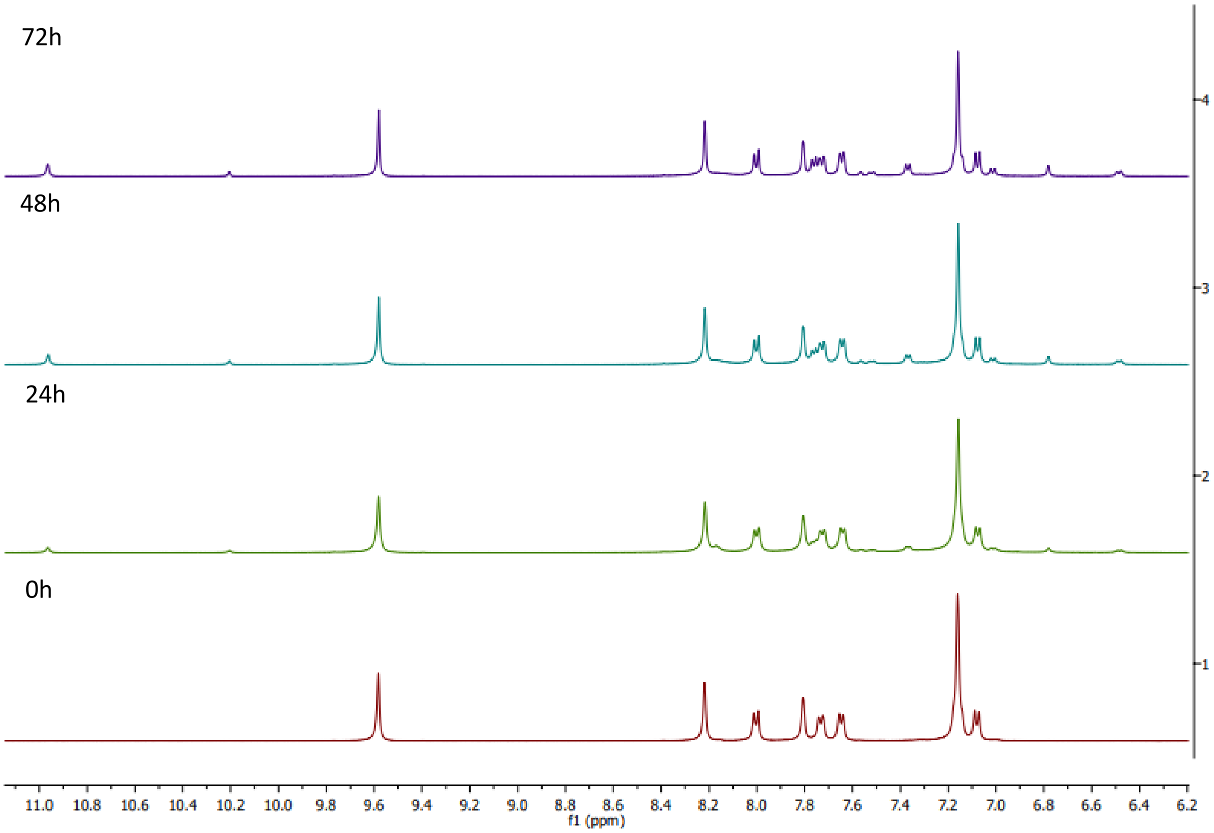
72h
48h
24h
0h
4
3
2
1
11.0 10.8 10.6 10.4 10.2 10.0 9.8 9.6 9.4 9.2 9.0 8.8 8.6 8.4 8.2 8.0 7.8 7.6 7.4 7.2 7.0 6.8 6.6 6.4 6.2
f1 (ppm)

40

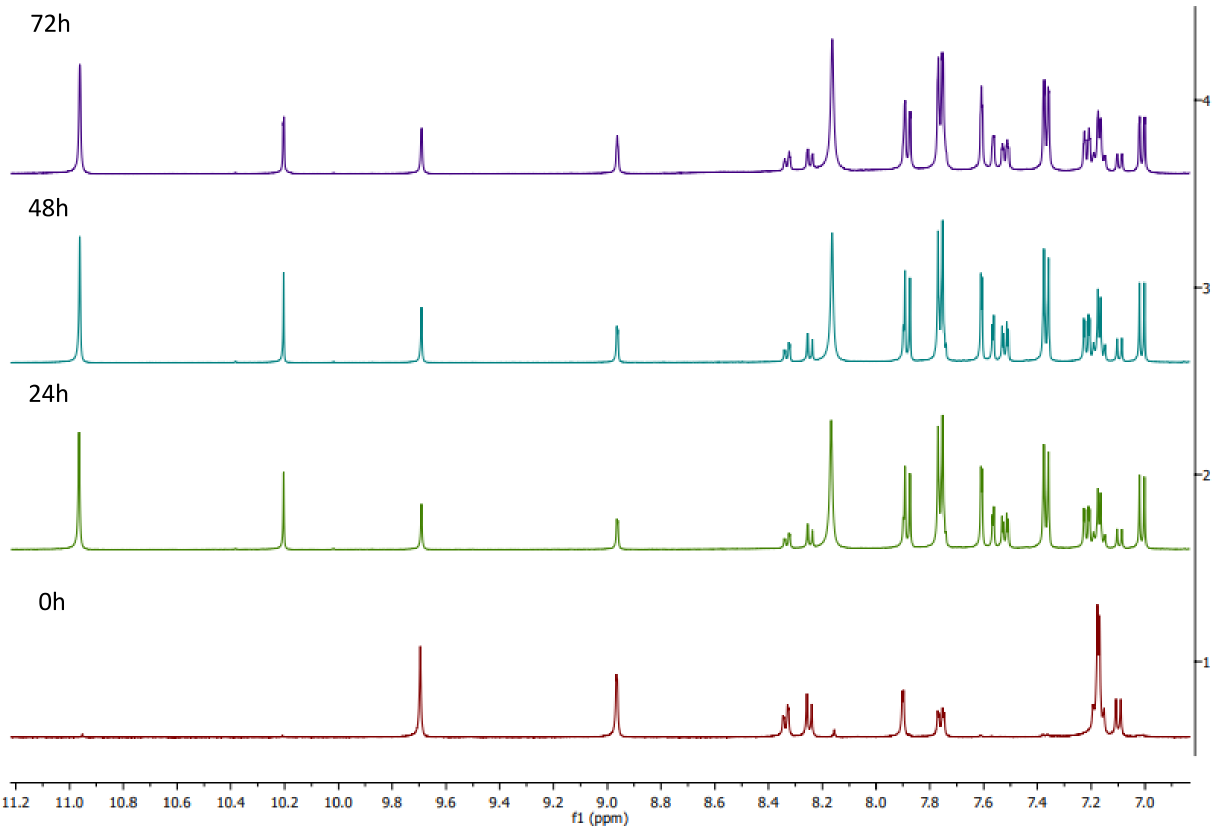

40

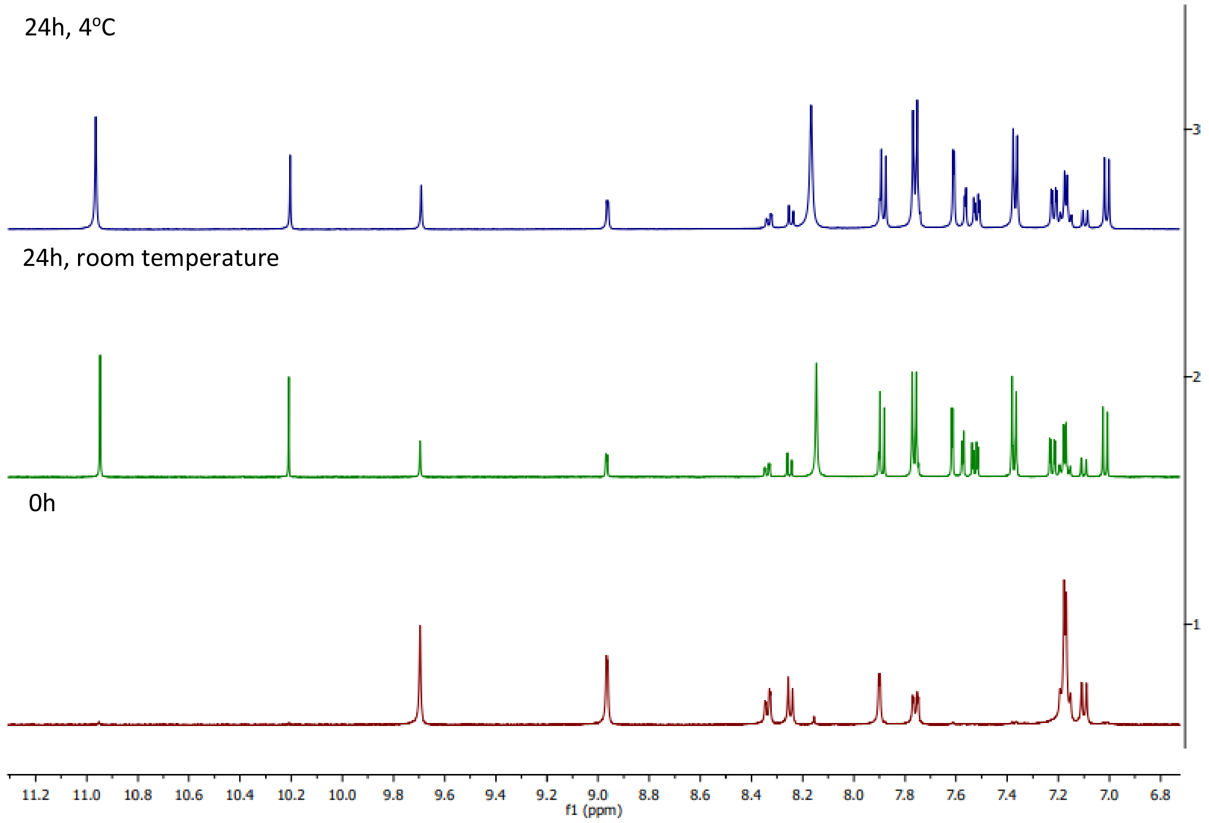

41

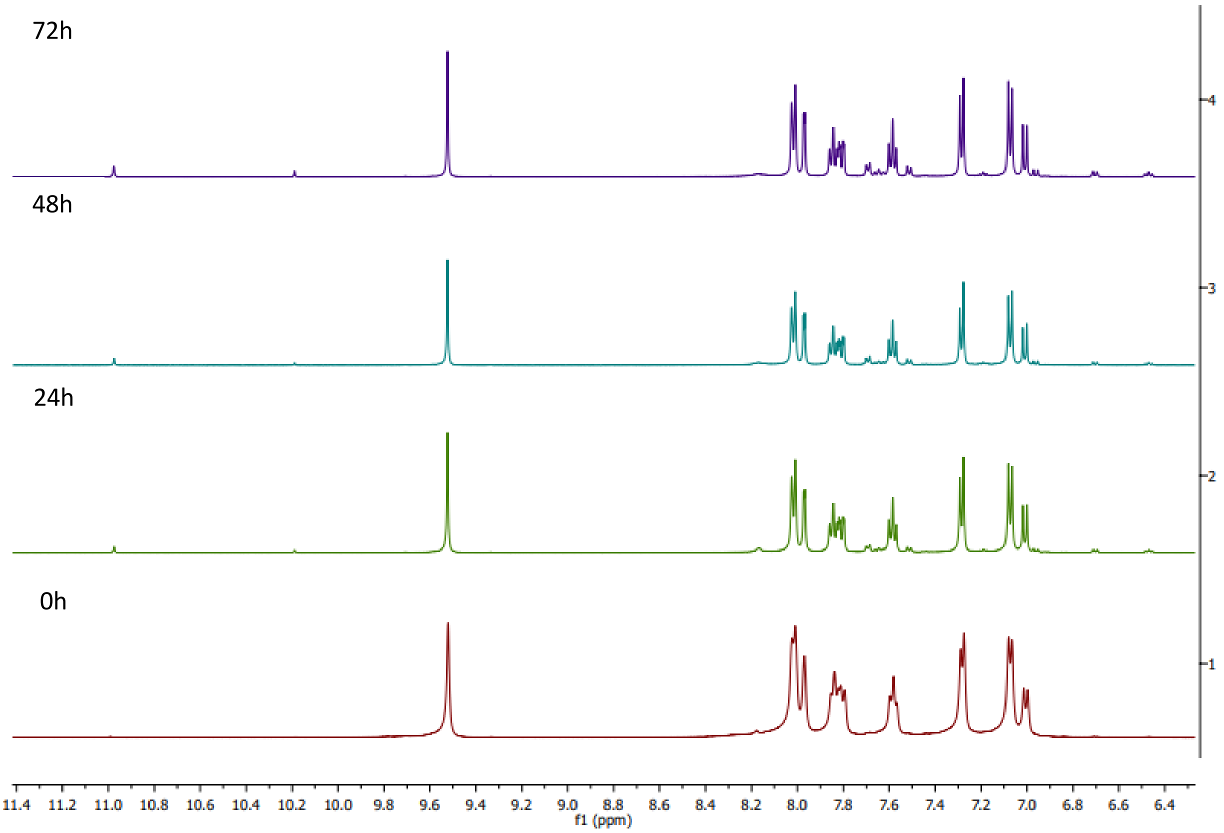
72h
48h
24h
0h
11.4 11.2 11.0 10.8 10.6 10.4 10.2 10.0 9.8 9.6 9.4 9.2 9.0 8.8 8.6 8.4 8.2 8.0 7.8 7.6 7.4 7.2 7.0 6.8 6.6 6.4
f1 (ppm)
4
3
2
1

42

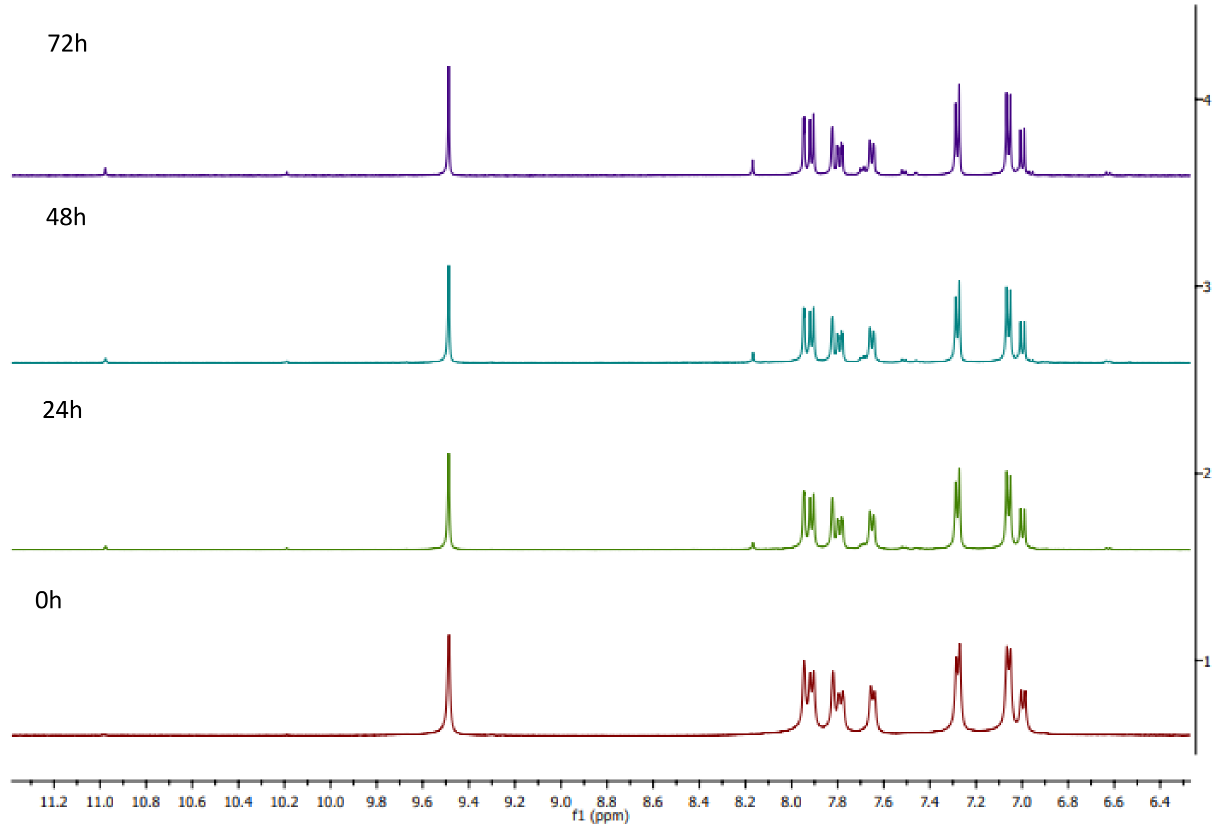
72h
48h
24h
0h
11.2 11.0 10.8 10.6 10.4 10.2 10.0 9.8 9.6 9.4 9.2 9.0 8.8 8.6 8.4 8.2 8.0 7.8 7.6 7.4 7.2 7.0 6.8 6.6 6.4
f1 (ppm)
4
3
2
1

43

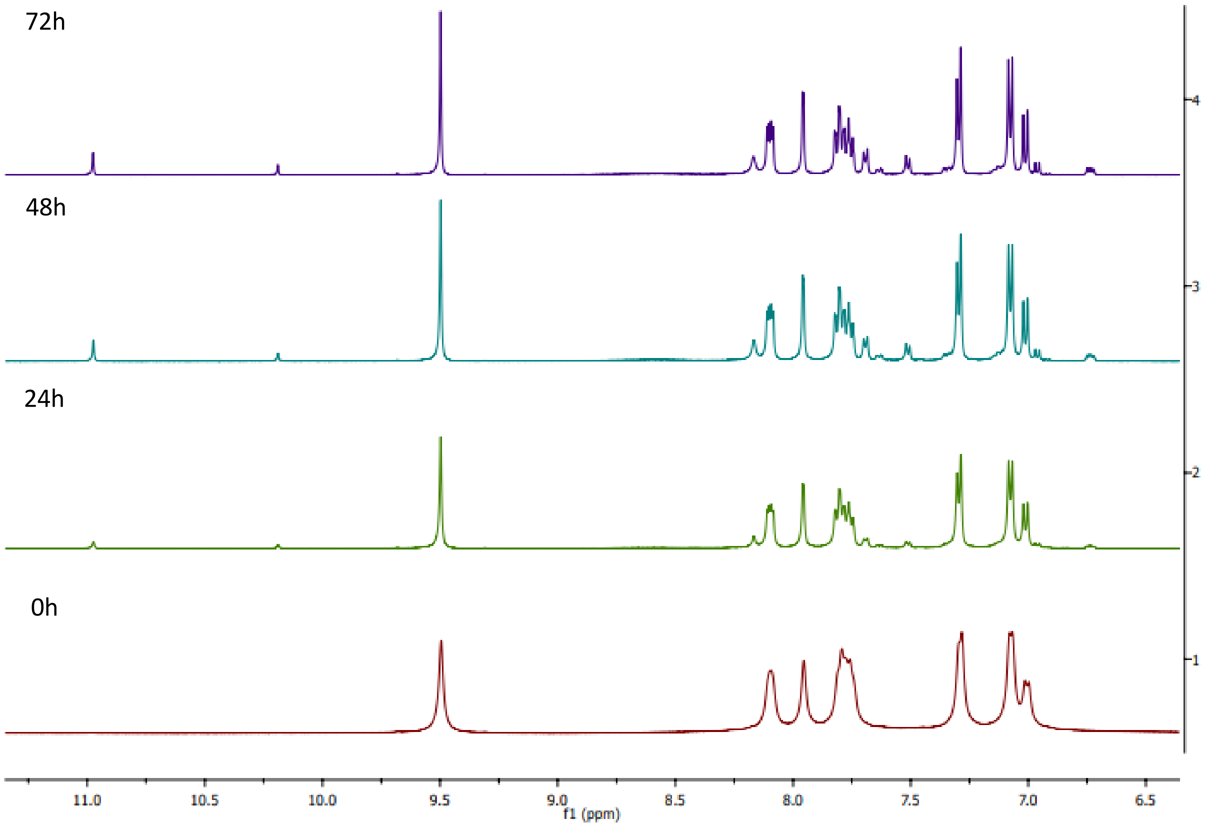
72h
48h
24h
0h
11.0
10.5
10.0
9.5
9.0
8.5
8.0
7.5
7.0
6.5
f1 (ppm)
4
3
2
1

44

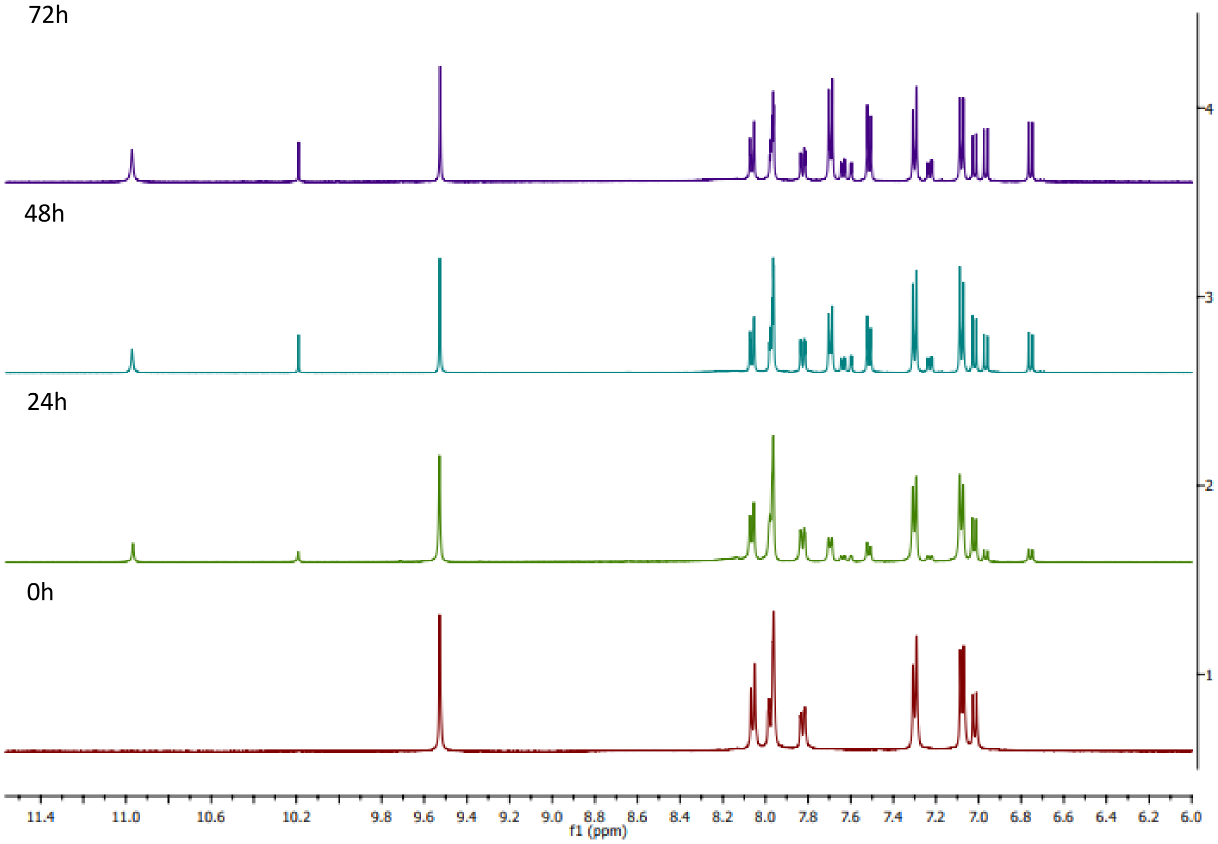
72h
48h
24h
0h
11.4
11.0
10.6
10.2
9.8
9.6
9.4
9.2
9.0
8.8
8.6
8.4
8.2
8.0
7.8
7.6
7.4
7.2
7.0
6.8
6.6
6.4
6.2
6.0
f1 (ppm)
4
3
2
1

45

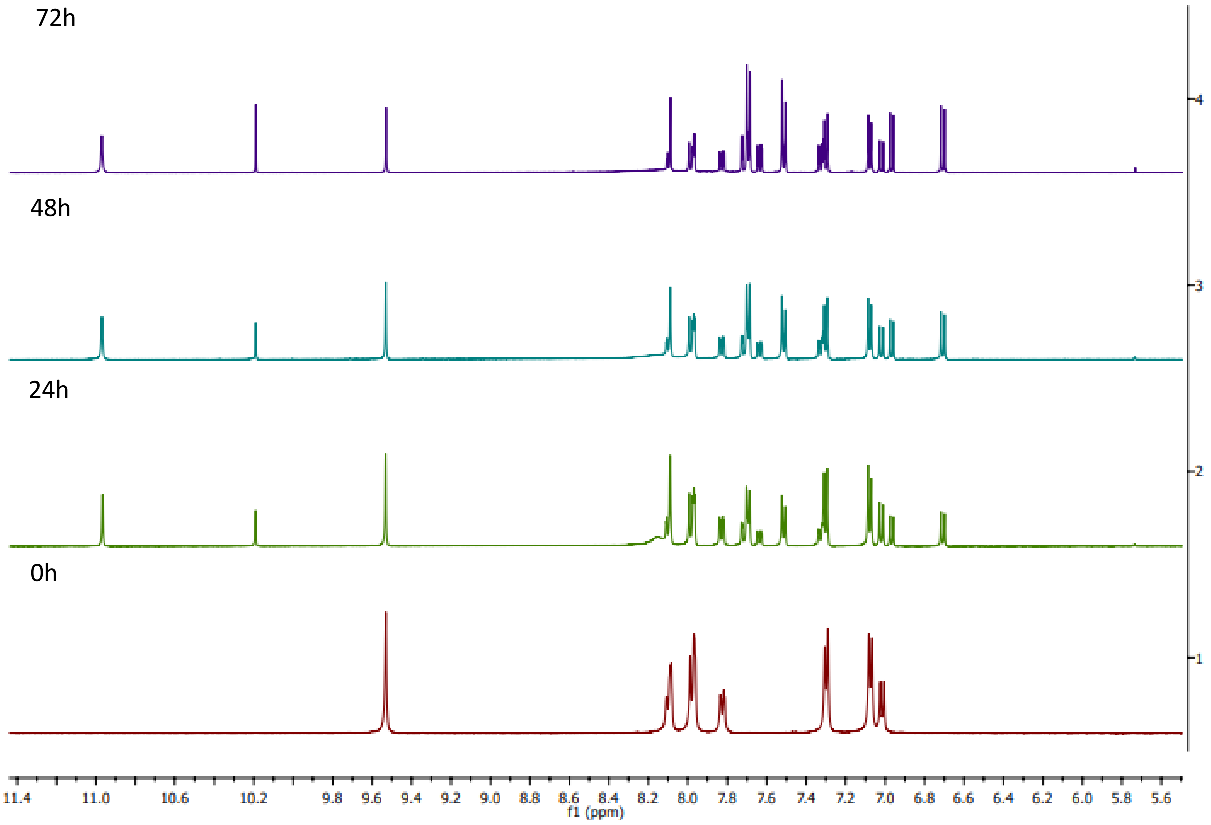
72h
48h
24h
0h
11.4 11.0 10.6 10.2 9.8 9.6 9.4 9.2 9.0 8.8 8.6 8.4 8.2 8.0 7.8 7.6 7.4 7.2 7.0 6.8 6.6 6.4 6.2 6.0 5.8 5.6
f1 (ppm)
4
3
2
1

46

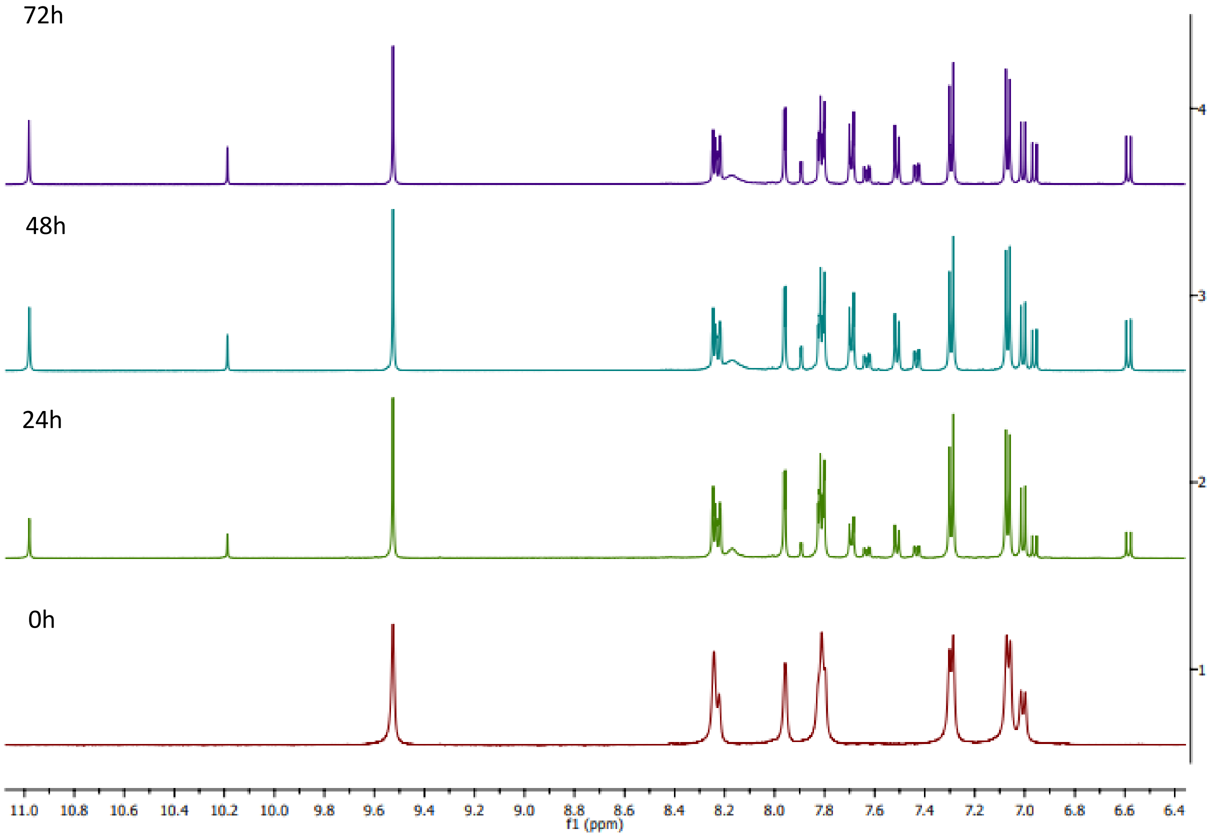
72h
48h
24h
0h
11.0 10.8 10.6 10.4 10.2 10.0 9.8 9.6 9.4 9.2 9.0 8.8 8.6 8.4 8.2 8.0 7.8 7.6 7.4 7.2 7.0 6.8 6.6 6.4
f1 (ppm)
4
3
2
1

47

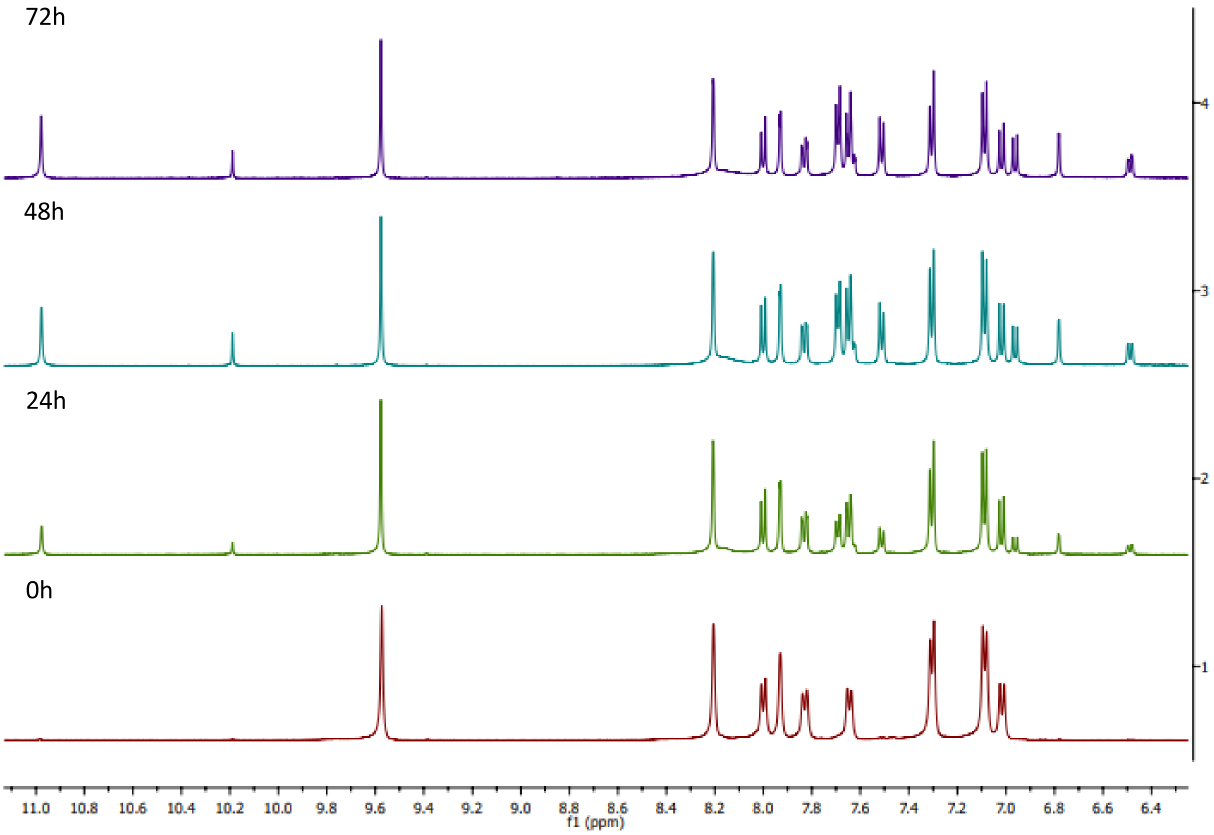
72h
48h
24h
0h
11.0 10.8 10.6 10.4 10.2 10.0 9.8 9.6 9.4 9.2 9.0 8.8 8.6 8.4 8.2 8.0 7.8 7.6 7.4 7.2 7.0 6.8 6.6 6.4
f1 (ppm)
4
3
2
1

48

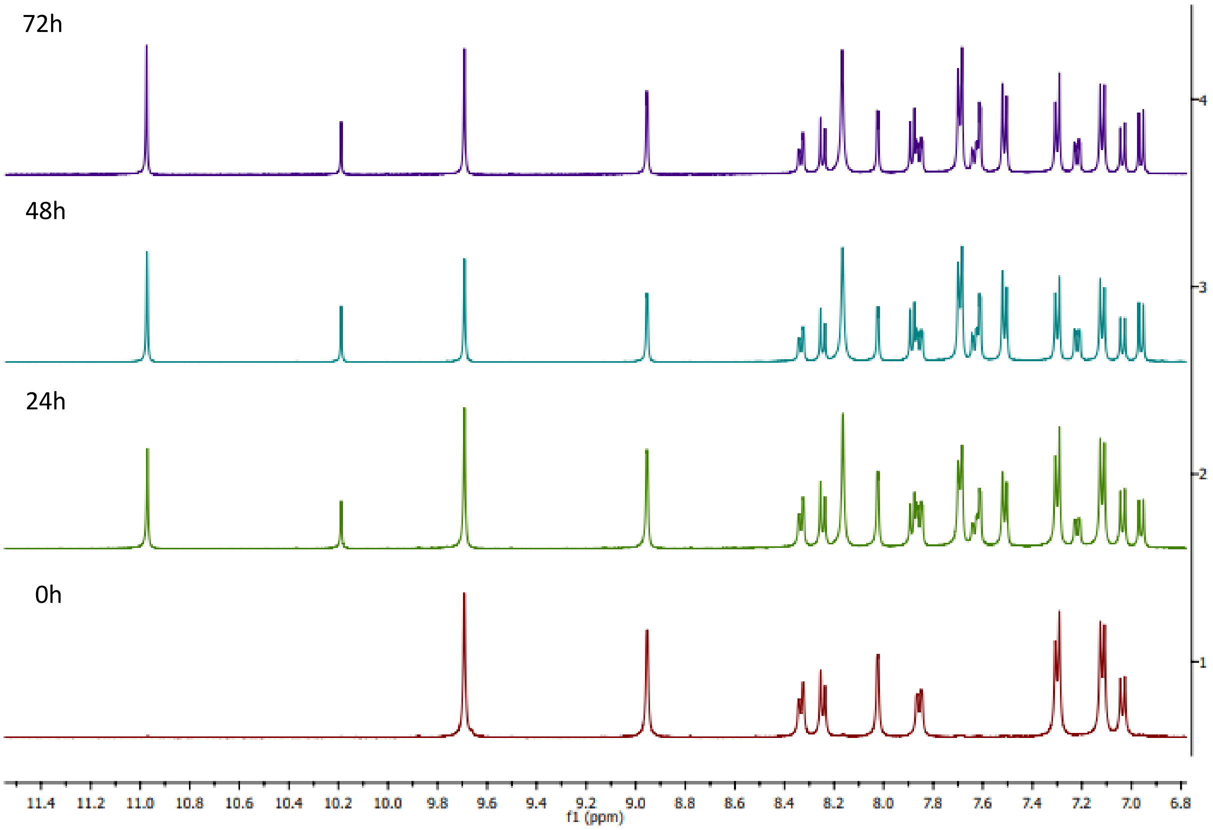
72h
48h
24h
0h
11.4 11.2 11.0 10.8 10.6 10.4 10.2 10.0 9.8 9.6 9.4 9.2 9.0 8.8 8.6 8.4 8.2 8.0 7.8 7.6 7.4 7.2 7.0 6.8
f1 (ppm)
4
3
2
1

S.3. Time dependent comparative NMR spectra of BASAN (18-27), BASAN-Cl (33-40) and BASAN-Br (41-48) derived from the same anthranilic acid

All H-anthranilic acid (1) derivatives; Top to bottom: 41 / 33 / 18 (after 48h at 4°C)

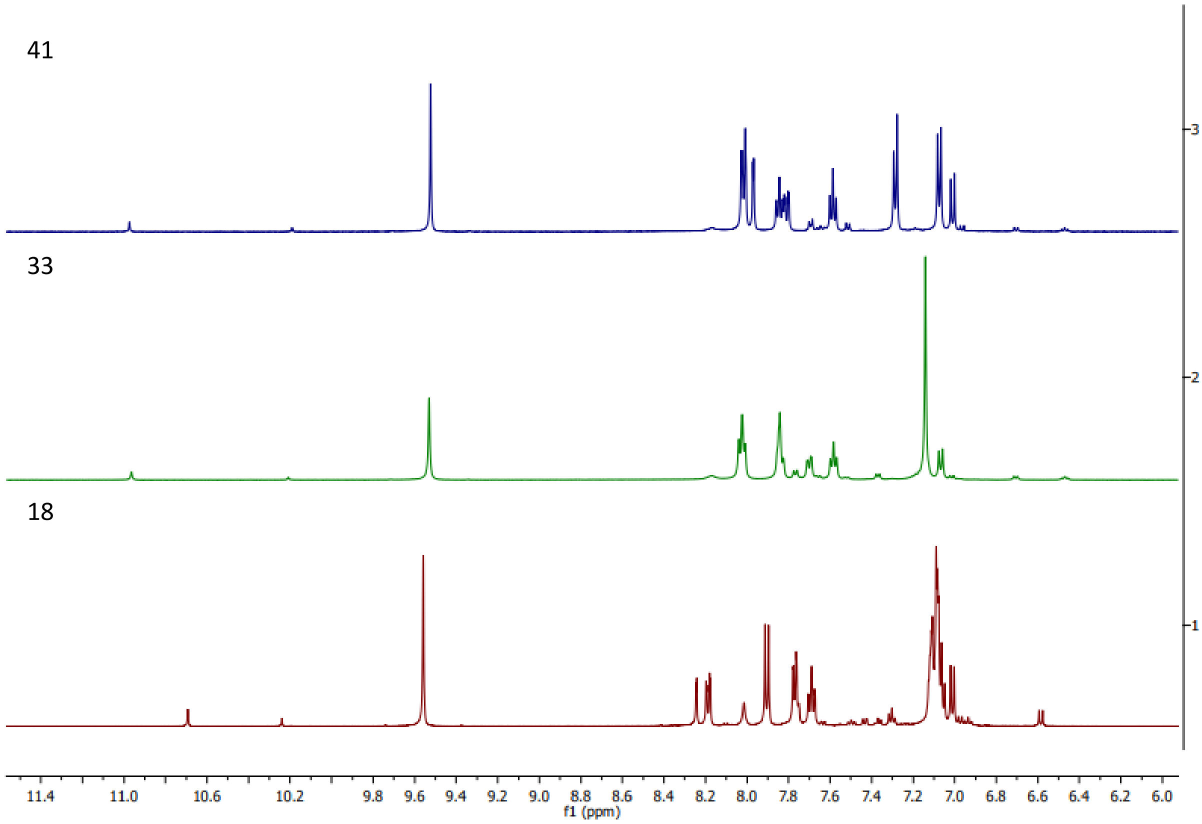

All Me-anthranilic acid (2) derivatives; Top to bottom: 42 / 34 / 19 (after 48h at 4°C)

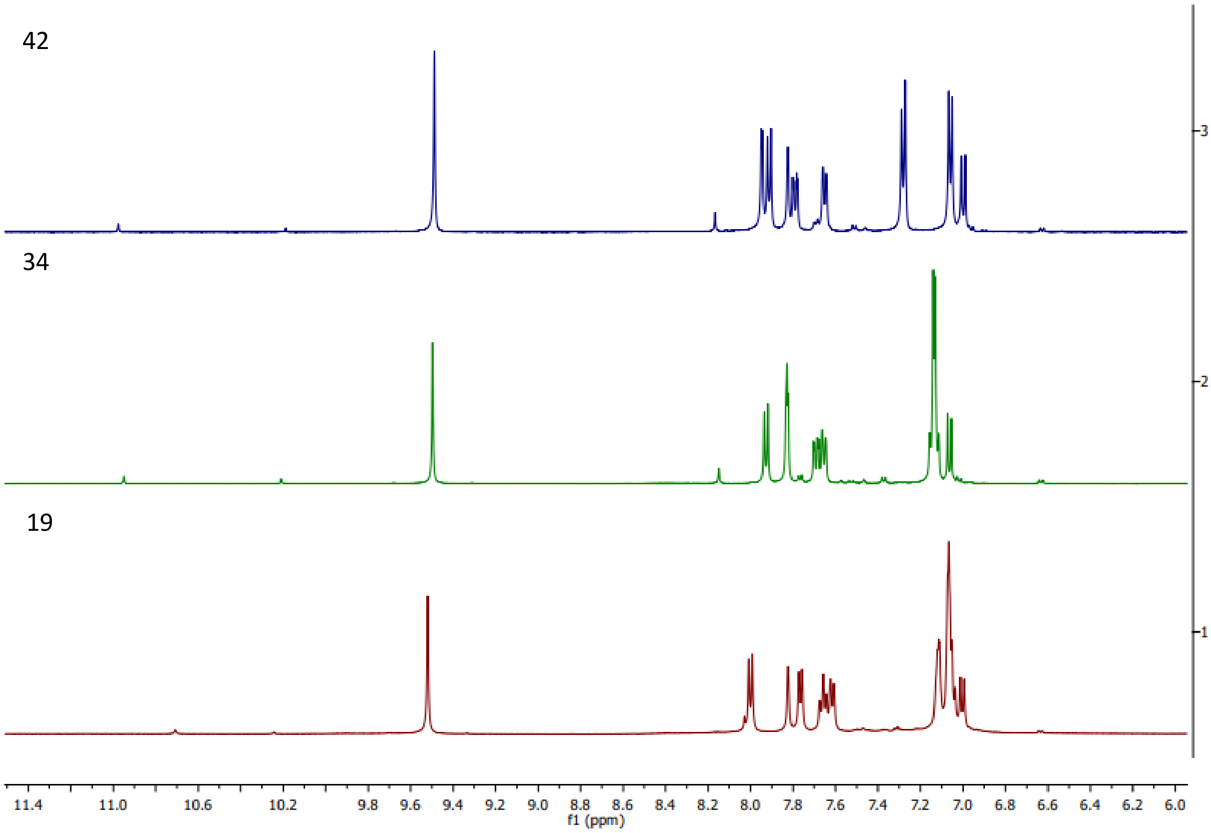

All F-anthranilic acid (4) derivatives; Top to bottom: 43 / 35 / 21 (after 48h at 4°C)

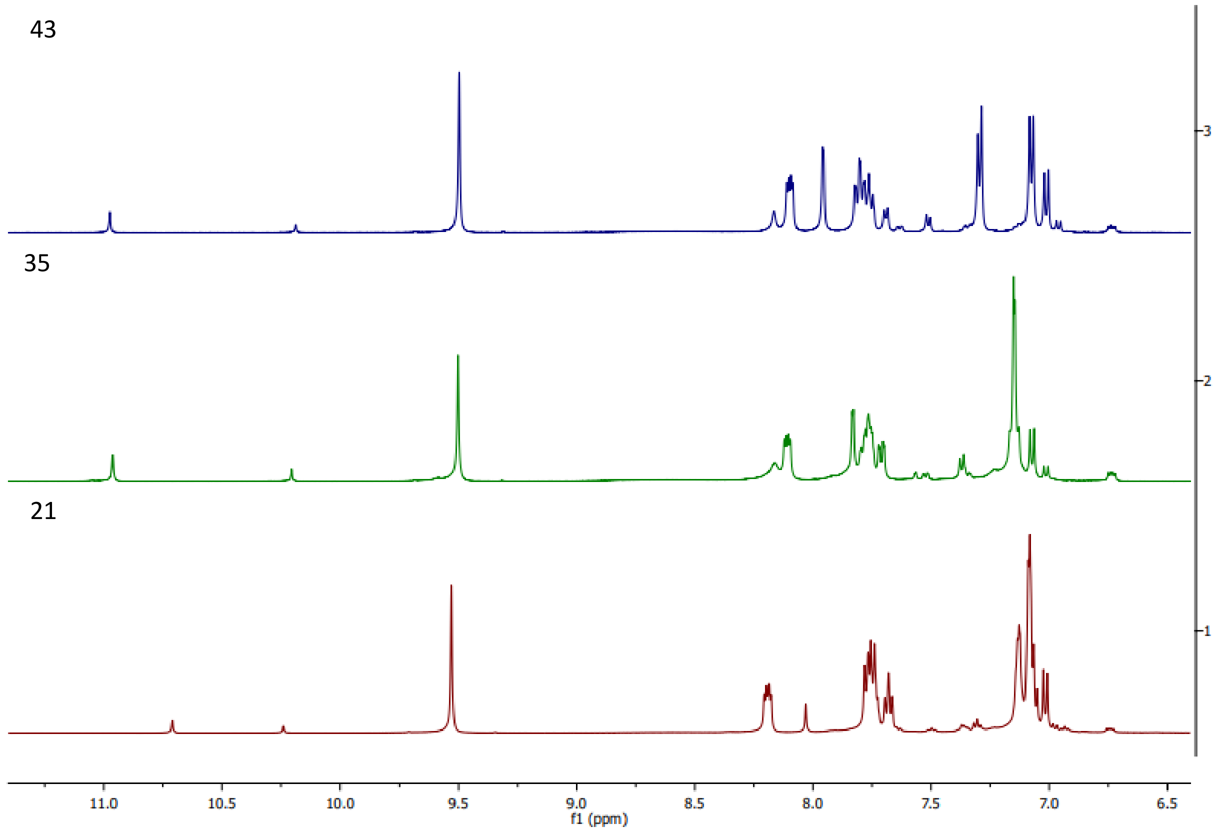

All Cl-anthranilic acid (5) derivatives; Top to bottom: 44 / 36 / 22 (after 48h at 4°C)

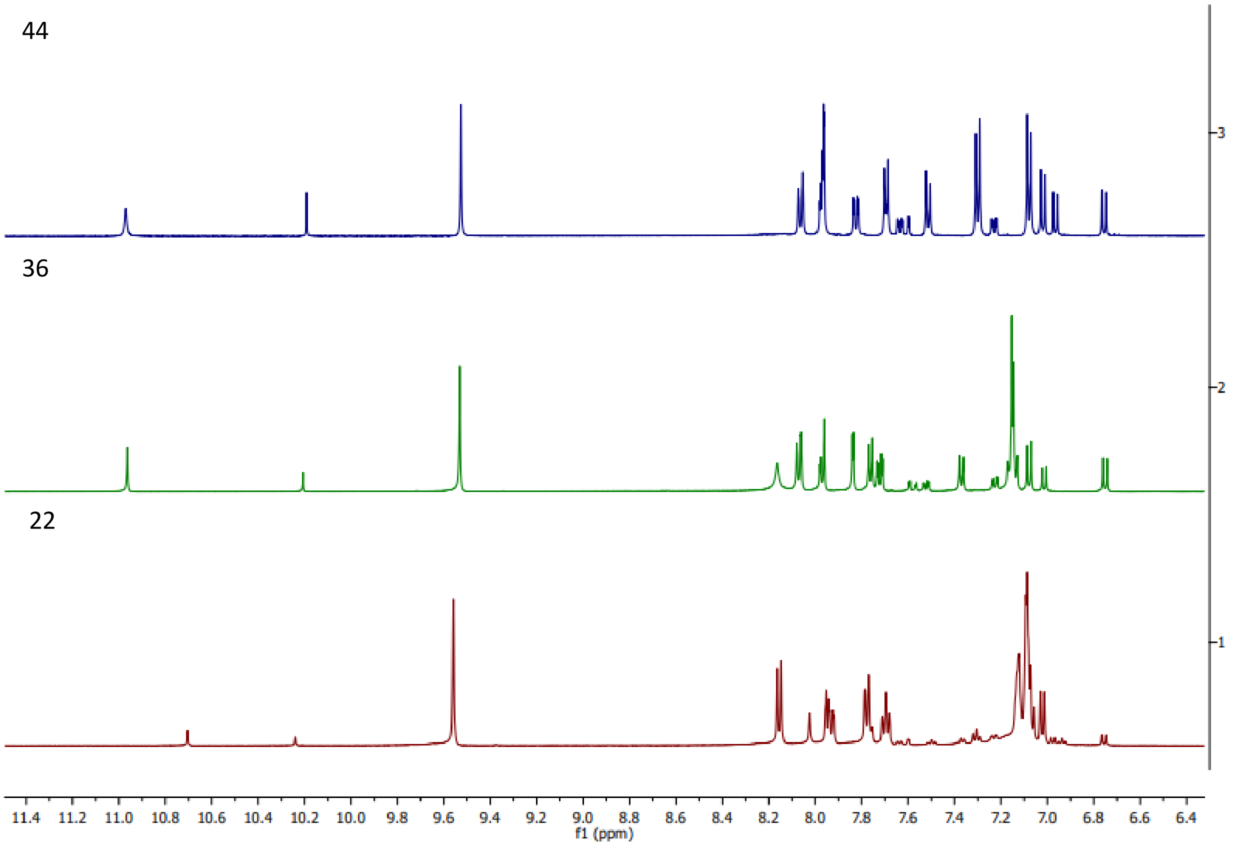

All Br-anthranilic acid (6) derivatives; Top to bottom: 45 / 37 / 23 (after 48h at 4°C)

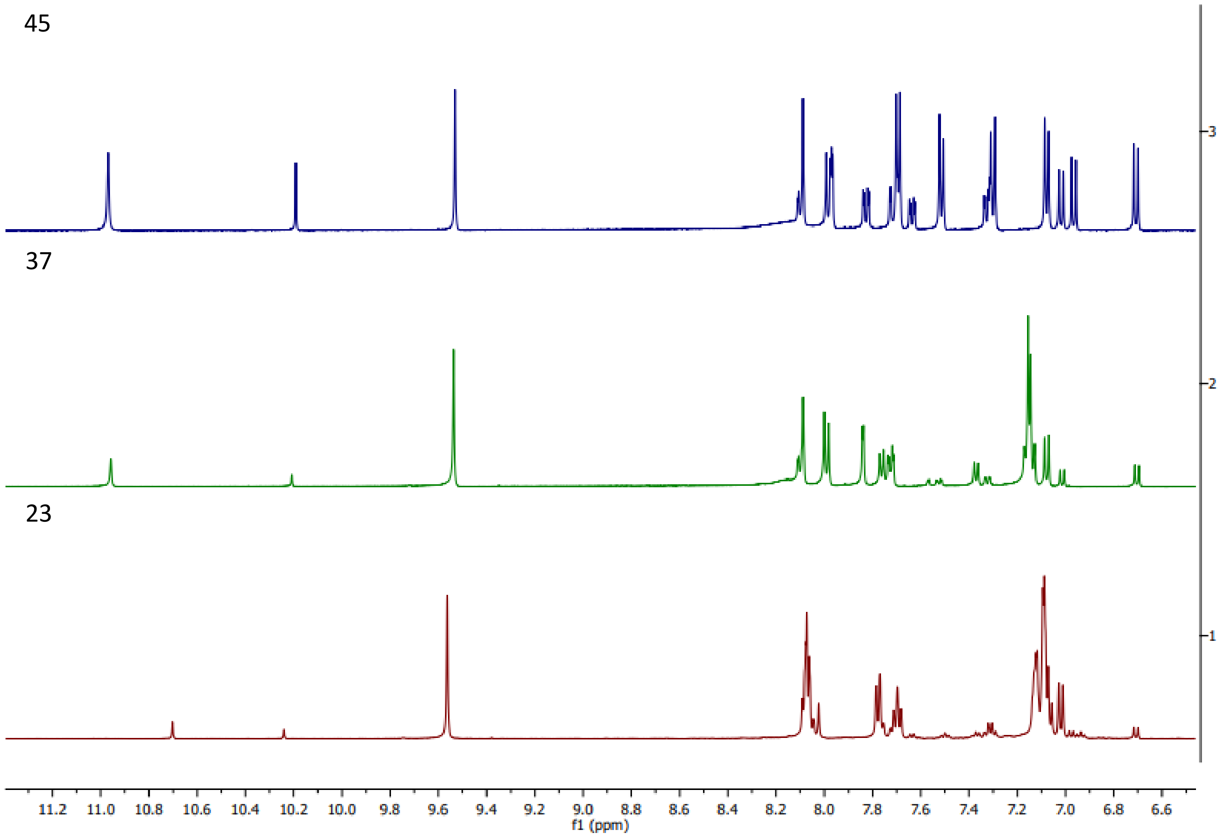

S.4. Time dependent comparative NMR spectra of BASAN and BACAN derived from the same anthranilic acid

All H-anthranilic acid (1) derivatives; Top to bottom: 28/ 18 (after 48h at 4°C)

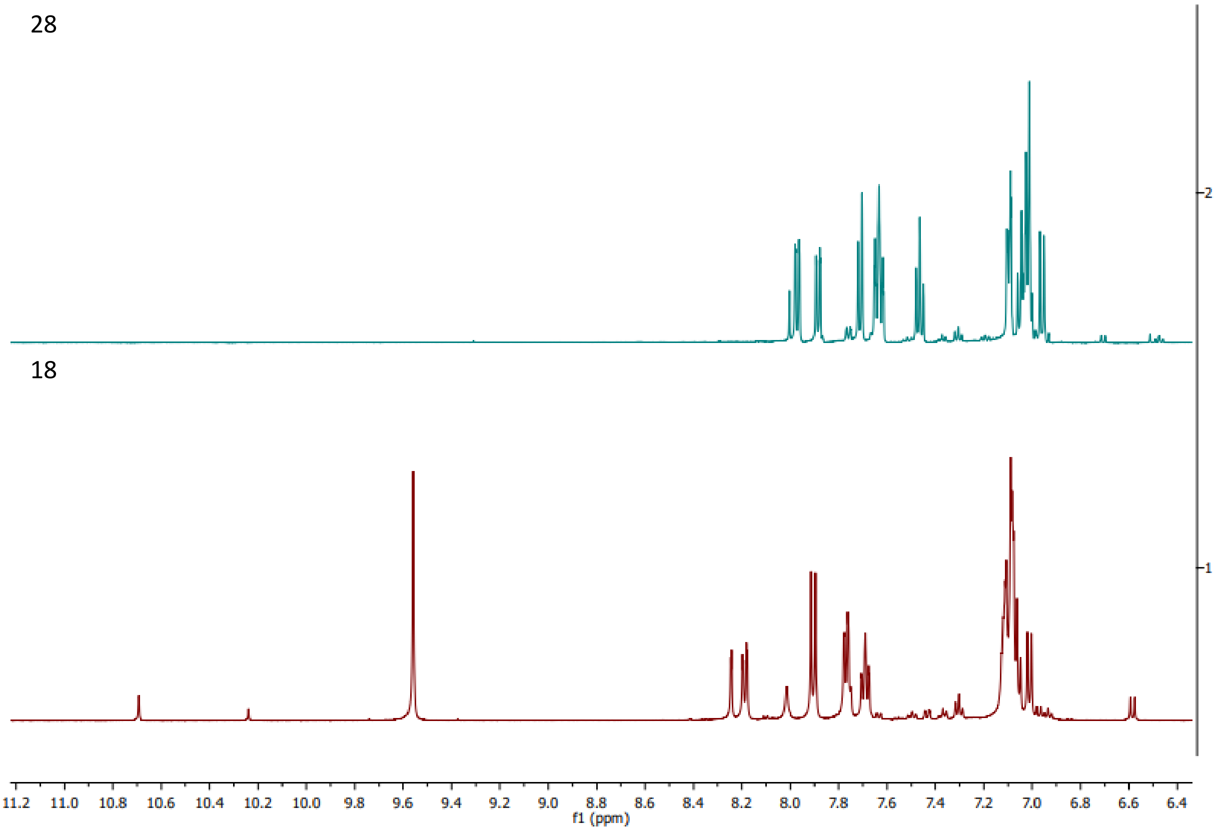

All Me-anthranilic acid (2) derivatives; Top to bottom: 29/ 19 (after 48h at 4°C)

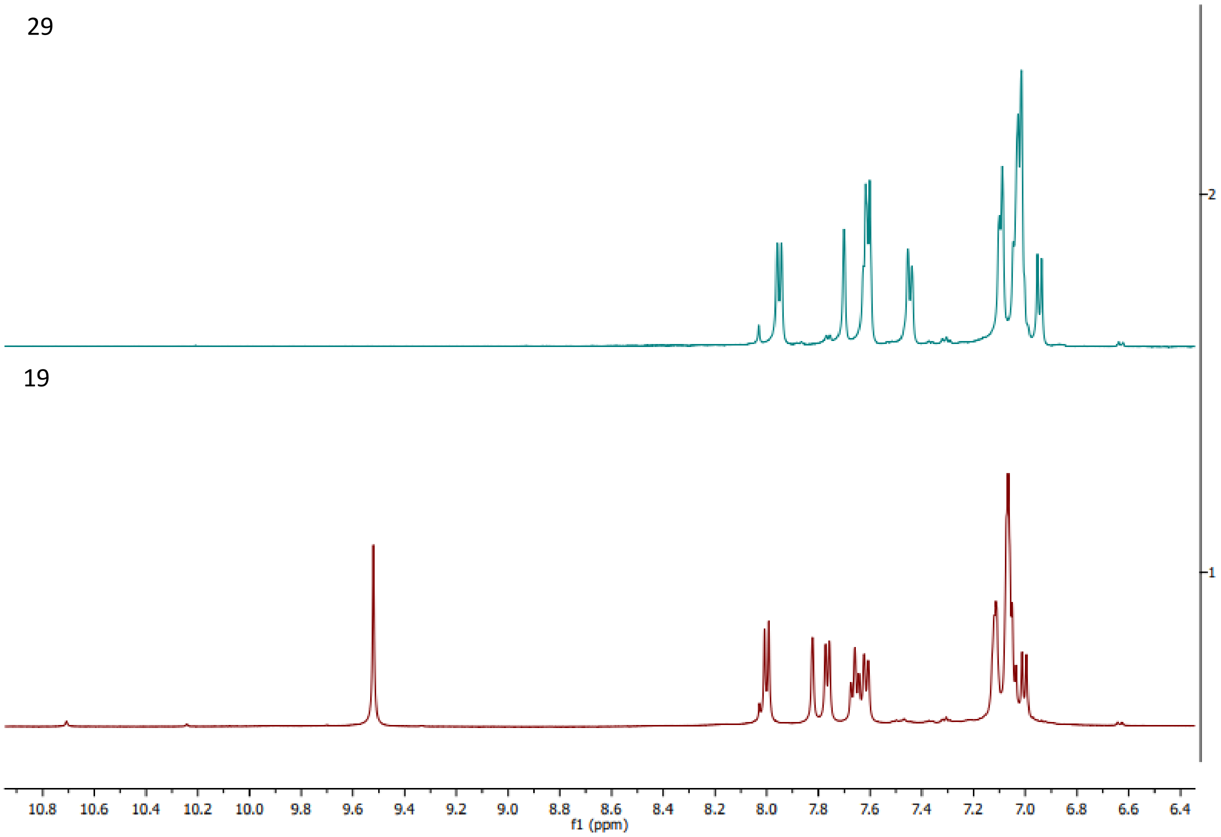

All F-anthranilic acid (4) derivatives; Top to bottom: 30/ 21 (after 48h at 4°C)

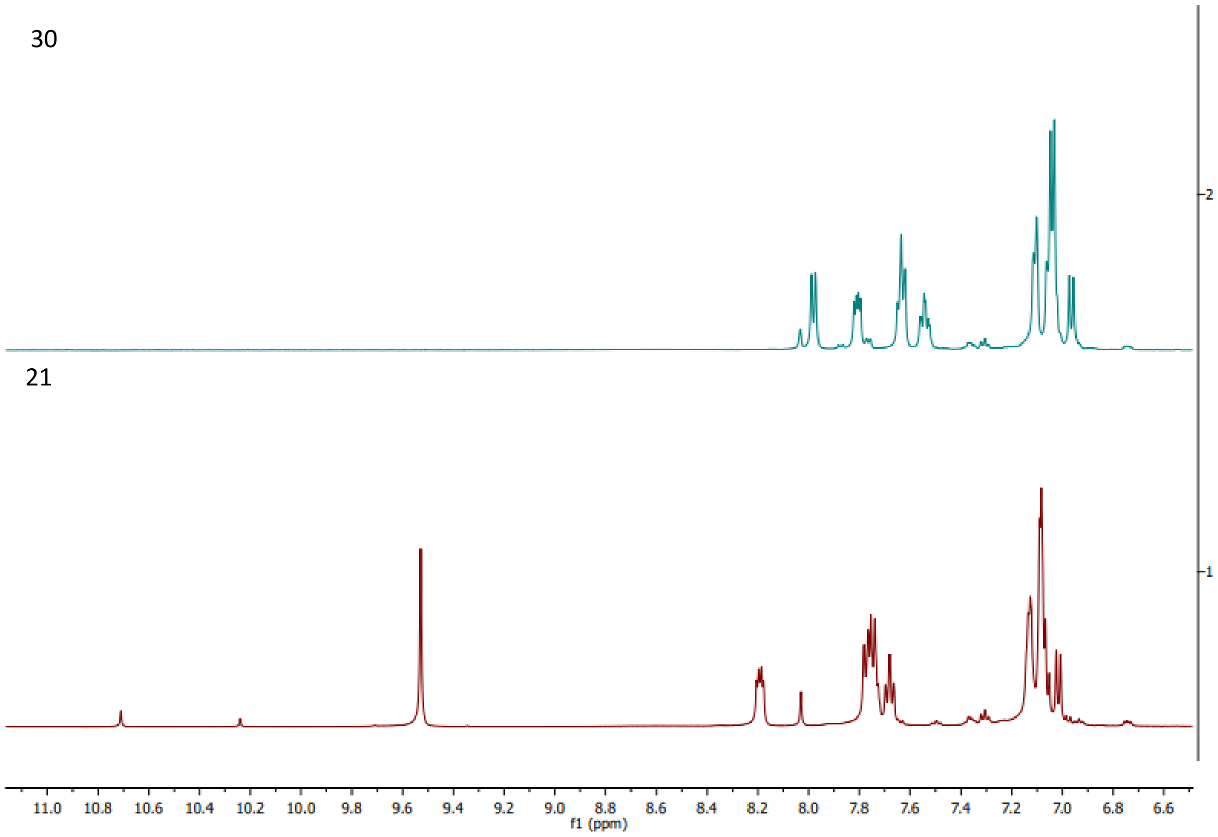

All Cl-anthranilic acid (5) derivatives; Top to bottom: 31/22 (after 48h at 4°C)

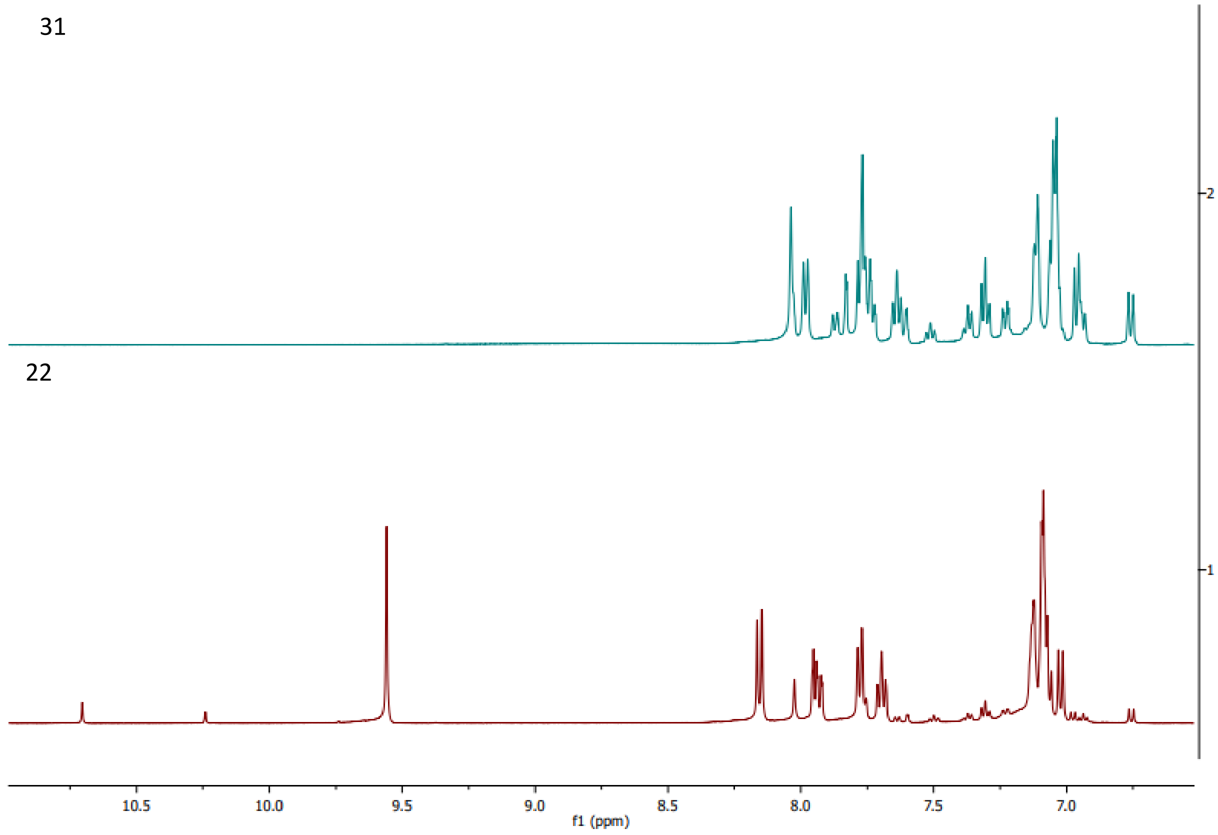

All Br-anthranilic acid (6) derivatives; Top to bottom: 32 / 23 (after 48h at 4°C)

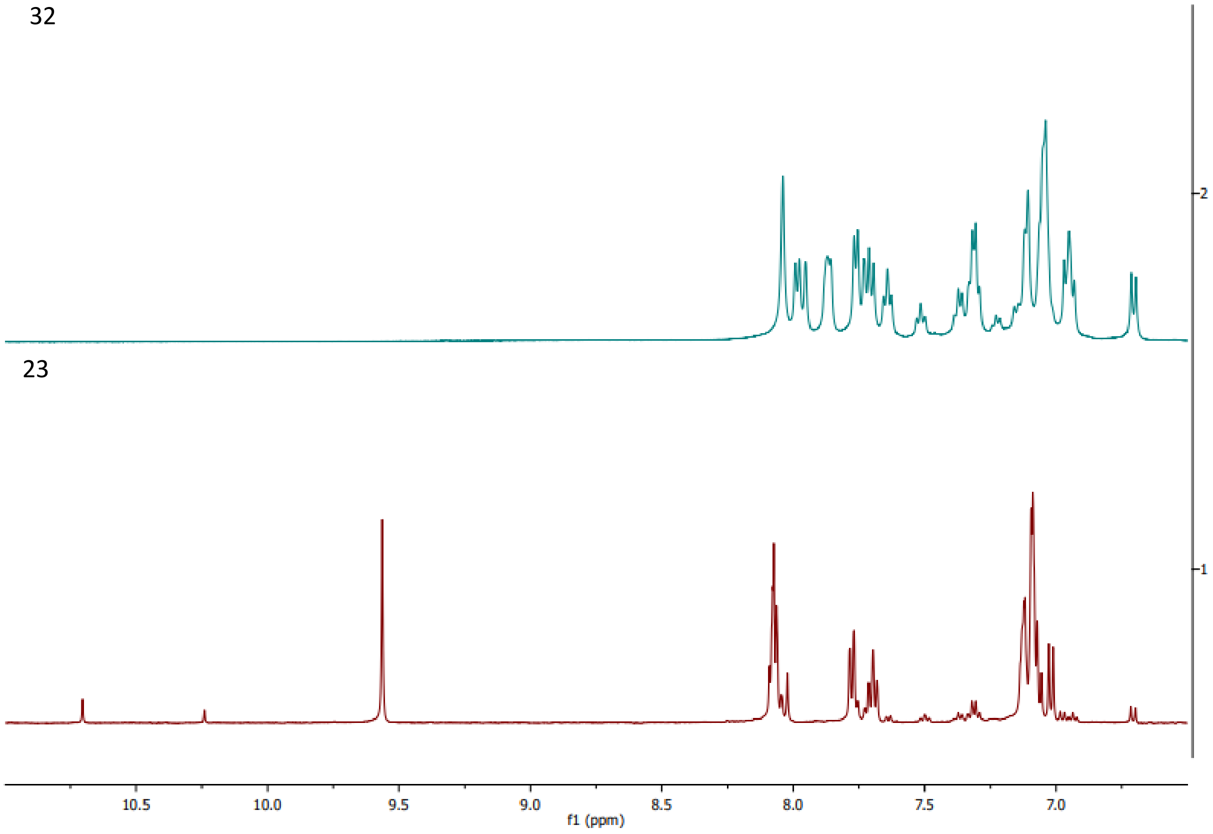

Supplement: Supplementary file 1 [file ijms-25-09842-s001.zip › IJMS_Paisidis_Fylaktakidou_SI_part-1_21072024.pdf]
