# Supplementary material for: One-Pot, Multi-Component Green Microwave-Assisted Synthesis of Bridgehead Bicyclo[4.4.0]boron Heterocycles and DNA Affinity Studies
Source: Int J Mol Sci. 2024 Sep 12;25(18):9842. doi: 10.3390/ijms25189842 (PMC11432172; doi:10.3390/ijms25189842)
Supplement: Supplementary file 1 [file ijms-25-09842-s001.zip › IJMS_Paisidis_Fylaktakidou_SI_part-2_21072024.pdf]

Supporting Information part 2

One-Pot Multi-Component Green Microwave Assisted Synthesis of Bridgehead Bicyclo[4.4.0]boron Heterocycles and DNA Affinity Studies

Polinikis Paisidis,[1] Maroula G. Kokotou,[2] Antigoni Kotali,[3] George Psomas,[4] Konstantina C. Fylaktakidou[1,*]

[1] Laboratory of Organic Chemistry, Aristotle University of Thessaloniki, Chemistry Department of Chemistry, 54124 Thessaloniki, Greece, email: ppaisidis@chem.auth.gr
[2] Laboratory of Chemistry, Department of Food Science and Human Nutrition, Agricultural University of Athens, Iera Odos 75, Athens 11855, Greece, email: mkokotou@aua.gr
[3] Laboratory of Organic Chemistry, Aristotle University of Thessaloniki, Department of Chemical Engineering, 54124 Thessaloniki, Greece, email: kotali@cheng.auth.gr
[4] Laboratory of Inorganic Chemistry, Aristotle University of Thessaloniki, Department of Chemistry, 54124 Thessaloniki, Greece, email: gepsomas@chem.auth.gr
Corresponding Author: email: kfylakta@chem.auth.gr

Table of contents

S.1. UV-vis spectra of compounds 18-24, 26-48 in DMSO 3

S.2. Interaction with CT DNA 8

S.2.1 Protocols concerning the interaction studies 8

S.2.1.1 Binding studies with CT DNA by UV-vis spectroscopy 8

S.2.1.2 CT DNA-binding studies by viscosity measurements 8

S.2.1.3 EB-displacement studies 8

S.2.1.4 References 9

S.2.2 Interaction with CT DNA studied with UV-vis spectroscopy 10

S.2.2.1 UV-vis spectra of DMSO solution of compounds 18-24, 26-48 in the presence of increasing amounts of CT DNA 10

S.2.2.2 Plot of [DNA]/(ε_A-ε_F) *versus* [DNA] for compounds 18-24, 26-48. 14

S.2.3 EB-displacement studies with fluorescence spectroscopy 18

S.2.3.1 Fluorescence emission spectra (λ_{ex} = 540 nm) for EB-DNA ([EB] = 20 μM, [DNA] = 26 μM) in buffer solution in the absence and presence of increasing amounts of compounds 18-24, 26-48 18

S.2.3.2 Stern-Volmer quenching plot of EB-DNA fluorescence for compounds 18-24, 26-48 22

S.3. Fluorescence excitation and emission spectra of compounds 18-24, 26-48 26

S.1. UV-vis spectra of compounds 18-24, 26-48 in DMSO

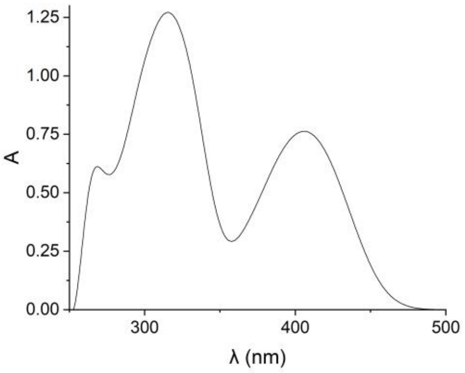

Compound 18

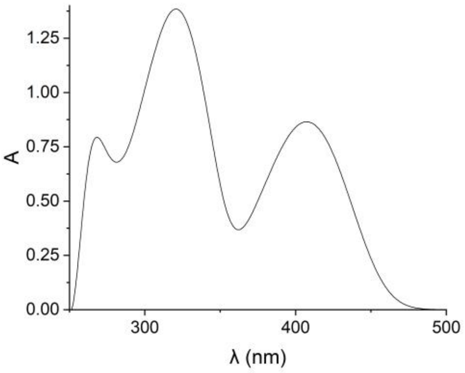

Compound 19

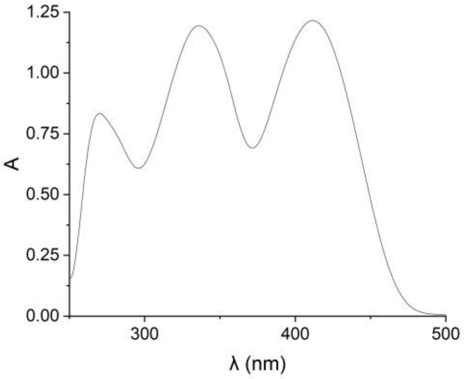

Compound 20

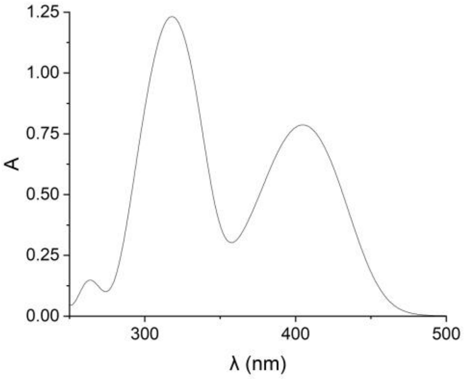

Compound 21

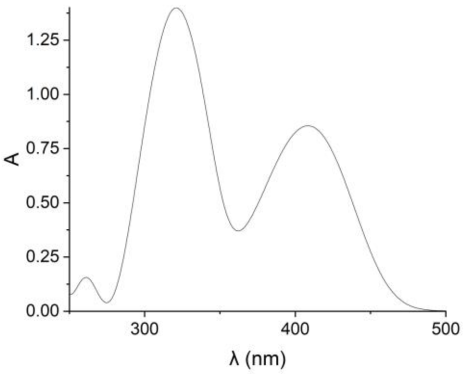

Compound 22

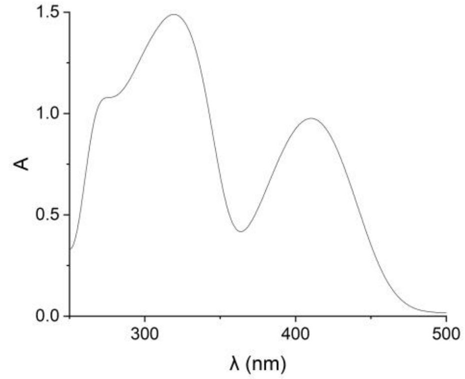

Compound 23

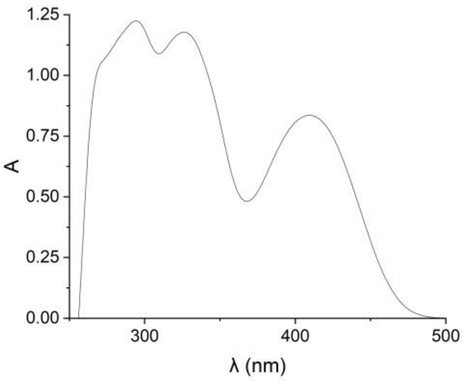

Compound 24

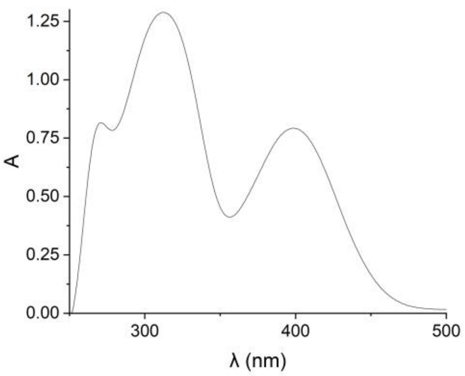

Compound 26

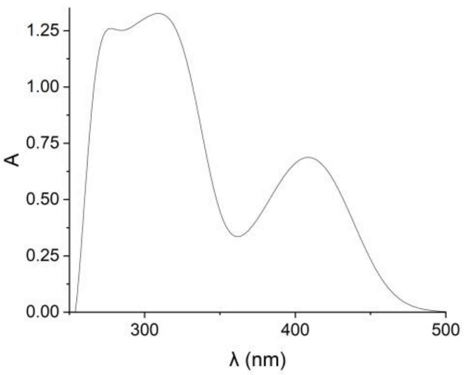

Compound 27

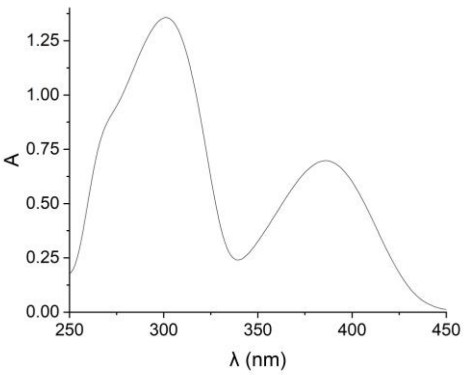

Compound 28

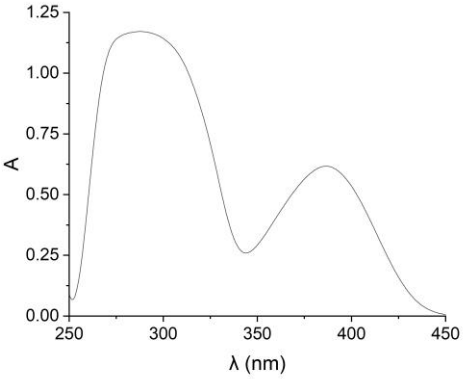

Compound 29

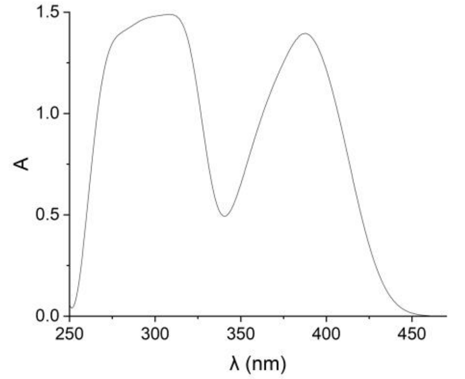

Compound 30

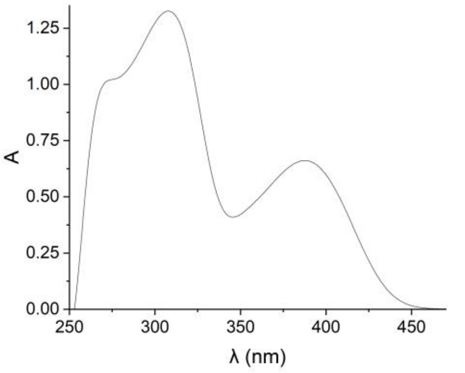

Compound 31

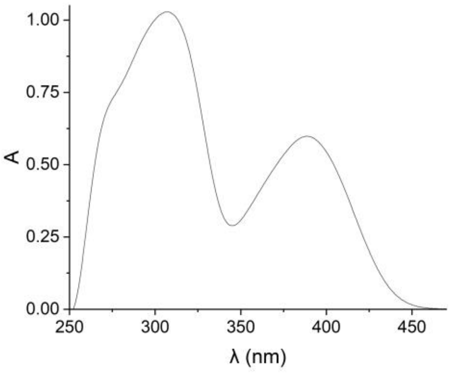

Compound 32

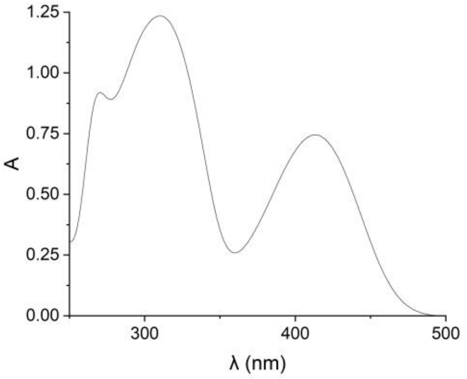

Compound 33

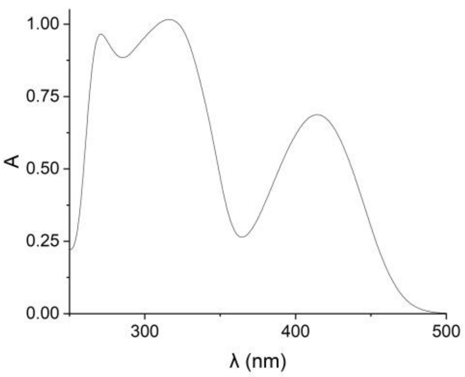

Compound 34

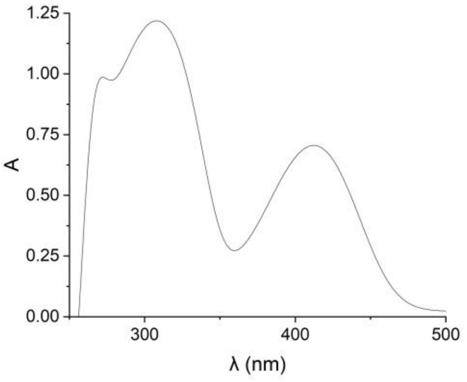

Compound 35

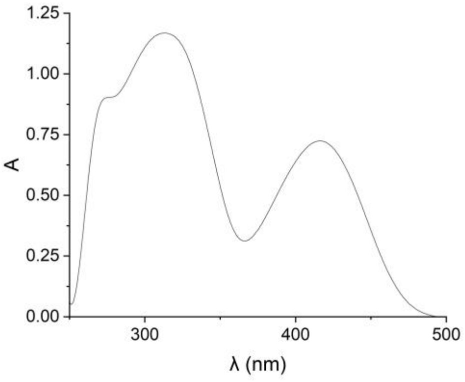

Compound 36

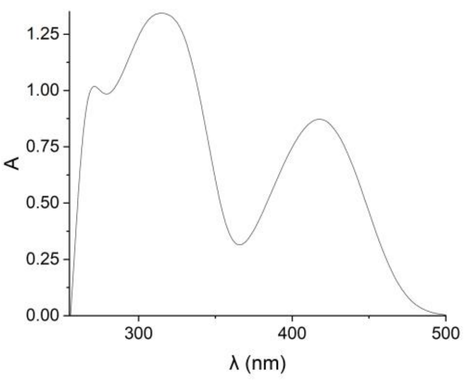

Compound 37

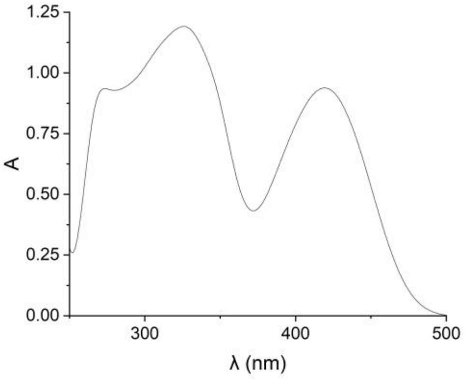

Compound 38

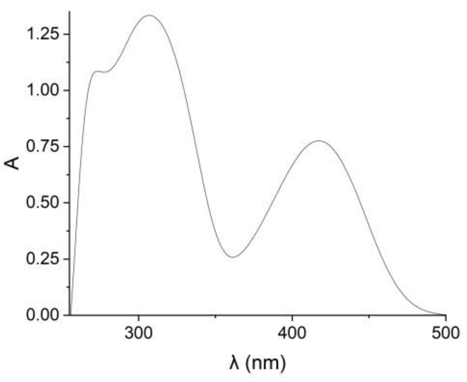

Compound 39

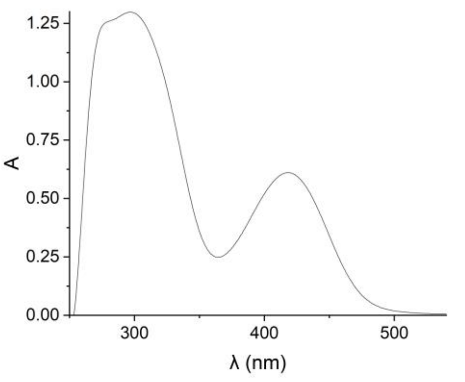

Compound 40

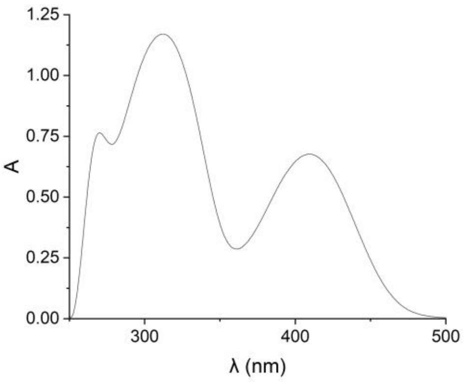

Compound 41

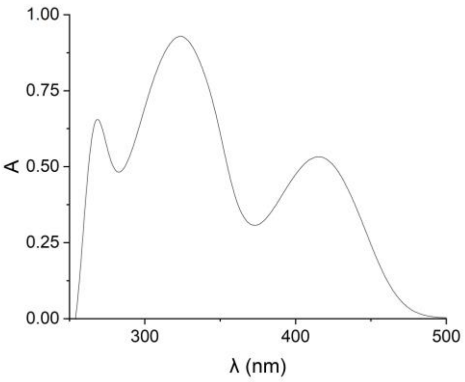

Compound 42

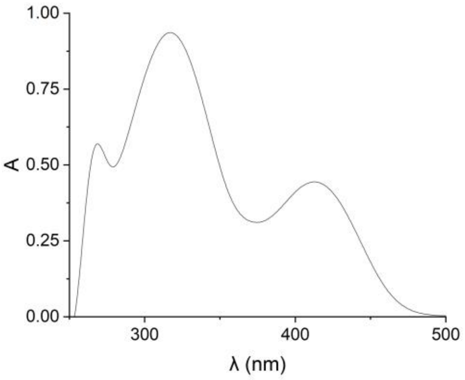

Compound 43

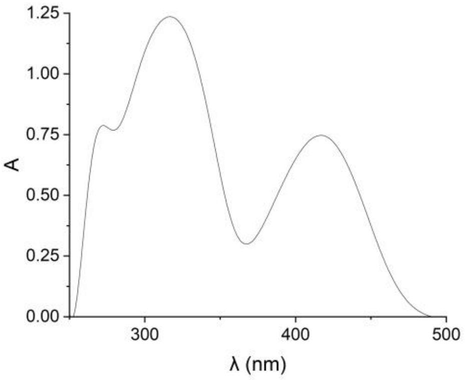

Compound 44

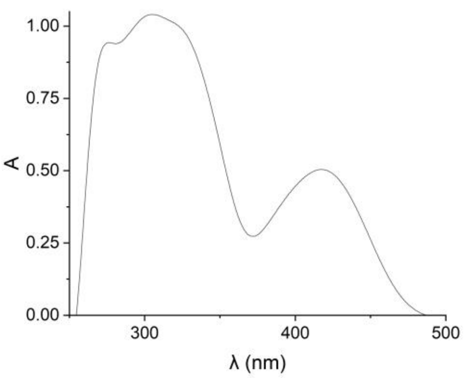

Compound 45

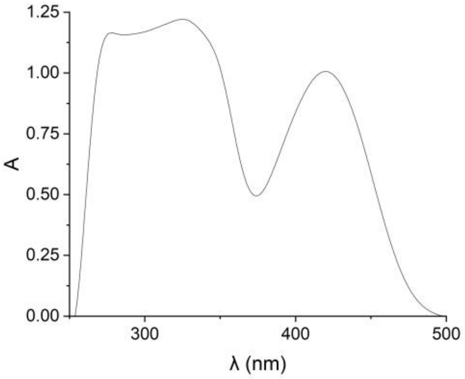

Compound 46

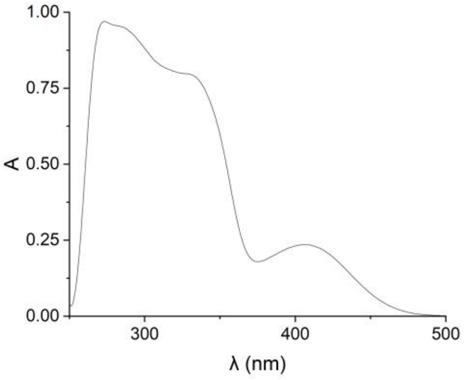

Compound 47

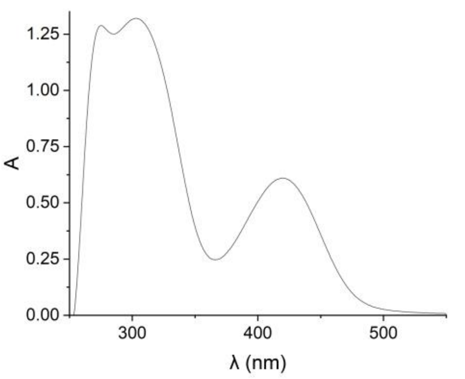

Compound 48

S.2. Interaction with CT DNA

The interaction of the compounds with CT DNA was investigated by UV-vis spectroscopy, viscosity measurements and fluorescence emission spectroscopy studies.

S.2.1 Protocols concerning the interaction studies

S.2.1.1 Binding studies with CT DNA by UV-vis spectroscopy

UV-vis spectroscopy was used for the evaluation of the interaction of the compounds with CT DNA, and specifically the possible binding modes of the compounds to CT DNA. Control experiments with DMSO were performed and no changes in the spectra of CT DNA were observed.

In order to determine the binding mode, the UV-vis spectra of the compounds were recorded for a constant concentration (5×10^{-5} – 10^{-4} M) with increasing concentrations of CT DNA for diverse *r* (*r* = [compound]/[DNA]) values. Effective use of the changes in the absorbance of the UV-vis spectra was made and the DNA-binding constants of the compounds (K_b, in M^{-1}) were calculated by the Wolfe-Shimer equation (eq. S1) [1] and the plots [DNA]/(ε_A–ε_f) *versus* [DNA]:

$$\frac{[DNA]}{(\varepsilon_A-\varepsilon_f)}=\frac{[DNA]}{(\varepsilon_b-\varepsilon_f)}+\frac{1}{K_b(\varepsilon_b-\varepsilon_f)} \qquad \text{(eq S1)}$$

where [DNA] = the concentration of DNA in base pairs, ε_f = the extinction coefficient for the free compound at the corresponding λ_{max}, ε_A = A_{obsd}/[compound] and ε_b = the extinction coefficient for the compound in the fully bound form. K_b is given by the ratio of slope to the y intercept in plots [DNA]/(ε_A–ε_f) *versus* [DNA].

S.2.1.2 CT DNA-binding studies by viscosity measurements

The viscosity of DNA (0.1 mM) in buffer solution was measured in the absence and presence of increasing amounts of the compounds. The experiments were executed at room temperature and the measurements are devised in a plot $(\eta/\eta_0)^{1/3}$ *versus* r (*r* = [compound]/[DNA]), where η = the viscosity of DNA in the presence of the compound, and η_0 = the viscosity of DNA in buffer solution.

S.2.1.3 EB-displacement studies

In order to determine and confirm the DNA-binding mode of the compounds, a competitive study with EB, as an intercalating marker, is performed by fluorescence emission spectroscopy. Therefore, the EB-displacing ability of the compounds from its EB-DNA adduct was examined.

The DNA-EB adduct was prepared by addition of 20 μM EB and 26 μM CT DNA in buffer solution (150 mM NaCl and 15 mM trisodium citrate at pH 7.0). The potential intercalation of the compounds between the DNA-bases was studied by the addition of a certain amount of the compound solution into the EB-DNA adduct solution. The influence of the compounds on the EB-DNA solution was monitored through the changes of the fluorescence emission spectra at excitation wavelength (λ_{ex}) at 540 nm [2]. The tested compounds do not show any significant fluorescence at room temperature in solution or in the presence of DNA, under the same experimental conditions (λ_{ex} = 540 nm). Bearing that in mind, the observed quenching of the EB-DNA solution is evidently associated to the displacement of EB from its EB-DNA adduct.

The quenching efficiency (K_{SV}) for each compound was assessed according to the Stern-Volmer equation (eq. S2) [2]:

$$\frac{Io}{I} = 1 + K_q \tau_0 [Q] = 1 + K_{SV} [Q] \qquad \text{(eq. S2)}$$

where I_0 and I = the fluorescence emission intensities of EB-DNA in the absence and presence of the quencher (i.e. compound), respectively, [Q] = the concentration of the quencher. K_{SV} is obtained from the Stern-Volmer plots by the slope of the diagram I_0/I *versus* [Q]. Taking τ_0 = 23 ns as the fluorescence lifetime of the EB-DNA adduct [3], the EB-DNA quenching constant (K_q, in $M^{-1}s^{-1}$) of the compounds can be determined according to equation S3:

$$K_{SV} = K_q \tau_o \qquad \text{(eq. S3)}$$

S.2.1.4 References

1. Wolfe, A.; Shimer, G.H.; Meehan, T. Polycyclic Aromatic Hydrocarbons Physically Intercalate into Duplex Regions of Denatured DNA. *Biochemistry* **1987**, *26*, 6392–6396, doi:10.1021/bi00394a013.
2. Lakowicz, J.R. *Principles of Fluorescence Spectroscopy*; Springer New York, NY, 2006; ISBN 978-0-387-31278-1.
3. Heller, D.P.; Greenstock, C.L. Fluorescence lifetime analysis of DNA intercalated ethidium bromide and quenching by free dye. *Biophys. Chem.* **1994**, *50*, 305–312, doi:10.1016/0301-4622(93)E0101-A.

S.2.2 Interaction with CT DNA studied with UV-vis spectroscopy

S.2.2.1 UV-vis spectra of DMSO solution of compounds 18-24, 26-48 in the presence of increasing amounts of CT DNA

Black curve is the UV-vis spectrum of the compound without CT DNA. The arrows show the changes upon increasing amounts of CT DNA.

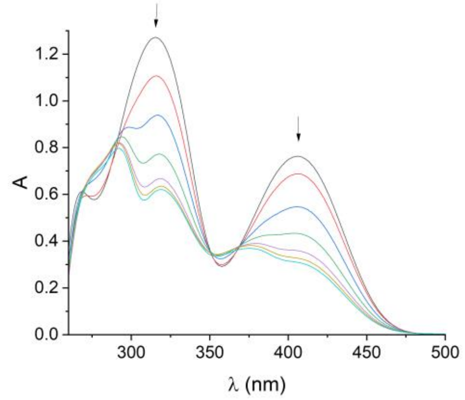

Compound 18

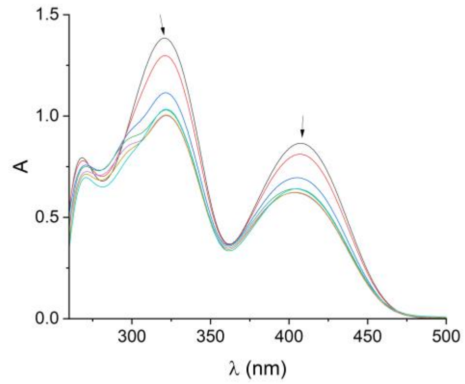

Compound 19

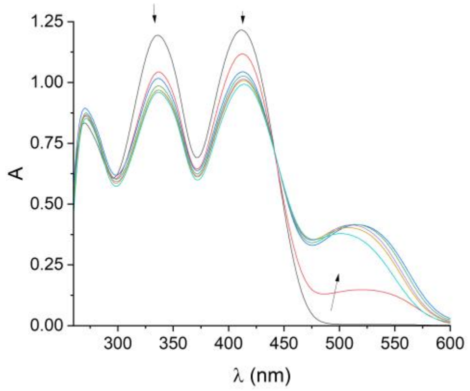

Compound 20

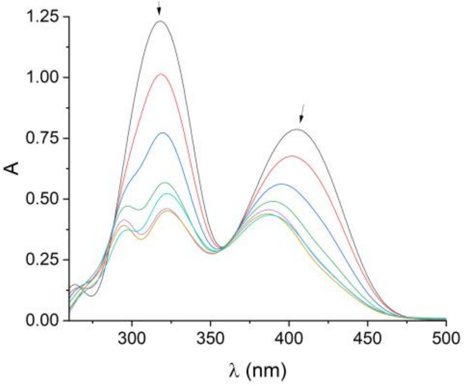

Compound 21

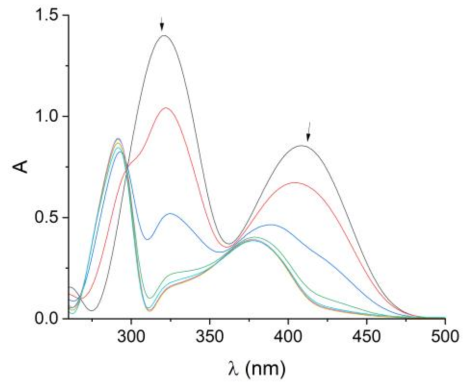

Compound 22

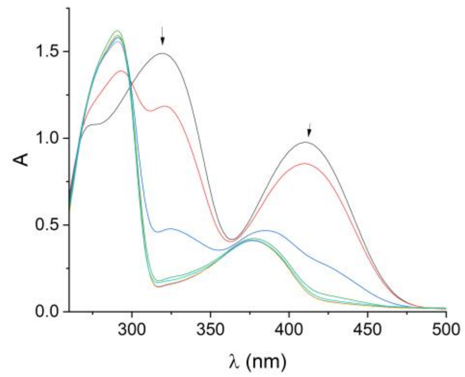

Compound 23

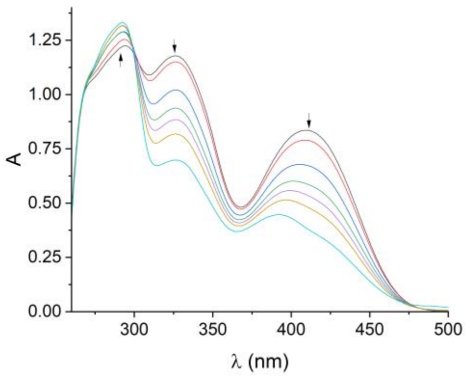

Compound 24

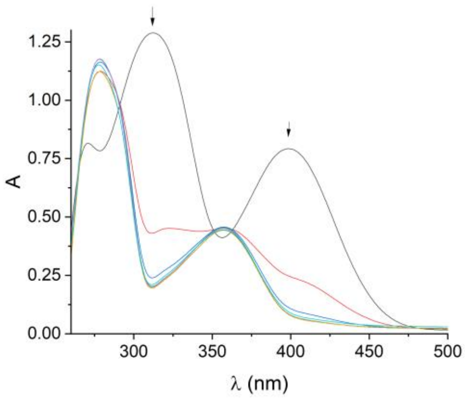

Compound 26

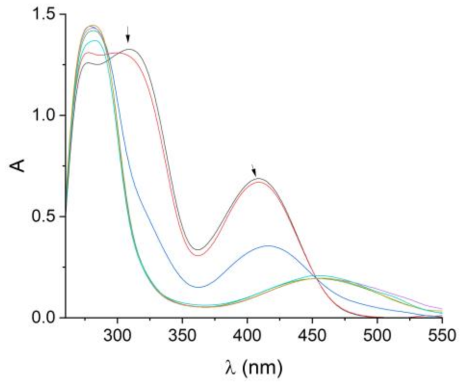

Compound 27

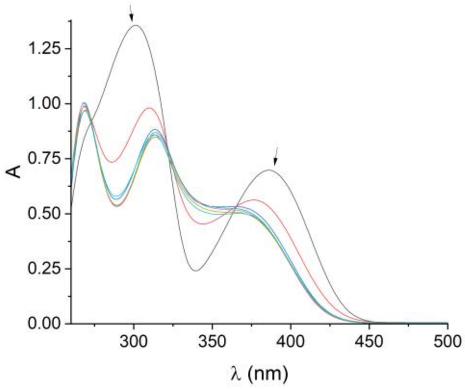

Compound 28

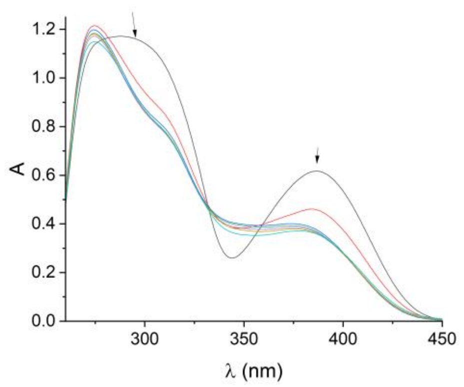

Compound 29

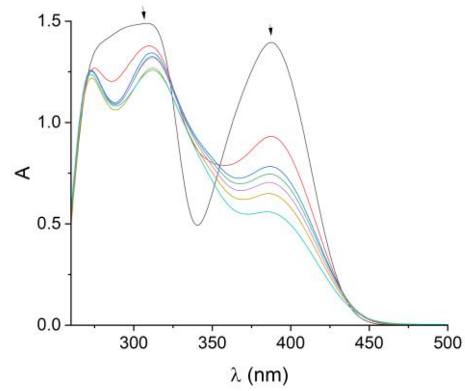

Compound 30

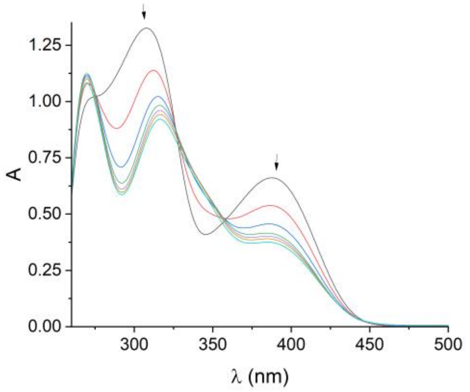

Compound 31

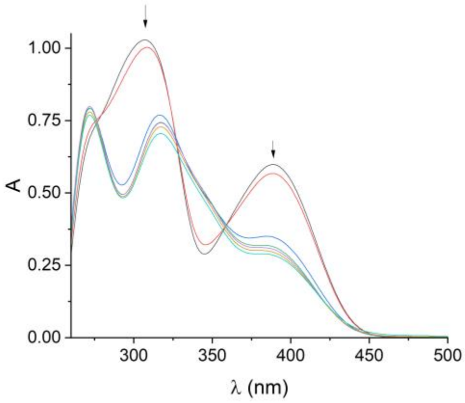

Compound 32

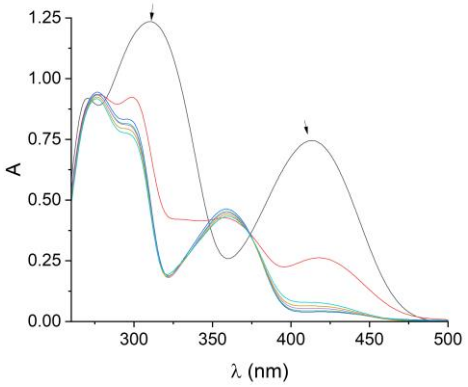

Compound 33

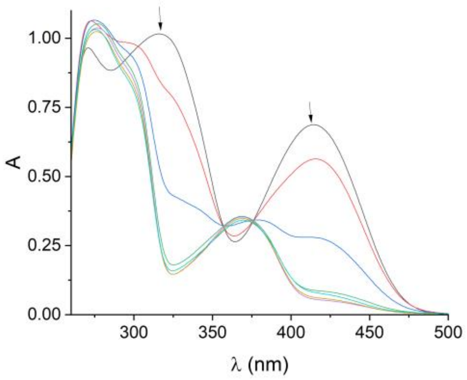

Compound 34

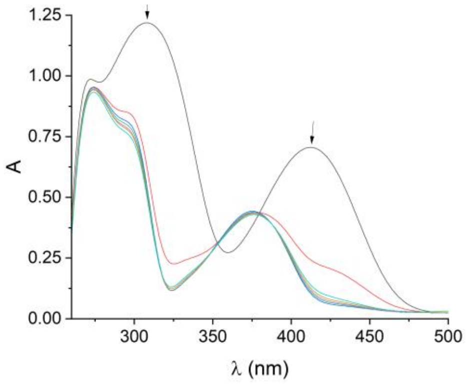

Compound 35

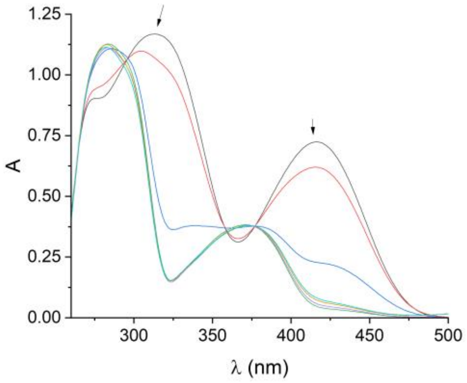

Compound 36

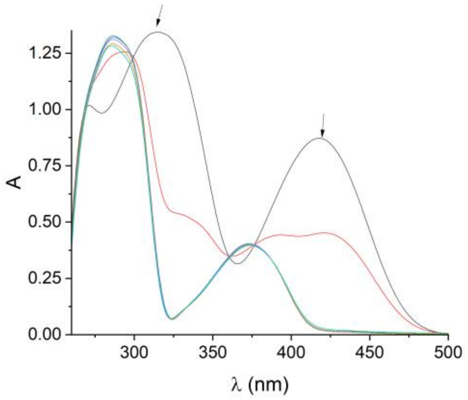

Compound 37

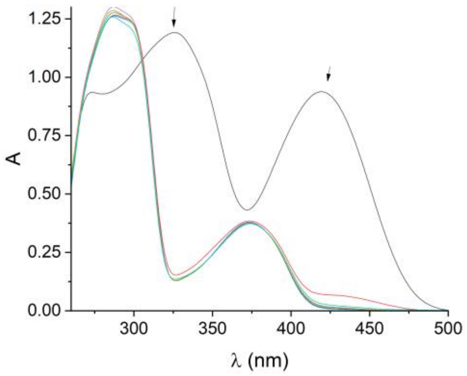

Compound 38

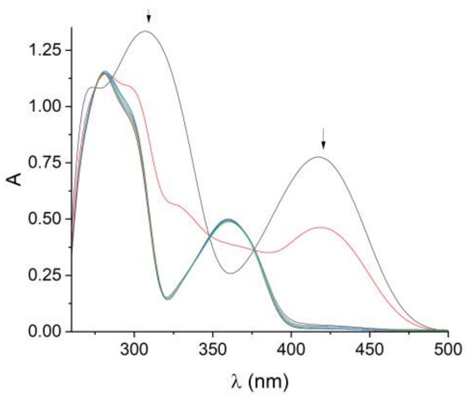

Compound 39

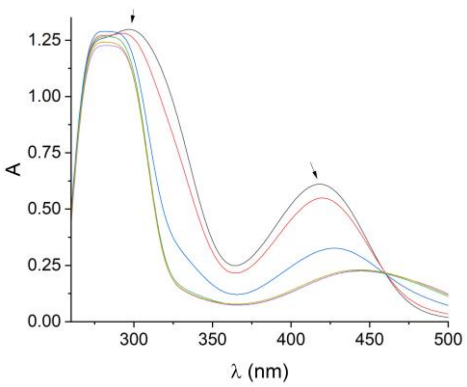

Compound 40

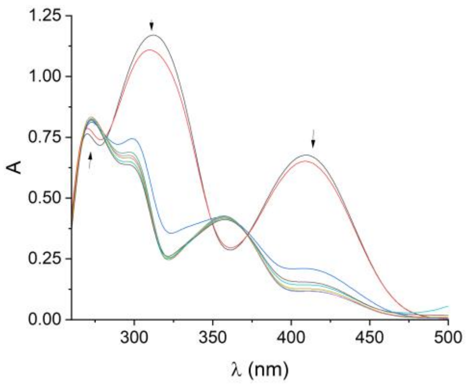

Compound 41

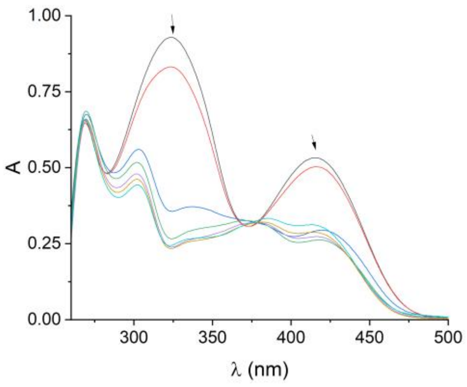

Compound 42

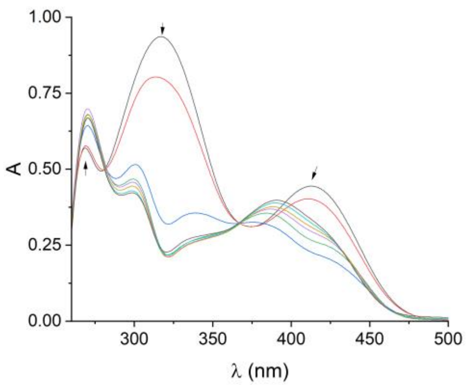

Compound 43

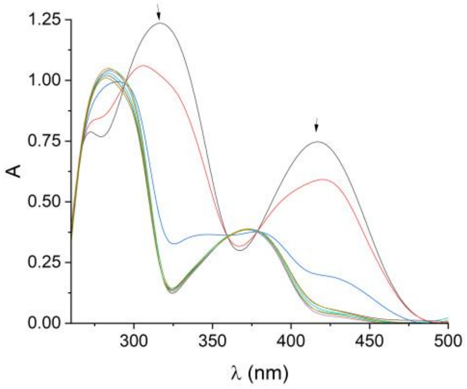

Compound 44

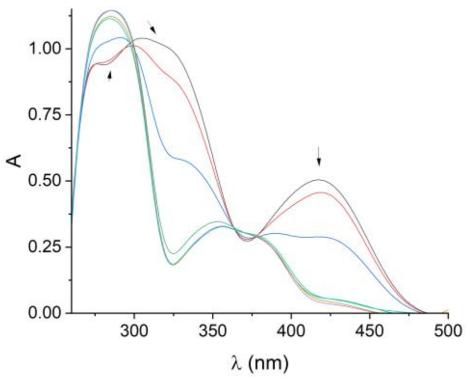

Compound 45

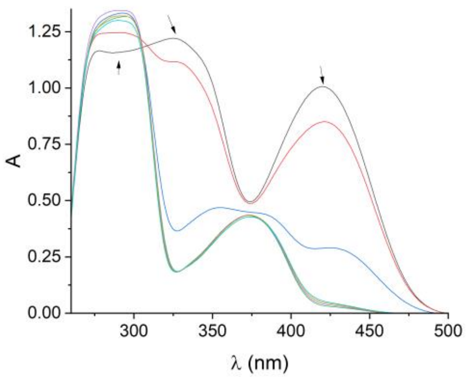

Compound 46

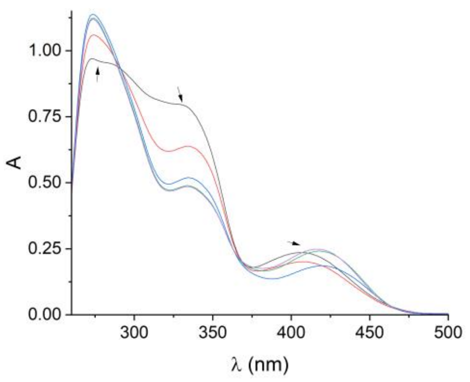

Compound 47

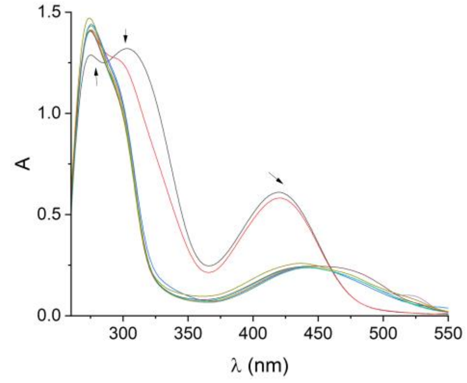

Compound 48

S.2.2.2 Plot of [DNA]/(ε_A-ε_F) *versus* [DNA] for compounds 18-24, 26-48.

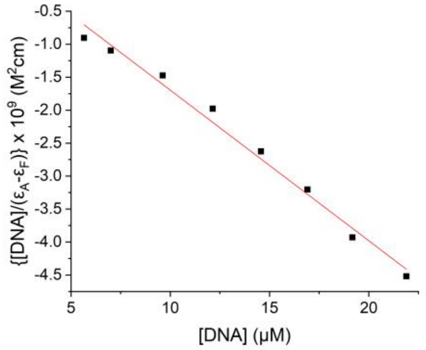

Compound 18

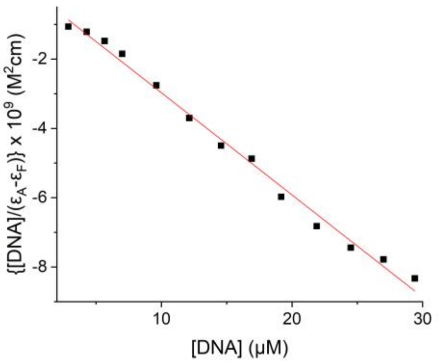

Compound 19

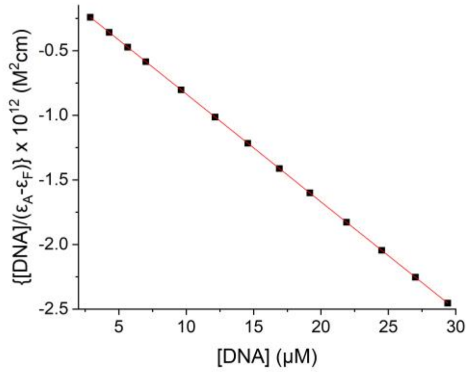

Compound 20

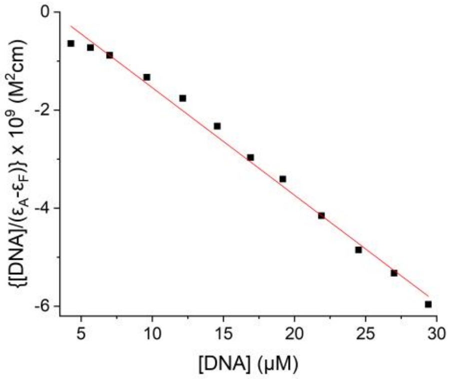

Compound 21

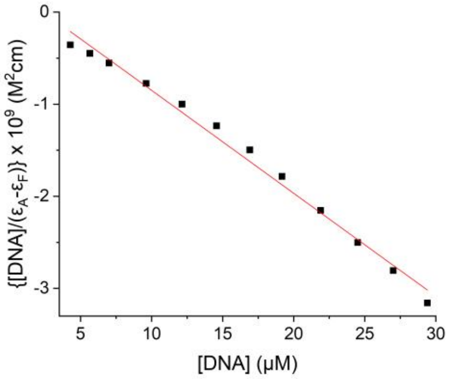

Compound 22

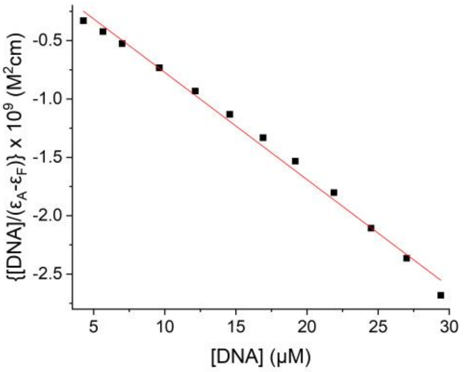

Compound 23

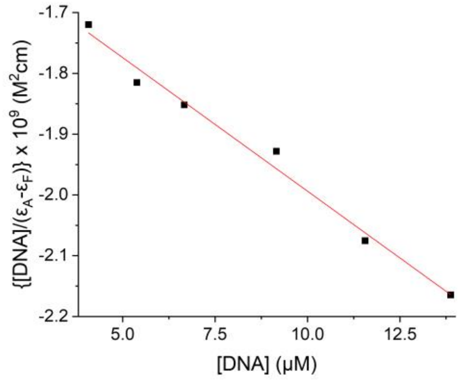

Compound 24

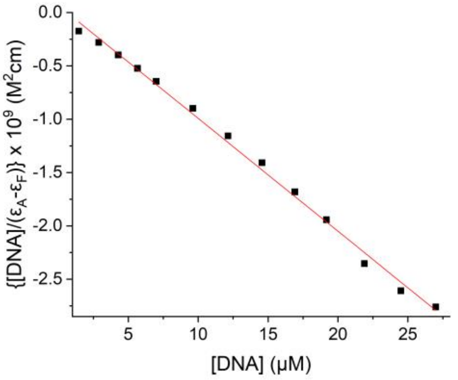

Compound 26

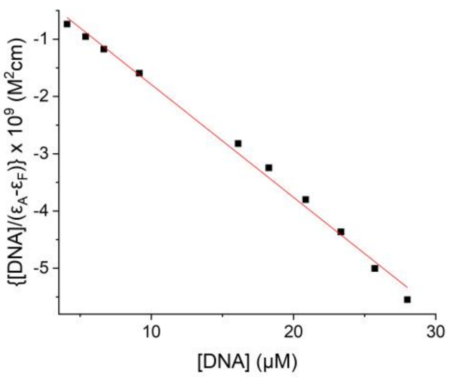

Compound 27

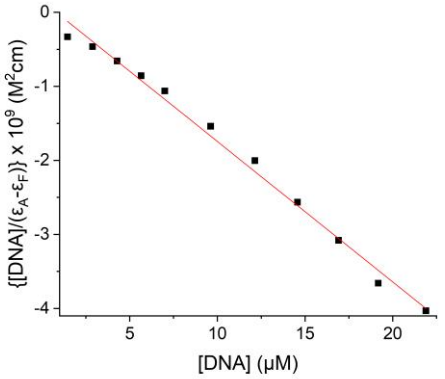

Compound 28

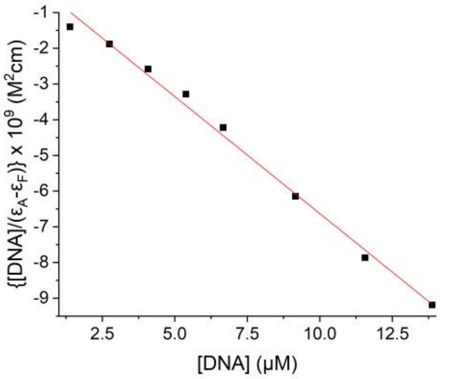

Compound 29

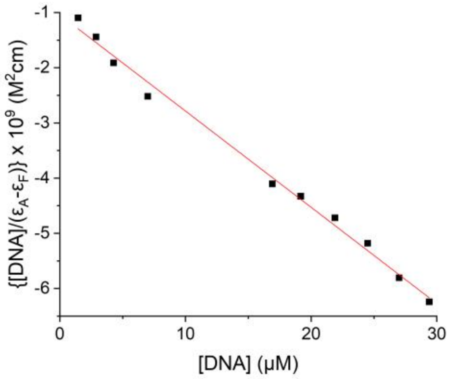

Compound 30

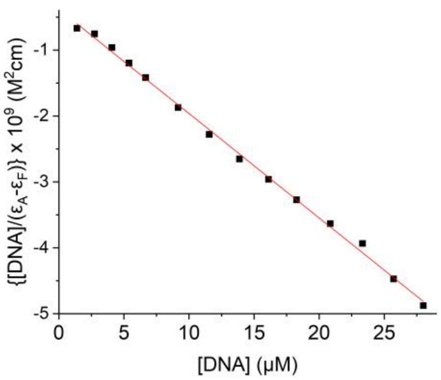

Compound 31

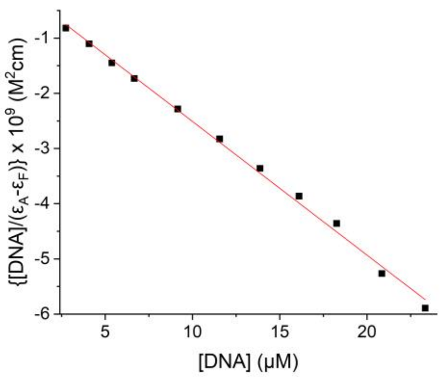

Compound 32

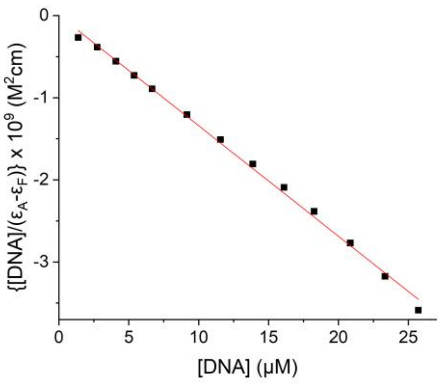

Compound 33

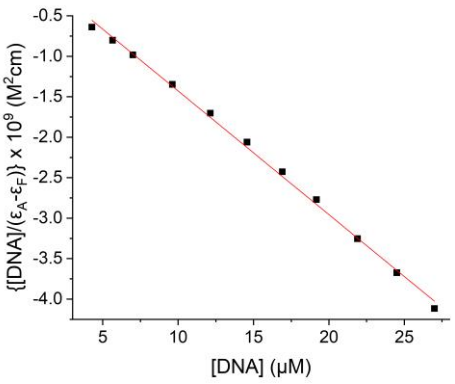

Compound 34

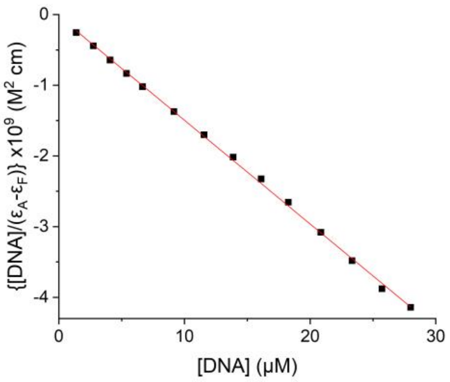

Compound 35

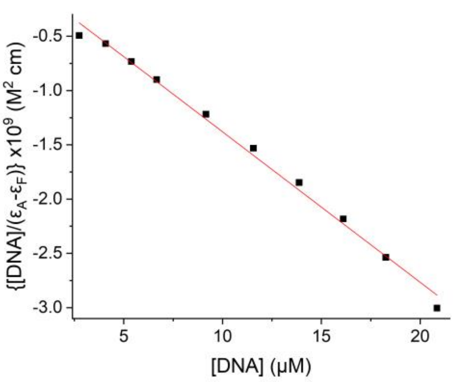

Compound 36

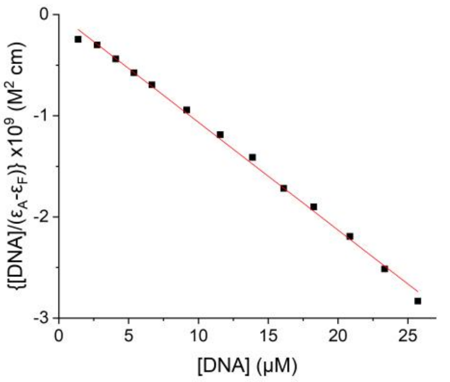

Compound 37

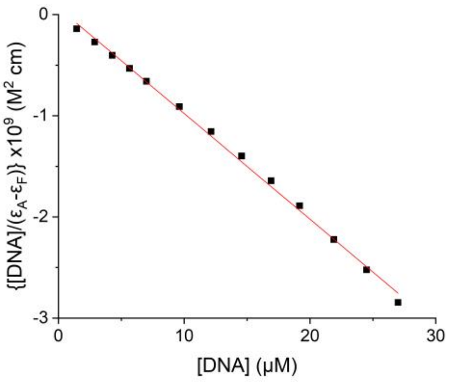

Compound 38

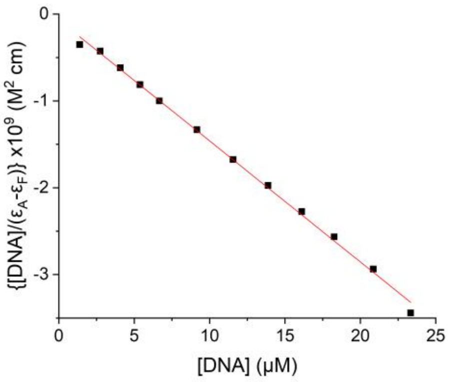

Compound 39

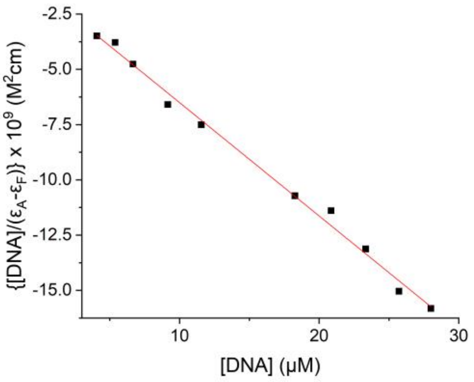

Compound 40

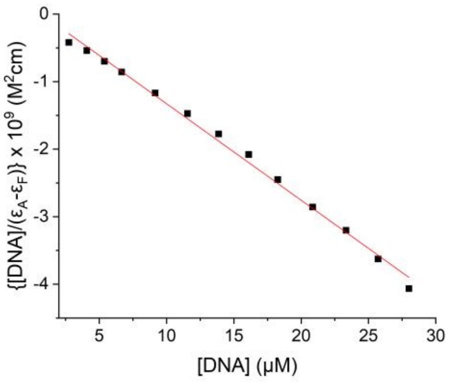

Compound 41

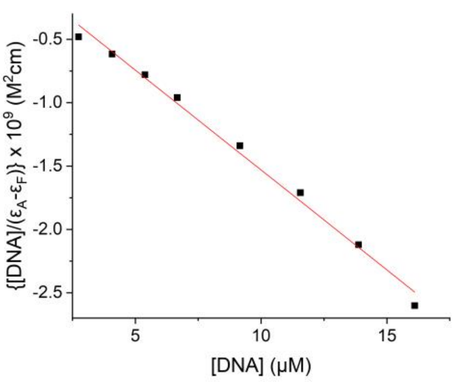

Compound 42

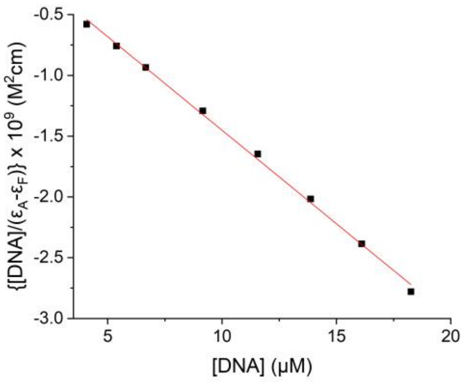

Compound 43

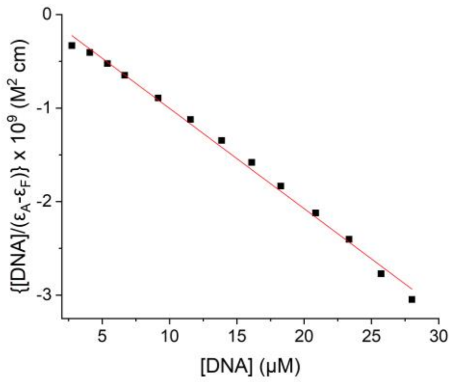

Compound 44

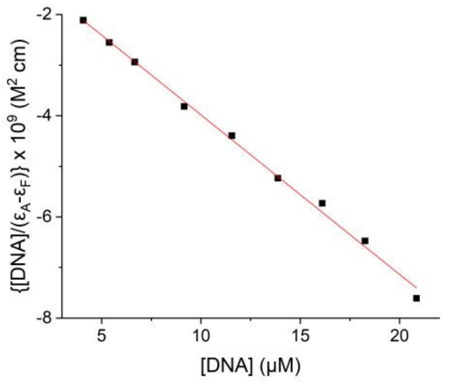

Compound 45

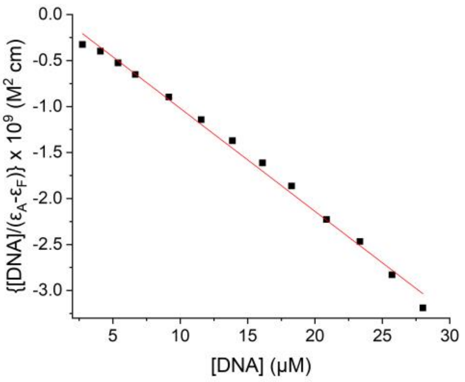

Compound 46

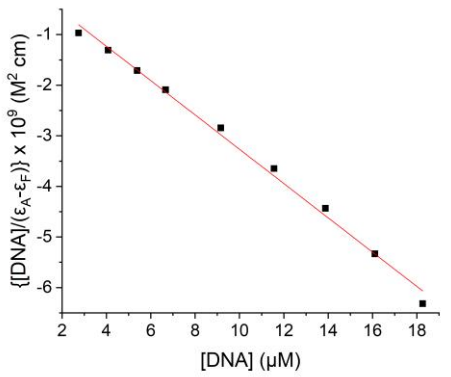

Compound 47

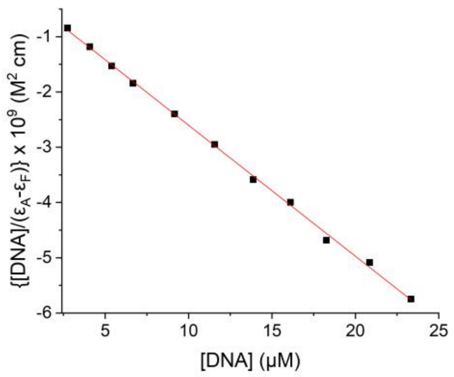

Compound 48

S.2.3 **EB-displacement studies** with fluorescence spectroscopy

S.2.3.1 Fluorescence emission spectra (λ_{ex} = 540 nm) for EB-DNA ([EB] = 20 μM, [DNA] = 26 μM) in buffer solution in the absence and presence of increasing amounts of compounds 18-48

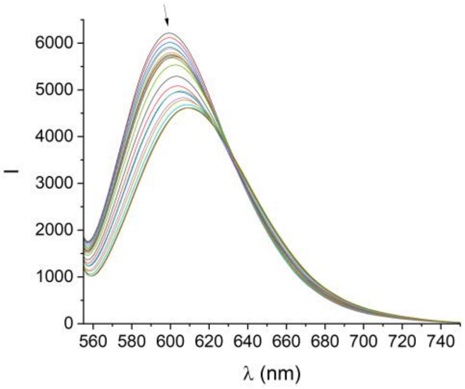

Compound 18

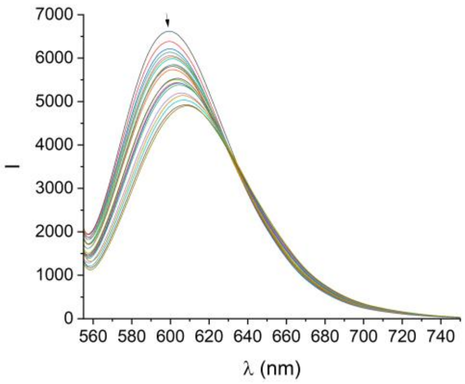

Compound 19

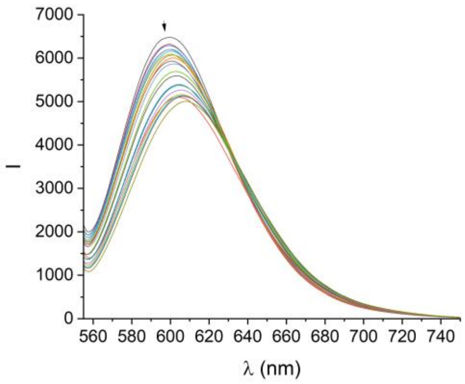

Compound 20

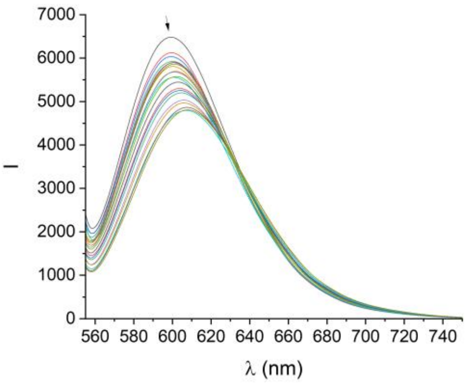

Compound 21

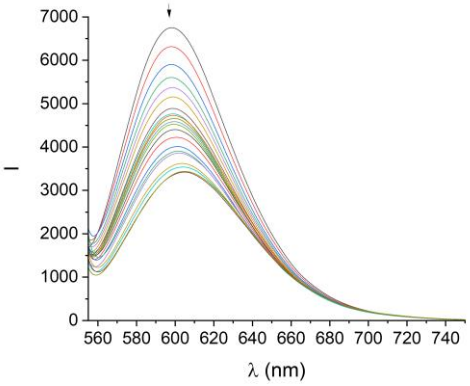

Compound 22

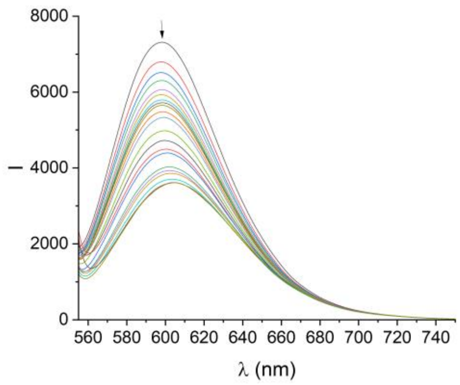

Compound 23

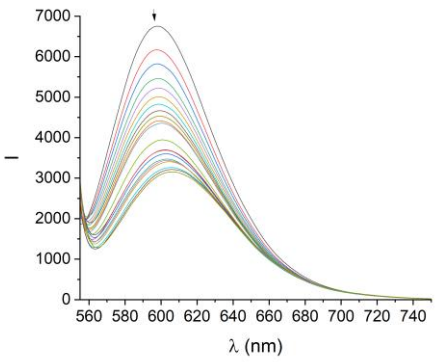

Compound 24

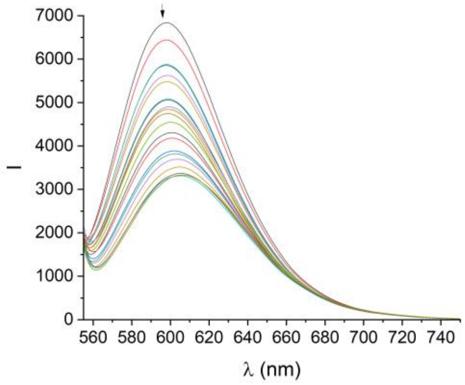

Compound 26

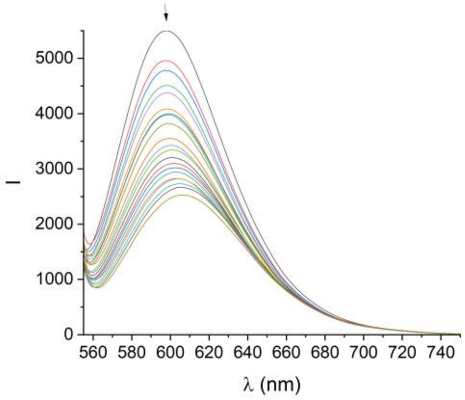

Compound 27

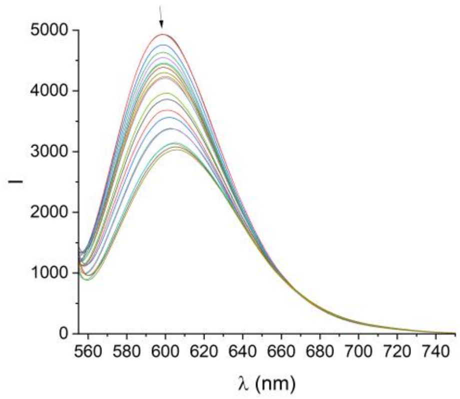

Compound 28

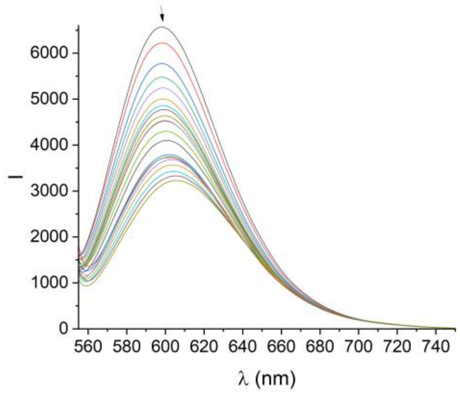

Compound 29

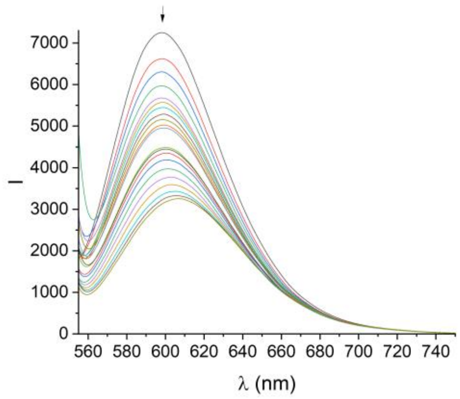

Compound 30

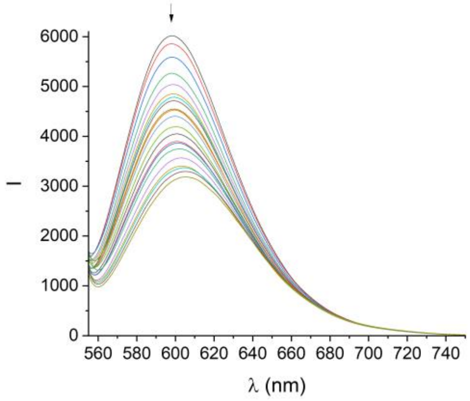

Compound 31

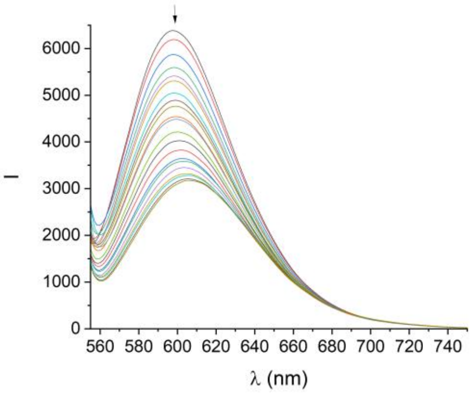

Compound 32

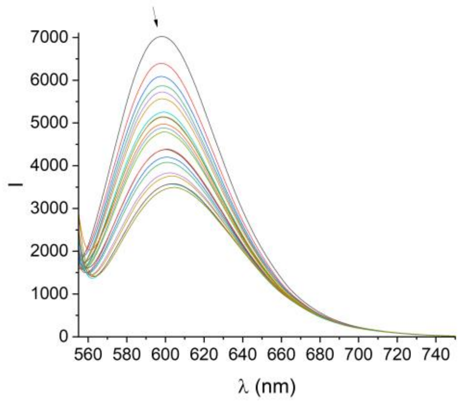

Compound 33

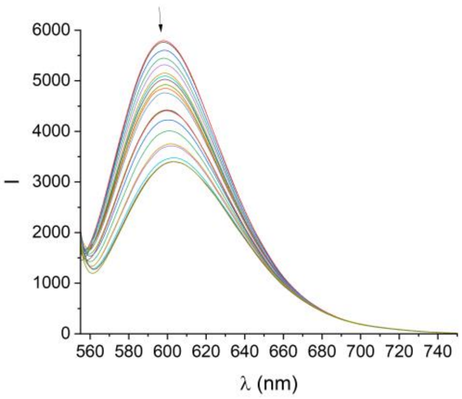

Compound 34

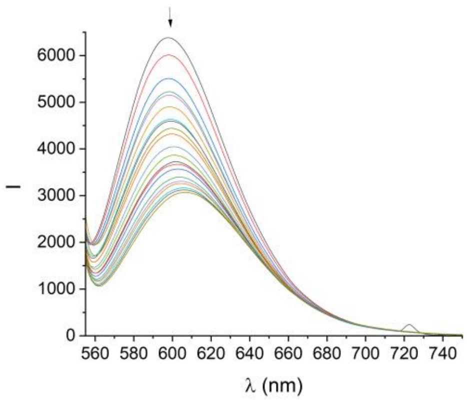

Compound 35

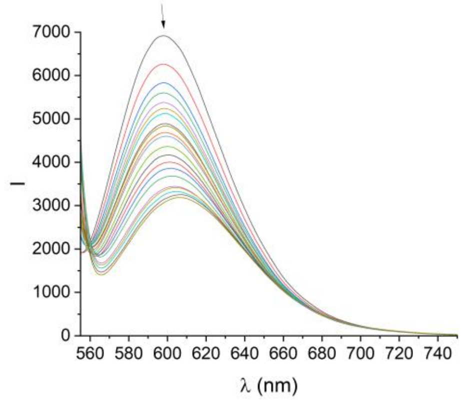

Compound 36

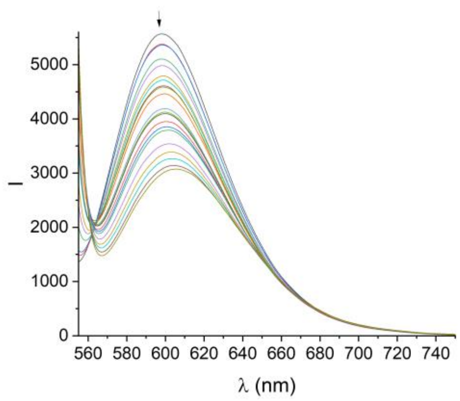

Compound 37

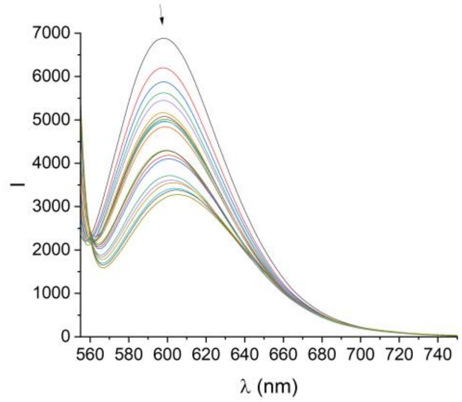

Compound 38

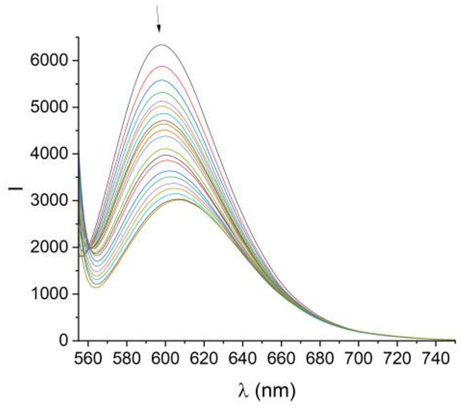

Compound 39

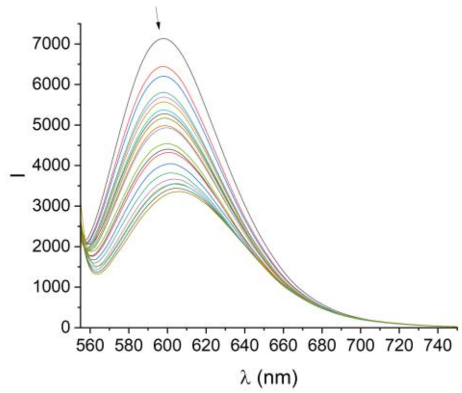

Compound 40

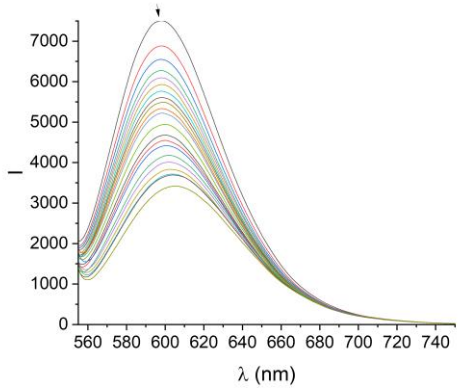

Compound 41

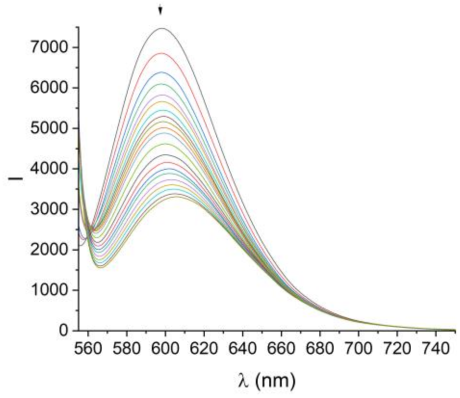

Compound 42

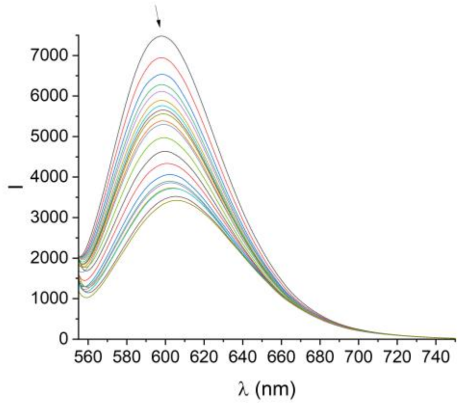

Compound 43

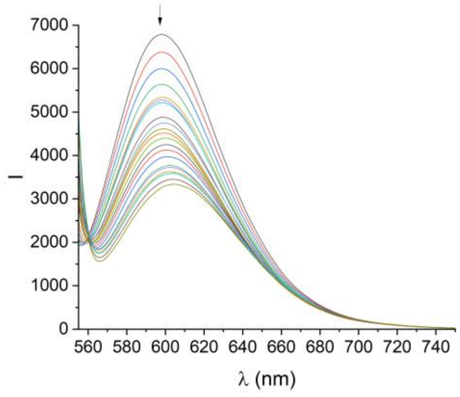

Compound 44

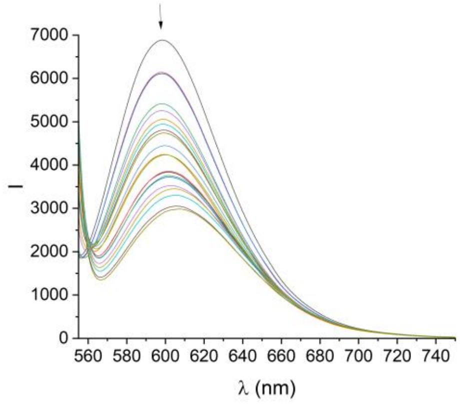

Compound 45

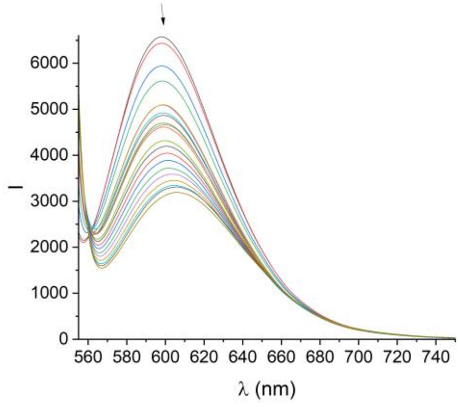

Compound 46

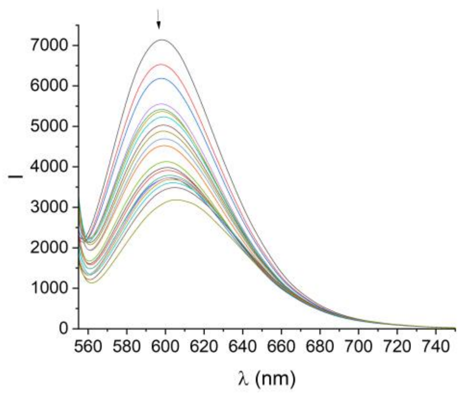

Compound 47

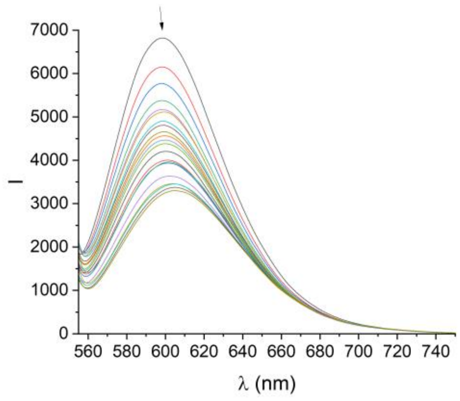

Compound 48

S.2.3.2 Stern-Volmer quenching plot of EB-DNA fluorescence for compounds 18-24, 26-48

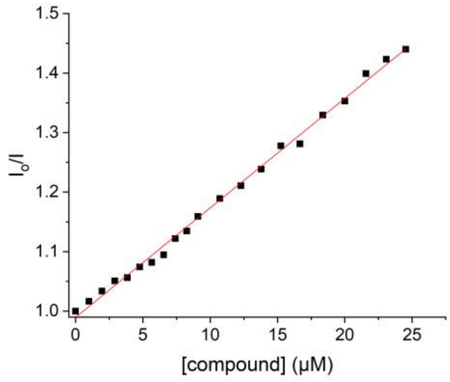

Compound 18

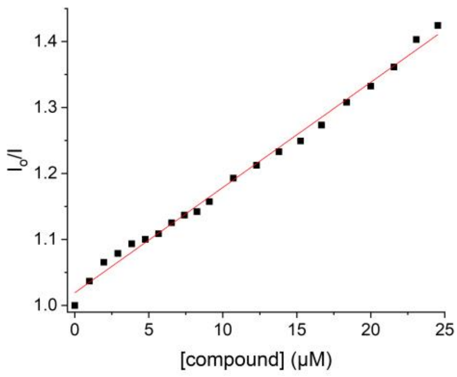

Compound 19

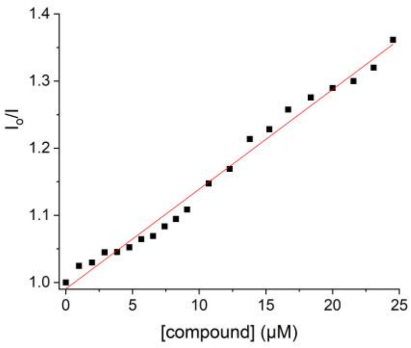

Compound 20

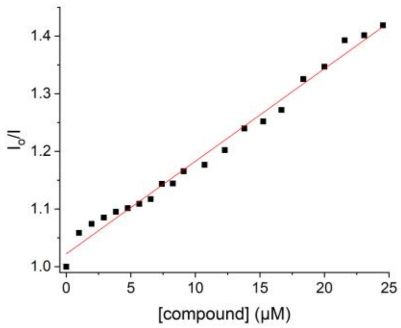

Compound 21

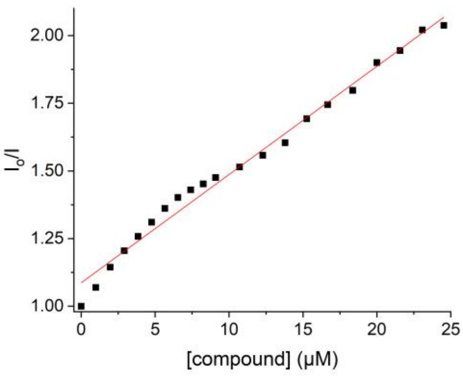

Compound 22

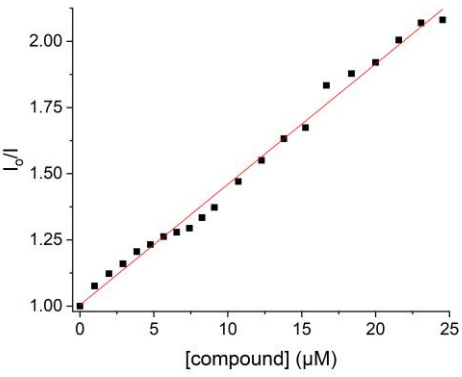

Compound 23

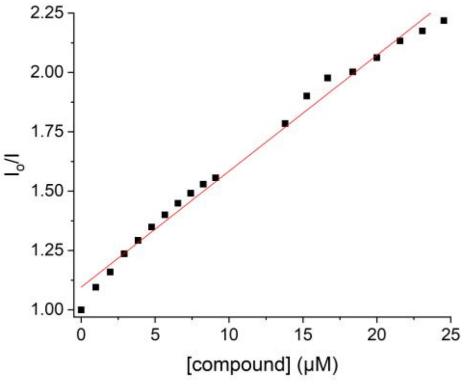

Compound 24

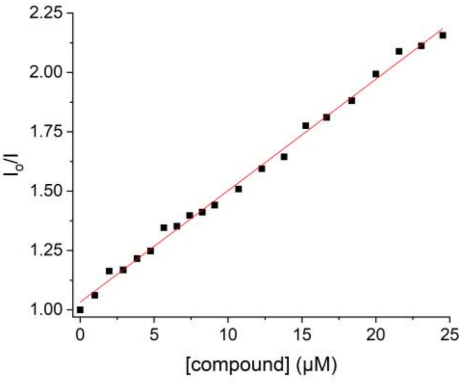

Compound 26

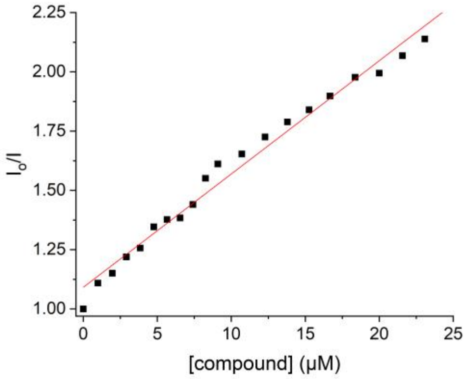

Compound 27

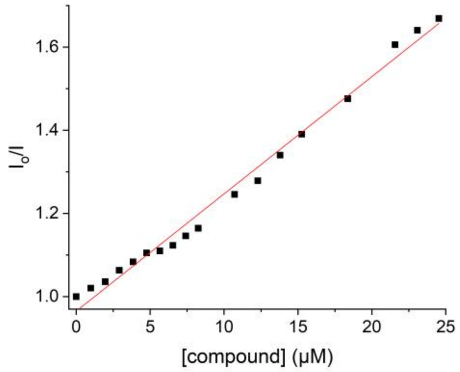

Compound 28

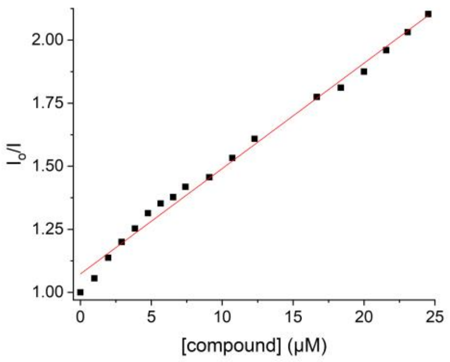

Compound 29

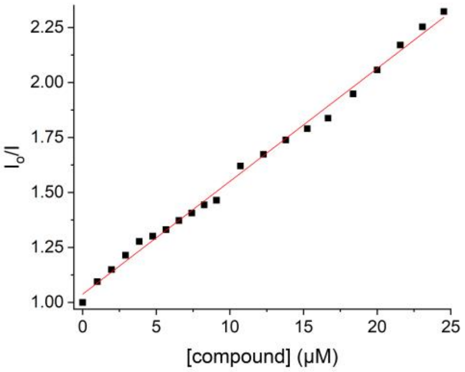

Compound 30

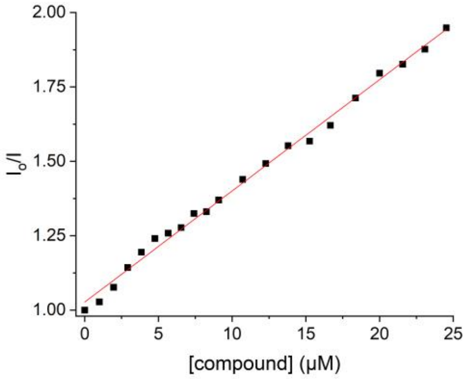

Compound 31

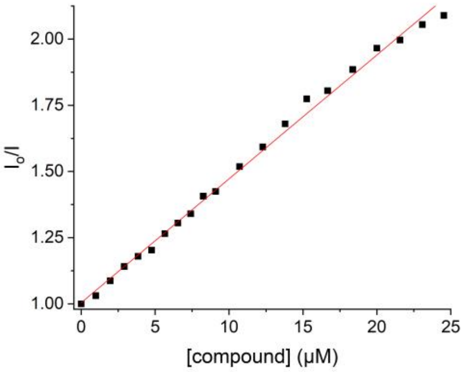

Compound 32

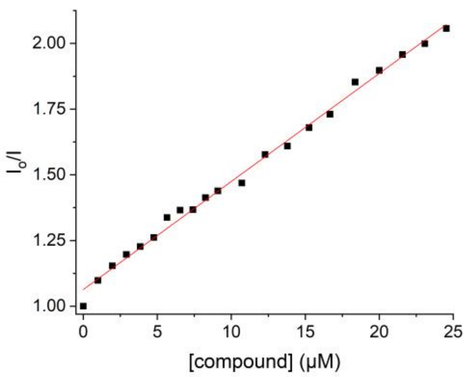

Compound 33

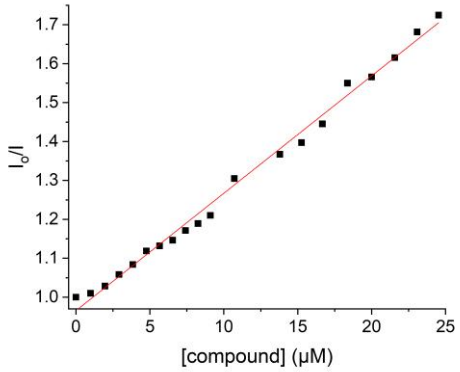

Compound 34

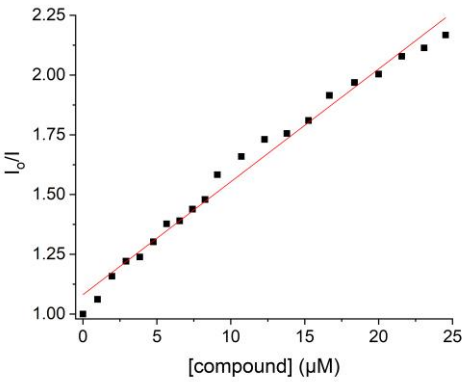

Compound 35

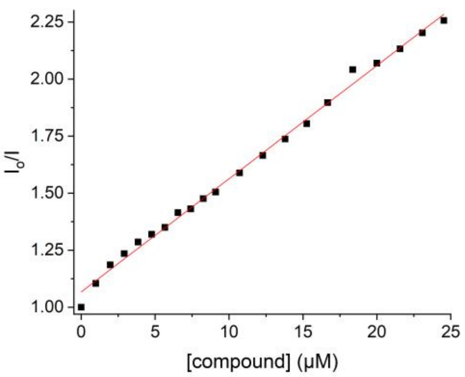

Compound 36

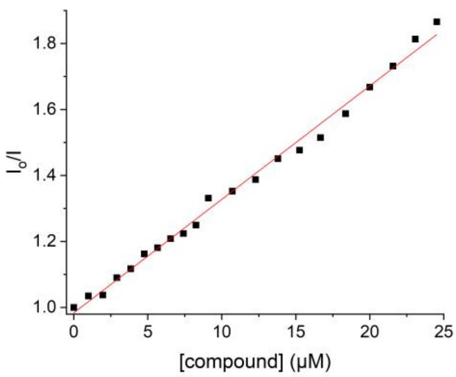

Compound 37

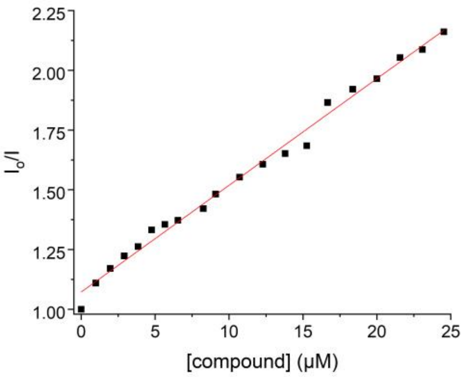

Compound 38

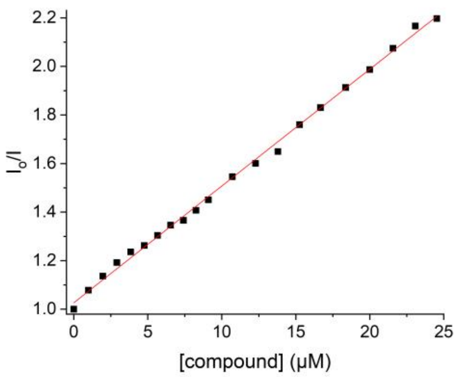

Compound 39

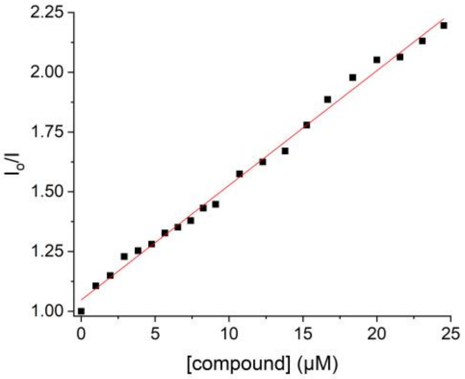

Compound 40

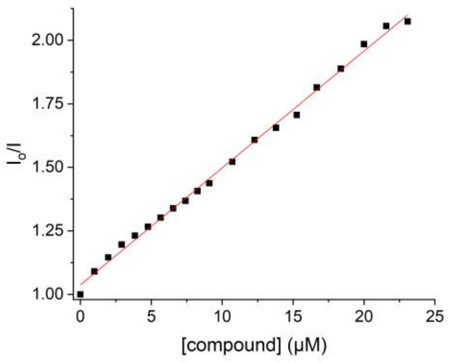

Compound 41

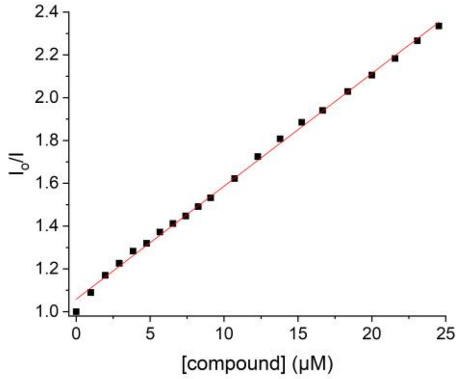

Compound 42

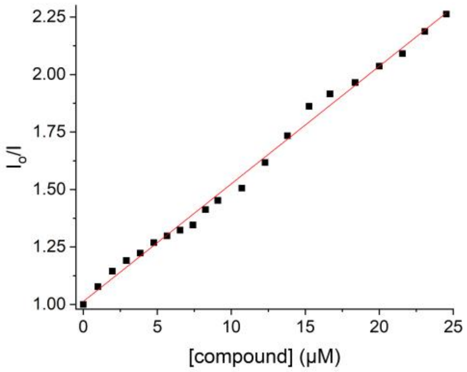

Compound 43

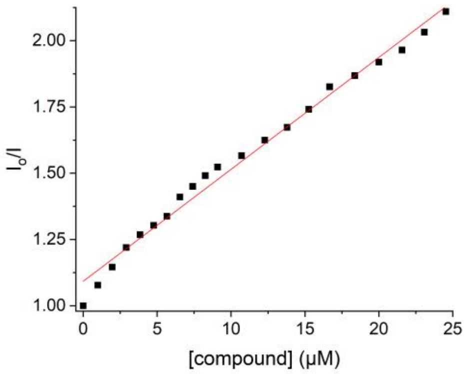

Compound 44

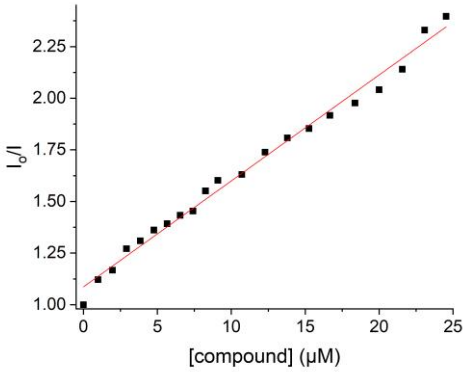

Compound 45

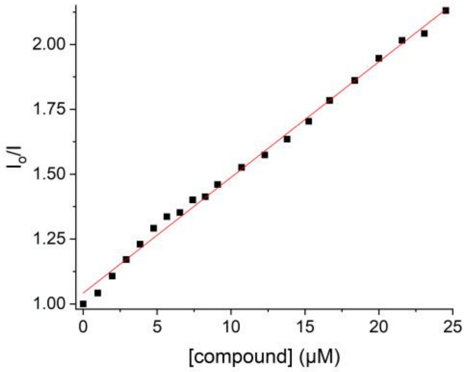

Compound 46

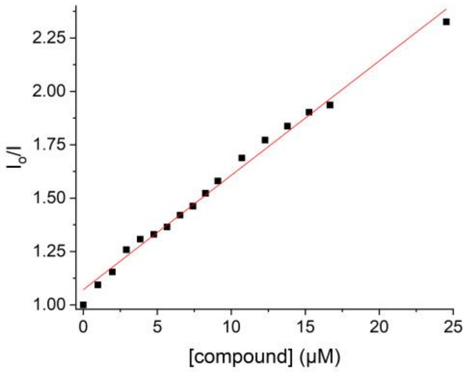

Compound 47

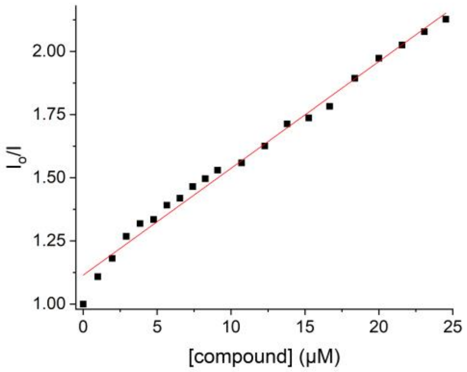

Compound 48

S.3. Fluorescence excitation and emission spectra of compounds 18-24, 26-48

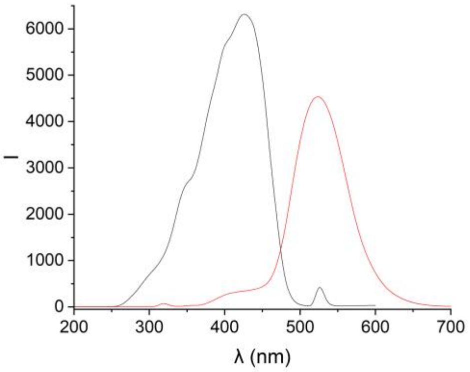

Compound 18, 315nm

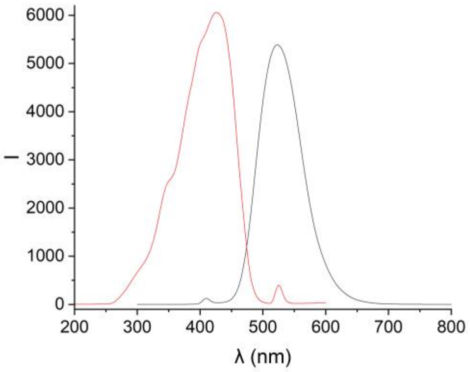

Compound 18, 406nm

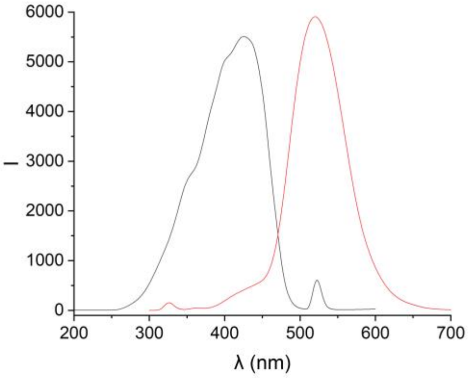

Compound 19, 322nm

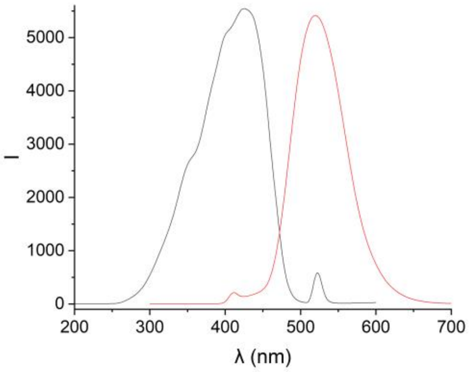

Compound 19, 407nm

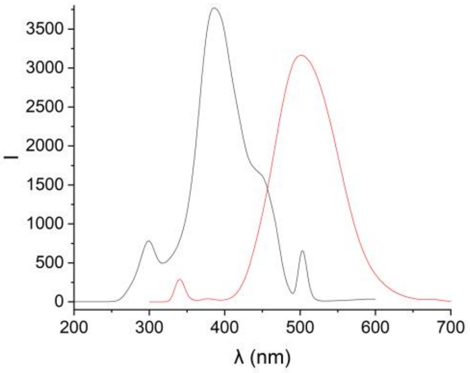

Compound 20, 336nm

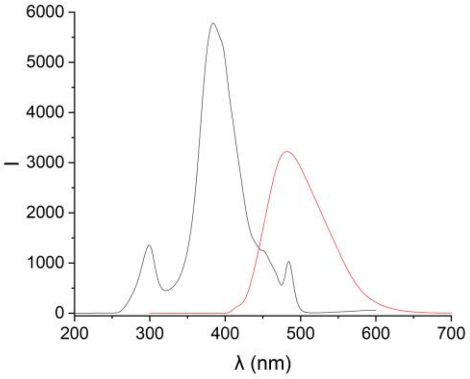

Compound 20, 412nm

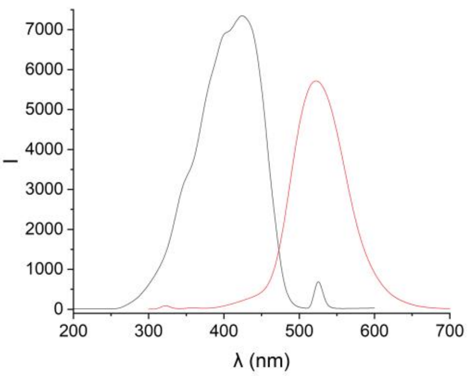

Compound 21, 318nm

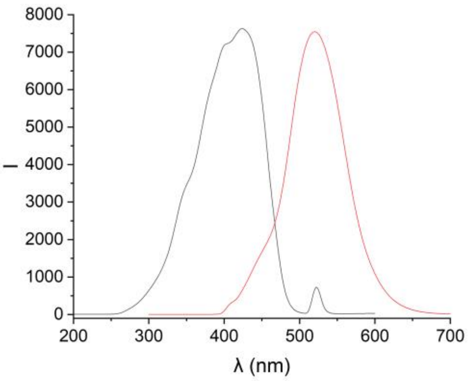

Compound 21, 404nm

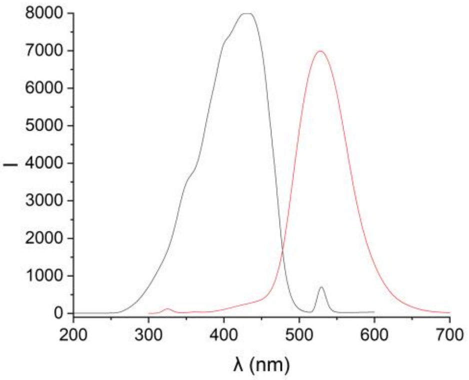

Compound 22, 321nm

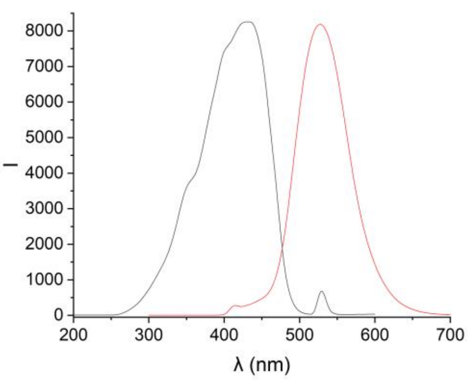

Compound 22, 409nm

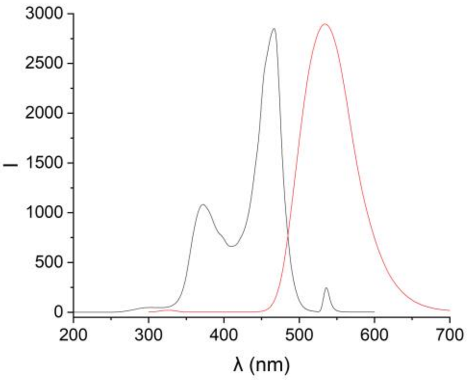

Compound 23, 319nm

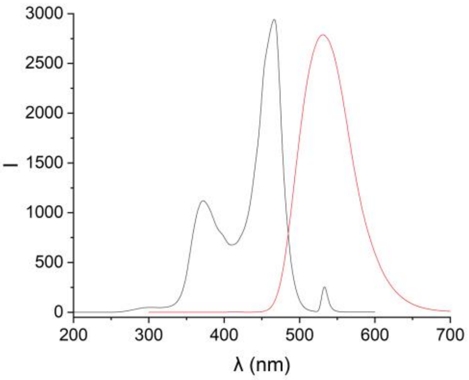

Compound 23, 410nm

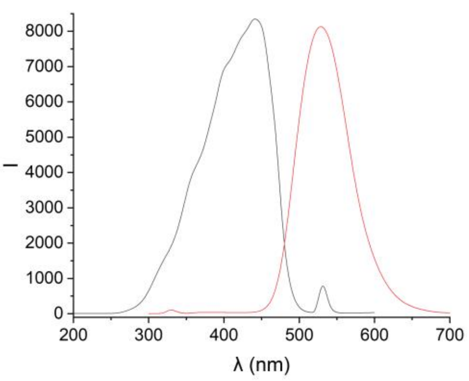

Compound 24, 326nm

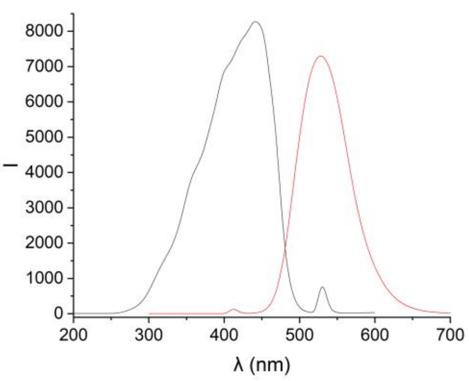

Compound 24, 409nm

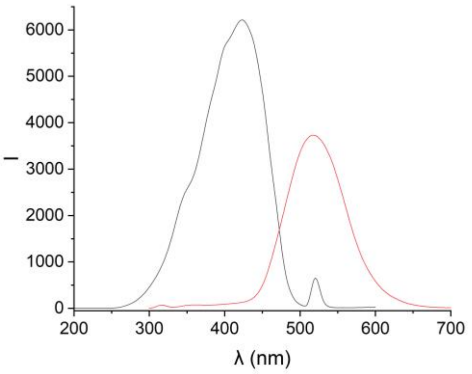

Compound 26, 312nm

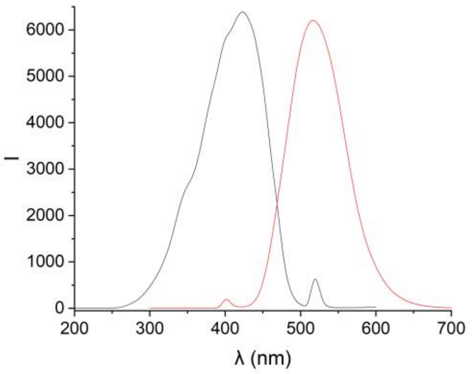

Compound 26, 398nm

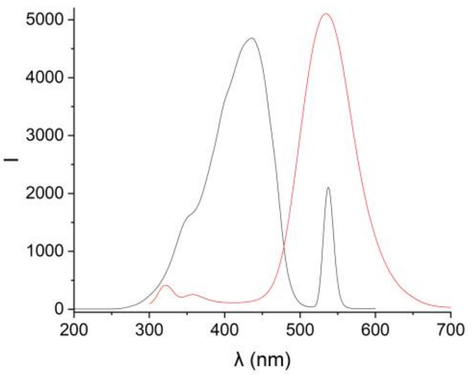

Compound 27, 312nm

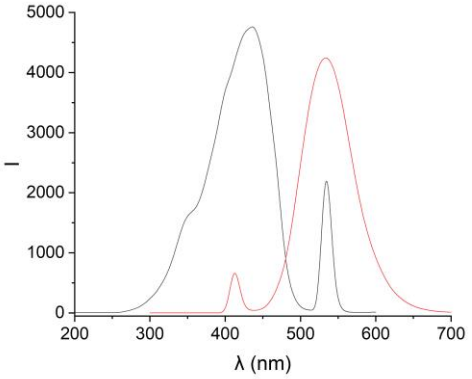

Compound 27, 409nm

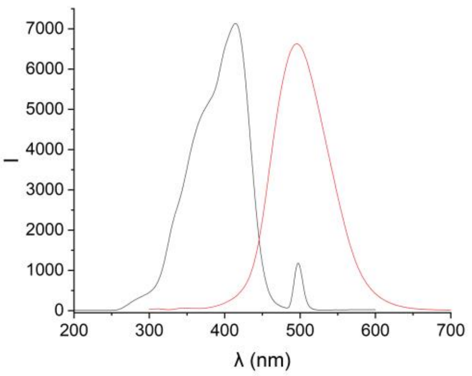

Compound 28, 301nm

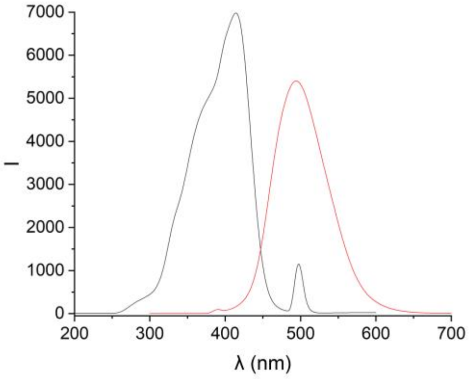

Compound 28, 385nm

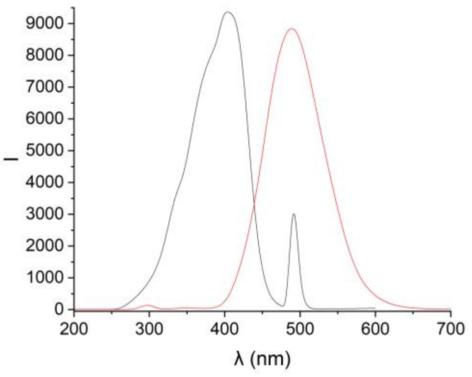

Compound 29, 291nm

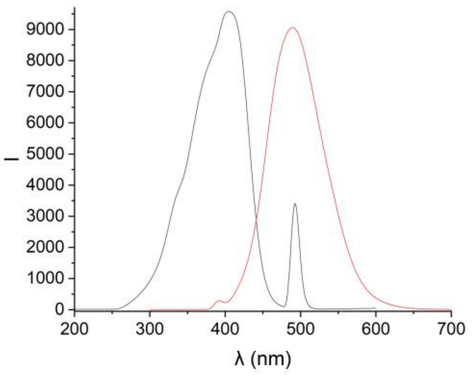

Compound 29, 387nm

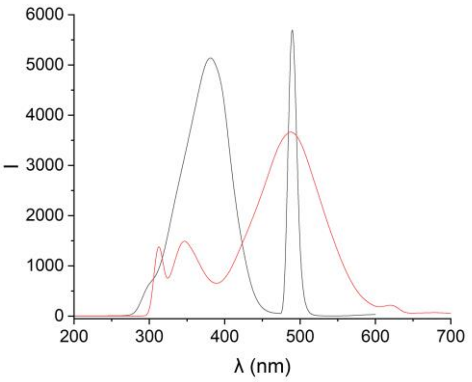

Compound 30, 308nm

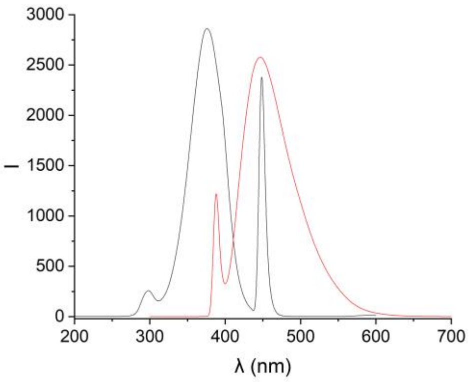

Compound 30, 385nm

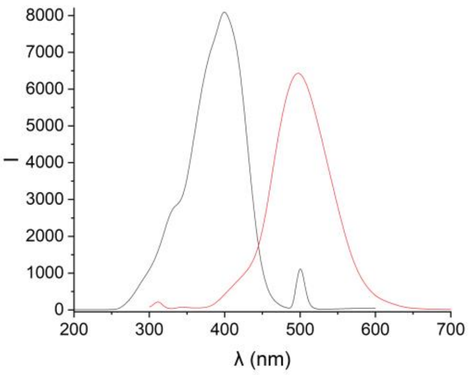

Compound 31, 307nm

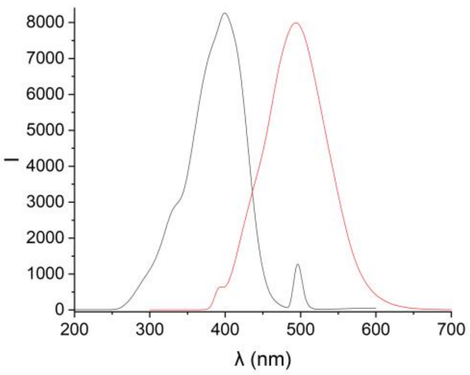

Compound 31, 387nm

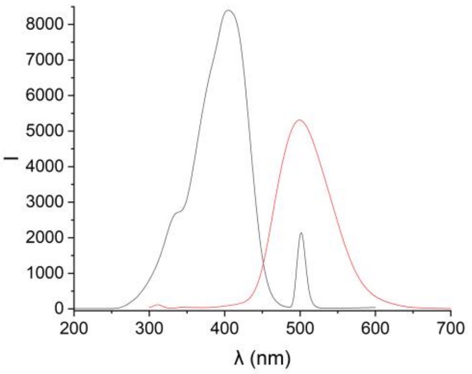

Compound 32, 307nm

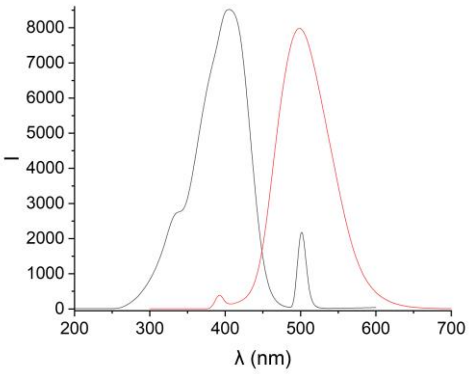

Compound 32, 388nm

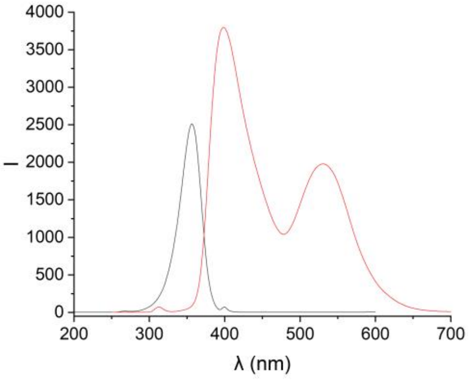

Compound 33, 309nm

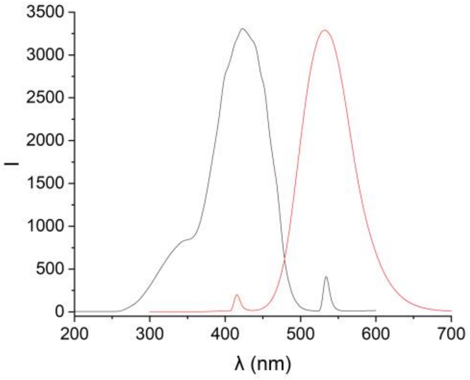

Compound 33, 387nm

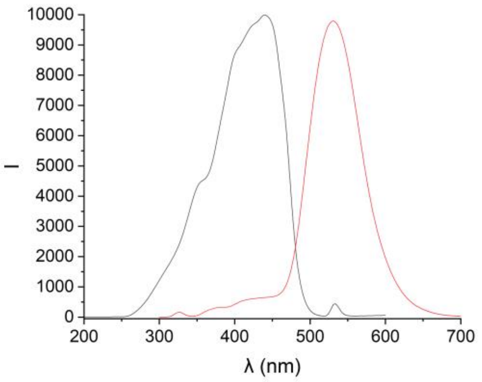

Compound 34, 322nm

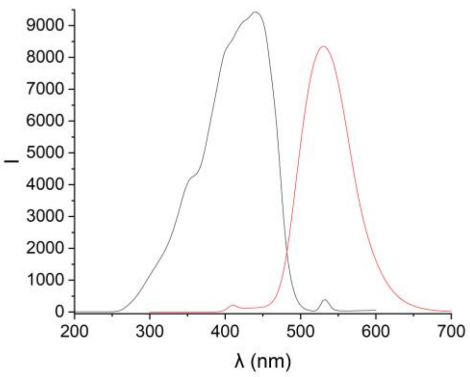

Compound 34, 406nm

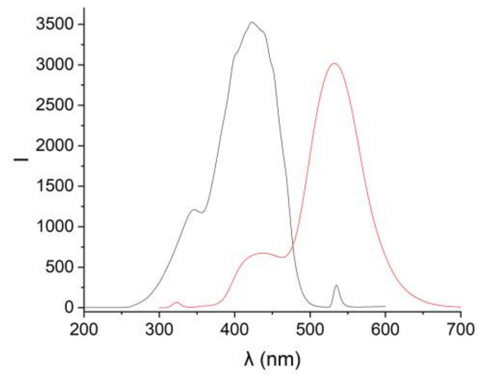

Compound 35, 307nm

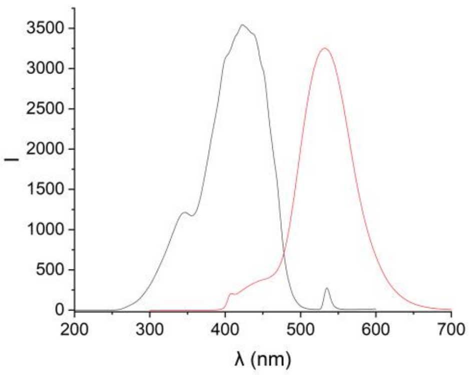

Compound 35, 404nm

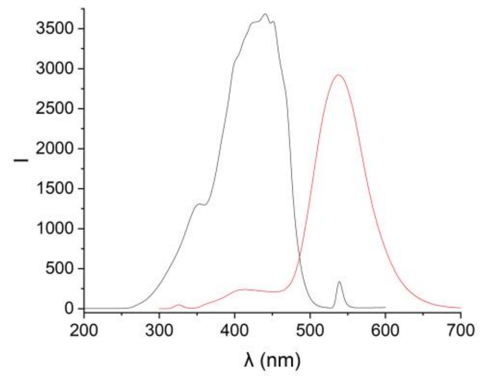

Compound 36, 321nm

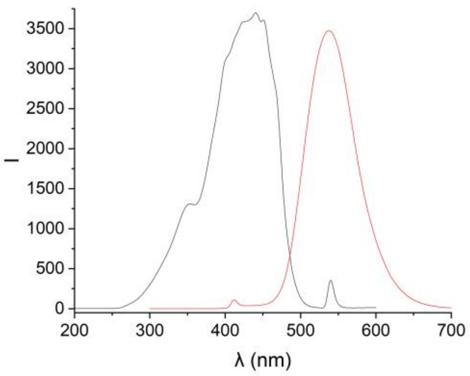

Compound 36, 409nm

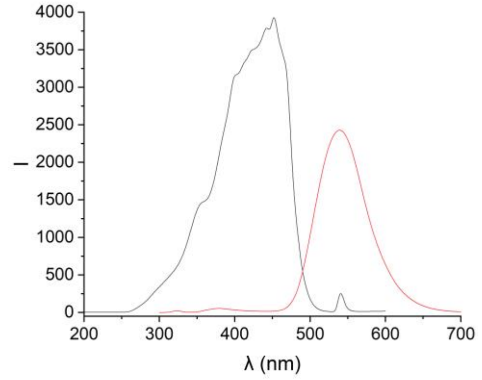

Compound 37, 319nm

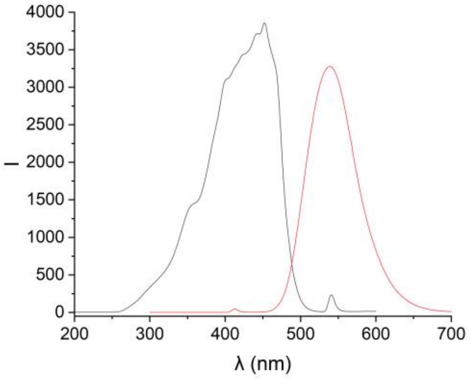

Compound 37, 410nm

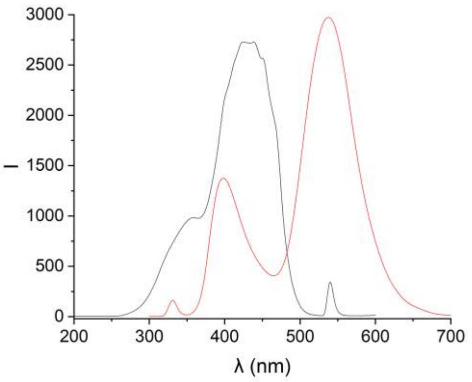

Compound 38, 327nm

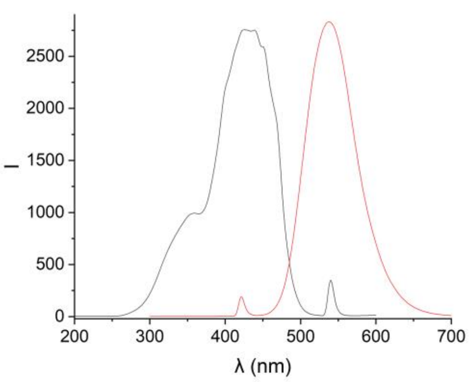

Compound 38, 419nm

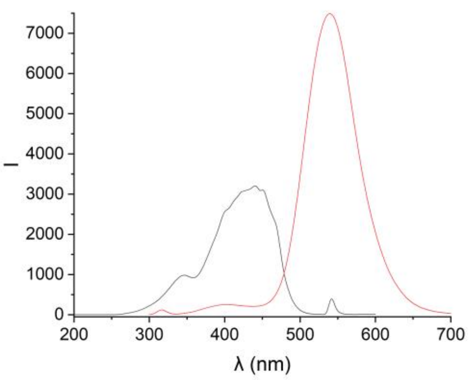

Compound 39, 312nm

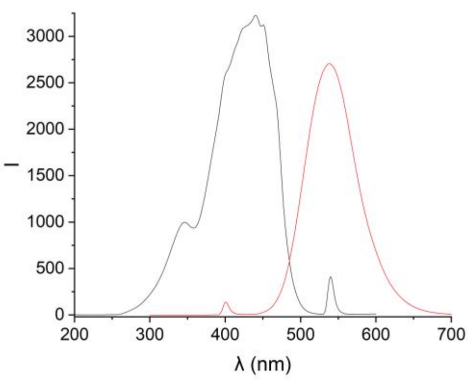

Compound 39, 398nm

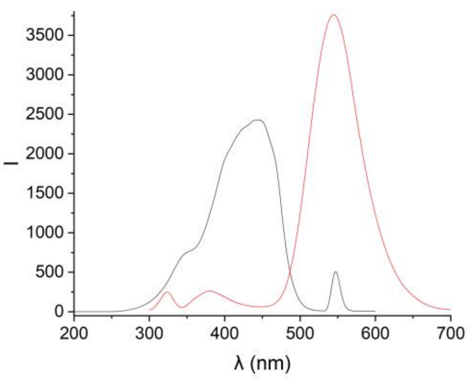

Compound 40, 314nm

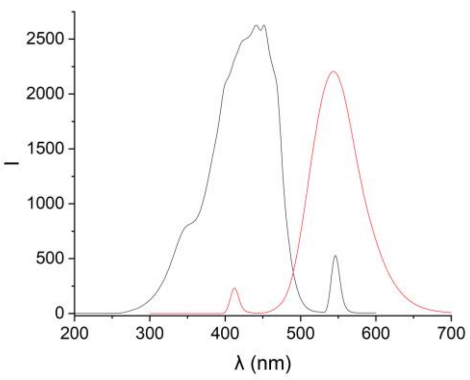

Compound 40, 409nm

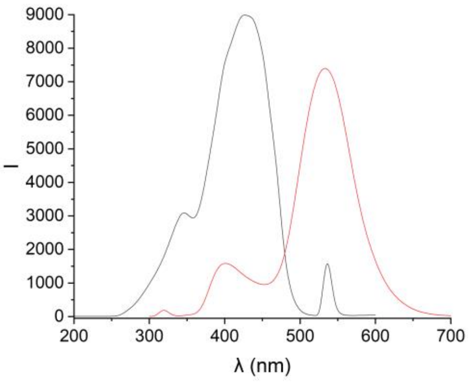

Compound 41, 315nm

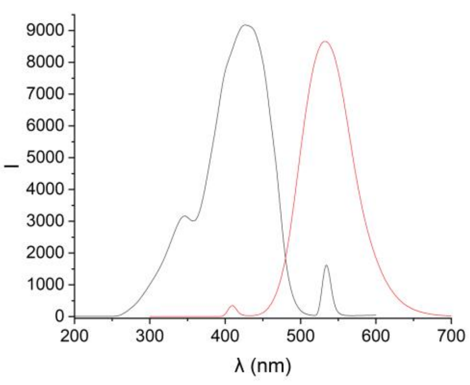

Compound 41, 406nm

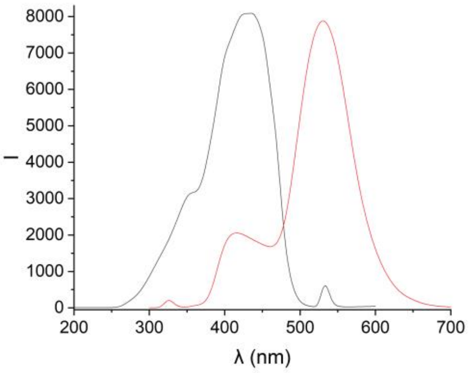

Compound 42, 322nm

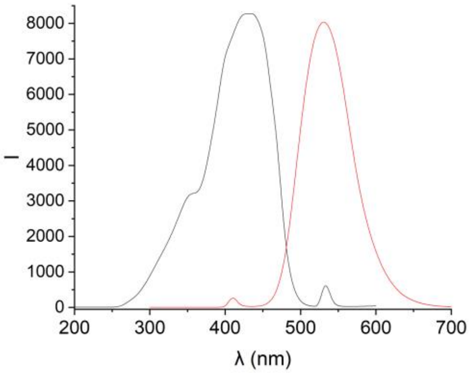

Compound 42, 407nm

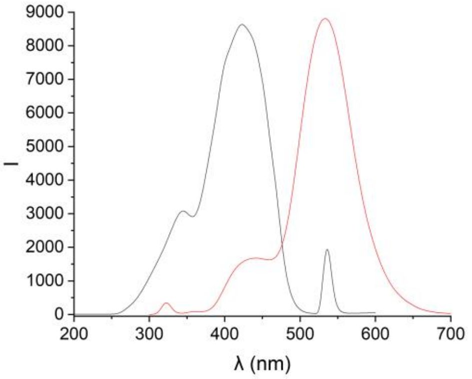

Compound 43, 318nm

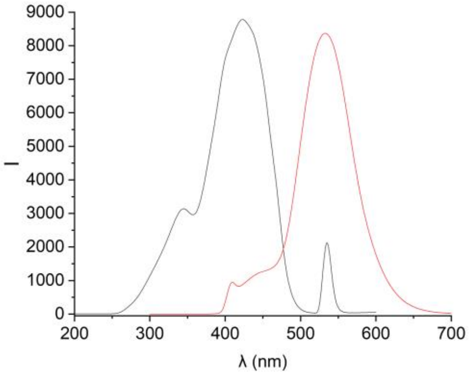

Compound 43, 409nm

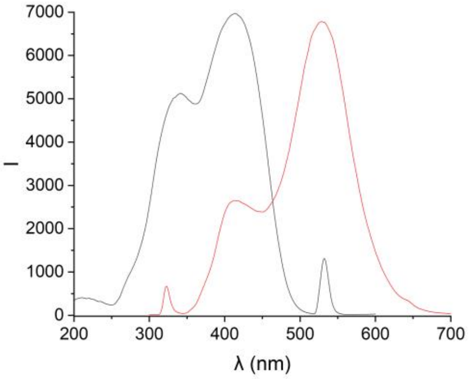

Compound 44, 320nm

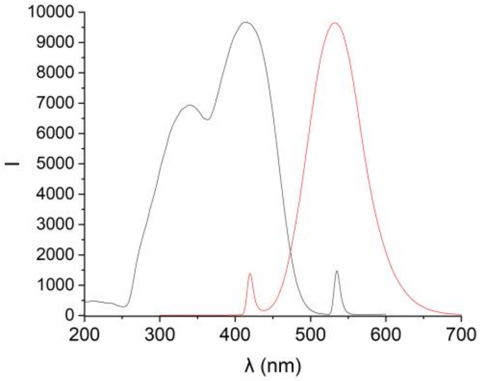

Compound 44, 417nm

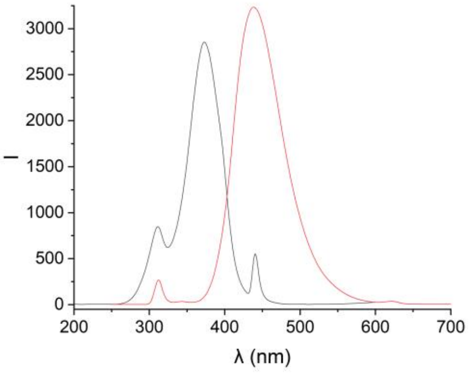

Compound 45, 307nm

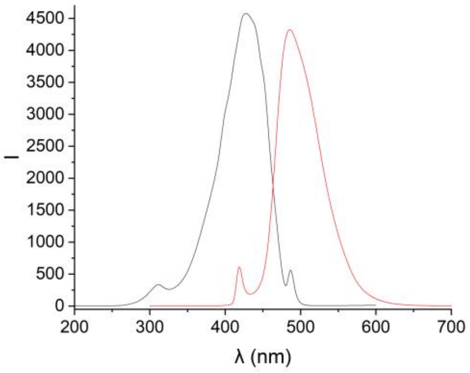

Compound 45, 416nm

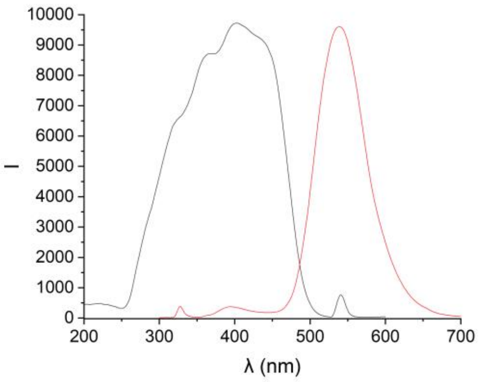

Compound 46, 315nm

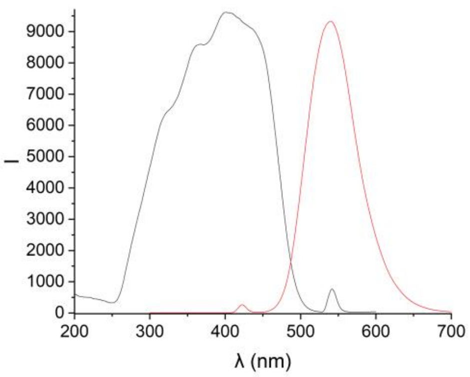

Compound 46, 419nm

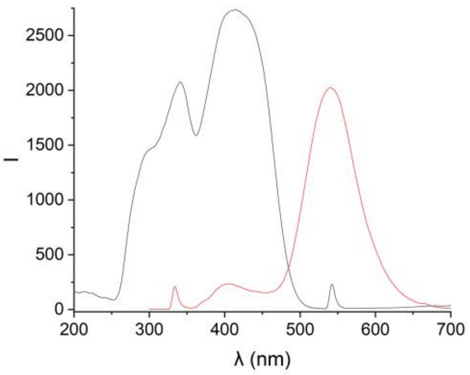

Compound 47, 331nm

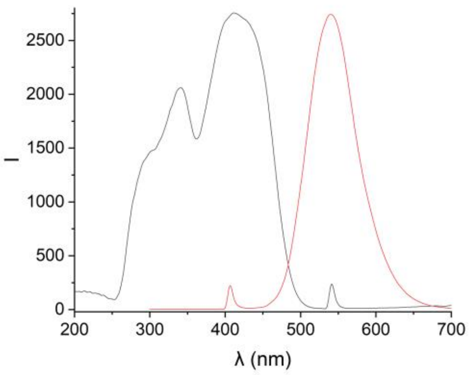

Compound 47, 404nm

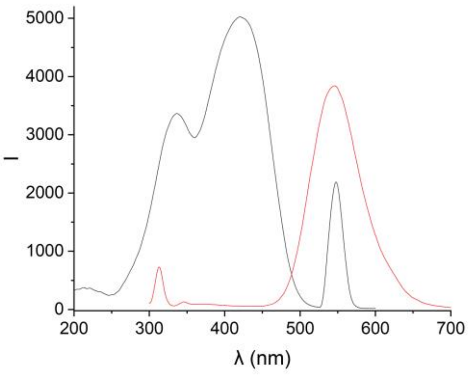

Compound 48, 308nm

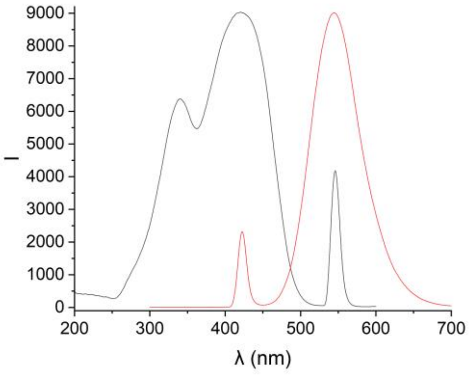

Compound 48, 419nm
